# Immune effects of faecal microbiota transplantation in amyotrophic lateral sclerosis: a pilot multicentre, randomised, masked, sham-controlled, proof-of concept trial

**DOI:** 10.1016/j.ebiom.2026.106509

**Published:** 2026-10-08

**Authors:** Elisabetta Zucchi, Elena Niccolai, Flavio De Maio, Riccardo Cuoghi Costantini, Gianluca Quaranta, Alessandra Guarnaccia, Stefano Bibbò, Ilaria Martinelli, Giulia Gianferrari, Cecilia Simonini, Giulia Nannini, Matteo Lulli, Simone Baldi, Gianluca Bartolucci, Roberto Vicini, Margherita Capasso, Giulia Bisogni, Amelia Conte, Paola Brunori, Giovanni Cammarota, Sabrina Paganoni, Roberto D'Amico, Amedeo Amedei, Luca Masucci, Jessica Mandrioli, FETR-ALS Study Group, Elisabetta Zucchi, Riccardo Cuoghi Costantini, Ilaria Martinelli, Giulia Gianferrari, Cecilia Simonini, Andrea Ghezzi, Roberta Bedin, Roberto D'Amico, Nicola Fini, Roberto Vicini, Annalisa Gessani, Francesca Prompicai, Silvia Parisi, Jessica Mandrioli, Elena Niccolai, Amedeo Amedei, Giulia Nannini, Matteo Lulli, Simone Baldi, Gianluca Bartolucci, Gianluca Quaranta, Luca Masucci, Alessandra Guarnaccia, Flavio De Maio, Giovanni Fancello, Chiara Agrillo, Vanessa Di Pietro, Desy De Lorenzis, Roberto Rosato, Francesca Romana Monzo, Giovanni Cammarota, Stefano Bibbò, Irene Venturini, Debora Rondinella, Nausicaa Antonini, Chiara Chiarini, Regina Manica, Micaela Angeletti, Marco Rossi, Tommaso Cosimo Caputo, Mario Sabatelli, Amelia Conte, Giulia Bisogni, Teresa Anna Cantisani, Paola Brunori, Rosella Papetti, Margherita Capasso, Laura Ferri, Paola Ajdinaj

**Affiliations:** a Department of Biomedical, Metabolic and Neural Sciences, University of Modena and Reggio Emilia, Modena, Italy; b Neurology Unit, Department of Neuroscience, Azienda Ospedaliero Universitaria Di Modena, Modena, Italy; c Department of Experimental and Clinical Medicine, University of Florence, Florence, Italy; d Department of Laboratory and Hematological Sciences, Fondazione Policlinico Universitario Gemelli IRCCS, Rome, Italy; e Unit of Statistical and Methodological Support to Clinical Research, Azienda Ospedaliero-Universitaria, Modena, 41121, Italy; f Department of Medical and Surgical Sciences for Children and Adults, University of Modena and Reggio Emilia, Modena, 41124, Italy; g Department of Medical and Surgical Sciences, UOC Gastroenterologia, Fondazione Policlinico Universitario Gemelli IRCCS, Rome, Italy; h Transfusion Medicine Unit, Azienda USL-IRCCS di Reggio Emilia, Reggio Emilia, Italy; i Department of Experimental and Clinical Biomedical Sciences, Biochemical Sciences Section, University of Florence, Florence, Italy; j Department of Neurosciences, Psychology, Drug Research and Child Health Section of Pharmaceutical and Nutraceutical Sciences, University of Florence, Florence, Italy; k Neuromuscular Centre, Clinica Neurologica, Ospedale “S.S. Annunziata”, Chieti, Italy; l Centro Clinico Nemo “Armida Barelli”- Fondazione Serena Onlus, Fondazione Policlinico Universitario Agostino Gemelli IRCCS, Rome, Italy; m Department of Neurophysiopathology, Perugia Hospital, Perugia, Italy; n Department of Neurology, Sean M. Healey & AMG Center for ALS and the Neurological Clinical Research Institute, Harvard Medical School, Massachusetts General Hospital, Boston, MA, USA; o Department of Physical Medicine and Rehabilitation, Harvard Medical School, Spaulding Rehabilitation Hospital, Boston, MA, USA; p Department of Basic, Clinical, Intensive and Perioperative Biotechnology Sciences, Fondazione Policlinico Universitario Agostino Gemelli IRCCS, Rome, Italy

**Keywords:** Amyotrophic lateral sclerosis, Faecal microbiota transplantation, Randomised controlled trial, T regulatory cells, Immune modulation, Gut microbiota

## Abstract

**Background:**

The gut microbiota modulates peripheral immunity, which is altered in amyotrophic lateral sclerosis (ALS). Faecal microbiota transplantation (FMT) may exert immunological effects, but its impact in ALS remains unclear.

**Methods:**

FETR-ALS was a multicentre, randomised, masked, sham-controlled proof-of-concept trial conducted at four Italian ALS centres. Adults with ALS of 18 months duration or less and forced vital capacity of at least 70% predicted were randomly assigned (2:1) to receive FMT or a sham procedure (SP). The intervention consisted of two nasojejunal administrations of screened-donor faecal suspension at baseline and month 6, without antibiotic preconditioning. The primary endpoint was change in circulating Tregs among CD4^+^ T cells at 6 months. Secondary endpoints included safety, ALS Functional Rating Scale–Revised slope, respiratory and nutritional measures, survival, and exploratory biomarker profiles. Analyses followed the intention-to-treat principle. ClinicalTrials.gov identifier: NCT03766321.

**Findings:**

Between 2020 and 2024, 43 individuals were screened and 42 were enrolled and randomly assigned to FMT (n = 28; mean age 56.1 years [SD 8.2]; 14 [50%] male) or SP (n = 14; 59.6 years [9.1]; eight [57%] male). Overall, 29 participants completed the second procedure and 6-month follow-up, and Treg measurements were available for 28. The primary outcome was not met: at 6 months, 8/18 (44%) participant receiving FMT versus 2/10 (20%) receiving SP achieved a ≥20% increase in circulating Tregs (odds ratio [OR] 3.20, 95% CI 0.59–25.36; p = 0.21). Mean Treg increase was 2.36 percentage points higher with FMT (95% CI 0.19–4.55; p = 0.04). ALSFRS-R declined by −1.07 points/month in the SP group and −0.76 points/month in the FMT group, with a between-group difference in monthly decline of 0.31 points/month favouring FMT (95% CI 0.11–0.51; p = 0.002). Conversely, at month 6, the MRC score was lower in the FMT group than in the SP group (0.75 versus 0.89; mean difference −0.13, 95% CI –0.26 to 0.00; p = 0.042). FMT was associated with 32 adverse events (12.5% severe) versus 14 (28.6% severe) in SP, mostly gastrointestinal or infectious.

**Interpretation:**

In this exploratory trial, FMT was not associated with major safety concerns. Although the primary endpoint was not met, FMT was associated with a modest increase in circulating Tregs.

**Funding:**

Italian Ministry of Health.

**Research in context**

**Evidence before this study**

To identify relevant clinical evidence, we searched PubMed, Embase, and ClinicalTrials.gov from Jan 1, 2010 to Aug 31, 2026, using the terms “amyotrophic lateral sclerosis”, “ALS”, “gut microbiota”, “microbiome”, “faecal microbiota transplantation” and “microbiota-targeted intervention”, without language restrictions. We included preclinical studies, observational human studies, systematic reviews, and interventional clinical trials evaluating the gut microbiota or microbiota-targeted interventions in ALS. Most of the available evidence consisted of experimental studies in animal models or observational human studies, which reported alterations in gut microbial composition and microbial metabolites in ALS, although findings were heterogeneous and causality remains uncertain. One randomised clinical trial of colonic-administered faecal microbiota transplantation (FMT) had been reported recently in ALS, which was early interrupted and did not demonstrate clear clinical benefit and did not include immunological endpoints. Additional microbiome-based interventional studies in ALS are ongoing, but no further randomised efficacy results were available at the time of our updated search. Overall, the available clinical evidence support an association between gut dysbiosis and ALS while highlighting substantial methodological heterogeneity, small sample sizes, limited longitudinal evidence, and the lack of definitive evidence for a disease-modifying effect of microbiota-targeted interventions.

**Added value of this study**

This multicentre, randomised, masked, sham-controlled trial provides new evidence on the biological and immunological effects of FMT in ALS. The study specifically evaluated the microbiota–immune axis by assessing circulating regulatory T cells as a mechanistic biomarker associated with slower disease progression. FMT was associated with a modest expansion of circulating Tregs and showed an acceptable safety profile. The trial further integrated longitudinal microbiota profiling, immunological analyses, and exploratory metabolic biomarkers, providing a multidimensional assessment of microbiota-based immune modulation in ALS.

**Implications of all the available evidence**

Taken together, the available evidence suggests that the gut microbiota is altered in ALS and that microbiota-targeted interventions can induce measurable biological changes, including modulation of systemic immune responses. Although the clinical efficacy of FMT remains uncertain, the observed immunological signal supports further investigation of microbiota-targeted interventions. Future studies should focus on optimising donor selection, dosing strategies, and treatment duration, and should integrate multi-omics approaches to better define patient subgroups and mechanisms of response.

## Introduction

Amyotrophic lateral sclerosis (ALS) is a fatal neurodegenerative disorder marked by significant clinical and biological heterogeneity, with limited ranges of therapeutic options that are currently yielding modest effects on disease progression and survival. Neuroinflammation plays a role in the disease pathogenesis, characterised by microglial activation, increased CD4+/CD8+ T-cell reactivity, and impaired regulatory T-cell (Treg) function.^1^ Reduced Tregs’ levels correlate with faster disease progression, highlighting the contribution of immune dysregulation.^2^

The gut microbiota (GM) is a key modulator of immune homoeostasis, including the differentiation and function of Treg, and plays a role in shaping neuroinflammatory responses through microbial metabolites (e.g., short-chain fatty acids–SCFA), vagal signalling, and immune modulation.^3^^,^^4^ Preclinical models of ALS have demonstrated early gut dysbiosis, altered intestinal permeability, and enteric inflammation.^5^, ^6^, ^7^ Moreover, interventions targeting GM, such as probiotics, antibiotics, or faecal microbiota transplantation (FMT), have shown disease-modifying effects,^6^^,^^7^ supporting a mechanistic link between microbial composition, immune regulation, and neurodegeneration. In humans, ALS is associated with reduced microbial diversity, lower Firmicutes/Bacteroidetes ratio, and loss of beneficial taxa, correlating with immune activation and metabolic alterations.^8^^,^^9^ However, clinical evidence on GM-targeted therapies remains inconclusive.^10^

We conducted an explorative pilot, randomised, multicentric, controlled trial on FMT in patients with ALS, aiming to investigate whether GM manipulation could significantly affect circulating Tregs six months after the first FMT procedure. Besides the primary immunological outcome, the safety and secondary clinical outcomes were analysed for the 12-month trial duration. Exploratory biological effects including faecal microbial composition, extensive cytokine profiling, and neurodegenerative markers, were considered to investigate the extent to which this type of intervention could affect the gut-immune-brain axis in ALS.

## Methods

### Study design

We conducted a multicentre, randomised, masked, sham-controlled, proof-of-concept trial at four academic centres in Italy: Azienda Ospedaliero-Universitaria di Modena, SS. Annunziata Hospital in Chieti, Centro Clinico Nemo Adulti–Fondazione Serena Onlus at Policlinico Universitario Agostino Gemelli IRCCS in Rome, and Perugia Hospital (Supplementary Material, FETR-ALS clinical trial sites and investigators).

Patients were enrolled between July 17, 2020 and Feb 15, 2023, and the last patient completed follow-up on Feb 15, 2024.

The study was sponsored by Azienda Ospedaliero-Universitaria di Modena and funded by the Italian Ministry of Health (Grant RF-2016-02361616). Participants received no compensation. Safety was overseen by an independent data safety monitoring board (DSMB). DSMB evaluations were scheduled after 14 patients had completed the second treatment procedure and subsequently every 6 months throughout the study. Study monitoring was performed by a certified contract research organisation (Evidenze Health, Italy). Data were collected through a secure web-based electronic case report form managed by the coordinating centre, and statistical analyses were performed by the Unit of Statistical and Methodological Support to Clinical Research at Azienda Ospedaliero-Universitaria di Modena.

The trial design, data analysis, and manuscript development were shared by the Steering Committee of the study, represented by all local PIs. The full membership of the FETR-ALS Study Group is provided in the Supplementary Material.

The authors were responsible for writing the manuscript and making the decision to submit it for publication.

All the authors guarantee data completeness and accuracy, and adherence to FETR-ALS study protocol (available as Supplementary Material) and already published.^11^

### Ethics

The study was approved by the ethics committee of the coordinating centre (Comitato Etico Area Vasta Emilia Nord; July 2018, protocol n. 0010722/18), by the ethics committees of the other three participating centres (Comitato Etico Fondazione Policlinico Universitario A. Gemelli IRCCS, protocol n. 16355/19; Comitato Etico Regionale Umbria, protocol n. 15934/19; Comitato Etico Delle Province Di Chieti E Pescara, protocol n. 14950/20), and by the Italian National Transplantation Centre (March 29, 2019, protocol n. 289/2019), as required under the Italian regulatory framework governing FMT as a transplantation of tissues and cells rather than as a medicinal product. The protocol underwent successive administrative revisions during the approval process following requests from regulatory authorities, including the National Transplant Centre and the Italian National Institute of Health (Istituto Superiore di Sanità, ISS). These revisions did not substantially modify the study design, primary endpoint, treatment schedule, or statistical analysis plan, and all occurred before enrolment of the first participant in July 2020. No protocol amendments were made after recruitment initiation. The trial was conducted in accordance with the Declaration of Helsinki and International Council for Harmonisation Good Clinical Practice guidelines. This trial is registered with ClinicalTrials.gov, number NCT03766321 (https://clinicaltrials.gov/study/NCT03766321). Registration was completed on 6 July 2018, before enrolment of the first participant. All participants provided written informed consent before screening.

### Participants

The FETR-ALS trial enrolled patients with sporadic or familial ALS, either laboratory-supported or clinically probable, or definite ALS as per the revised El Escorial criteria.^12^ Eligible participants were 18–70 years old, with disease duration of ≤18 months, forced vital capacity (FVC) ≥70% of predicted, BMI ≥18 kg/m^2^, and using effective contraception if applicable. All participants were undergoing riluzole treatment. Patients were excluded if they had received immunosuppressive or chemotherapeutic treatments within the previous two years, had autoimmune or gastrointestinal diseases, food intolerances (e.g., lactose), recent surgery or antibiotic use, active infections or organ failure, haematologic abnormalities, required non-invasive ventilation, tracheostomy, or gastrostomy, were pregnant or breastfeeding, or were participating in other clinical trials or taking experimental drugs. Further details on inclusion and exclusion criteria are available in previous publications.^11^

### Randomisation and masking

Participants were randomised in a 2:1 ratio to FMT or sham procedure (SP), to maximise safety and biological information on the experimental intervention, using a computer-generated allocation sequence created in STATA software (Release 15; StataCorp LLC, College Station, TX, USA) by an unblinded statistician from the Unit of Statistical and Methodological Support to Clinical Research, Azienda Ospedaliero-Universitaria di Modena, who was not involved in clinical assessments or participant management. Allocation was stratified according to the rate of disease progression, measured by the monthly decline in the Revised ALS Functional Rating Scale (ALSFRS-R) from disease onset to the screening visit, with a cut-off set at ≤ or > 0.7. Patients assigned to the treatment arm received two FMT procedures six months apart, performed under deep sedation. Patients in the SP arm also underwent deep sedation for gastroscopy and duodenal biopsy but did not receive FMT. These procedures were centrally performed for all recruited patients at “Foundation A. Gemelli University Hospital IRCCS, Catholic University of Sacred Heart”, Rome, Italy, by a multispecialistic team composed of an endoscopist, an anaesthesiologist, and a microbiologist. Treatment allocation was maintained in password-protected restricted-access files managed by the coordinating statistical unit and was accessible only to designated unblinded personnel involved in procedural organisation. Treating neurologists, participants, outcome assessors, laboratory investigators, and data analysts remained blinded throughout the study. Besides the planned endoscopic procedures, endoscopists, and anaesthesiologists had no role in clinical evaluations, outcome assessment, biomarker analyses, or statistical analyses. The success of masking was not formally assessed by asking participants or study personnel to guess treatment allocation.

### FMT and sham procedures

Baseline neurological and clinical evaluations were performed at the recruiting ALS centres before treatment exposure, whereas all endoscopic procedures and FMT administrations were centralised at Fondazione Policlinico Gemelli IRCCS (Rome) to ensure procedural standardisation. For logistical reasons related to centralised processing of fresh immunological samples, the procedure was performed within four weeks after baseline assessment. Peripheral blood samples were collected on the morning of the procedure and promptly shipped under standardised conditions to the University of Florence for centralised PBMC isolation and immunophenotyping analyses. Then, on the same day participants underwent upper gastrointestinal endoscopy under propofol sedation. Participants randomly assigned to the FMT group received a suspension of screened donor faeces, amounting in a total of 200 mL. The infusion was administered through a nasojejunal tube at a rate of approximately 30 mL/min, with a total infusion time of about 15 min, in order to avoid distension-related reflux while ensuring optimal endoscopic visualisation. A second FMT was administered at month 6 using the same procedure. The interval between the two FMT administrations and the infusion procedure were defined pragmatically, based on the limited available experience with FMT in neurodegenerative diseases at the time of study design and on established gastroenterological practice for nasojejunal administration, aiming to balance feasibility, tolerability, and sustained immunomodulatory effects, by analogy with the long-term documented engraftment data reported for recurrent *Clostridioides difficile* infection. Participants allocated to the SP group underwent the same endoscopic procedure under deep sedation administered by an anaesthesiologist but did not receive any infusion. This approach was used to maintain masking of treatment allocation. No antibiotic pretreatment was given before FMT.

### Stool donor screening and preparation

Because FMT is regulated in Italy under legislation governing tissues and cells for transplantation, healthy stool donors were screened according to national requirements and European directives, as previously described.^11^^,^^13^ Briefly, donor eligibility was assessed through a detailed questionnaire covering medical and family history, gastrointestinal and systemic diseases, medication use, lifestyle and behavioural risk factors, recent infections and travel, and potential exposure to transmissible diseases. Major exclusion criteria included gastrointestinal, autoimmune, neurological, neurodegenerative, or psychiatric disorders; overweight or obesity (BMI >25 kg/m^2^); recent antibiotic, immunosuppressive, or chemotherapy treatment; chronic proton-pump inhibitor use; and infectious, behavioural, occupational, or travel-related risk factors for transmissible diseases or multidrug-resistant organisms. Eligibility was reassessed on the day of each donation, and donors received no financial compensation.

Screening included blood testing for major blood-borne and transmissible infections, including HIV, hepatitis viruses, and *Treponema pallidum*, together with routine haematological, inflammatory, renal, and liver function tests. Stool testing included *C. difficile*, major enteric bacterial pathogens (*Salmonella*, *Shigella*, *Campylobacter*, *Escherichia coli* O157:H7, and *Yersinia*), multidrug-resistant organisms, norovirus, intestinal protozoa, and helminths; additional pathogen-specific testing was performed when clinically or epidemiologically indicated.^11^^,^^13^ Before processing, each donation underwent rapid molecular testing for intestinal pathogens.

Donor screening also included assessment of recent SARS-CoV-2 infection, COVID-19-compatible symptoms, and exposure to confirmed or suspected cases, as participant enrolment started during the COVID-19 pandemic. SARS-CoV-2 testing of respiratory samples was performed on the day of donation, and donors were reassessed within 30 days after donation for incident infection or compatible symptoms. Donations from donors who tested positive or developed compatible symptoms were not used.

Stool processing was performed under anaerobic conditions in a laminar flow hood. Approximately 60 g of donor stool was suspended in 150 mL of 10% glycerol solution using a Stomacher® 400 Circulator (Seward, UK) and stored at −80°C until use. Before transplantation, the material was thawed and resuspended in 200 mL of 0.9% sodium chloride solution for administration. Aliquots of the original donation and final preparation were stored at −80°C for potential subsequent analyses.^13^^,^^14^ Eleven screened donors contributed stool samples used for the two FMT procedures administered to participants during the trial.

### Clinical assessments and safety monitoring

Clinical assessments and routine laboratory tests for safety concerns were performed at baseline and at months 1, 3, 6, 7, 9, and 12. At each visit, demographic and clinical information, laboratory results, and adverse events were recorded in an electronic case report form. Participants underwent a full clinical evaluation that included the ALSFRS-R^15^ to assess functional impairment across bulbar, fine motor, gross motor, and respiratory domains. The 12 items are each scored from 0 (complete impairment) to 4 (normal function), yielding a total score ranging from 0 to 48, with higher scores indicating better function. FVC was measured in the seated position, with at least three trials performed and the highest value expressed as a percentage of predicted normal values according to age, sex, height, and weight. Quality of life was assessed using the ALS Assessment Questionnaire (ALSAQ-40).^16^ The ALSAQ-40 comprises five domains (physical mobility, activities of daily living/independence, eating and drinking, communication, and emotional functioning), each scored from 0 to 100, with higher scores indicating greater impairment and poorer health-related quality of life. Weight and body mass index (BMI) were noted at each visit. Clinical events, including initiation of non-invasive ventilation, invasive ventilation, or percutaneous endoscopic gastrostomy, were recorded during follow-up.

Safety was assessed throughout the study by monitoring adverse events, clinical status, vital signs, and laboratory parameters. Adverse events were graded according to the Common Terminology Criteria for Adverse Events (CTCAE), version 5.0,^17^ and were recorded in the electronic case report form at each visit. Serious adverse events and serious adverse reactions (SAEs and SARs) were defined according to Directive 2004/23/EC, as implemented in Italy by D.Lgs 191/2007, as previously detailed.^11^ Serious or potentially life-threatening events were reported to the sponsor within 24 h. Predefined stopping rules were established in the event of excess severe adverse events potentially related to FMT. Specifically, the trial could be stopped in the event of a 30% excess in the treatment group of predefined serious events, including peritonitis, upper gastrointestinal haemorrhage, sepsis, or bacteraemia.

Routine laboratory tests included complete blood count, creatinine clearance, electrolytes, liver enzymes, bilirubin, and lactate dehydrogenase to monitor organ function and treatment safety.

### Biological sample collection

Peripheral blood mononuclear cells (PBMC) were isolated for immunological analyses at baseline and at months 6 and 12, and serum was collected at baseline, month 3, 6, 9, and 12 for cytokine profiling, biomarker measurements, including neurofilament light and heavy chains, and short chain fatty acids assessment; lumbar puncture was optional at baseline and month 6, and available CSF samples were used for neurofilament measurements. Blood, saliva, and faecal samples were collected at baseline, and at month 6 and 12, together with duodenal biopsies at the time of endoscopic procedures. The study was conducted during the COVID-19 pandemic. While the pandemic did not lead to substantial protocol modifications, COVID-related logistical restrictions occasionally affected biological sample availability and completeness of follow-up assessments.

### T-cell immunophenotyping and intestinal FoxP3 expression

Immunological analyses were performed at the Department of Clinical and Experimental Medicine, University of Florence (Florence, Italy). Peripheral blood and intestinal biopsy samples were processed fresh on the day of collection, without cryopreservation, as previously described.^13^ PBMCs were isolated using Ficoll–Paque density gradient centrifugation. Gut mucosa-derived single-cell suspensions were obtained through mechanical and enzymatic dissociation using the GentleMACS tissue dissociator (Miltenyi Biotec, Germany) and the Tumour Dissociation Kit, human (Miltenyi Biotec, Cat# 130-095-929). Fresh intestinal biopsies were collected in Tissue Storage Solution (Miltenyi Biotec, Germany) and placed into gentleMACS C Tubes containing 4.7 mL RPMI medium (EuroClone) supplemented with 200 μL Enzyme H, 100 μL Enzyme R, and 25 μL Enzyme A (for tissues weighing 0.2–1 g). Samples were processed using a validated GentleMACS dissociation program (∼1 h) to obtain viable single-cell suspensions. Cell suspensions were then filtered through a 40 μm cell strainer, centrifuged at 1500 rpm for 10 min at 20°C, and resuspended in the appropriate buffer for downstream applications. Both PBMCs and mucosal cell suspensions were immediately used for T cell immunophenotyping by polychromatic flow cytometry (PFCM). The antibody panel included specific monoclonal antibodies (mAbs, Miltenyi Biotec), targeting CD45, (clone REA747, VioBlue®), CD3 (clone REA613, VioBlue®, Cat# 130-114-519, RRID:AB_2726687), CD4 (clone REA623, VioGreen™), CD8 (clone REA734, APC-Vio® 770, Cat# 130-110-681, RRID:AB_2659247), CD25 (clone REA570, Vio® Bright B515), CD127 (clone REA614, PE), CXCR3/CD183 (clone REA232, Vio® Bright FITC, Cat# 130-118-545, RRID:AB_2734058), CCR4/CD194 (clone REA279, PE-Vio® 770, Cat# 130-118-359, RRID:AB_2751493), CCR6/CD196 (clone REA190, APC, Cat# 130-117-375, RRID:AB_2733354), CCR10 (clone REA326, PE, Cat# 130-120-407, RRID:AB_2752080), and FoxP3 (clone REA944, Vio R667). CD45, CD4, CD25, CD127, and FoxP3 antibodies were supplied as components of the Treg Phenotyping Kit, anti-human, REAfinity™ (Miltenyi Biotec, Cat# 130-122-994). Cells were analysed using a MacsQuant Analyser flow cytometer (Miltenyi Biotec), acquiring 100,000 events per sample. The staining strategy enabled the identification of multiple CD4^+^ and CD8^+^ T-cell subsets based on chemokine receptor expression, including Th1/Tc1 (CXCR3^+^), Th2/Tc2 (CCR4^+^CCR6^-^), Th9/Tc9 (CCR4^−^CCR6^+^), Th17/Tc17 (CCR4^+^CCR6^+^), Th1–Th17/Tc1–Tc17 (CXCR3^+^CCR6^+^), and Th22/Tc22 (CCR4^+^CCR6^+^CCR10^+^) cells. Circulating Tregs were identified using intracellular staining and defined as viable CD45^+^CD4^+^CD25^+^CD127--FoxP3^+^ cells. Data were processed with FlowLogic V7 Software (Inivai Technologies, Melbourne, Australia). Intestinal Tregs were quantified by measuring FoxP3 gene expression by quantitative real-time PCR. Total RNA was extracted from intestinal biopsies preserved in nucleoprotect RNA (Macherey–Nagel, Germany) and stored frozen until processing reverse-transcribed into cDNA, and FoxP3 expression was quantified using the ΔΔCt method with β2-microglobulin (B2M) as the endogenous control. For B2M, the following primer sequences were used: forward, 5′-AGTATGCCTGCCGTGGAAC-3′, and reverse, 5′-GCGGCATCTTCACAAACCT-3′. FoxP3 amplification was performed using the Hs_FOXP3_1_SG QuantiTect Primer Assay (Qiagen, Germany; Cat. No. 249900). This approach was used because the number of FoxP3^+^ cells in intestinal mucosa was insufficient for reliable flow-cytometric quantification. Further methodological details have been previously reported.^13^

### Cytokine profiling

Cytokine concentrations were quantified in serum, saliva, faeces, and gastrointestinal biopsies using multiplex bead-based immunoassays. Serum samples were additionally collected at months 3 and 9, whereas saliva, faecal samples, and gastrointestinal biopsies were collected at baseline, month 6, and month 12, as specified by the study protocol.^11^ Cytokines were measured in the residual CSF samples available after completion of the other planned biomarker analyses. Because lumbar puncture was optional and the residual sample volume was limited, CSF was available for only a small subset of participants at baseline and month 6. Moreover, concentrations of several cytokines were below the lower limit of quantification. The resulting data were therefore analysed descriptively. A Luminex assay was used to assess systemic and intestinal inflammatory profiles using Bio-Plex Pro™ Human Cytokine magnetic bead assays (Bio-Rad Laboratories), comprising the Bio-Plex Pro Human Cytokine 17-plex Assay (Cat# M5000031YV), supplemented with the Bio-Plex Pro Human Cytokine IL-15 Set (Cat# 171B5013M), MIP-1α Set (Cat# 171B5022M), and VEGF Set (Cat# 171B5027M). The panel detected granulocyte colony-stimulating factor (G-CSF), granulocyte macrophages colony-stimulating factor (GM-CSF), interferon-gamma (IFN-γ), interleukins (IL-1β, IL-2, IL-4, IL-5, IL-6, IL-7, IL-8, IL-10, IL12p70, IL-13, IL-15, IL-17A), monocyte chemotactic protein-1 (MCP-1), macrophage inflammatory protein-1 alpha (MIP-1α) and beta (MIP-1β), tumour necrosis factor-alpha (TNF-α), and vascular endothelial growth factor-A (VEGF-A) (BioRad Laboratories). Cytokine levels were estimated using a five-parameter polynomial curve with Bio-Plex Manager Software (Bio-Rad Laboratories).

### Fatty acid analysis

Additional exploratory analyses included measurement of serum free fatty acids (FFA) using gas chromatography–mass spectrometry (GC–MS) at the Department of Neurosciences, Psychology, Drug Research and Child Health Section of Pharmaceutical and Nutraceutical Sciences, University of Florence, Florence. Serum levels of short chain fatty acids (SCFAs: acetic, propionic, butyric, isobutyric, 2-methylbutyric, isovaleric and valeric acids), medium chain fatty acids (MCFAs: hexanoic, isohexanoic, octanoic, decanoic and dodecanoic acids), and long chain fatty acids (LCFAs; tetradecanoic, hexadecanoic and octadecanoic acids) were measured according to a previously described GC–MS protocol.^18^ Briefly, 200 μl of serum sample was mixed with 10 μl of internal standards (ISTD) mixture, 100 μl of tert–butyl methyl ether, and 20 μl of 6 M HCl + 0.5 M NaCl solution in a 0.5 mL centrifuge tube. The mixture was vortexed for 2 min and centrifuged at 10,000 rpm for 5 min. The solvent layer was transferred to a vial with a microvolume insert for GC–MS analysis. The GC–MS system consisted of an Agilent HP 5890 gas chromatograph, coupled to an HP 5971 single-quadrupole mass spectrometer, and equipped with an HP 7673 autosampler.

### Neurofilaments analysis

Neurofilaments concentrations were measured at the Laboratory of Neuroimmunology of the University of Modena and Reggio Emilia, and AOU Modena (Modena, Italy). CSF and serum samples were collected, processed, and stored at −80°C, until analysis. Neurofilament heavy (NfH, catalogue number SPCKB-PS-013074) and light chain protein (NfL, Cat# SPCKB-PS-002448) in CSF and serum were quantified using the Ella Automated Immunoassay System (Simple Plex, R&D Systems, Minneapolis, MN, USA), as previously described.^19^ Each sample was analysed in triplicate.

### Gut microbiota sequencing and bioinformatic analysis

Gut microbiota characterisation was performed at the Department of Microbiology, Policlinico Universitario “A. Gemelli”, Rome, by a microbiologist blinded to treatment allocation. Freshly collected faecal samples were placed in sterile collection tubes and stored at −80°C until processing. DNA extraction was performed under strictly controlled biosafety level 2 conditions. According to a previously described protocol,^20^ 200 mg of each faecal sample was suspended in hexadecyltrimethylammonium bromide (CTAB) buffer, and bacterial DNA was extracted using the DANAGENE MICROBIOME Faecal DNA kit (Danagen-Bioted, Barcelona, Spain). DNA was eluted in 200 μL of preheated nuclease-free water and stored at −20°C until further processing.^21^^,^^22^ DNA concentration was measured using a Qubit 4.0 fluorometer (Life Technologies, California, USA). The V3, V4, and V6 hypervariable regions of the 16S rRNA gene were amplified using Microbiota and Solution B ((AD-002.024, CE-IVD, Arrow Diagnostics, Genova, Italy), as previously described.^22^^,^^23^ Extracted DNA (5.0 ng) was used as template in a final PCR volume of 20 μL containing Enzyme Mix 1 and Amp Mix solutions. Thermal cycling conditions were as follows: (i) 95°C for 5 min; (ii) 25cycles, each consisting of 95°C for 30 s, 55°C for 30 s, and 72°C for 30 s and (iii) 72°C for 5 min.

Amplicons were purified using 29.5 μL of Agencourt AMPure XP beads (Beckman Coulter), previously diluted in 19.0 μL of nuclease-free water, and eluted in 35 μL of nuclease-free water. For each sample, 10 μL of Enzyme Mix 2 and 8 μL of nuclease-free water were mixed, and the resulting suspension was used to resuspend the assigned lyophilised index. Two microlitres of the previously generated amplicon were then added to each recovered index. Thermal cycling conditions were as follows: (i) 95°C for 5 min; (ii) eight cycles of 95°C for 30 s, 65°C for 30 s, and 72°C for 30 s; and (iii) 72°C for 5 min. Indexed amplicons were purified using Agencourt AMPure XP beads as described above and eluted in 25 μL of 10 mM Tris/1 mM EDTA (pH 8.0). Amplicon quality was assessed by 1% agarose gel electrophoresis (Thermo Fisher), and DNA concentration was determined as described above. The final amplicon length was approximately 900 bp.

Indexed amplicons from each sample were pooled at equimolar concentrations, and the final library (5 pM) was subjected to 2 × 250-bp paired-end sequencing on an Illumina MiSeq instrument.^21^ PhiX v3 internal control (Illumina, California, USA) was added to the final library to increase sequence diversity.

Raw sequencing data were analysed using MicrobAT (Microbiota Analysis Tool), version 1.1.0 (SmartSeq Bioinformatics). Briefly, raw paired-end reads exported as FASTQ.gz files were quality-filtered to remove reads shorter than 200 nucleotides or with a mean Phred quality score <25. Taxonomic assignment was performed by aligning the retained sequences against the Ribosomal Database Project (RDP) reference database (version 11.5). Sequences with an alignment covering ≥80% of their length and a similarity threshold of ≥97% were assigned to the species taxonomic level.^24^ A biological observation matrix (BIOM) was subsequently generated at different taxonomic levels.

Downstream analyses were performed in R (version 4.0.2) using the phyloseq package (version 1.46)^25^ and an in-house analysis pipeline.^26^ Unclassified bacterial and archaeal features were excluded. Low-abundance features were also removed by retaining only those detected at least three times and present in at least 20% of samples. To minimise differences in sequencing depth, samples were normalised to the median sequencing depth for alpha-diversity and ordination (beta-diversity) analyses.

Alpha diversity was assessed using observed richness, the Shannon diversity index, and Pielou's evenness. Beta diversity was evaluated using Bray–Curtis dissimilarities and visualised by principal coordinates analysis (PCoA). Relative abundances were calculated at the phylum and genus levels.^27^

Differences in alpha-diversity metrics were assessed using the Wilcoxon signed-rank test for paired data. Differences in beta diversity were evaluated by permutational multivariate analysis of variance (PERMANOVA) using the *adonis* function in the vegan package (version 2.6-4). Differences in multivariate dispersion were assessed using PERMDISP with the *betadisper* function. Differences in relative abundance were assessed using the Wilcoxon signed-rank test for paired data. Differential abundance analysis was performed using DESeq2 (version 1.40.2), and results were visualised using volcano plots.^28^

### Outcomes

The primary outcome was the proportion of participants showing a positive biological response at 6 months, defined as a ≥20% increase in peripheral Tregs frequency (defined as CD4+CD25+CD127^−^FoxP3+ T cells, and considered as Tregs% over CD4+ T cells throughout the paper) from baseline, comparing the FMT group with the SP group. Key supportive analyses included the between-group difference in the change in peripheral Tregs from baseline to month 6, analysed as a continuous variable, and the proportion of participants showing any increase in Tregs at month 6 in the FMT group compared with the SP group.

Secondary outcomes included changes from baseline to each time point (months 1, 3, 6, 9, and 12) in the ALSFRS-R and FVC, and tracheostomy-free survival from randomisation, safety, and quality of life assessed as changes from baseline in the ALSAQ-40 at month 6 and 12.

Exploratory biological outcomes included between-group comparisons of changes from baseline at each time point in T-cell distribution, with particular focus on the Tregs/Th1 and Tregs/Th17 ratios; cytokine profiles in serum, CSF, gut mucosa, saliva, and feces; serum free fatty acid levels (FFA); and peripheral and CSF biomarkers, including creatinine, albumin, and neurofilament light and heavy chain proteins. Changes in faecal microbiota composition from baseline to months 6 and 12 were also assessed.

Given the exploratory nature of the trial, no formal hierarchical testing strategy was prespecified, and p values for secondary and exploratory analyses were therefore considered nominal.

### Sample size calculation

Sample size estimation was based on an anticipated difference in the proportion of patients achieving a ≥20% increase in Tregs at month 6, which was considered a clinically meaningful biological response and potentially achievable target consistent with partial restoration toward the immunoregulatory profile observed in slower disease progression at the time of study design. This assumption was informed by prior evidence indicating that Treg levels are approximately 31% lower in fast-progressing ALS and are inversely correlated with disease progression.^1^^,^^29^ For the purpose of the sample size calculation, we specified a target absolute difference of 45% in responder rates between treatment groups, hypothesising that 50% of FMT-treated patients would achieve this response compared with 5% of controls. With 80% power and a two-sided α of 0.05 (accounting for multiple comparisons), the target sample size was 39 participants, randomised in a 2:1 ratio. Allowing for an anticipated 10% dropout rate, the planned enrolment was 42 patients.

### Statistics

The primary population for analyses was the intention-to-treat (ITT) cohort, including all participants who were randomised. All randomised participants allocated to the treatment arm received at least one FMT. A per-protocol (PP) analysis was also performed excluding non-compliers (patients who did not perform the second round of FMT) and dropouts for any reason. Continuous variables were summarised as means (SD) or medians (IQRs), and categorical variables as counts and percentages.

The primary outcome, i.e. the proportion of positive responders (≥20% Tregs increase), was compared between groups using generalised linear models, with results expressed as odds ratios (ORs) and relative risks (RRs) with 95% CIs. Moreover, Cohen's h effect size for the primary endpoint was also reported.

Mean absolute differences from baseline to month 6 and other time points among treatment arms for different T cell subpopulations, biomarkers (neurofilaments and all the measured laboratory parameters), and cytokines, were calculated and compared using linear regression models that included indicator variables for treatment arms as the independent variables. Results were expressed as the mean difference (MD) comparing treatment arms. For outcome variables characterised by a strongly skewed distribution, a logarithmic transformation was applied prior to regression modelling. In these cases, results were reported as ratio between geometric mean of FMT group and geometric mean of control group. Correlations between numerical variables were calculated with the Pearson's linear correlation coefficient. The time to death, tracheostomy or permanent ventilation, were compared by using the log-rank test. Cox Proportional hazard models were used to estimate hazard ratios (HR) between groups. The assumption of proportionality of the hazard was checked by analysing the Schoenfeld residuals.

Linear mixed models were used to analyse repeated measurements on biological and clinical outcomes, when appropriate. For each parameter measured over time, a mixed effect model was fitted, which included a random intercept in order to take into account the correlation among observations taken on the same individuals. These models included as covariates arm, time (months from baseline) and the arm × time interaction.

In addition, a segmented repeated measures linear mixed model analysis was carried out to assess whether the average monthly variation from baseline in selected numerical outcomes was different amongst treatment arms in different segments of time In these models we considered four segments of time: from baseline (first FMT/placebo) to month 1, from month 1 to month 6, from month 6 to month 7 (suddenly after the second FMT/placebo administration), and finally from month 7 to month 12. When applied to ALSFRS-r data, an additional segment was included in the model, to take into account the pre-baseline ALSFRS-r variation over time, hence considering a total of 5 segments of time. The dependent variables were the raw measurements of the outcomes, whereas the independent variables were: arm, time (months from baseline) × period (during or after treatment) interaction, and arm × time × period interaction. A random intercept term was also used to account for repeated measurements over the same individual, as well as a random slope term was used to account for individual linear variations over time. Within-subjects correlation among repeated observations was modelled using an unstructured covariance matrix. To estimate mixed-effects models parameters, Restricted maximum likelihood (REML) method was used. Results of these analyses were expressed as: the monthly variation of outcomes in the control group; the MD in the monthly variations, comparing treatment arm versus control arm. Both these quantities were reported for all segments of time considered for each outcome.

For the comparison of FMT and SP arms and, when applicable, for all other statistical measures, a p value of 0.05 or less was considered to indicate statistical significance and uncertainty in results was expressed with the 95% CI. CIs for linear models were calculated based on the exact t distribution. For linear mixed models, the Satterthwaite's method for degrees of freedom was used, with Kenward-Roger method used for a sensitivity analysis on the endpoints at 6 months. All statistical tests were two-tailed. No formal adjustment for multiplicity was applied for secondary and exploratory analyses. Missing values were excluded from the analyses and the number of analysed individuals in each arm was reported. As sensitivity analysis, different imputation strategies were considered for the analysis of the primary outcome. These strategies included: 1) imputation of missing values as non-responders; 2) imputation of missing values in control arm as responders, and imputation of missing values in treatment arm as non-responders; 3) Multiple imputation by chained equations (MICE), based only on control group data; 4) MICE imputation based only on arm.

Missing values were due to Covid-19 pandemic, and, for secondary biological outcome measures, also to the reduced amount of material of biological samples for difficulty in blood withdrawal; for FVC they were due to increasing patients’ difficulties to perform spirometry with advancing respiratory or bulbar impairment.

Analyses were performed using STATA software, version 18 (StataCorp. 2023. Stata Statistical Software: Release 18. College Station, TX: StataCorp LLC) and R software, version 4.3.2 (The R Foundation for Statistical Computing, Wien).

### Role of funders

The funder had no role in the design and conduct of the study; collection, management, analysis, and interpretation of the data; writing, review, or approval of the manuscript; and decision to submit the manuscript for publication.

## Results

The study design and participant flow-chart is visualised in Fig. 1. All analyses reported in this paper were performed in the ITT population. Baseline demographic and clinical characteristics are reported in Table 1; baseline biomarker levels are reported in Supplementary Tables S1 and S2. Primary and secondary efficacy outcomes in the ITT population at month 6 are reported in Table 2.

**Fig. 1:**
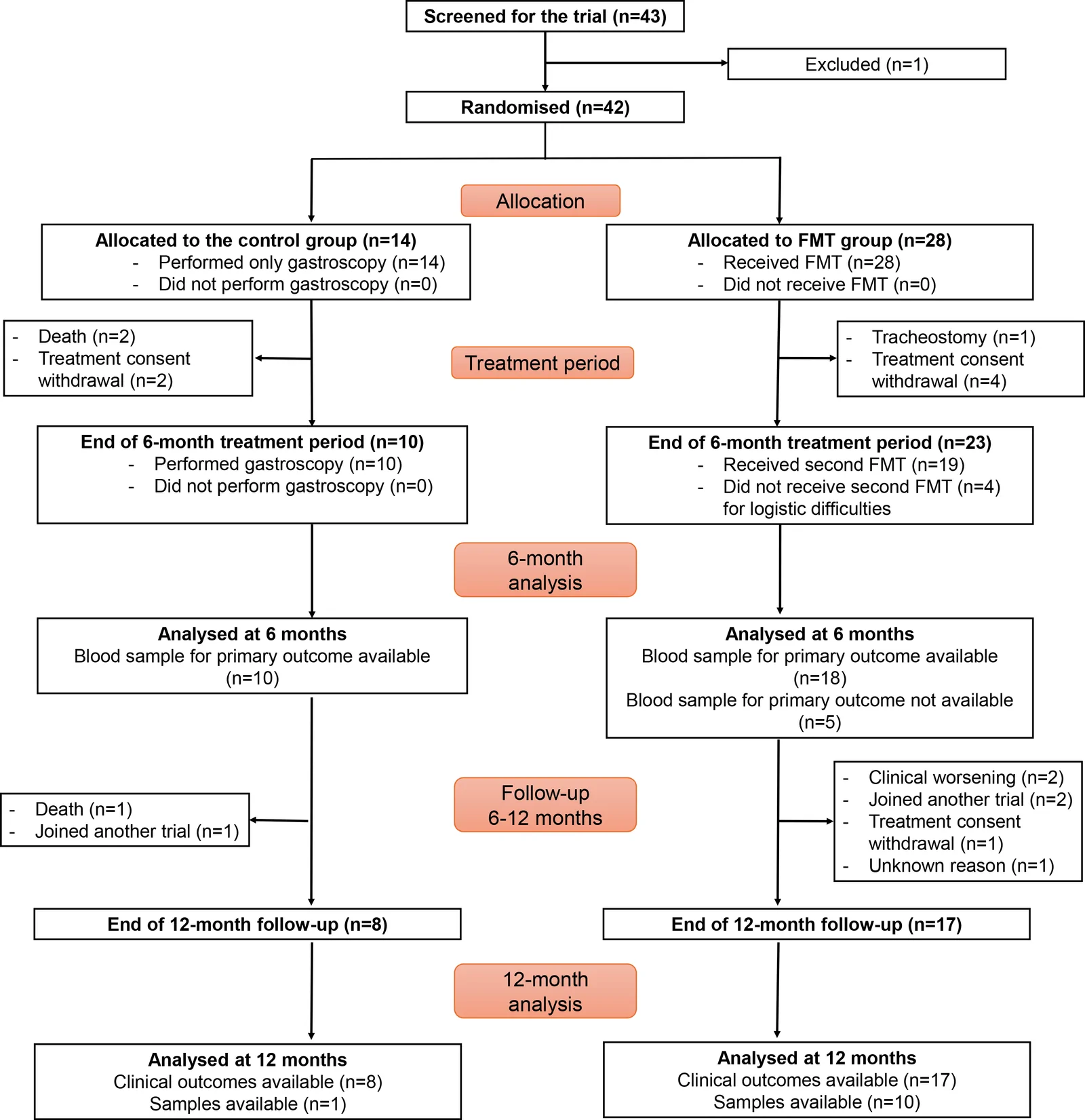
**CONSORT diagram of the FETR-ALS study.** The diagram reports screening, randomisation and follow-up of ALS participants in the trial.

**Table 1:** Demographic and clinical characteristics of the participants to the FETR-ALS study at baseline (Intention-to-Treat Population).

Characteristic	SP arm (n = 14)	FMT arm (N = 28)
Sex, male—n (%)	8 (57.14)	14 (50.00)
Sex, female—n (%)	6 (42.86)	14 (50.00)
Race, white—n (%)	14 (100.0)	28 (100.0)
Age at baseline, years–median (IQR)	61.02 (57.97–65.96)	54.32 (49.76–62.46)
Bulbar onset–n (%)	5 (35.71)	5 (17.86)
Spinal onset—n (%)	9 (64.29)	23 (82.14)
Months since ALS symptom onset–median (IQR)	10 (8.25–13.50)	14 (9.75–16.25)
ALSFRS-R total score–mean (SD)	41.21 (4.49)	38.89 (5.59)
Bulbar score (item 1–3)–mean (SD)	10.64 (1.74)	10.96 (1.69)
Fine-motor score (item 4–6)–mean (SD)	9.86 (1.92)	7.96 (2.97)
Gross-motor score (item 7–9)–mean (SD)	8.71 (3.15)	8.18 (2.88)
Breathing score (item 10–12)–mean (SD)	12.00 (0.00)	11.79 (0.97)
Prebaseline ALSFRS-R slope^a^–mean (SD)	0.80 (0.95)	0.71 (0.47)
FVC–% of predicted normal value–mean (SD)	91.86 (17.39)	99.61 (17)
MRC score–proportion of the maximum possible score–mean (SD)	0.90 (0.12)	0.84 (0.12)
Body-mass index, kg/m^2^–median (IQR)	27.1 (24.75–28.75)	23.85 (21.58–27.28)
Weight loss since onset of the disease (%)–mean (SD)	4.36 (8.20)	2.06 (8.94)
ALSAQ40 total score–median (IQR)	23.75 (11.56–40.16)	33.13 (20.00–43.28)
Riluzole treatment–n (%)	14 (100.00)	28 (100.00)
Any cardiovascular disorder^b^–n (%)	8 (57.14)	8 (28.57)
Any endocrine/metabolic disorder^c^–n (%)	8 (57.14)	11 (39.29)
Any psychiatric disorder–n (%)	1 (7.14)	3 (10.71)
Any respiratory disorder–n (%)	0 (0.00)	3 (10.71)
Any musculoskeletal disorder–n (%)	2 (14.29)	5 (17.86)
Any other chronic disorder^d^–n (%)	3 (21.43)	6 (21.43)
Centre 1—n (%)	14 (100.00)	19 (67.86)
Centre 2—n (%)	0 (0.00)	4 (14.29)
Centre 3—n (%)	0 (0.00)	1 (3.57)
Centre 4—n (%)	0 (0.00)	4 (14.29)

Abbreviations: IQR = Interquartile Range; ALSFRS-R = Amyotrophic Lateral Sclerosis Functional Rating Scale Revised; SD = Standard deviation; FVC = Forced Vital Capacity; MRC = Medical Research Council scale; ALSAQ40 = Amyotrophic Lateral Sclerosis Assessment Questionnaire.

a Prebaseline ALSFRS-R slope has been calculated as monthly decline of ALSFRS-R score assuming a total score of 48 at onset.

b Including hypertension, ischaemic heart disease, atrial fibrillation, carotid atherosclerosis, and cardiac conduction disorders.

c Including diabetes mellitus, dyslipidaemia, hypercholesterolaemia, hypertriglyceridaemia, thyroid disorders, and polycystic ovary syndrome.

d Including benign prostatic hyperplasia, monoclonal gammopathy of undetermined significance, iron deficiency disorders, uterine fibromatosis, endometriosis, vitiligo, and allergy-related conditions.

**Table 2:** Primary and secondary efficacy outcomes at month 6.

Outcome	SP arm	FMT arm	Effect estimate (95% CI)	p value
Primary endpoint				
Participants with ≥20% increase in circulating Tregs, n/N (%)	2/10 (20.0%)	8/18 (44.4%)	OR 3.20 (0.59–25.36)	0.21
Participants with any increase in circulating Tregs, n/N (%)	3/10 (30.0%)	12/18 (66.7%)	OR 4.67 (0.94–28.47)	0.071
Change in circulating Tregs from baseline (percentage points), mean (SE)	−1.39 (0.90)	0.96 (0.66)	MD 2.36 (0.19–4.55)	0.042
Change in intestinal Tregs from baseline (percentage points), mean (SE)	−0.15 (1.30)	1.07 (0.84)	MD 1.22 (−1.77 to 4.27)	0.44
Secondary clinical outcomes				
ALSFRS-R monthly decline (points/month), mean (SE)	−1.07 (0.08)	−0.76 (0.06)	MD 0.31 (0.11–0.51)	0.002
FVC at month 6 (% of predicted normal value), mean (SD)	77.30 (19.77)	86.87 (19.61)	MD 9.57 (−5.62 to 24.75)	0.21
MRC at month 6 (proportion of the maximum possible score), mean (SD)	0.89 (0.08)	0.75 (0.19)	MD −0.13 (−0.26 to 0.00)	0.042
BMI at month 6 (kg/m^2^), mean (SD)	25.95 (2.61)	24.44 (3.93)	MD −1.20 (−3.55 to 1.15)	0.31
ALSAQ-40 total score at month 6, mean (SD)	30.56 (22.61)	44.13 (19.71)	MD 13.57 (−2.34 to 29.48)	0.092
PEG placement, n/N (%)	3/14 (21.4%)	2/28 (7.1%)	HR 0.26 (0.04–1.59)	0.15
NIV initiation, n/N (%)	3/14 (21.4%)	9/28 (32.1%)	HR 1.30 (0.35–4.80)	0.69
Tracheostomy-free survival events	3 deaths	1 tracheostomy	HR 0.15 (0.02–1.48)	0.10
Secondary biological outcomes				
Treg/Th1 ratio change from baseline to month 6, mean ratio (SE)	1.19 (1.20)	3.07 (1.92)	Ratio 2.58 (0.46–13.78)	0.28
Treg/Th17 ratio change from baseline to month 6, mean ratio (SE)	0.85 (0.51)	1.23 (0.50)	Ratio 1.45 (0.43–4.79)	0.55
Serum NfH at month 6 (pg/ml), mean (SD)	3755.39 (2637.86)	2431.50 (2127.41)	MD −1323.89 (−3109.51 to 461.73)	0.14
Serum NfL at month 6 (pg/ml), mean (SD)	145.35 (72.18)	134.86 (93.21)	MD −10.49 (−78.59 to 57.61)	0.75
Creatinine at month 6 (mg/dL), mean (SD)	0.69 (0.17)	0.61 (0.18)	MD −0.08 (−0.21 to 0.05)	0.23
Albumin at month 6 (g/dL), mean (SD)	4.36 (0.14)	4.33 (0.29)	MD −0.03 (−0.22 to 0.17)	0.76
Vitamin D at month 6 (ng/mL), mean (SD)	26.06 (8.08)	27.16 (13.51)	MD 1.11 (−10.03 to 12.24)	0.84

Abbreviations: ALSAQ-40 = Amyotrophic Lateral Sclerosis Assessment Questionnaire; ALSFRS-R = Amyotrophic Lateral Sclerosis Functional Rating Scale–Revised; BMI = body-mass index; CI = confidence interval; FMT = faecal microbiota transplantation; FVC = forced vital capacity; HR = hazard ratio; MD = mean difference; MRC = Medical Research Council scale; NfH = neurofilament heavy chain; NfL = neurofilament light chain; NIV = non-invasive ventilation; OR = odds ratio; PEG = percutaneous endoscopic gastrostomy; SE = standard error; SP = sham procedure; Tregs = regulatory T cells.

The table summarises the main efficacy outcomes comparing FMT versus SP at the primary study timepoint (month 6). For binary outcomes, results are reported as number and percentage of participants with the corresponding event, together with odds ratios (ORs) or hazard ratios (HRs) and 95% confidence intervals (CIs). The reported p-values were obtained from Wald test carried out on the coefficients of logistic regression models or Cox proportional hazard regression models used to investigate these outcomes. For continuous outcomes, results are reported, as appropriate, as changes from baseline, average monthly variation, or values at month 6, together with corresponding mean differences (MDs) or ratios and 95% CIs. Linear mixed models were used for longitudinal continuous outcomes, with p-values obtained from t test on the regression parameters, using the Satterthwaite method to estimate degrees of freedom. Positive MD values indicate a treatment effect favouring FMT, corresponding to slower functional decline or improved biological response compared with SP. Survival outcomes were analysed as time-to-event variables from randomisation to death, tracheostomy, PEG placement, or initiation of NIV, as appropriate. Analyses were performed in the intention-to-treat population including all randomised participants with available longitudinal assessments.

Intention-To-Treat Population.

### Study population and participant flow

A total of 42 participants with ALS were enrolled between 17 July 2020 and 15 Feb 2023. The last participant last visit was conducted on 15 Feb 2024. Twenty-eight participants were allocated to FMT and 14 to SP. Nine participants (21.4%) dropped out during the treatment period (n = 5, 17.8% in FMT, n = 4, 28.6% in SP arm), and an additional eight (19.05%) discontinued during the 6-to-12-month follow-up period (n = 6, 21.4% in FMT, n = 2, 14.2% in SP arm). However, all participants with available biological samples at 6 months had undergone the second procedure. Therefore, for biomarker outcomes, the ITT and PP populations overlapped, whereas clinical outcomes differed slightly between ITT and PP due to two participants who discontinued before the second intervention but were still clinically followed.

### Primary endpoint: effects of FMT on circulating and intestinal regulatory T cells

The primary endpoint, i.e. the proportion of participants showing a positive biological response at 6 months, defined as a ≥20% increase in peripheral Tregs, was not met. In the ITT population, a ≥20% increase in circulating Tregs at month 6 was observed in 8/18 (44.4%) FMT-treated participants compared with 2/10 (20.0%) participants in the SP group (OR 3.20; 95% CI 0.59–25.36; p = 0.207 Wald test) corresponding to a moderate effect size (Cohen's h = 0.53, 95% CI –0.24 to 1.31). For intestinal Tregs, a ≥20% increase was observed in 10/19 (52.6%) FMT-treated participants versus 1/8 (12.5%) SP-treated participants (OR 7.78; 95% CI 0.80–76.09; p = 0.078 Wald test) corresponding to a large effect size (Cohen's h = 0.90, 95% CI 0.07–1.73) (Table 2, Supplementary Table S3). Sensitivity analyses performed under different missing-data assumptions yielded broadly consistent estimates across all imputation strategies, with the expected attenuation of the treatment effect only under the deliberately extreme conservative scenario imputing missing FMT participants as non-responders and missing SP participants as responders (Fig. 2).

**Fig. 2:**
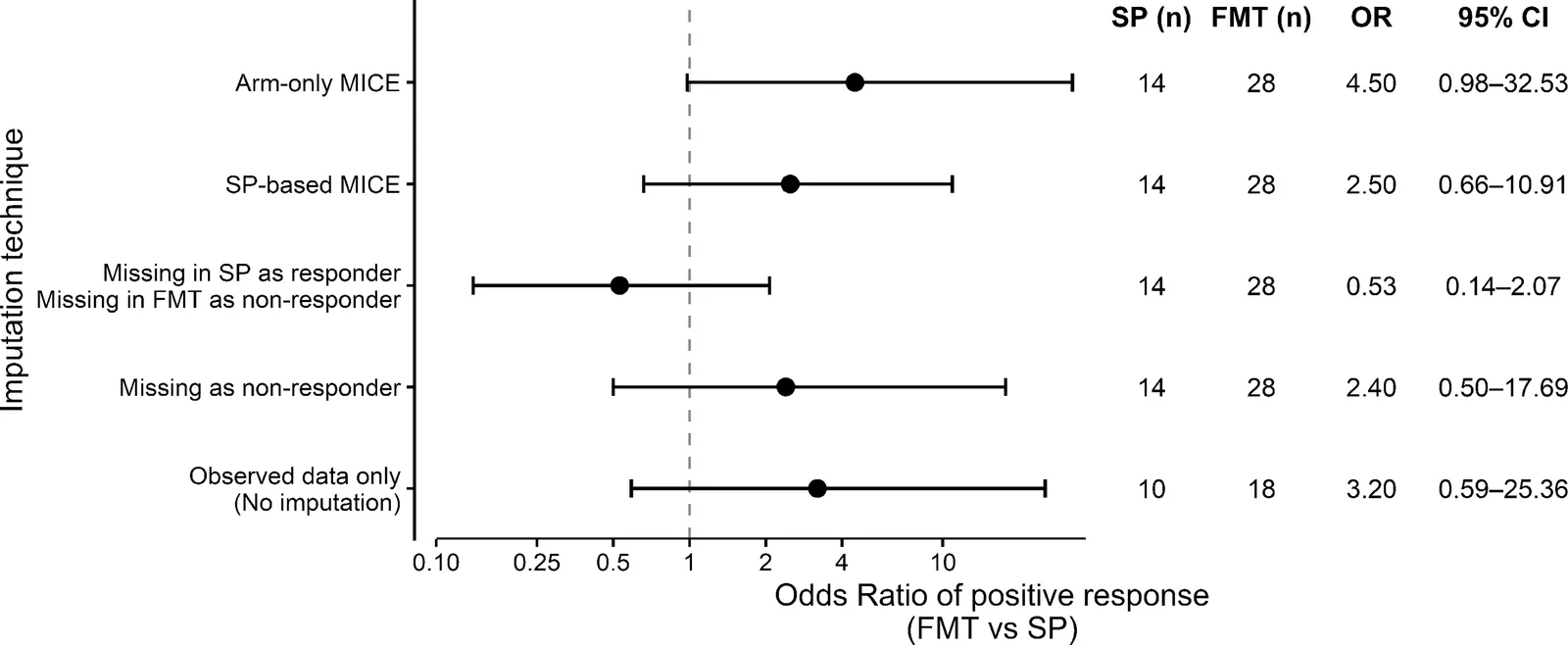
**Sensitivity analyses of the primary endpoint under different missing-data assumptions.** Forest plot showing the odds ratio (OR) and 95% CI for achieving a positive biological response defined as a ≥20% increase in circulating Tregs at month 6, in the FMT group (n = 28) compared with the SP group (n = 14) under different approaches for handling missing data. The x-axis is displayed on a logarithmic scale, and numerical ORs and 95% CIs are reported for each analysis. Analyses included: observed data only (no imputation performed), imputation of missing values as non-responders, a conservative scenario imputing missing values in the SP group as responders and in the FMT group as non-responders, multiple imputation by chained equations (MICE) based on SP-group data only, and MICE carried out considering only arm information. The vertical dashed line indicates the null effect (OR = 1). Results were generally consistent across imputation strategies. Univariable logistic regression models were used to estimate the reported OR and their variability.

Overall, 12/18 (66.7%) participants in the FMT group showed any increase in circulating Tregs at month 6, compared with 3/10 (30.0%) in the SP group (OR 4.67; 95% CI 0.94–28.47; p = 0.071 Wald test; Cohen's h = 0.75, 95% CI −0.02 to 1.52, indicating a moderate to large effect size). Similarly, 13/19 (68.4%) FMT-treated participants versus 2/8 (25.0%) SP-treated participants showed an increase in gut Tregs (OR 6.50; 95% CI 1.00–42.17; p = 0.050 Wald test; Cohen's h = 0.90, 95% CI 0.07–1.73, indicating a large effect size).

From baseline to month 6, circulating Tregs increased by a mean 0.96 (SE 0.66) in the FMT group and decreased by 1.39 (SE 0.90) in the SP group, resulting in a mean between-group difference (MD) of 2.36 (95% CI 0.19–4.55; p = 0.042 t test from a linear mixed-effects model with Satterthwaite approximation for degrees of freedom). The mean between-group difference observed for gut Tregs was 1.22 (95% CI −1.77 to 4.27; p = 0.437 t test from a linear mixed-effects model with Satterthwaite approximation for degrees of freedom) (Table 2; Supplementary Tables S4–S6, Supplementary Figure S1).

### Clinical outcomes and functional progression

Changes in ALSFRS-R over time were analysed in the ITT population, including all randomised participants with available longitudinal assessments (Supplementary Table S7). The MD between the average of the difference of the ALSFRS-R score in the FMT-treated and SP arm between month 6 and baseline was −3.70 (SE 3.08, 95% CI −9.98 to 2.58, p = 0.24 Wald test) (Supplementary Table S8).

In linear mixed-effects models accounting for repeated measures and individual trajectories based on ALSFRS-R scores collected at months 1, 3, 6, 7, 9, and 12, the ALSFRS-R total score declined on average by −1.07 points/month in the SP arm and by −0.76 points/month in the FMT arm, corresponding to a between-group MD in monthly decline of 0.31 points/month favouring FMT (95% CI 0.11–0.51; p = 0.002 t test for the arm-by-time interaction, with Satterthwaite approximation for degrees of freedom) (Supplementary Tables S9–S11, Supplementary Figure S2).

Three deaths and one tracheostomy occurred respectively in the SP and FMT arm within the study period, all attributed to respiratory failure by the treating neurologist at the participating centre. Between-group differences in FVC, BMI, ALSAQ40, NIV, and PEG placement and tracheostomy free survival are reported in Table 2, Supplementary Tables S6, S12 and S13, and Supplementary Figures S2–S4.

### Safety and tolerability

Table 3 reports treatment-emergent AE at month 6, by treatment arm (FMT versus SP) and categorised according to MedDRA classification, in the ITT population. Extended safety analyses across additional follow-up and the complete adverse-event dataset are provided in Supplementary Tables S14 and S15 and Supplementary Figures S5 and S6. At month 6 of FETR-ALS trial, 9/28 (32.1%) FMT and 4/14 (28.6%) SP patients experienced at least one AE (Risk difference = 0.04, 95% CI −0.26 to 0.33). During the entire trial duration, a total of 32 AEs were reported in the FMT group and 14 in the SP group. Severe AEs represented 4/32 (12.5%) events in FMT and 4/14 (28.6%) in SP. Three AEs led to treatment discontinuation (2 in FMT, 1 in SP arm). Individuals experiencing serious AEs were 4/28 (10.7%) in FMT and 4/14 (28.6%) in SP arms.

**Table 3:** Treatment-emergent adverse events during the FETR-ALS trial at month 6, by treatment arm (FMT versus SP) and categorised according to MedDRA classification.

Treatment-emergent adverse events, MedDRA preferred term	SP arm		FMT arm		Risk difference	95% CI	
	(n = 14)		(n = 28)				
	N	%	N	%			
Drop-outs	4	28.57	5	17.86	−0.11	−0.38	0.17
Deaths/Tracheostomy	2	14.29	1	3.57	−0.11	−0.30	0.09
Withdrawals due to harms	0	0.00	0	0.00	0.00	−0.22	0.12
Nr participants at risk of harms at month 6	10	71.43	23	82.14	0.11	−0.17	0.38
Nr patients with at least one AE	4	28.57	9	32.14	0.04	−0.26	0.33
AEs	7		18				
SAEs	2		2				
Adverse events of special interest							
Gastrointestinal disorders	**1**	**7.14**	**6**	**21.43**	**0.14**	**−0.06**	**0.35**
Constipation	0	0.00	1	3.57	0.04	−0.03	0.10
Faecaloma	0	0.00	1	3.57	0.04	−0.03	0.10
Diarrhoea–Nausea	1	7.14	2	7.14	0.00	−0.17	0.17
Hypogastrium distension–Hypogastrium pain	0	0.00	1	3.57	0.04	−0.03	0.10
Chronic gastritis	0	0.00	1	3.57	0.04	−0.03	0.10
Enteritis	0	0.00	0	0.00	0.00	−0.22	0.12
Infections and infestations	**0**	**0.00**	**2**	**7.14**	**0.07**	**−0.02**	**0.17**
Covid 19	0	0.00	1	3.57	0.04	−0.03	0.10
Peristomal infection	0	0.00	0	0.00	0.00	−0.22	0.12
Gastroenteritis	0	0.00	1	3.57	0.04	−0.03	0.10
Others adverse events							
Metabolism and nutrition disorders	**0**	**0.00**	**1**	**3.57**	**0.04**	**−0.03**	**0.10**
Hypoglycemia	0	0.00	1	3.57	0.04	−0.03	0.10
Dysphagia	0	0.00	0	0.00	0.00	−0.22	0.12
Respiratory, thoracic, and mediastinal disorders	**5**	**35.71**	**5**	**17.86**	**−0.18**	**−0.47**	**0.11**
Cough	0	0.00	1	3.57	0.04	−0.03	0.10
Dyspnoea	1	7.14	0	0.00	−0.07	−0.21	0.06
Pneumonia–Pulmonary embolism	1	7.14	3	10.71	0.04	−0.14	0.21
Respiratory failure	3	21.43	1	3.57	−0.18	−0.40	0.05
General disorders and administration site conditions	**0**	**0.00**	**1**	**3.57**	**0.04**	**−0.03**	**0.10**
Pyrexia	0	0.00	1	3.57	0.04	−0.03	0.10
Injury, poisoning, and procedural complications	**2**	**14.29**	**1**	**3.57**	**−0.11**	**−0.30**	**0.09**
Fall	2	14.29	0	0.00	−0.14	−0.33	0.04
Foot fracture	0	0.00	0	0.00	0.00	−0.22	0.12
Leg fracture	0	0.00	0	0.00	0.00	−0.22	0.12
Rib contusion	0	0.00	1	3.57	0.04	−0.03	0.10
Accidental removal of percutaneous endoscopic gastrostomy tube	0	0.00	0	0.00	0.00	−0.22	0.12
Musculoskeletal and connective tissue disorders	**0**	**0.00**	**1**	**3.57**	**0.04**	**−0.03**	**0.10**
Arthralgia	0	0.00	1	3.57	0.04	−0.03	0.10
Psychiatric disorders	**0**	**0.00**	**1**	**3.57**	**0.04**	**−0.03**	**0.10**
Insomnia–Anxiety–Agitation	0	0.00	1	3.57	0.04	−0.03	0.10
Renal and urinary disorders	**1**	**7.14**	**1**	**3.57**	**−0.04**	**−0.19**	**0.12**
Bladder distension and urinary catheterisation	1	7.14	0	0.00	−0.07	−0.21	0.06
Dysuria	0	0.00	1	3.57	0.04	−0.03	0.10
Skin and subcutaneous tissue disorders	**0**	**0.00**	**1**	**3.57**	**0.04**	**−0.03**	**0.10**
Allergic reaction erythema	0	0.00	1	3.57	0.04	−0.03	0.10

Abbreviations: MedDRA = Medical Dictionary for Regulatory Activities.

The AEs and SAEs rows report total numbers of events rather than numbers of participants; therefore, percentages and risk differences are not applicable.

Bold type indicates summary rows and MedDRA system organ class categories from individual adverse event terms.

Intention-to treat population.

AEs more frequently reported in the FMT group included gastrointestinal, infectious, skin, musculoskeletal, nervous system and psychiatric disorders. Most severe AEs were related to disease progression, such as respiratory failure and/or pneumonia.

Time-to-first AE and recurrent-event analyses accounting for variable follow-up yielded similar findings, with no evidence of an increased safety burden associated with FMT (Supplementary Figures S5 and S6).

### Analyses on neurofilament and other peripheral biomarkers

No between-group differences over time were observed in serum or CSF neurofilaments, their monthly rates of change, or other exploratory biomarkers, including creatinine, albumin, and C-reactive protein (Supplementary Tables S16 and S17, Supplementary Figure S7).

### Exploratory analyses of immune-cell subpopulations and cytokine profiles

Cytofluorimetric analyses showed largely stable peripheral and intestinal T-cell subset distributions over time, with only modest between-group differences that did not translate into consistent immune shifts (Supplementary Tables S18–S23, Supplementary Figures S8 and S9). Multiplex cytokine profiling across serum, tissue, saliva, and faeces revealed no differences in changes from baseline to months 6 or 12, nor in average monthly variation (Supplementary Tables S24–S31). CSF cytokines were measured in seven participants at baseline and 4 at month 6, with several values below the lower limit of quantification. No consistent between-group differences emerged, but the small sample size precluded meaningful interpretation.

### Effects of FMT on circulating free fatty acids

Free fatty acid analyses indicated trends toward increased hexadecanoic and tetradecanoic acids at months 6 and 12 and decreased butyric acid at month 12 in the FMT arm compared with SP (Supplementary Tables S32 and S33).

### Gut microbiota composition and diversity analyses

Microbial community profiles were assessed on faecal samples collected at baseline and at 6 months in both FMT and SP arms. Firmicutes and Bacteroidetes remained the dominant phyla throughout the study period in both FMT and SP groups (Supplementary Table S34). Alpha diversity, beta diversity and community dispersion analyses did not reveal significant longitudinal changes in either treatment arm, indicating the absence of major ecosystem-wide modifications in gut microbiota composition (Supplementary Figures S10–S12).

Despite the overall stability of microbial community diversity and composition, taxonomic analyses identified significant longitudinal variations in specific bacterial taxa, mainly among FMT-treated participants. At the phylum level, Actinobacteria increased, whereas Bacteroidetes and Proteobacteria decreased over time in the FMT arm (Supplementary Figure S13). At the genus level, FMT was associated with increases in *Bifidobacterium*, *Collinsella*, unclassified Firmicutes, and unclassified Ruminococcaceae, together with decreases in *Barnesiella*, *Blautia*, *Gemmiger*, *Lachnospiraceae incertae sedis*, and *Parabacteroides* (Supplementary Figure S14). On the contrary, fewer taxa exhibited significant longitudinal changes in the SP arm (Supplementary Table S35).

Differential abundance analysis using DESeq2 identified a greater number of significantly altered taxa in the FMT group compared with the SP group (Supplementary Figure S15). However, these changes were heterogeneous across participants and were not accompanied by convergence toward a shared microbial configuration. Consistently, donor-recipient analyses did not reveal movement of recipient microbiota toward donor centroids, clustering according to donor identity, or evidence of stable donor-derived microbial engraftment (Supplementary Figure S16).

Overall, these findings indicate that FMT induced selective and individualised microbial responses, characterised by modulation of specific bacterial taxa rather than by a reproducible restructuring of the gut microbial ecosystem.

Finally, exploratory analyses did not identify consistent associations between the magnitude of GM compositional changes, and neurofilament trajectories or clinical progression, although the limited sample size substantially decreased the power of these analyses.

## Discussion

FMT has gained increasing attention as a potential immunomodulatory therapy in neurodegenerative diseases,^10^^,^^30^^,^^31^ yet its role in ALS remains largely unexplored.^10^ Recent systematic reviews support an association between GM alterations and ALS, while emphasising substantial heterogeneity across studies and the lack of evidence establishing a causal relationship.^32^ There is a lack of other studies that have comprehensively investigated the microbiota–immune axis or the immune mechanisms by which FMT may exert biological effects in this population.

In this exploratory randomised clinical trial we investigated whether FMT could modulate peripheral immunity in ALS, focussing on Tregs, a biomarker consistently associated with slower disease progression and longer survival.^33^ Although the prespecified binary primary endpoint, defined as a ≥20% increase in circulating Tregs at 6 months, was not met, FMT was associated with a between-group difference in mean Treg change, along with a trend towards a higher proportion of participants exhibiting any increase in Tregs. These findings suggest a biologically meaningful immunological signal, while underscoring the challenges of dichotomising immune responses using conservative thresholds in exploratory phase II studies. Notably, the higher-than-expected proportion of spontaneous Treg increases observed in SP participants suggests substantial intrinsic variability of circulating Tregs in ALS, which may have diminished the effective treatment contrast and complicated interpretation of immunological endpoints in small exploratory studies.

Prior preclinical and clinical evidence indicates that FMT can induce systemic and mucosal immune shifts,^34^^,^^35^ which may persist even after a single infusion.^36^ Our results extend these observations to ALS, supporting the concept that microbiota-based interventions may influence immune homoeostasis in ALS. This concept is also supported by preclinical evidence showing that manipulation of the GM can modulate neurodegenerative microglial phenotypes and disease progression in ALS models.^37^^,^^38^ However, in our study, exploratory analyses of additional immune-cell populations and cytokine profiles did not identify a consistent treatment-related immunological signature beyond the predefined Treg endpoints. These findings suggest that the immunomodulatory effects of FMT in ALS may be relatively selective rather than reflect broad immune remodelling. However, these results should be interpreted with caution given the exploratory nature of the analyses and the limited sample size.

Finally, the modest and heterogeneous nature of the observed microbiota and immune changes also suggests that the treatment schedule adopted in this study, consisting of only two administrations six months apart, may have been inappropriate to induce sustained microbiota remodelling and long-term immunomodulation in a chronic neurodegenerative disease such as ALS. Of note, the magnitude and variability of the observed immune changes suggest that prolonged or maintenance strategies, such as repeated dosing, shorter dosing intervals, or oral faecal capsules, may be required to achieve more robust and sustained immunomodulation.^39^

Microbiota profiling revealed heterogeneous community changes following FMT. Unlike trials conducted in inflammatory bowel and metabolic disease, where pronounced microbial restructuring has been reported,^40^^,^^41^ we observed no major shifts in alpha or beta diversity. This finding may reflect the lower taxonomic resolution of 16S sequencing compared with shotgun metagenomics.^42^ Nonetheless, phylum-level changes emerged, with increased Actinobacteria and decreased Bacteroidetes and Proteobacteria in the FMT group, taxa previously linked to immune regulation and anti-inflammatory signalling.^43^ Notably, post-FMT microbial shifts were heterogeneous and not donor-specific. These findings suggest that the biological effects of FMT in ALS may be highly context- and host-dependent, with substantial inter-individual variability potentially masking microbiota–clinical correlations in relatively small exploratory cohorts. Indeed, experimental evidence indicates that host genetic background can substantially influence GM composition, microbial metabolism, and immune responses in ALS models.^44^ In our study stool material in this study was obtained from multiple rigorously screened donors, due to difficulties to find a single donor for each patient through the entire duration of the study. Although, the use of multiple donors may be a potential confounding variable, the identification of a healthy microbiota remains hard to pinpoint.^45^ Additionally, the inter-individual variability observed in our cohort further support the possibility of patient-specific responses to FMT, potentially shaped by host- or donor-related factors.^46^ Our results suggest that studies integrating donor-recipient matching, taxonomic and functional metagenomic evaluation, host and environmental factors investigation will be needed to define engraftment dynamics and to optimise FMT efficacy.^47^

Metabolically, FMT-treated patients showed a trend towards higher circulating tetradecanoic (myristic) and hexadecanoic (palmitic) acid concentrations at 6 and 12 months, suggesting systemic lipid remodelling with potential immunometabolic implications.^9^^,^^48^ In contrast, serum butyrate tended to decrease at 12 months, a finding that may reflect enhanced local utilisation within the gut rather than reduced microbial production. This interpretation aligns with emerging evidence that butyrate is rapidly metabolised at sites of immune and epithelial activity rather than accumulating systemically.^49^^,^^50^

FMT was not associated with major safety concerns and well tolerated, confirming its feasibility in the ALS population.^10^ Adverse events, primarily gastrointestinal and infectious, occurred more frequently in the FMT arm but were generally mild to moderate, and comparable to those reported in other FMT trials.^50^ Time-to-event and recurrent-event analyses further suggested that this imbalance was largely confined to the early post-intervention period. Kaplan–Meier and mean cumulative function plots showed a higher initial frequency of adverse events in the FMT group, with progressive convergence of the curves over time and no apparent difference by approximately six months. Although these descriptive analyses assign equal weight to all AEs regardless of severity, they suggest that any early excess of AEs associated with FMT was transient rather than sustained.

Although this exploratory proof-of-concept trial was not powered to assess clinical efficacy, we found a slower decline in ALSFRS-R in FMT-treated patients, corresponding to an average difference of 0.31 points per month. Although this effect was modest and mainly driven by fine motor upper-limb items, its statistical significance in such a small cohort can suggest that the immunological changes induced by FMT may have translated into a measurable functional signal. Moreover, the composite tracheostomy-free survival outcome was slightly in favour of FMT, with fewer events in the FMT group than in the SP group. However, the nature of the events differed between groups: the three events in the SP group were deaths, whereas the single event in the FMT group was a non-fatal tracheostomy, with no deaths occurring in the FMT group.These findings should be interpreted cautiously, as clinical efficacy was a secondary exploratory outcome, but they provide additional support for further evaluation of microbiota-targeted interventions in adequately powered clinical trials.

A previous study, prematurely discontinued for financial reasons, reported no clinical benefit of FMT in ALS.^10^ However, that trial differed in several key aspects: it employed a seven-day colonoscopy-based regimen, included no immunological endpoints, and required antibiotic pre-conditioning, which may reduce gut microbial diversity and hinder engraftment.^51^ By contrast, our protocol used repeated nasojejunal infusions without antibiotics and incorporated comprehensive immune and biomarker analyses. The nasojejunal route may offer advantages in terms of tolerability and access to small-intestinal immune niches, although further investigation is warranted.^52^

This study has some strengths. Its multicentre, randomised, sham-controlled and masked design, with centralised FMT procedures and standardised clinical assessments across participating centres, reduced potential sources of bias. The longitudinal collection of clinical and biological measures enabled the integration of functional outcomes with peripheral and intestinal immunological profiling, biomarker analyses, and GM characterisation. In addition, the use of fresh samples for immunopheotyping and the assessment of both circulating and intestinal T-cell populations allowed a detailed evaluation of the potential immunological effects of FMT in ALS.

This study has also several limitations. The modest sample size, designed primarily to detect a biological signal and assess feasibility, resulted in limited precision for secondary and exploratory outcomes and reduced statistical power for subgroup analyses. The conservative, prespecified binary primary endpoint may have reduced sensitivity to more subtle immune changes. Attrition was substantial, and biological analyses were restricted to participants with available samples. This was partly related to the vulnerability of the ALS population, disease progression, and the logistical complexity of repeated endoscopic procedures and biological sampling. Furthermore, the study was conducted during the COVID-19 pandemic, which contributed to logistical challenges, delayed procedures, and incomplete biological sampling in a subset of participants. Although missing data may have influenced the precision of the estimates, sensitivity analyses using different imputation strategies yielded broadly consistent results. Nevertheless, residual bias related to non-random missingness cannot be completely excluded. Additionally, although the study was multicentre, most participants were recruited at the coordinating site, which may limit generalisability. Microbiota profiling was based on 16S rRNA sequencing, limiting functional inference and potentially underestimating donor engraftment or metabolically relevant changes. The use of multiple donors, while improving feasibility and reducing dependence on individual donor characteristics, may have increased variability in microbiota responses among recipients. More broadly, the marked inter-individual heterogeneity of both the gut microbiome and ALS itself may have decreased our ability to detect consistent microbiota–clinical associations. Finally, as this was an exploratory proof-of-concept study, effect sizes should be interpreted cautiously and not directly extrapolated for planning subsequent efficacy trials, as early-phase studies may overestimate treatment effects due to limited sample size and exploratory design. Future efficacy trials should be adequately powered to confirm the exploratory signals observed here, with sample-size calculations informed by the effect estimates and their uncertainty observed in this study, together with other available evidence, rather than relying directly on the point estimates from this pilot trial.

Overall, this study indicates that FMT is associated with modest immunological effects and no major safety concerns. Future trials should refine donor selection, optimise treatment frequency and duration, and integrate multi-omics approaches to delineate the therapeutic relevance of GM modulation in ALS.

## Contributors

J.M., L.M., A.A., and R.D.A. conceived and designed the study. E.Z., E.N., F.D.M., R.C.C., G.Q., A.G., S.Bi., I.M., G.G., C.S., G.N., M.L., S.Ba., G.Ba., R.V., M.C., A.C., G.Bi., P.B., and G.C. contributed to acquisition of data. R.C.C., R.D.A., E.Z., J.M., E.N., A.A., F.D.M., L.M., and S.P. contributed to data analysis and interpretation. R.C.C., R.D.A., and F.D.M. performed the statistical analyses. J.M., L.M., and A.A., obtained funding. E.Z., E.N., R.C.C., and J.M. drafted the manuscript. E.Z., J.M., R.C.C., and R.D.A. accessed and verified the underlying data. All authors critically revised the manuscript for important intellectual content and approved the final version. All authors had full access to all the data in the study and take responsibility to submit for publication.

FETR-ALS Study Group contributed to clinical assessments, biological sample collection and processing, and/or data collection at the participating centres, as appropriate to their respective roles.

## Data sharing statement

The data that support the findings of this study are available from the corresponding author (jessica.mandrioli@unimore.it) to external researchers who provide methodologically sound scientific proposals and whose proposed use of the data has been approved by an independent review committee identified for this purpose. All requests will be reviewed by corresponding author and Steering Committee of the Study and a response to requests will be given in 2 months. A materials transfer and/or data access agreement with the study promoter will be required for accessing shared data. However, individual participant data will not be available because informed consent did not explicitly cover sharing of participant-level data. The study protocol, including the statistical analysis plan, has been uploaded in the Supplementary files.

## Declaration of interests

J.M. reports the following: consulting for Biogen, Amylyx, Zambon, Italfarmaco, Maat Pharma; research support from Pfizer, Biogen, Ionis, Roche, Apellis, Ferrer, ABScience, Amylix, PTC Therapeutics, Novartis.

S.P. reports the following: research grants from Amylyx Therapeutics, Revalesio, Eledon, Alector, UCB Pharma, Biohaven, Clene Nanomedicine, Prilenia Therapeutics, Seelos, Calico, Denali, Neurizon, NIH, DoD, the Muscular Dystrophy Association; consulting fees from Amylyx, Arrowhead, Biogen, BMS, Clene, Cytokinetics, Eikonizo, J&J, Merck, PharmAust, Prilenia, and Sola; a Scientific Advisory Board role with the NEALS Consortium; Editorial Board roles with the American Journal of PM&R and Muscle & Nerve. She has been a paid educational speaker for Medscape, PeerView, and i3Health.

E.Z. and I.M report having received financial support for the participation to scientific meetings by Zambon Italia.

A.C. reports having received consulting fees from Zambon Italia.

E.N., F.D.M., R.C.C., G.Q., A.G., S.B., G.G., C.S., G.N., M.L., S.B., G.B., R.V., M.C., G.B., P.B., G.C. R.D.A., A.A., L.M., declare no competing interests.

The FETR-ALS Study Group received no separate funding.

## Appendix A Supplementary data

Supplementary Material

FETR-ALS Study Protocol
