# Supplementary material for: Immune effects of faecal microbiota transplantation in amyotrophic lateral sclerosis: a pilot multicentre, randomised, masked, sham-controlled, proof-of concept trial
Source: eBioMedicine. 2026 Oct 8;133:106509. doi: 10.1016/j.ebiom.2026.106509 (PMC13676721; doi:10.1016/j.ebiom.2026.106509)
Supplement: Supplementary file 1 [file mmc2.pdf]

UNIMORE
UNIVERSITÀ DEGLI STUDI DI MODENA E REGGIO EMILIA

Dipartimento Integrato di Neuroscienze
Unità Operativa di Neurologia
Ospedale Civile Sant'Agostino Estense

SERVIZIO SANITARIO REGIONALE
EMILIA-ROMAGNA
Azienda Ospedaliero - Universitaria di Modena

Via Giardini, 1355
Baggiovara
41126 Modena
tel. : 059396111
fax.: 0593962409

Clinical Study Protocol

INTERPLAY BETWEEN GUT MICROBIOTA AND ADAPTIVE IMMUNITY IN AMYOTROPHIC LATERAL SCLEROSIS: A CLINICAL TRIAL

**Protocol Acronym:**	**FETR-ALS**
**Title:**	**Interplay between gut microbiota and adaptive immunity in amyotrophic lateral sclerosis: a clinical trial**
**Study design:**	**Multicenter, randomised, double-blind, controlled, interventional study to compare efficacy, biological effects, safety of fecal transplantation in patients with Amyotrophic Lateral Sclerosis (ALS)**
**Diagnosis:**	**Patients with definite or probable ALS**
**Intervention:**	**Fecal transplantation**
**Comparator product:**	**None**
**Duration of the study:**	**36 months**
**Coordinating investigator:**	**Dr Jessica Mandrioli, Ospedale Civile S. Agostino Estense, Via P. Giardini 1355, 41126 Modena, Italy**
**Sponsor:**	**Azienda Ospedaliero Universitaria di Modena**
**CRO**	**High Research s.r.l.**

Study Synopsis

**Study Title**	**INTERPLAY BETWEEN GUT MICROBIOTA AND ADAPTIVE IMMUNITY IN AMYOTROPHIC LATERAL SCLEROSIS: A CLINICAL TRIAL**
**Protocol acronym**	FETR-ALS
**Principal Investigator**	JESSICA MANDRIOLI
**Principal Institution**	DEPARTMENT OF NEUROSCIENCE, OSPEDALE CIVILE S. AGOSTINO-ESTENSE DI MODENA, AZIENDA OSPEDALIERO UNIVERSITARIA – MODENA
**Investigators and Participating Centres**	1) PROF.SSA TERESA SPANU, PROF. LUCA MASUCCI, DEPARTMENT OF MICROBIOLOGY – POLICLINICO UNIVERSITARIO "A. GEMELLI", ROME<br>2) DR.SSA ELENA NICCOLAI, PROF. AMEDEO AMEDEI, DEPARTMENT OF EXPERIMENTAL AND CLINICAL MEDICINE, UNIVERSITY OF FLORENCE, FLORENCE<br>3) PROF. ROBERTO D'AMICO, UNIVERSITY OF MODENA AND REGGIO EMILIA, MODENA<br>4) DR MARIO SABATELLI, DR.SSA AMELIA CONTE NEUROMUSCULAR OMNICENTRE CENTRE, 1NEUROMUSCULAR OMNICENTRE (NEMO) FONDAZIONE SERENA ONLUS-FONDAZIONE POLICLINICO A. GEMELLI, ROME; INSTITUTE OF NEUROLOGY, CATHOLIC UNIVERSITY OF THE SACRED HEART, FONDAZIONE POLICLINICO A. GEMELLI, ROME<br>5) PROF. VINCENZO DI LAZZARO, INSTITUTE OF NEUROLOGY, POLICLINICO CAMPUS BIO-MEDICO, ROME<br>6) DR.SSA SABRINA MATA, PROF. SANDRO SORBI, DIPARTIMENTO NEUROMUSCOLOSCHELETRICO E DEGLI ORGANI DI SENSO, AZIENDA OSPEDALIERO-UNIVERSITARIA CAREGGI, FLORENCE<br>7) DR SSA TERESA ANNA CANTISANI, DR.SSA PAOLA BRUNORI, UO NEUROFISIOPATOLOGIA, AZIENDA OSPEDALIERA DÌ PERUGIA<br>8) DR.SSA MARGHERITA CAPASSO, PROF. MARCO ONOFRJ, CLINICA NEUROLOGICA, OSPEDALE CLINICIZZATO "SS ANNUNZIATA", CHIETI<br>9) PROF. GIOVANNI CAMMAROTA, DIPARTIMENTO DI MEDICINA INTERNA E SCIENZE SPECIALISTICHE, POLICLINICO UNIVERSITARIO "A. GEMELLI", ROMA<br>10) PROF. FABIO GIANNINI, DIPARTIMENTO DI SCIENZE MEDICHE, CHIRURGICHE E NEUROLOGICHE, UNIVERSITÀ DI SIENA, OSPEDALE "LE SCOTTE" , SIENA
**Study Aims and Objectives**	**Objectives, primary aim:** to assess whether FMT increases Tregs number in ALS patients treated with FMT compared to the control arm and measured at baseline and at month 6; **secondary aims:** to assess biological activity of FMT on phenotype of peripheral blood and tissue cells, in CSF biomarkers (neurophylaments) cytokines, and on fecal and saliva microbiota; to assess FMT safety and tolerability in a cohort of ALS patients; to assess clinical activity (ALSFRS-R score, survival, FVC) and effect on quality of life (ALSAQ40) of FMT
**Study Rationale**	ALS is characterized by motor neurons (MNs) degeneration leading to death within 3-5 years from disease onset. The disease etiology and pathogenesis is largely unknown, and this represent one of the major causes of the failure of research for an effective treatment. Many recent clinical trials in ALS target microglia because it has been demonstrated that microglia can influence disease progression. In fact, in ALS there is simultaneous activation of distinct lymphocyte populations, Th1 and Th17, and a decrease of T regulatory cells (Tregs), the last ones having a protective role in both mice and patients with ALS: a greater number of Tregs is associated with slow disease progression . Tregs have been shown to directly differentiate macrophages from M1 to M2 state and M2 microglia has been associated with the stable disease phase, whereas Th1L and M1 microglia predominate during the rapidly progressing phase, suggesting a shift from protection to toxicity.<br>Intestinal microbiota includes a complex ecosystem with an exceptionally high bacterial density and diversity. Gut microbiota (GM) interacts directly with the enteric immune system, contributing to the maintenance of immune tolerance and shaping the immune responses during inflammation. Besides innate immune cells, enteric bacteria can directly affect adaptive immune responses. The GM influences the development and differentiation of both CD4+ and CD8+ T cells. Indeed, germ-free (GF) mice display a marked decrease in the number of both CD4+ and CD8+ T cells, and

Via Giardini, 1355
Baggiovara
41126 Modena
tel. : 059396111
fax.: 0593962409

	treatment with SCFA mixtures is associated with an increase in the density of T cells. Moreover, Erny et al. observed that GF mice displayed an altered density, morphology and maturity of microglia, and that treatment with a SCFA mixture restored the density and morphology of microglia, thus suggesting that GM can influence both the development and functions of CNS immune cells. Moreover GM contribute to preserve the BBB integrity. In this context, microbiota gut-brain axis is currently regarded as the key player in the regulation of mutual signaling between gut microflora and CNS employing bidirectional communication via neuronal, hormonal, immunologic and toxic signalling.<br>Further proposed mechanisms of interaction between gut and brain include direct communication through the vagus nerve, changes in tryptophan and norepinephrine metabolism, production and absorption of neuroactive metabolites, immune activation through molecular mimicry, and the direct production of neurotoxins as known for Clostridia.<br>These assumptions have led to study a possible microbiota role in neurological diseases such as multiple sclerosis, myasthenia gravis and Parkinson's disease. On the basis of the pathogenicity of some clostridial strains on MNs, the putative role of cyanobacteria in ALS, the relationship between gut microbiota and CNS, the role of adaptive immunity in ALS, and some published case reports, some researchers hypothesized a role of intestinal microbiota also in the pathogenesis of ALS.
**Preliminary data**	There are preclinical evidences of peculiar microbiota characteristics in ALS animal models and patients with respect to wild type animals and healthy controls.<br>Two pre-clinical studies, carried out in G93A transgenic models of ALS, investigated the correlation between gut dysbiosis, altered intestinal permeability and enteric inflammatory/neurogenic responses. Wu et al., (2015) found signs of leaky intestine in a G93A transgenic mouse model with a defective intestinal tight junction structure and related protein expression, leading to increased gut permeability, compared to wild-type mice. Normal epithelial Paneth cells, which impact the gut microbiome and help tune the innate immune response, were also decreased in number and function, with functionality measured as levels of specific antimicrobial proteins secreted into the gut. Shifts in the gut microbiome included a lower relative abundance of butyrate-producing bacteria such as Butyrivibrio fibrisolvens, Escherichia coli and Firmicutes. Likewise, Zhang et al. (2017) reported a correlation between gut dysbiosis and morphofunctional alterations of intestinal permeability in G93A animals, since the earliest stages of the disease. They demonstrated for the first time that the ALS-causing SOD1 mutation that leads to protein aggregate formation in neurons and skeletal muscle indeed forms aggregates also in the intestine of ALS mice and human intestinal epithelial cells. Moreover, they observed that, following treatment with butyrate, G93A mice displayed a restored gut microbiota homeostasis and intestinal epithelial barrier integrity, as well as an improvement of the disease. The leaking intestine might be at least partially responsible for the observed discordance between increased presymptomatic total daily energy intake and decreased presymptomatic body mass index in ALS patients.<br>In humans with ALS Fang et al. (2016) found significantly increased population of harmful microorganisms (genus Dorea) and significantly reduced population of beneficial microorganisms (genus Oscillibacter, Anaerostipes, Lachnospiraceae) and suggested that the imbalance in intestinal microflora constitution may cause a pro-inflammatory dysbiosis that could compromise the intestinal epithelial barrier and promote immune/inflammatory responses with consequent alterations and a role in ALS pathogenesis.<br>However, the authors did not evaluate whether, besides enteric bacteria, ALS patients were characterized by alterations of intestinal epithelial barrier and/or immune/inflammatory cells activation, data that will be evaluated in the present study. Moreover, these findings do not allow to firmly establish whether the alterations of the enteric bacterianeuro-immune network contribute to the pathophysiology of ALS, or whether they occur as a consequence of the central neurodegenerative processes.<br>A recent study (Mazzini et al., 2018) focused on microbiota analysis in ALS patients, and detected a clear cluster division between the bacterial profiles of ALS patients and the healthy subjects, mainly based on the presence/absence of bands in the profiles. The authors observed a lower DNA concentration in patients compared with controls, with a low abundance of Clostridium and yeast, and a high abundance of E. Coli and Enterobacteria, confirming that microbiota is modified in ALS patients and it could contribute to the pathogenesis of the disease.<br>Therefore, further investigations should be implemented to better clarify the role of enteric bacteria-neuro-immune network in ALS patients, since the earlier stages of the disease, and microbiota-based treatment approaches can represent a new therapeutic target for ALS.<br>Individual case reports of PALS reported benefits as a result of FMT. This research field is therefore highly innovative and is gathering increasing interest from the international scientific community.

Via Giardini, 1355
Baggiovara
41126 Modena
tel. : 059396111
fax.: 0593962409

**Study type**	X Interventional<br>☐ Laboratory<br>☐ Other/Specifications: ______________________________	☐**Non-interventional**<br>☐ Pre-clinical
**Research Setting**	☐ Single site<br>☐ Database-based	X **Multiple sites**<br>☐ Other______________________
**Study design**	Randomized, double blind, controlled, multicentre clinical trial	
**Sample size**	42	
**Main inclusion criteria**	- Patients diagnosed with a laboratory supported, clinically "probable" or "definite" amyotrophic lateral sclerosis according to the Revised El Escorial criteria (Brooks, 2000)<br>- Sporadic or familial ALS<br>- Female or male patients aged between 18 and 70 years old<br>- Disease duration from symptoms onset no longer than 18 months at the screening visit<br>- Patients treated with a stable dose of Riluzole (100 mg/day) for at least 30 days prior to screening<br>- Patients with a weight > 50 kg and a BMI ≥18<br>Patients with a FVC (Forced Vital Capacity) equal or more than 70% predicted normal value for gender, height, and age at the screening visit<br>- Patients able and willing to comply with study procedures as per protocol<br>- Patients able to understand, and capable of providing informed consent at screening visit prior to any protocol-specific procedures<br>- Use of effective contraception both for males and females	
**Main exclusion criteria**	- Known organic gastrointestinal disease<br>- History of gastrointestinal malignancy; ongoing malignancies<br>- Use of immunosuppressive or chemotherapy within the past 2 years<br>- Celiac disease and/or food (e.g.lactose) intolerance<br>- Previous gastrointestinal surgery<br>- Any condition that would make endoscopic procedures contraindicated<br>- Acute infections requiring antibiotics<br>- Antimicrobial treatment or probiotics 4 weeks prior to screening<br>- Severe comorbidities (heart, renal, liver failure); severe renal (eGFR< 30ml/min/1.73m2), or liver failure or liver aminotransferase (ALT/AST > 2x Upper limit of normal),<br>- Autoimmune diseases, inflammatory disorders (SLE, Rheumatoid arthritis, connective tissue disorder) or chronic infections (HIV, hepatitis B or C infection)<br>- Abuse of alcohol or drugs<br>- HIV, TBC, hepatitis<br>- Participation in clinical trials <30 days before screening<br>- Existing blood dyscrasia (e.g., myelodysplasia)<br>- White blood cells<4,000/mm³, platelets count<100,000/mm³, hematocrit<30%<br>- Patients who underwent non-invasive ventilation, tracheotomy and /or gastrostomy<br>- Women who are pregnant or breastfeeding	
**Study methods incl. treatment**	The study will include 42 ALS patients with 2:1 allocation in 2 groups of subjects (28 FMT vs 14 controls); computerized randomization will be stratified by ΔFS (</≥0.7) [21]. Randomization time: 18 months. Treatment will be double blinded to patients and physicians,and will be done at baseline and at month 6. Active treatment will be FMT. Post-treatment follow up: 6 months<br>ALS patients will undergo upper GI endoscopy with small-intestine biopsies (n° 4 biopsies of small intestine, performed with a standard biopsy forces) at baseline and after 6 months. At baseline patients will be randomized (2:1) to either an allogenic (from donors) infusion of collected feces (fecal microbiota transplantation, FMT) (or no procedure in case of allocation to control arm) in the duodenum-jejunum. The infusion will be performed through a standard nasojejunal tube, that will be placed during endoscopy. Infusion of feces will be performed at time 0 and repeated at month 6. In accordance with the current provisions of the national transplant center (CNT), fresh or frozen feces may be used (in relation to the need for screening for potential infectious diseases). Fecal microbiome will be diluted in saline solution 200 ml and infused at 30 ml/minute speed (whole amount of the performance: 15 minutes). The patients allocated to control arm will not receive any treatment but will undergo intestinal biopsy.<br>Upper GI endoscopy 12 months after FMT will be performed only to identify specific microbioma	

	and mucosal immunological evaluation. Fecal samples and small intestine biopsy samples (at baseline, before treatment, and at month 6 and 12) will be obtained from patients to perform the gut microbiota typing.<br>Every endoscopic procedure will be performed with deep sedation of the patient.<br>Feces for FMT will be obtained by habitual healthy donors for C. difficile infection. Procedures that are usually performed for the selection of donors for transplantation of feces are as follows. Potential donors stool had been chosen in healthy volunteers that have been given a questionnaire with questions about lifestyle, health status, current therapy, etc., significant clinical symptoms of gastrointestinal disease, etc.. Based on this questionnaire, the potential donor has been considered eligible if were excluded: I) Habits of life and risk behaviors, II) Concomitant significant known disorders, III) chronic or recent use of concomitant medications that may interfere with the state of the intestinal microflora (eg, antibiotics), IV) Clinical symptoms indicative of gastrointestinal disease or other diseases of importance, V) Personal or family medical history known of neurodegenerative diseases or other autoimmune diseases.<br>Moreover, each suitable potential donor has been subjected to the following screening tests: I) Examination of stool for *Clostridium difficile* bacterial pathogens and protozoa and helminths of the small intestine and colon, VRE (Vancomycin-resistant Enterococci), MRSA (Methicillin-resistant Staphylococcus aureus), Gram-negative MDR (Multidrug-Resistant Organisms), II) Serological screening for HAV, HBV, HCV, HIV 1-2, *Treponema pallidum*, *H. pylori*, blood count with differential, dose transaminasemia, creatinine and C-reactive protein.<br>Any further investigations aimed at excluding further potential infectious risks (e.g. SARS-Cov2 swab) will be performed in accordance with the current provisions of the National Transplant Center (CNT) regarding FMT.<br>Potential donors negative for this screening have been considered definitively suitable and will be invited to give a stool sample to prepare for the fecal transplantation. The same donor will give stool sample both for the first (baseline) and the second (month 6) FMT. The donation will be made in the appropriate circles in the UOC of Internal Medicine and Gastroenterology, and the preparation of feces (60 grams, manual homogenization in 200 mL of saline solution) for infusion will be performed at the UOC of Analysis 2 ° (Virology and Microbiology).<br>Analysis of T cell sub-populations will be performed both in peripheral blood and gut mucosa: especially the ratio T Regulatory cells (Tregs)/Th17 cells. A Contract Research Organization (CRO) will be in charge for centers' monitoring, and an authorized company for pharmacovigilance.
**Primary Endpoint**	Change from baseline to month 6 in Tregs number in ALS patients treated with FMT compared to the control arm.
**Secondary Endpoints**	**Clinical endpoints**<br>Comparison of the following variables between FMT group and control group:<br>- Change from baseline to each time point (month 1, 3, 6, 9, 12) of ALSFRS-R<br>- Survival defined as the time from randomization to the date of documented death or tracheotomy<br>- Change of Forced Vital Capacity (FVC) from baseline to each time point (month 1, 3, 6, 9, 12)<br>- Frequency of procedures (PEG, NIV, IV) from baseline to month 3,6,9,12<br>**Safety**<br>The Safety will be assessed on the occurrence of adverse events and per-treatment arising changes in physical examination, vital signs (blood pressure, pulse rate and body temperature), body weight, and clinical laboratory tests (biochemistry, haematology) in treated patients and controls.<br>**Quality of life**<br>Absolute and relative change from baseline in ALSAQ-40 (Amyotrophic Lateral Sclerosis Specific Assessment Questionnnaire) at month 6 and 12 in treated groups and control group.<br>**Biological endpoints**<br>- Change from baseline to each time point (month 3, 6, 9, 12) of the T cell distribution especially the ratio Tregs/Th1 or Tregs/Th17comparing FMT arm and control group.<br>- Changes from baseline to each time point (month 3, 6, 9, 12) in inflammatory status (citokine profile) comparing FMT and control group.<br>- Changes from baseline to each time point (month 3, 6, 9, 12) in different biomarkers (including peripheral and CSF biomarkers i.e. creatinine and albumin, CK, vitamin D, plasma/CSF neurofilament heavy/light chain protein) comparing FMT arm and control group.<br>- Changes from baseline to each time point (month 6 and 12) in the composition of saliva, gut and fecal microbiota comparing FMT and control group.
**Safety Evaluation**	**Safety assessment**:<br>✓ SCREENING VISIT: This visit will be performed in one of the recruiting Neurological ALS Centers (Modena, Firenze, Rome, Chieti, Perugia, Siena). Prior to any study activities, the

Via Giardini, 1355
Baggiovara
41126 Modena
tel. : 059396111
fax.: 0593962409

patient will be asked to read and sign an informed consent form that has been approved by the Independent Ethics Committees and which complies with regulatory requirements. Patients will be given adequate time to consider any information concerning the study given to them by the investigator. As part of the informed consent procedure, patients will be given the opportunity to ask the investigator any question regarding potential risks and benefits of a participation in the study. The following assessments and investigations will be conducted during this visit: written informed consent; give information form for general practitioner; document the relevant past medical history, any planned surgery and current medical conditions not related to the diagnosis of ALS; ALS history and diagnosis; medical history and procedure history; ALS medication history; concomitant medications and/or non-drug therapies, including the reason for administration; check of the patient's current practice for effective contraception; physical examination including vital signs, weight, height (BMI); neurological examination; MRC, ALSFRS-R and FVC measurement; urinalysis; blood sample will be collected for haematology and biochemistry; serum pregnancy test in females of child-bearing potential; screening for Hepatitis B, Hepatitis C, HIV; 12-lead ECG, Chest X-Ray, only if not performed at diagnosis or in the last 12 months; Check of inclusion/exclusion criteria; recording of symptoms present at screening; appointment for the next visit

- ✓ BASELINE VISIT: This visit will be performed in one of the recruiting Neurological ALS Centres (Modena, Firenze, Rome, Chieti, Perugia, Siena). The baseline visit can occur from 1 day and up to 3 weeks after the screening visit when all results from the screening evaluation are available, and study treatment appointment at Gemelli Policlinico in Rome is available. The following measures should be conducted before the randomization: check of the patient's current practice for effective contraception; check of inclusion/exclusion criteria; physical examination including vital signs, weight, height (BMI); blood sample will be collected for haematology and blood chemistry (analysis will not be repeated if Visit 2 occurs less than 7 days after Visit 1); urinalysis; neurological examination; MRC, ALSFRS-R and FVC measurement; ALSAQ – 40; lumbar puncture for biomarkers studies; blood sample for biological assessment and peripheral biomarkers; recording of menstrual cycles (non menopausal female patients); query for medical procedures, hospitalizations, changes in concomitant medications and therapies; randomization and treatment allocation.
  The following measures should be conducted after randomization: appointment for FMT to be performed at Gemelli Policlinico in Rome (within 4 weeks from screening); ask the patient to come back 4 week later.
- ✓ TREATMENT 1: This visit will be performed at Policlinico Gemelli in Rome. This visit can occur from 1 day and up to 4 weeks after the screening visit when all results from the screening evaluation are available. The following measures should be conducted before treatment: check of the patient's current practice for effective contraception; physical examination including vital signs, weight, height (BMI); blood, saliva and feces samples collection and gut biopsies; query for medical procedures, hospitalizations, changes in concomitant medications and therapies;
  Treatment will be then performed; the procedure will last approximately 15 minutes, will consist in deep sedation followed by gastroscopy and fecal infusion via naso-duodenal tube. The patients allocated in the control arm will not undergo any treatment; they will do intestinal biopsy alone.
  After the procedure the patient will be observed for 3 hours and then he could come back home; the appointment for the next treatment will be given (at six months).
- ✓ VISIT MONTH 1 TREATMENT PERIOD: This visit will be performed in one of the recruiting Neurological ALS Centres (Modena, Firenze, Rome, Chieti, Perugia, Siena). This visit will be performed at 1 month from treatment. The following assessments and investigations will be conducted at each visit: check of the patient's current practice for effective contraception; recording of adverse events; recording of menstrual cycles; query for medical procedures, hospitalizations, changes in concomitant medications and therapies; physical examination including vital signs, weight, height (BMI); neurological examination; MRC, ALSFRS-R and FVC measurement; blood sample will be collected for haematology and blood chemistry; urinalysis; appointment for the next visit
- ✓ VISIT MONTH 3 TREATMENT PERIOD: This visit will be performed in one of the recruiting Neurological ALS Centres (Modena, Firenze, Rome, Chieti, Perugia, Siena). This visit will be performed at 3 months from treatment. The following assessments and investigations will be conducted at each visit: check of the patient's current practice for effective contraception; recording of adverse events; recording of menstrual cycles; query for medical procedures, hospitalizations, changes in concomitant medications and therapies;

Via Giardini, 1355
Baggiovara
41126 Modena
tel. : 059396111
fax.: 0593962409

physical examination including vital signs, weight, height (BMI); blood sample will be collected for haematology and blood chemistry; urinalysis; neurological examination; MRC, ALSFRS-R and FVC measurement; blood sample for biological assessment and peripheral biomarkers; appointment for the next visit

- ✓ VISIT MONTH 6: This visit will be performed in one of the recruiting Neurological ALS Centres (Modena, Firenze, Rome, Chieti, Perugia, Siena). This visit will be performed 1 week to 1 day before the second treatment. The following measures should be conducted before the randomization: check of the patient's current practice for effective contraception; physical examination including vital signs, weight, height (BMI); blood sample will be collected for haematology and blood chemistry; urinalysis; neurological examination; MRC, ALSFRS-R and FVC measurement; ALSAQ – 40; lumbar puncture (LP) for biomarkers studies; blood sample for biological assessment and peripheral biomarkers; recording of menstrual cycles; query for medical procedures, hospitalizations, changes in concomitant medications and therapies; ask the patient to come back 4 week later.
- ✓ TREATMENT 2: This visit will be performed at Policlinico Gemelli in Rome. This visit can occur from 1 day and up to 1 week after visit month 6. The following measures should be conducted before treatment: check of the patient's current practice for effective contraception; physical examination including vital signs, weight, height (BMI); blood, saliva and feces samples and and gut biopsies collection; query for medical procedures, hospitalizations, changes in concomitant medications and therapies.

  Treatment will be then performed; the procedure will last approximately 15 minutes, will consist in deep sedation followed by gastroscopy and fecal infusion via naso-duodenal tube. The patients allocated in the control arm will not undergo any treatment; they will do intestinal biopsy alone.

  After the procedure the patient will be observed for 3 hours and then he could come back home; the appointment for the next visit will be given (at six months).
- ✓ FOLLOW UP VISIT, MONTH 7: This visit will be performed in one of the recruiting Neurological ALS Centres (Modena, Firenze, Rome, Chieti, Perugia, Siena). This visit will be performed 1 month after the second treatment. The following assessments and investigations will be conducted at each visit: check of the patient's current practice for effective contraception; recording of adverse events; recording of menstrual cycles (non menopausal female patients); query for medical procedures, hospitalizations, changes in concomitant medications and therapies; physical examination including vital signs, weight, height (BMI); neurological examination; MRC, ALSFRS-R and FVC measurement; appointment for the next visit
- ✓ FOLLOW UP VISIT, MONTH 9: This visit will be performed in one of the recruiting Neurological ALS Centres (Modena, Firenze, Rome, Chieti, Perugia, Siena). These visits will be performed at month 9. The following assessments and investigations will be conducted at each visit: check of the patient's current practice for effective contraception; recording of adverse events; recording of menstrual cycles; query for medical procedures, hospitalizations, changes in concomitant medications and therapies; physical examination including vital signs, weight, height (BMI); neurological examination; MRC, ALSFRS-R and FVC measurement; blood sample will be collected for haematology and blood chemistry; urinalysis; blood sample for biological assessement and peripheral biomarkers; appointment for the next visit.
- ✓ FOLLOW UP VISIT, MONTH 12: This visit will be performed at Policlinico Gemelli in Rome. This visit will be performed at 12 months from the first treatment. The following measures should be conducted before treatment: check of the patient's current practice for effective contraception; physical examination including vital signs, weight, height (BMI); blood, saliva and feces samples collection; query for medical procedures, hospitalizations, changes in concomitant medications and therapies. GI endoscopy and gut biopsies will be performed.

  After the procedure the patient will be observed for 3 hours and then he could come back home; the appointment for the next visit will be given (at six months).
- ✓ END OF STUDY VISIT (MONTH 12): This visit will be performed in one of the recruiting Neurological ALS Centres (Modena, Firenze, Rome, Chieti, Perugia, Siena). This visit will be performed 1 week to 1 month after follow up visit at month 12. The following assessments and investigations will be conducted at each visit: check of the patient's current practice for effective contraception; recording of adverse events; recording of menstrual cycles (non menopausal female patients); query for medical procedures, hospitalizations, changes in concomitant medications and therapies; physical examination including vital signs, weight, height (BMI); neurological examination; MRC, ALSFRS-R, ALSAQ - 40 and FVC

Via Giardini, 1355
Baggiovara
41126 Modena
tel. : 059396111
fax.: 0593962409

	measurement; blood sample will be collected for haematology and blood chemistry; urinalysis; blood sample for biological assessment and peripheral biomarkers.<br><br>**Data recording and study monitoring:** All data will be recorded by an electronic CRF. The study will be monitored by a certified contract research organization (CRO).<br><br>**An independent DSMB will be established**, to address safety and efficacy concerns that could arise during the study. Reports including all relevant clinical data of patients will be sent by the Statistics Unit of the University of Modena to an independent DSMB for scheduled DSMB meetings (also through Skype) when 14 patients have done the second treatment and subsequently after every 6 months.<br><br>**An Independent Ethics Advisory Board (IEAB)** will be established to monitor study procedures, from the ethic point of view, with particolar attention to the informe consent form collection. The informed consent form (ICF) will be obtained by a neurologist not involved in the care relationship with the patient. In the patient information sheet that will be given to the patient at least two weeks before the ICF collection and screening, the patient could find a telephone number of the IEAB, that he/she could call in case of further information needed from an independent position. On the same day when the neurologist will give the information sheet to the patient, the investigator will advise the IEAB members of a potential enrollment to allow them to be present at the moment of the ICF collection (optional). The IEAB will send to the Ethics Committee a report within 2 months from study actual starting date and another report at the end of the study.<br><br>**Stopping rules for safety reasons:**<br>To establish stopping rules, we will consider the following list of toxicities as acceptable: diarrhea, abdominal cramping and pain, nausea, vomiting, flatulence, fever, constipation, dizziness, sore throat, rhinhorrea, bloating, nasal stuffiness, urinary infections, headache, mild/moderate blood cells alterations; elevated CRP, rashes, urticaria, dermatoses, dermatitis (mild/moderate severity), elevated AST/ALT/ALP, loss of appetite, colitis/gastroenteritis.<br>A procedure consisting of stopping the trial in case of excess of AE recording the treating group in case of 30% of the occurrence of the following: peritonitis, upper gastrointestinal hemorrhage, sepsis, bacteriemia.
**Sample size justification**	The sample size has been estimated considering as the primary outcome measure the proportion of positive response (Tregs number increase) at treatment end (6 MONTHS) in pts treated with FMT vs controls.<br><br>ALS pts have a slight reduction of Tregs% (mean±SD:2.1±0.7) with respect to healthy controls (2.6±0.6) (Tregs% calculated on total lymphocytes; normal values of total lymphocytes: 1000-4500/mmc; normal values of total Tregs: 71.5±17/mmc) (Mantovani et al., 2009).<br>Slowly progressive ALS pts have a number of Tregs that is equal to healthy controls, whereas fast progressors have 31% fewer Tregs than slowly progressing pts, and Tregs % is inversely correlated with the rate of disease progression (Beers,2011). These data indicate that ALS pts have 60±17 Tregs/mmc (fast progressors: 49.3 Treg/mmc; slow progressors: 71.5 Tregs/mmc). As a result, a “positive response” can be considered an increase of the proportion of Tregs by at least 20%. The null hypothesis is that FMT does not increase significantly the proportion of positive responses in treated pts after the second FMT round compared to their baseline stage and to control group. The alternative hypothesis is that FMT determines a proportion of positive responses in at least 50% of treated patients compared to a maximum 5% of patients in the control group. The study has been designed to reject the null hypothesis with an alpha error of 0.05 (in order to take into account a multiple comparison with a control arm) and a power of 0.80. For this purpose, a sample of 39 pts randomized in 2 treatment arms would be needed. Considering an average drop out of 10% then a recruitment of 42 patients will be necessary.
**Statistics**	**Data management and statistical plan:** Unit 7 will be in charge for this work (details in WP4). An independent Data and Safety Monitoring Committee will be established with several meetings aimed at preserving safety (see safety evaluation box).<br>**Statistical methods:**<br>Separate analyses will be performed in:<br>1. All randomized subjects receiving at least 1 FMT (**Intention-to-treat population**);<br>2. All randomized subjects excluding protocol deviations (**Per protocol, PP population**).<br>**Descriptive statistics** will be performed comparing the groups of FMT treatment and controls.

Via Giardini, 1355
Baggiovara
41126 Modena
tel. : 059396111
fax.: 0593962409

	Continuous variables will be described using mean and S.D. or median and I.Q. range; categorical variables will be described as counts and percentages.<br>**Biological activity:** Immune response to FMT will be analyzed as the difference in positive response to FMT (mean Tregs increase >20%) between the control group and the FMT groups. This will be calculated with the use of Tregs data obtained at baseline and at month 6.<br>We will compare the mean values of different T, cell subpopulations, of biomarkers, cytokines, comparing baseline and treatment end (month 6) between FMT and control group. Mean differences in plasma concentrations from baseline to month 6 in the 2 treatment arms will be calculated and compared using t-test or Wilcoxon-Mann-Whitney test.<br>The mean change over time for the same variables as above will be assessed using repeated measures ANOVA, with treatment as between-subjects factor and time as within-subjects factor. Different models will be used, each with a different biomarker of activity as the dependent variable. Models will be adjusted for any unbalanced distribution of the main prognostic factors (e.g. age) between the two treatment arms.<br>**Analysis of microbiota** will be carried out through specific softwares.<br>**Safety analysis** will be performed in all subjects receiving at least one FMT round. All AEs and SAEs will be recorded according to ICH Guidelines and compared in the treatment arms at any follow-up visit and at study end.<br>Differences in tracheostomy-free survival (Kaplan-Meier method) between the treated groups and control group will be compared using the log-rank test. Cox's proportional hazard model would be used to adjust for any possible unbalanced prognostic factors. Statistical significance will be set at 0.05 level for a two-tailed test. Missing data will be handled using the last observation carried forward.<br>**FMT efficacy:** The comparison of clinical endpoint among arms will be carried out by using the logistic regression model. Results will be presented as odds ratios (OR) and 95%C.I. FMT will be considered effective in relation to control group whether the OR of positive results will provide a p-value<0.05.
**Study Start date**	15th April 2019
**Study Completion date**	15th April 2022

PROTOCOL SIGNATURE PAGE

PROTOCOL TITLE: INTERPLAY BETWEEN GUT MICROBIOTA AND ADAPTIVE IMMUNITY IN AMYOTROPHIC LATERAL SCLEROSIS: A CLINICAL TRIAL

PROTOCOL CODE: FETR-ALS

COORDINATING INVESTIGATOR AND SPONSOR SIGNATURE:

______________________________ **___03/04/2019________**

Dr.ssa Jessica Mandrioli **Date**
(Azienda Ospedaliero Universitaria di Modena)

Investigator Agreement: I have read the protocol and agree to

- Implement and conduct this study diligently and in strict compliance with the protocol, good clinical practices and all the applicable laws and regulations including the Declaration of Helsinki and applicable National Laws.
- Maintain all information in confidence.
- Recognize that any substantial changes in Study Protocol must be approved in writing by the Coordinating Investigator, the Sponsor, the corresponding authorities, and the Ethics Committee before implementation except when necessary to eliminate immediate hazards to subjects or when the changes involve only logistical or administrative aspects of the study.

I have read this protocol in its entirety and I agree to all parts.

______________________________ ______________________________
Investigator's Signature Date

______________________________ ______________________________
Investigator's Name Investigator's Address

____

____

TABLE OF CONTENT

1. GLOSSARY OF ABREVIATIONS AND DEFINITION OF TERM 15
2. GENERAL INFORMATION 17
3. BACKGROUND & RATIONALE 18
4. PRELIMINARY DATA 24
5. OBJECTIVES 27
5.1 PRIMARY OBJECTIVE 27
5.2 SECONDARY OBJECTIVEs 27
5.2.1 Biological assessment 27
5.2.2 FMT safety and tolerability in a cohort of ALS patients 27
5.2.3 Clinical assessment 27
5.2.4 Quality of Life assessment 27
6. INVESTIGATIONAL PLAN 28
6.1 STUDY DESIGN 28
6.2 STUDY FLOW CHART 28
6.3 WORK PLAN 31
6.3.1 WP1: management and Project Coordination 34
6.3.2 WP2: RCT conduction 35
6.3.3 WP3: Preparation of fecal material 38
6.3.4 WP4: Laboratory Assessment 39
6.3.5 WP5: Analysis of data and results dissemination 44
7. STUDY POPULATION 45
7.1 INCLUSION CRITERIA 46
7.2 EXCLUSION CRITERIA 46
7.3 PATIENT IDENTIFICATION 47
7.4 DONORS SELECTION 47
8. STUDY TREATMENT 50
8.1. STUDY TREATMENT DEFINITION 50

Via Giardini, 1355
Baggiovara
41126 Modena
tel. : 059396111
fax.: 0593962409

8.2. PATIENT COMPLIANCE 51

8.3. CONCOMITANT MEDICATIONS 51

8.3.1. Mandatory Concomitant Treatment 51

8.3.2. Prohibited Concomitant Treatment 51

8.4 RANDOMIZATION 52

8.5. BLINDING PROCEDURES 52

8.6. MANAGEMENT OF TOXICITY 53

8.6.1. Procedures to manage adverse events potentially related to study treatment 53

9. EVALUATION CRITERIA 55

9.1. PRIMARY ENDPOINT 55

9.2. SECONDARY ENDPOINTS 55

9.2.1. Biological Activity Assessment 55

9.2.2. SAFETY 56

9.2.3. Clinical Assessment 57

9.2.4. Quality of Life 57

10. ASSESSMENT SCHEDULE 58

10.1. **SCREENING VISIT** 58

10.2. **BASELINE VISIT** 59

10.3 TREATMENT 1 59

10.4 VISIT MONTH 1 TREATMENT PERIOD 60

10.5. VISIT MONTH 3 TREATMENT PERIOD 60

10.6. VISIT MONTH 6 61

10.7 TREATMENT 2 61

10.8. FOLLOW UP VISITS, MONTH 7 62

10.9. FOLLOW UP VISIT, MONTH 9 62

10.10 FOLLOW UP VISIT, MONTH 12 63

10.11 END OF STUDY VISIT (MONTH 12) 63

10.12 witHDRAWAL OF PATIENTS 64

11. STATISTICAL METHODS 64
11.1. SAMPLE SIZE JUSTIFICATION 64
11.2 STATISTICAL ANALYSIS 65
11.3 STOPPING RULES FOR SAFETY REASONS 66
12. DATA RECORDING AND STUDY MONITORING 67
13. DATA MANAGEMENT 67
14.ETHICS 67
14.1 ETHICAL CONSIDERATIONS 67
14.2 SUBJECT INFORMATION AND INFORMED CONSENT 67
14.3 PATIENT WITHDRAWALS AND DROPOUTS 68
14.4 ETHIC COMMITTEE APPROVAL 68
14.5 INDEPENDENT BOARDS 69
15. ADMINISTRATIVE CONSIDERATIONS 69
16. REFERENCES 71
17. APPENDIXES 76
17.1. ADVERSE EVENTS 76
17.1.1. Definition of Adverse Event 76
17.1.2. Definition of Serious Adverse Event 76
17.1.3. Definition of Serious Adverse Reaction 76
17.1.4. Reporting of Adverse Events 77
17.1.5. Reporting of Serious Adverse Events/reactions 77
17.1.6. Causality Assessment 79
17.1.7. Intensity of Adverse Events 79
17.1.8. Actions Taken in Response to an Adverse Event 80
17.1.9. Follow-Up on Ongoing Adverse Events 80
17.1.10. Follow-up form to be sent every six months to italian national transplantation centre 81
17.2 DECLARATION OF HELSINKI 83
17.3 ITALIAN REGULATION ON CELLs/TISSUEs TRANSPLANTATION 88
17.4 ITALIAN VERSION OF THE ALSFRS-R 105

17.5 ITALIAN VERSION OF ALSAQ-40......108

17.6 ITALIAN VERSION OF MRC......111

Via Giardini, 1355
Baggiovara
41126 Modena
tel. : 059396111
fax.: 0593962409

1. GLOSSARY OF ABREVIATIONS AND DEFINITION OF TERM

AE	Adverse Event
ALS	Amyotrophic Lateral Sclerosis
ALSFRS-R	Amyotrophic Lateral Sclerosis Functional Rating Scores – Revised
ALSAQ-40	Amyotrophic Lateral Sclerosis Assessment Questionnaire
ALT	Alanine Aminotransferase
AST	Aspartate Aminotransferase
BAG3	BCL2 associated athanogene 3
BMI	Body Mass Index
CNS	Central Nervous System
CRF	Case Report Form
CSF	Cerebrospinal Fluid
C9ORF72	Chromosome 9 open reading frame 72
ΔFS	Disease progression rate
EC	Ethics Committee
ECG	Electrocardiogram
eCRF	Electronic CRF
EDC	Electronic Data Capture
EMA	European Medicines Agency
FDA	Food and Drug Administration
FMT	Fecal microbiota transplantation
FTD	Fronto-Temporal Dementia
FTLD	Fronto-Temporal Lobar Degeneration
FUS	Fused in sarcoma
FVC	Forced Vital Capacity
Gamma GT	Gamma-Glutamyl Transferase
GCP	Good Clinical Practice
GMP	Good Manufacturing Practice
GM	Gut Microbiota
HSP70	Heat shock protein 70
HSPB8	Heat shock protein B8
IMP	Investigational Medicinal Product
ITT	Intent To Treat
IV	Intravenous
LDH	Lactate Dehydrogenase
LO	Last Observation
MRC	Medical Research Council
PLT	Platelet
PP	Per Protocol
PR	Partial Response
OPTN	Optineurin
QOL	Quality of Life
R-EEC	Revised El Escorial Criteria
SAE	Serious Adverse Event
SOD1	Superoxide dismutase 1
SOP	Standard Operating Procedure
SPC	Summary of Product Characteristics
SQSTM1	Sequestosome 1

TDP-43	TAR DNA-binding protein, 43-KD
T-reg	Regulatory T Cells
UBI	Ubiquinate Inclusions
UBQLN2	Ubiquilin 2
US	Ultrasound
ULN	Upper Limit of Normal
VC	Vital capacity

2. GENERAL INFORMATION

PROJECT TITLE	INTERPLAY BETWEEN GUT MICROBIOTA AND ADAPTIVE IMMUNITY IN AMYOTROPHIC LATERAL SCLEROSIS: A CLINICAL TRIAL
ACRONYM	FETR-ALS
PRINCIPAL INVESTIGATOR	JESSICA MANDRIOLI
HOST INSTITUTION	DEPARTMENT OF NEUROSCIENCE, AZIENDA OSPEDALIERO UNIVERSITARIA – MODENA
CONSORTIUM AND INVESTIGATORS	1) PROF.SSA TERESA SPANU, PROF. LUCA MASUCCI, DEPARTMENT OF MICROBIOLOGY – POLICLINICO UNIVERSITARIO "A. GEMELLI", ROME<br>2) DR.SSA ELENA NICCOLAI, PROF. AMEDEO AMEDEI, DEPARTMENT OF EXPERIMENTAL AND CLINICAL MEDICINE, UNIVERSITY OF FLORENCE, FLORENCE<br>3) PROF. ROBERTO D'AMICO, UNIVERSITY OF MODENA AND REGGIO EMILIA, MODENA<br>4) DR MARIO SABATELLI, DR.SSA AMELIA CONTE NEUROMUSCULAR OMNICENTRE CENTRE, 1NEUROMUSCULAR OMNICENTRE (NEMO) FONDAZIONE SERENA ONLUS-FONDAZIONE POLICLINICO A. GEMELLI, ROME; INSTITUTE OF NEUROLOGY, CATHOLIC UNIVERSITY OF THE SACRED HEART, FONDAZIONE POLICLINICO A. GEMELLI, ROME<br>5) PROF. VINCENZO DI LAZZARO, INSTITUTE OF NEUROLOGY, POLICLINICO CAMPUS BIO-MEDICO, ROME<br>6) DR.SSA SABRINA MATA, PROF. SANDRO SORBI, DIPARTIMEN-TO NEUROMUSCOLOSCHELETRICO E DEGLI ORGANI DI SENSO, AZIENDA OSPEDALIERO-UNIVERSITARIA CAREGGI, FLORENCE<br>7) DR SSA TERESA ANNA CANTISANI, DR.SSA PAOLA BRUNORI, UO NEUROFISIOPATOLOGIA, AZIENDA OSPEDALIERA DÌ PERUGIA<br>8) DR.SSA MARGHERITA CAPASSO, PROF. MARCO ONOFRJ, CLINICA NEUROLOGICA, OSPEDALE CLINICIZZATO "SS ANNUNZIATA", CHIETI<br>9) PROF. GIOVANNI CAMMAROTA, DIPARTIMENTO DI MEDICINA INTERNA E SCIENZE SPECIALISTICHE, POLICLINICO UNIVERSITARIO "A. GEMELLI", ROMA

Via Giardini, 1355
Baggiovara
41126 Modena
tel. : 059396111
fax.: 0593962409

	10) PROF. FABIO GIANNINI, DIPARTIMENTO DI SCIENZE MEDICHE, CHIRURGICHE E NEUROLOGICHE, UNIVERSITÀ DI SIENA, OSPEDALE "LE SCOTTE" , SIENA
**PROJECT DURATION**	36 months

3. BACKGROUND & RATIONALE

Several mechanisms have been proposed to account for the pathogenetic process of Amyotrophic lateral sclerosis (ALS). These include protein misfolding, oxidative stress, axonal transportation damage, mitochondrial abnormalities, glutamate-mediated excitotoxicity, and altered response to hypoxia. Microglial activation also plays a role in the pathogenesis of ALS and other neurodegenerative disorders. At any rate motor neurons (MNs) death invariably leads to severe disability and death in a few years after diagnosis, but the clinical course may vary greatly from one patient to another and no accepted prognostic factors are currently available. The only drug showing to improve survival in patients with ALS is riluzole. However, the benefits consist in a three-month delay of death while disability and other outcome measures are virtually unaffected (van Es et al., 2017). Recently, Food and Drug Administration (FDA) and Agenzia Italiana del Farmaco (AIFA) approved treatment with Edaravone for ALS for a limited number of patients with very mild disability and very recent disease onset, based on an observed slowing of disease progression as measured by ALSFRS-R in a subgroup of patients (Hardiman and van den Berg, 2017). The lack of effective treatment for ALS urges new therapeutic approaches for treatment in humans affected by the disease.
This trial aims at evaluating the biological basis of a potential treatment for ALS in order to plan a following efficacy study on fecal microbiota transplantation for this disease.
One of the reasons accounting for the failure of almost all previous clinical trials in ALS is the uncomplete comprehension of the pathogenic processes that cause disease onset and progression. While in the past ALS was considered as caused by isolated motorneuron degeneration, it has been demonstrated that ALS pathology is characterized not only by MNs death, but also by infiltrating lymphocytes, and early microglia activation (Boillée et al., 2006). Microglia is the most abundant resident immune cells in the brain, it is involved in neurodevelopment, phagocytosis, antigen presentation, cytokine production and activation of inflammatory responses (Nayak et al., 2014). Microglia involvement in ALS is so important that it is a target of many recent clinical trials in ALS (e.g. trials testing the following drugs: masitinib, GCSF; but also trials using stem cells like Neurown). With the use of mice carrying a deletable mutant gene, expression within motor neurons was shown to be a primary determinant of disease onset and of an early phase of disease progression. Diminishing the mutant levels in microglia had little effect on the early disease phase but sharply slowed later disease progression. Onset and progression thus represent distinct disease phases defined by mutant action within different cell types to generate non-cell-autonomous killing of MNs; these findings validate therapies, including cell replacement, targeted to the non-neuronal cells in ALS (Boillée et al., 2006). Moreover, in ALS there is simultaneous activation of distinct lymphocyte populations, Th1 and Th17, and a decrease of T regulatory cells (Tregs) (Saresella et al., 2013) that have a protective role as demonstrated in both mice with ALS and in patients with ALS: a greater number of Tregs is associated with slow disease progression (Beers et al., 2011; Henkel et al., 2013). Tregs have been shown to directly differentiate macrophages from M1 to M2

state (Tiemessen et al., 2007) and M2 microglia has been associated with the stable disease phase, whereas Th1L and M1 microglia predominate during the rapidly progressing phase, suggesting a shift from protection to toxicity.

Intestinal microbiota includes a complex ecosystem with an exceptionally high bacterial density and diversity (**Figure 1**). The bacteria living in the human GI tract achieve the highest cell densities recorded for any ecosystem: the adult alimentary tract contains 1–2 kg of microbial cells of hundred bacterial species, of which over 80% have not been cultured. (Backhed et al. 2005; Eckburg et al., 2005; Conway 1997)

Figure 1: Damage to the immune system typically represents a break in the continual development of immune cells in the lining of the intestines. Immune cells development in the gut is dependent on the gut flora (**1A**). Damage to the gut flora, e.g. by antibiotics, processed foods that lack flora feeding fiber or extreme diets, disrupts development of immune cells. Typically, loss of the immune cells that keep the aggressiveness of the immune system in check, regulatory T cells or Tregs, results in autoimmunity (**1B**).

1A

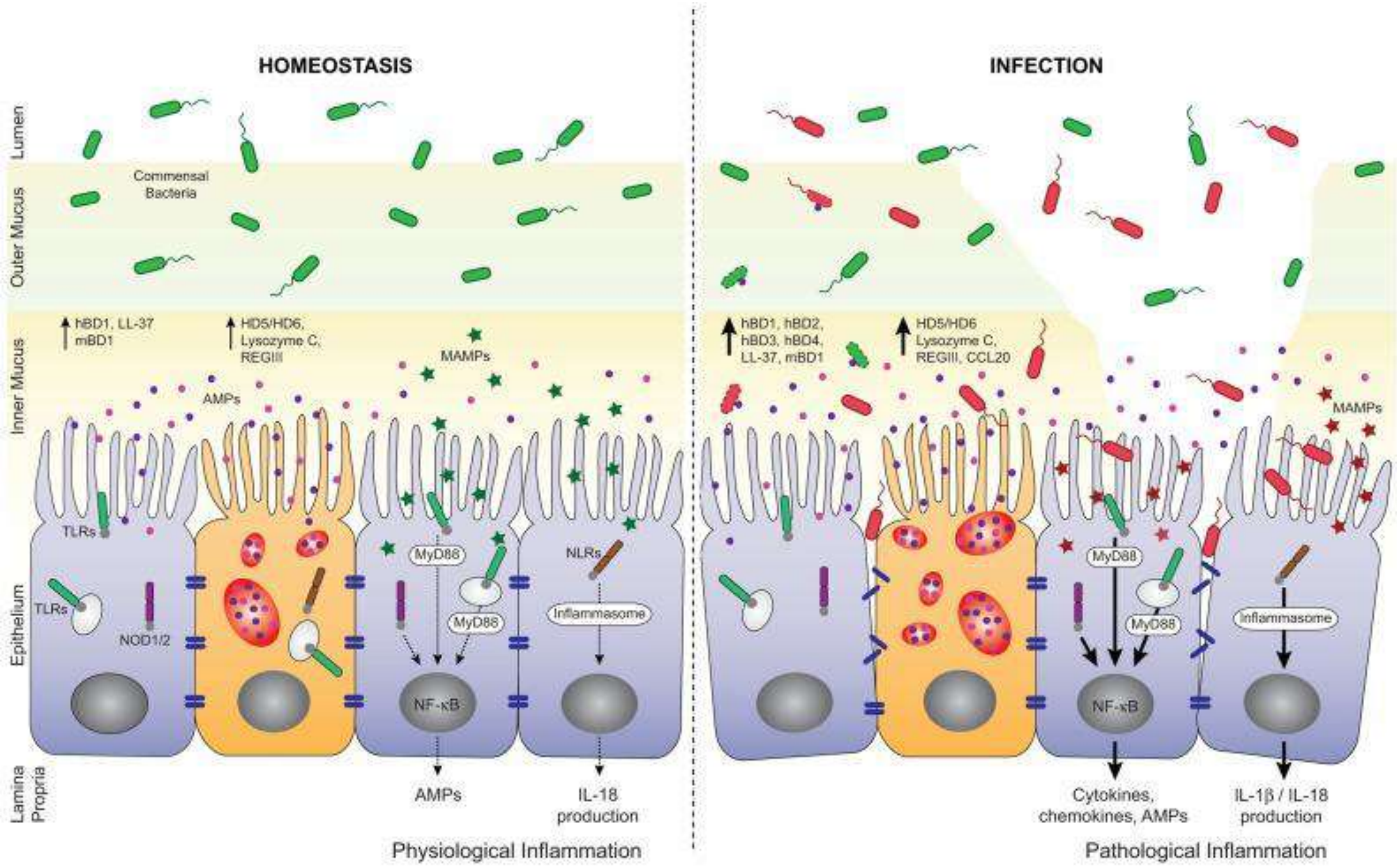

Via Giardini, 1355
Baggiovara
41126 Modena
tel. : 059396111
fax.: 0593962409

1B

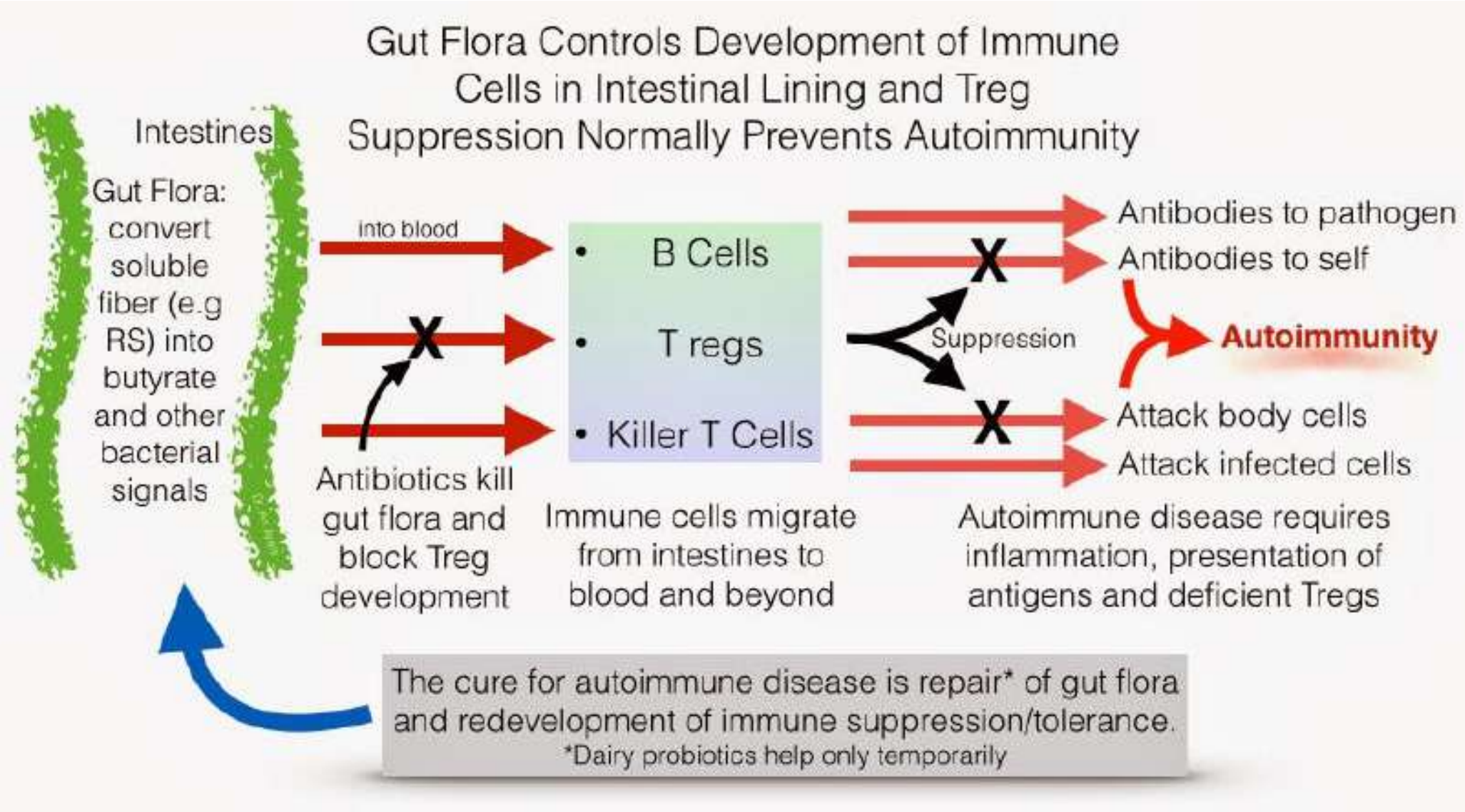

Gut microbiota (GM) interacts directly with the enteric immune system, contributing to the maintenance of immune tolerance and shaping the immune responses during inflammation (Shi et al., 2017). Conversely, upon pathogen invasion, dysbiosis or barrier break, microbe-associated molecular patterns stimulate macrophages and dendritic cells to produce pro-inflammatory cytokines that, in turn, activate adaptive immune cells, thus contributing to the breakdown of immune homeostasis (Maynard et al., 2012).
Besides innate immune cells, enteric bacteria can directly affect adaptive immune responses. The main components of the adaptive immune system are T cells, in particular CD4 + and CD8 + T cells (Wu et al., 2012). Upon stimulation, naïve CD4 + T cells can differentiate into seven subtypes: T helper 1 (Th1), Th2, Th9, Th17, Th22, regulatory T cells (Treg) or T follicular helper (Tfh) cells, which express different arrays of transcription factors and cytokines (Wu at al., 2012). The GM influences the development and differentiation of both CD4+ and CD8+ T cells. Indeed, germ-free (GF) mice display a marked decrease in the number of both CD4+ and CD8+ T cells, and treatment with SCFA mixtures is associated with an increase in the density of T cells (Smith et al., 2013, Wu et al., 2012). Taken together, these findings suggest that the GM and the enteric immune system interact continuously to maintain a complex dynamic equilibrium supporting the intestinal homeostasis (Louveau et al., 2015; Rooks et al., 2016).
Alterations of enteric bacteria, besides determining the breakdown of intestinal homeostasis, have been found to affect several brain biological processes. In a recent study, Erny et al. observed that GF mice displayed an altered density, morphology and maturity of microglia, and that treatment with a SCFA mixture restored the density and morphology of microglia, thus suggesting that GM can influence both the development and functions of CNS immune cells (Erny et al., 2015). Moreover GM contribute to preserve the BBB integrity (Braniste et al., 2014).
In this context, microbiota gut-brain axis (**Figure 2**) is currently regarded as the key player in the regulation of mutual signaling between gut microflora and CNS (Fung et al., 2017) employing bidirectional communication via neuronal, hormonal, immunologic and toxic signalling (Bienenstock et al., 2015; Mayer et al., 2015).
Further proposed mechanisms include direct communication through the vagus nerve, changes in tryptophan and norepinephrine metabolism, production and absorption of neuroactive metabolites,

Via Giardini, 1355
Baggiovara
41126 Modena
tel. : 059396111
fax.: 0593962409

immune activation through molecular mimicry, and the direct production of neurotoxins (Tremlett et al., 2017).

Enteric bacteria and their metabolites (e.g., SCFAs) can indeed stimulate directly the enterochromaffin cells to produce several neuropeptides (e.g., peptide YY, neuropeptide Y, cholecystokinin, glucagon-like peptide-1 and -2, and substance P) or neurotransmitters (e.g., serotonin), which, in turn, can diffuse into the blood stream, reach the brain and influence directly CNS functions. In addition, the intestinal epithelium regulates the translocation of specific bacterial products (e.g., SCFAs, vitamins or neurotransmitters, such as acetylcholine, dopamine, noradrenaline, gamma-aminobutyric acid or serotonin) into the blood stream, which, in turn, through the circulatory system, can spread upwards to the CNS (Fung et al., 2017)

In this way, circulating microbiota-derived metabolites, neuropeptides and neurotransmitters can enter the CNS and influence directly its neurobiology.

Figure 2: the gut-brain axis

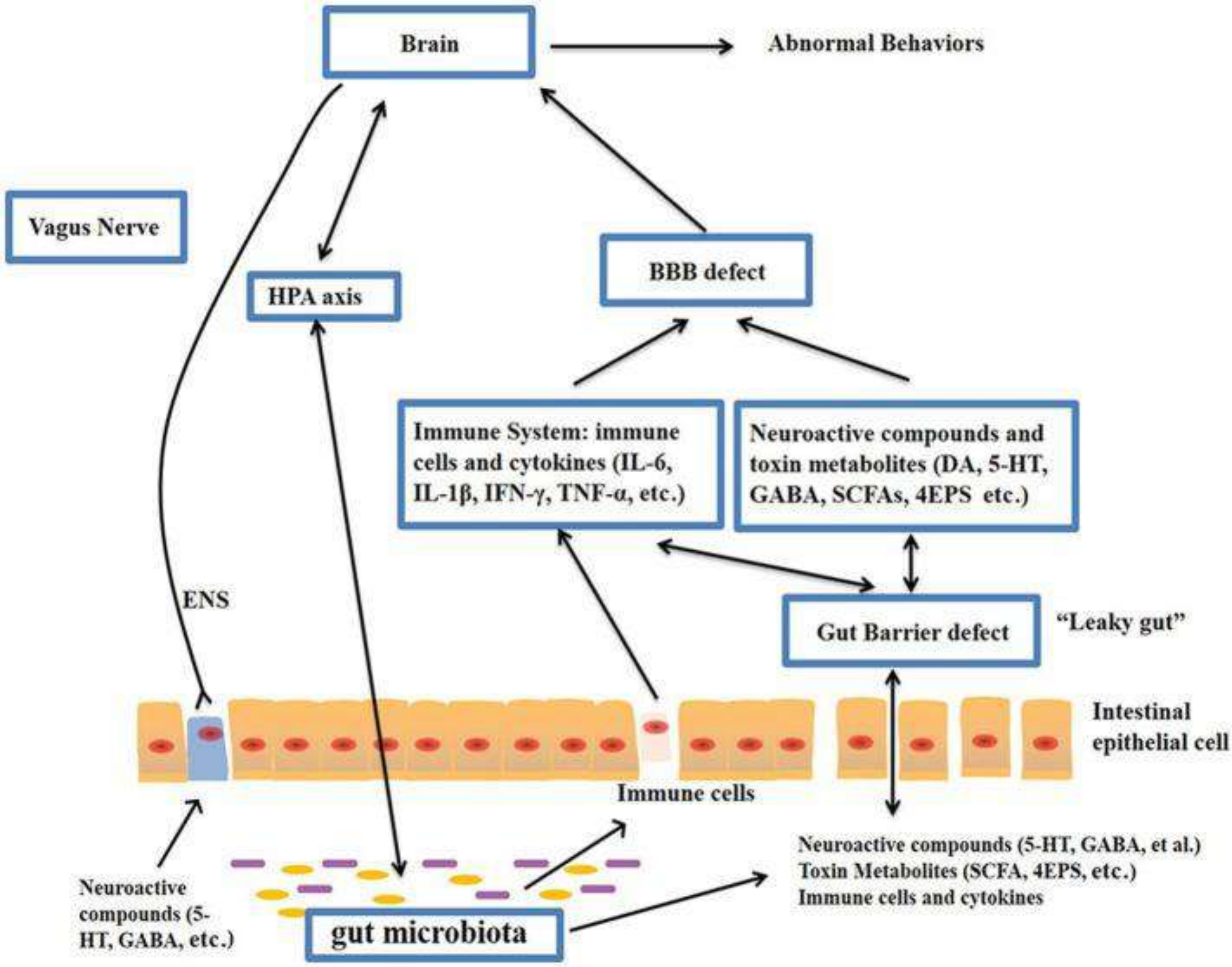

Via Giardini, 1355
Baggiovara
41126 Modena
tel. : 059396111
fax.: 0593962409

Another link between the GM and ALS may be seen in the potential role of pathogenic environmental factors: some researchers have postulated that neurotoxins produced by related species may be neuro-pathogenic (Longstreth et al., 2005) and that ALS may be caused by an as-yet-unidentified motor neuron toxin produced by a clostridial species in susceptible patients.

Current research lines on ALS include the hypothesis of neurotoxins produced by cyanobacteria, as a possible contributor to ALS and to the presence of geographical cluster of the disease near contaminated waters (Bradley et al., 2013)

Interestingly, several pathogenic clostridial strains are well known to cause neuromuscular disease, such as *C. botulinum* and *C. tetani* – which produce neurotoxins that can infect lower motor neurons, and in the case of tetanus, utilize retrograde signaling to the CNS. Given the well characterized pathogenicity of these clostridial strains, the frequency of constipation in many chronic neurologic disorders, and published case reports in which FMT and/or antibiotic regimens targeting clostridial infections appeared to serendipitously attenuate neurologic symptoms, some researchers have postulated that neurotoxins produced by related species may be neuro-pathogenic. There are animal data demonstrating that clostridial toxins, such as tetanus toxin, can be transported to the CNS: the non-toxic C fragment of tetanus toxin is known to be transported in a retrograde fashion to the CNS, and thus has been studied as a potential vehicle to target therapies for neurodegenerative disorders. In one study, naked DNA encoding the non-toxic C fragment of tetanus toxin was injected intramuscularly into SOD1-G93A mice and was transported retrograde and trans-synaptically back to the spinal cord, and surprisingly had therapeutic benefits in the ALS mice (Moreno-Igoa et al., 2010).

We can assume that a colonization of ALS patients with certain species of bacteria that produce neurotoxins on the motor neuron can influence the disease in susceptible patients. An alteration in the intestinal bacterial flora as an external trigger could explain the rare cases of ALS in spouses or in some clusters (Sabel et al. 2003).

The potential role of GM dysbiosis in facilitating the onset of ALS or driving disease progression and related outcomes, can be hypothesized to act though three different paths that are not necessarily mutually exclusive. Specifically, GM dysbiosis may contribute to disease onset, and also result from disease presence. The GM composition, combined with the presence of other risk factors, may play a role in disease causation. In other words GM could represent one of several possible risk factors that when combined would cause disease. Additionally, GM dysbiosis may be (further) altered by the disease presence and in some individuals contribute to disease progression, prognosis, also in terms of variable response to drug treatments (Tremlett et al., 2017).

Circumstantial evidence includes the overlap between many of the potential risk factors for ALS and the GM; environmental factors associated with ALS onset such as smoking, viruses, and vitamin D can also profoundly impact the GM, as can host genetics, and a person's age and sex (Tremlett et al., 2017)

Based on these premises, to treat GM dysbiosis through microbiota restoration would have the potential to interfere with the progression of the disease (Khanna 2018).

Fecal Microbiota Transplantation (FMT) is an intervention in which the fecal microbiota from a healthy individual is delivered to a patient with a dysbiotic GM **(Figure 3)**. FMT dates back to fourth-century China, when a traditional Chinese medicine doctor described an oral human fecal suspension that effectively cured food poisoning and severe diarrhea (Zhang et al., 2012).

In the 16th century, Li Shizhen described oral fecal preparations to treat diarrhea, vomiting, or constipation (Zhang et al., 2012).

In the 17th century, FMT was being used in veterinary medicine (Brandt 2015).

In 1958, Eiseman et al. treated four patients with pseudomembranous colitis from Micrococcus pyogenes with fecal enemas (Eiseman et al.,1958).
Use of FMT for *Clostridium Difficile* Infection (CDI) successfully by retention enema was first reported in 1983 (Schwan et al., 1983).
Until 1989, retention enema was the most common technique for FMT. Since the 1990s, techniques have evolved, and include infusion via nasogastric tube, upper endoscopic and colonoscopic techniques, along with self-administered enemas. FMT is now emerging as a safe and effective treatment for the management of recurrent, and possibly refractory, CDI by restoring gut microbial diversity, with cure rates greater than 85% (Kassam Z., et al. 2013).
FMT has been approved as an endoscopic treatment for patients with recurrent *Clostridium difficile* diarrhea, for which it has had unanimously excellent results (Cammarota et al., 2015; van Nood et al. 2013); in Italy FMT is considered a transplantation of human substance and the Superior Health Council (Consiglio Superiore di Sanità) has decided to regulate it with the quality and safety requirements of transplantation of cells and tissues (see "The Human Microbiota: from research for clinical applications, recommendations and guidelines "- June 2018). Policlinico A. Gemelli in Rome performs this treatment.
Fecal microbiota transplantation is a potential emerging therapeutic option for diseases implicated in altered microbial compositions. Since a perturbed microbiota is associated with several diseases including inflammatory bowel disease, irritable bowel syndrome, nonalcoholic fatty liver disease, obesity, neuropsychiatric illnesses among others, it is conceivable that microbiota restoration therapies could be useful in their management (Khanna 2018).
Several studies are now testing FMT potentiality to treat neurological disorders such as multiple sclerosis, myasthenia gravis, Parkinon's Disease, epilepsy (Borody and Khoruts, 2011; Tremlett et al., 2017).

Figure 3: Fecal microbiota transplantation

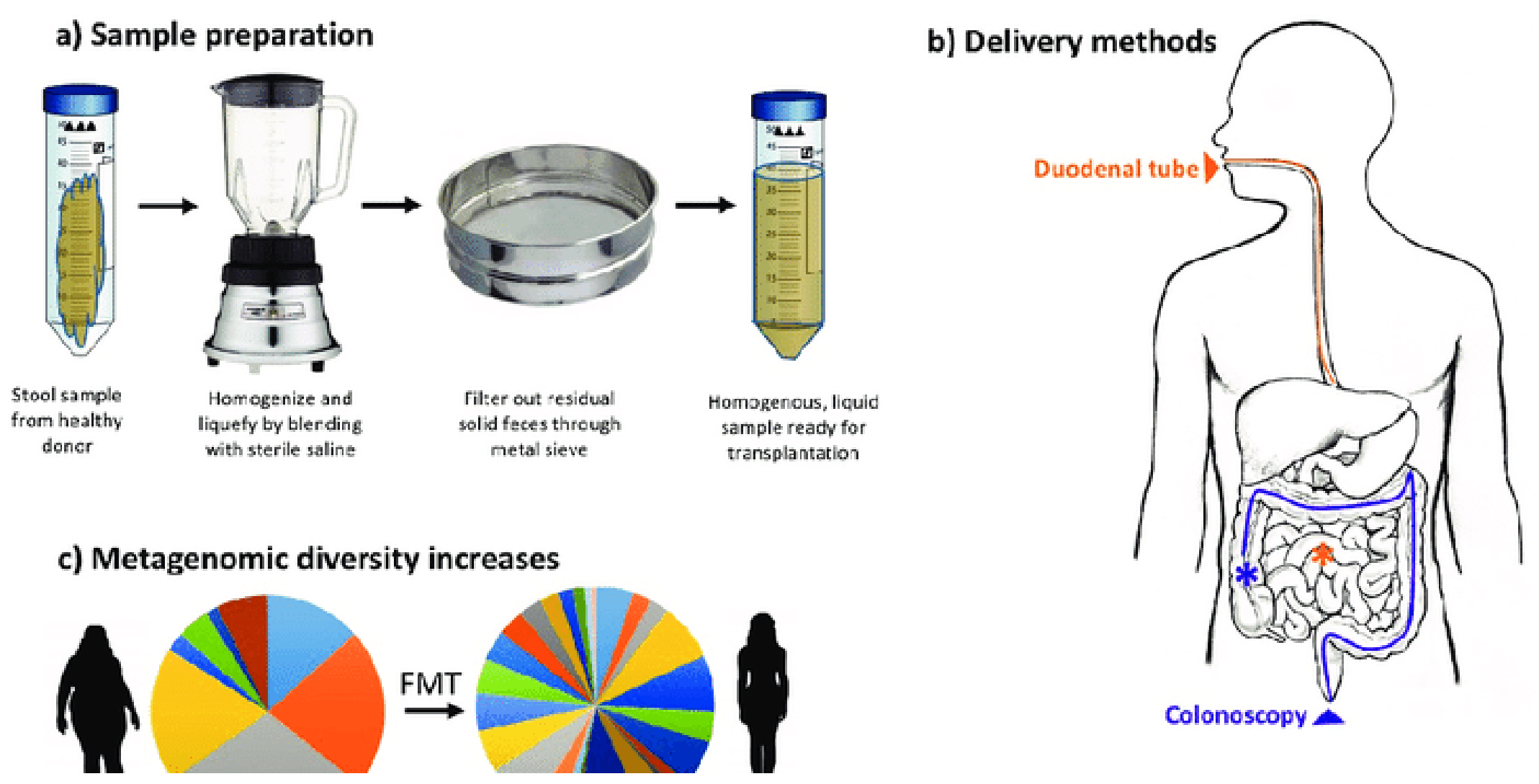

4. PRELIMINARY DATA

Some preliminary data place the theoretical basis for a GM involvement in ALS. Summing up, disturbances to the GM complexity can affect immune regulation and contribute to the development of inflammatory and autoimmune diseases (Frank et al., 2007). In ALS there is simultaneous activation of distinct lymphocyte populations, Th1 and Th17, and a decrease of T regulatory cells (Tregs) (Saresella et al., 2013) that have a protective role as demonstrated in both mice with ALS and in patients with ALS: a greater number of Tregs is associated with slow disease progression (Beers et al., 2011; Henkel et al., 2013) Moreover, the GM constitutively controls maturation and function of microglia within the CNS (Erny et al., 2015) and there are evidences for microglia involvement in ALS (Boillée et al., 2006), whose pathology is characterized not only by MN death, but also by infiltrating lymphocytes, and early microglia activation (Boillée et al., 2006b). Among T Lymphocytes (TL) Tregs suppress TL proliferation, downregulate pro-inflammatory cytokine production, and directly inhibit IL2 mRNA transcription. Tregs secrete anti-inflammatory cytokines (IL4, IL10, IL13) and neurotrophic factors, transform a Th1 to a Th2 response and attenuate toxic microglial responses; Tregs have been shown to directly differentiate macrophages from M1 to M2 state (Tiemessen et al., 2007). In cellular cultures mSOD1 Tregs co-cultured with activated mSOD1 microglia attenuated the expression of microglial toxic factors by IL4 release, and promoted MN survival by suppressing M1 activation, inducing an M2 protective phenotype, and reducing the ROS release (Zhao,2006). In SOD1 mice increased Tregs and M2 microglia were associated with the stable disease phase, whereas Th1L and M1 microglia predominate during the rapidly progressing phase, suggesting a shift from protection to toxicity.

The passive transfer of mSOD1 Tregs into ALS mice lacking functional TL prolonged survival; mRNA of FoxP3 in the spinal cord of mSOD1 mice inversely correlated with disease progression (Beers et al., 2011). In ALS patients Tregs % in the blood inversely correlated with ALS progression rate (Beers et al., 2011). Tregs' numbers and FoxP3 expression decreased with faster ALS progression; leukocytes from rapidly progressing patients (pts) expressed low mRNA levels of Gata3 (a Th2 transcription factor), IL4 e TGFbeta. FoxP3 levels were early predictors of ALS progression and survival (Henkel et al., 2013). In post-mortem studies reduced Foxp3 mRNA levels were found in rapidly progressing patients vs controls, and Gata3 mRNA increased in slowly progressive patients vs controls. Enhanced infiltration of Tregs and Th2L into the CNS of slowly progressing pts suggest that Tregs and Th2L could suppress inflammation in the spinal cord and influence the rate of ALS progression (Henkel et al., 2013)

All these evidences clearly show the role of immune system in ALS, that could be modulated through gut-brain axis. The gut environment favors the generation of autoreactive T-cells with unique regulatory functions, important for preventing CNS autoimmunity (Kudowaki et al., 2016). Commensal bacteria can induce Tregs development and FMT determine Tregs' increase (Round and Mazmanian, 2010).

The importance of healthy gut microbiome has been shown in three most recent articles published in Science, reporting that the patients with low levels of the bacterium Akkermansia muciniphila poorly responded to antitumor treatment (Gopalakrishnan et al., 2018; Matson et al., 2018; Routy et al., 2018). Moreover, a few papers have recently highlighted a potential link of gut dysbiosis and neurological diseases (Routy et al., 2018, Fang 2016; Scheperjans 2016; Rowin et al., 2017).

In fact, some animal data on alterations in the gut microbiome in neuroimmune disorders like multiple sclerosis (MS) have interesting implications for ALS. Investigating the role for the microbiota during the induction of experimental autoimmune encephalomyelitis (EAE), shows that

Via Giardini, 1355
Baggiovara
41126 Modena
tel. : 059396111
fax.: 0593962409

germ-free mice develop significantly attenuated EAE compared with conventionally colonized mice and produce lower levels of the proinflammatory cytokines IFN-γ and IL-17A in both the intestine and spinal cord with a reciprocal increase in $CD4^+CD25^+Foxp3^+$ Tregs. In germ-free animals, gut dendritic cells show a reduced ability to stimulate proinflammatory T cell responses. These findings reveal that the intestinal microbiota profoundly impacts the balance between pro- and anti-inflammatory immune responses during EAE, suggesting that the modulation of gut bacteria may provide therapeutic targets for extraintestinal inflammatory diseases (Lee et al., 2011). An increased prevalence of "leaky gut" was also recently suggested in a pilot study of 22 relapsing–remitting MS cases compared to controls. One small longitudinal study involving pediatric MS subjects found that some GM profiles were associated with future relapse risk (Tremlett et al., 2016).

Neurodegenerative diseases share accumulation of misfolded proteins inside the cells. Pathological changes in Parkinson's Disease (PD), involving a-synuclein deposition, can initiate in the nervous system of the gut and the olfactory bulb. Recent intriguing work in an a-synuclein overexpressing mouse model demonstrated that mice raised in a germ-free environment developed little PD-related pathophysiology (motor dysfunction, neuroinflammation, and a-synuclein pathology (Sampson et al., 2016), akin to that observed in the mouse MS' models. When germfree mice were either colonized with feces from wild-type mice or orally fed bacterial metabolites (short chain fatty acids) without colonization, this effect was reversed (Sampson et al., 2016). Colonization of these germ-free mice with feces from human subjects with PD (relative to feces from healthy donors) resulted in worse motor symptoms. Evidence for the gut as an initial site of PD pathology and the establishment of the gut–brain axis provided the framework for recent cross-sectional, moderately sized studies on the GM in PD. (Scheperjans et al., 2015).

Further, the gut microbiome is structurally altered and seems to increase cerebral amyloid-b pathology in mouse models of Alzheimer's disease (Brandscheid et al., 2016; Harach et al., 2017). Misfolded protein aggregation/oligomerization and neuroinflammation are thought to be major drivers also of motor neuron degeneration in ALS. Taken together, these data raise the hypothesis that these processes are influenced by the gut microbiota in ALS in a similar way.

As for ALS, Fang et al. (2016) found significantly increased population of harmful microorganisms (genus Dorea) and significantly reduced population of beneficial microorganisms (genus Oscillibacter, Anaerostipes, Lachnospiraceae) in ALS patients and suggested that the imbalance in intestinal microflora constitution (significant decrease in Firmicutes/Bacteroidetes ratio along with a reduced relative abundance of Oscillibacter, Anaerostipes and Lachnospiraceae) may cause a pro-inflammatory dysbiosis that could compromise the intestinal epithelial barrier and promote immune/inflammatory responses with consequent alterations and a role in ALS pathogenesis.

However, the authors did not evaluate whether, besides enteric bacteria, ALS patients were characterized by alterations of intestinal epithelial barrier and/or immune/inflammatory cells activation, data that will be evaluated in the present study.

In this context, two pre-clinical studies, carried out in G93A animals (a genetic model of ALS), have investigated a correlation between gut dysbiosis, altered intestinal permeability and enteric inflammatory/neurogenic responses. Wu et al., (2015) found signs of leaky intestine in a G93A transgenic mouse model with a defective intestinal tight junction structure and related protein expression, leading to increased gut permeability, compared to wild-type mice (Wu et al., 2015). Normal epithelial Paneth cells, which impact the gut microbiome and help tune the innate immune response, were also decreased in number and function, with functionality measured as levels of specific antimicrobial proteins secreted into the gut. Shifts in the gut microbiome included a lower relative abundance of butyrate-producing bacteria such as Butyrivibrio fibrisolvens, Escherichia

coli and Firmicutes, demonstrated by 16s rRNA real-time polymerase chain reaction (Wu et al., 2015). Likewise, Zhang et al. (2017) reported a correlation between gut dysbiosis and morphofunctional alterations of intestinal permeability in G93A animals, since the earliest stages of the disease. Thery demonstrated for the first time that the ALS-causing SOD1 mutation that leads to protein aggregate formation in neurons and skeletal muscle indeed forms aggregates also in the intestine of ALS mice and human intestinal epithelial cells. Moreover, they observed that, following treatment with 2% butyrate, a natural bacterial product, able to restore the intestinal microbial homeostasis G93A mice displayed a restored gut microbiota homeostasis and intestinal epithelial barrier integrity, as well as an improvement of central and peripheral symptoms of the disease, of survival and of weight loss (Zhang et al., 2017)

The leaking intestine might be at least partially responsible for the observed discordance between increased presymptomatic total daily energy intake and decreased presymptomatic body mass index in ALS patients (Huisman et al., 2015).

These data suggest that changes in GM, impaired intestinal permeability and enteric inflammation represent one of the earliest events in ALS, and that treatments can restore the intestinal homeostasis and counteract the progression of the disease.

However, these findings do not allow to firmly establish whether the alterations of the enteric bacterianeuro-immune network contribute to the pathophysiology of ALS, or whether they occur as a consequence of the central neurodegenerative processes.

A recent study (Mazzini et al., 2018) focused on microbiota analysis in ALS patients, and detected a clear cluster division between the bacterial profiles of ALS patients and the healthy subjects, mainly based on the presence/absence of bands in the profiles. For the yeast, the profiles was much more simpler with respect to that of Eubacteria and no unequivocal association with the presence or absence of disease could be evidenced. The authors also observed a lower DNA concentration in patients compared with controls, with a low abundance of Clostridium and yeast, and a high abundance of E. coli and Enterobacteria, confirming that microbiota is modified in ALS patients and it could contribute to the pathogenesis of the disease.

Therefore, further investigations should be implemented to better clarify the role of enteric bacteria-neuro-immune network in ALS patients, since the earlier stages of the disease, and microbiota-based treatment approaches can represent a new therapeutic target for ALS.

Individual case reports of PALS reported benefits as a result of FMT (Pecere et al., 2015). This research field is therefore highly innovative and is gathering increasing interest from the international scientific community (Scheperjans 2016; ALS Untangled Group 2013).

Finally, as nearly 30% of ALS patients show autonomic dysfunctions; the intermediolateral columns and the Onuf nucleus are involved in ALS. The vagal nerve could be a route for GM and brain communication (Piccione et al., 2015). Of note, gut microbiota has been found to interact with ENS-vagus nerve pathways (Furness 2012) because, bacterial derived-neurotransmitters and neuropeptides can activate directly myenteric neurons, which, through vagal nerve ascending fibers, deliver nerve inputs to the brain (Quigley 2017).

5. OBJECTIVES

5.1 PRIMARY OBJECTIVE

The primary objective is to assess whether FMT increases Tregs' number in ALS patients treated with FMT compared to the control arm and measured at baseline and at month 6.

5.2 SECONDARY OBJECTIVES

5.2.1 Biological assessment

We expect to find that FMT changes gut microbiota, and influences gut-brain axis, increasing the expression of Tregs, and modulating the inflammatory response of the disease.

Specific biological aims will be pursued comparing baseline and different time points, between FMT and control arms, and will include:

- ✓ Tregs cells and T cell subsets at different time points in blood and gut tissue samples: at baseline (both) and at month 3-6 (both)-9-12 (both) we will quantify Tregs by polychromatic flow cytometry (PFC), using specific mAbs
- ✓ CSF samples at baseline and at study end only after a proper given consent (lumbar puncture will not be mandatory) to study neurophylaments and CSF cytokines and cells.
- ✓ Analysis of fecal and saliva samples at baseline and at month 6 and 12 (to evaluate microbiota and cytokines' profile)

5.2.2 FMT safety and tolerability in a cohort of ALS patients

To assess safety and tolerability: at each neurological visit (screening, baseline, month 1-3-6-7-9-12) patients will be monitored with particular attention to possible side effects, including increased risk of infections, constipation, diarrhea, pain, nausea, headache, fever. Routine blood samples will be performed at each neurological examination including blood cell count, serum cholesterol and triglycerides, liver and renal function, urine examination, fecal calprotectin. Death from any cause or tracheotomy will be also considered.

5.2.3 Clinical assessment

- ✓ Overall survival from randomization to date of documented death or tracheostomy
- ✓ Forced vital capacity score from baseline to month 3,6,9,12
- ✓ ALSFRS-R score from baseline to month 3,6,9,12
- ✓ Frequency of procedures (PEG, NIV, IV) from baseline to month 3,6,9,12

5.2.4 Quality of Life assessment

ALSAQ-40 from baseline to month 6 and 12

6. INVESTIGATIONAL PLAN

6.1 STUDY DESIGN

We are going to perform a randomized double blind multicentre study on FMT in ALS.
Patients will undergo screening procedures that must be completed during the 15 days screening time; then they will be randomly allocated to either FMT or control group. Randomization will be 2:1 (FMT: controls). Given the heterogeneous ALS progression, patients will be stratified by ΔFS (progression rate, calculated at randomization according to Kimura et al.2006 (Kimura et al., 2006).
Riluzole, an anti-glutamatergic agent that inhibits the presynaptic release of glutamate, is the only drug for the treatment of all ALS patients approved by EMA and will be maintained during the entire study duration unless adverse events or patients' decision to withdraw.
Fecal microbiota transplantation will be performed at baseline and then after 6 months. After treatment end patients will be followed up for further 6 months. This FU time has the aim to assess potential late adverse events (AEs) and to assess later biological or clinical effects of transplantation.

Figure 4: Study design

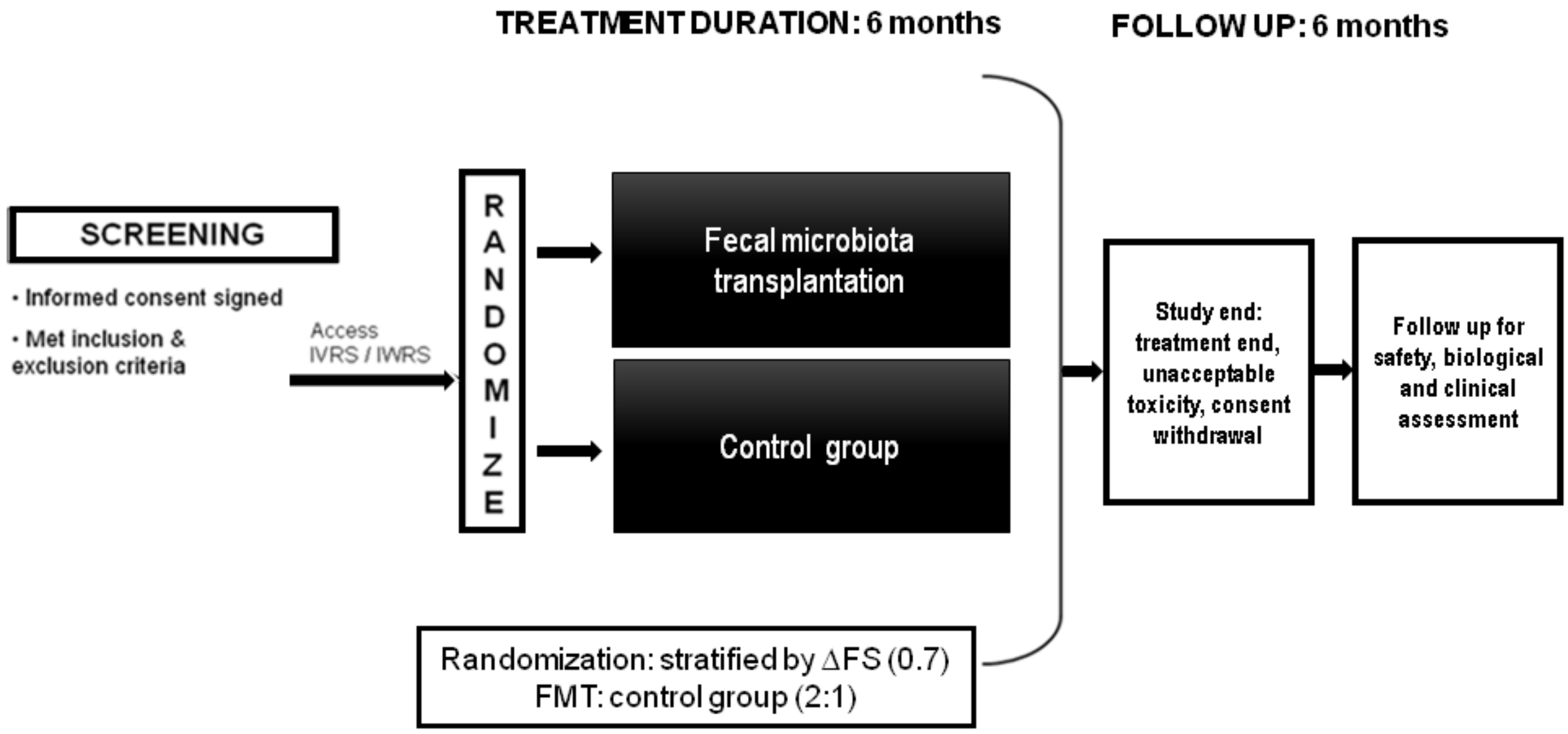

6.2 STUDY FLOW CHART

Each patient will be treated at baseline and at month 6, with a follow up period of 6 months. Patients must be followed at the study centre according to the flow charts.

Via Giardini, 1355
Baggiovara
41126 Modena
tel. : 059396111
fax.: 0593962409

Table 1: Study flow chart

	Pre-treatment		Treatment					Follow up			Study end
**Examinations**	**Screening (VS)**	**Baseline (W0)**	**FMT 1 (Rome)**	**M1**	**M3**	**M6**	**FMT 2 (Rome)**	**M7**	**M9**	**M12 (Rome)**	**M12**
**Time window**		<3 weeks from screening	<4 weeks from screening	± 3 day	± 3 day	± 3 day	± 7 days	± 7 days	± 7 days	± 7 days	≥7 days <4 weeks from M12
Informed Consent	x										
Medical History	x										
Inclusion exclusion criteria	x										
Patient able to understand and follow procedures	x										
FMT			x				X				
**Clinical assessment**											
Neurological examination	x	x		x	x	x		x	X		x
ALSFRS-R	x	x		x	x	x		x	X		x
FVC	x	x		x	x	x		x	X		x
MRC	x	x		x	x	x		x	X		x
BMI	x	x	x	x	x	x	x	x	X	x	x
**Safety assessment**											
Adverse events		x		x	x	x		x	X		x
Vital signs	x	x	x	x	x	x	X	x	X	x	x
Physical examination	x	x	x	x	x	x	X	x	X	x	x
Concomitant medications	x	x	x	x	x	x	X	x	X	x	x
Chest X-ray	x (1)										
ECG	x (1)										
Hematology	x	x		x	x	x			X		x
Biochemistry	x	x		x	x	x			X		x
Urinalysis	x	x		x	x	x			X		x
Pregnancy test	x										
Infectious markers	x										
Fecal calprotectin			x				X			x	
**Biological activity**											
T-regs		x	x		x		X		X	x	x
Lymphocytes phenotype		x	x				X			x	
Fecal and saliva samples(microbiota)			x				X			x	
Gut tissue			x				X			x	
CSF		x				x					
Peripheral biomarkers		x			x	x			X		x
**Quality of life assessment**											
ALSAQ40		x				x					x

(1) If not done at diagnosis or in the last 12 months.

Table 2: Blood test: time and location

	**Pre-treatment**		**Treatment**					**Follow up**			**Study end**
**Examinations**	**Screening (VS)**	**Baseline (W0)**	**FMT 1 (Rome)**	**M1**	**M3**	**M6**	**FMT 2 (Rome)**	**M7**	**M9**	**M12 (Rome)**	**M12**
**Time window**		**<3 weeks from screening**	**<4 weeks from screening**	**± 3 day**	**± 3 day**	**± 3 day**	**± 7 days**	**± 7 days**	**± 7 days**	**± 7 days**	**≥7 days <4 weeks from M12**
**Routine laboratory tests**											
Cell count with formula (1)	x	x		x	x	x			x		x
Creatinine	x	x		x	x	x			x		x
Albumin		x			x	x			x		x
Protein electrophoresis	x				x	x			x		x
AST	x	x		x	x	x			x		x
ALT	x	x		x	x	x			x		x
Gamma GT	x	x		x	x	x			x		x
Amylase	x	x		x	x	x			x		x
Bilirubin	x	x		x	x	x			x		x
Glucose	x	x		x	x	x			x		x
PCR	x	x		x	x	x			x		x
Sodium	x	x		x	x	x			x		x
Potassium	x	x		x	x	x			x		x
Urinanalysis	x	x		x	x	x			x		x
CK		x			x	x			x		x
Total Cholesterol		x			x	x			x		x
LDL Cholesterol		x			x	x			x		x
Triglycerides		x			x	x			x		x
Urea		x			x	x			x		x
Pregnancy test	x										
Hepatis B, Hepatitis C	x										
Fecal calprotectin			x				x			x	
**CSF tests**											
**CSF cells**		FI				FI					
**pNfH**		MO				MO					
**Cytokines**		FI				FI					
**Biological activity**											
T-regs		FI	FI		FI	FI	FI		FI	FI	FI
Lymphocytes phenotype		FI	FI		FI	FI	FI			FI	
Gut tissue			FI, RO				FI, RO			FI, RO	
Fecal and saliva samples			FI, RO				FI, RO			FI, RO	

(microbiota)											
**Peripheral biomarkers**											
Vitamin D		x			x	x	x		x		x

FI = Laboratory of Immunology, Florence University
RO = Laboatory of microbiology, Policlinico Gemelli, Rome
MO= Laboratory of Neuroimmunology, AOU Modena

6.3 WORK PLAN

This multicentre study involves 9 Italian Units: 6 referral ALS Centres, 3 renown laboratories/endoscopy service and 1 statistical unit.

Clinical ALS Centres:

1. Dr. Jessica Mandrioli, ALS Center, S. Agostino-Estense Hospital, Azienda Ospedaliero Universitaria di Modena (Coordinating Centre)
2. Prof Vincenzo di Lazzaro, Institute of Neurology, Policlinico Campus Bio-medico, Rome
3. Dr. Mario Sabatelli, Dr.ssa Amelia Conte, NEuroMuscular Omnicentre Centre (NeMO), Fondazione Serena Onlus-Fondazione Policlinico A. Gemelli, ROME; Institute of Neurology, Catholic University of the Sacred Heart, Fondazione Policlinico A. Gemelli, Rome, Italy Rome, Catholic University, Rome
4. Dr. Sabrina Matà, Prof. Sandro Sorbi, Azienda Ospedaliero Universitaria Careggi, Florence
5. Dr. Teresa Anna Cantisani, Dr. Paola Brunori, UO Neurofisiopatologia, Azienda Ospedaliera dì Perugia
6. Dr. Margherita Capasso, Prof. Marco Onofrj, Clinica Neurologica, Ospedale Clinicizzato "SS Annunziata", Chieti
7. Prof. Fabio Giannini, Dipartimento di Scienze Mediche, Chirurgiche e Neurologiche, Università di Siena, Ospedale “le scotte” , Siena

Statistical Unit:

8. Prof. Roberto D’Amico, Department of Diagnostic, Clinical and Public Health Medicine, University of Modena and Reggio Emilia

Laboratories and endoscopy:

9. Dr. Elena Niccolai, Prof. Amedeo Amedei, Department of Experimental and Clinical Medicine, University of Florence, Florence
10. Prof. Teresa Spanu and Prof. Luca Masucci, Department of Microbiology – Policlinico Universitario "A. Gemelli", Rome
11. Prof. Giovanni Cammarota, Department of Internal Medicine and Specialistic Sciences – Policlinico Universitario "A. Gemelli", Rome

ALS is a rare disease with fast progression and no effective treatment, which requires a solid methodological, clinical approach and biological background for clinical trials conduction. FMT is a new endoscopic treatment that can be performed only at Policlinico Gemelli in Rome.
Clinical and laboratory centers have an already established collaboration in the field of ALS, in particular on the study of microbiome, they share experience in clinical management of patients, in the realization of clinical trials and research through the ITALSGEN and SLAGEN Genetics Consortia.

Via Giardini, 1355
Baggiovara
41126 Modena
tel. : 059396111
fax.: 0593962409

Dr. Mandrioli (ALS Centre, Modena) is the chief a regional ALS reference centre for Emilia Romagna Region (ERR) and follows currently more than 200 patients. The centre is involved in clinical (clinical trials, deep phenotyping) and basic research in collaboration with the ITALSGEN Consortium, which contributed to the discovery of new ALS genes (VCP, C9orf72, Matrin3). She was the coordinator of the Italian MND Study Group (2013-2017). She created and coordinates the population-based registry of ALS in ERR (4.4 million pop). She is coordinating other Italian RCTs on ALS (RAP-ALS and co-ALS).
Dr. Sabatelli and Dr. Conte work at ALS care and research of the Italian Centre for Neuromuscular diseases in Rome, a nationwide referral Centre for ALS management and research. They focused their studies on genetics and created a biobank for cellular cultures in ALS.
Dr. Matà and Prof. Sorbi work at Florence Centre for Neuromuscular Diseases, one of the referral Centre for ALS care and researche in Tuscany. They focused their studies on immunology and genetic of neurologic disorders. The center is leading a study on metabolomic and one on epidemiology and end-stage decisions of ALS patients.
Prof. Vincenzo Di Lazzaro is Professor of Neurology, Medical Director for Clinical Neurology and serves as Director for the Neurology Residency Program and Research Coordinator of Campus Bio-Medico Medical School. He is President-elect of Italian Society of Clinical Neurophysiology. His clinical focus is the care of patients with neurodegenerative and neuromuscular disorders, with stroke, and with spinal cord diseases. The main areas of research are the study of the physiological bases of recovery in stroke and the development of methods of neuromodulation (both invasive and non-invasive) as potential treatment tools for several neurological diseases, movement disorders and stroke in particular. The studies on the invasive deep brain stimulation are highly cited and have a significant impact on the field of deep brain stimulation. Another relevant area is the use of neurophysiological techniques for the diagnosis of neurological disorders (dementia in particular), for the evaluation of the effects of drugs on the intact human brain and for the study of human brain plasticity. Professor Di Lazzaro participated in several clinical studies (including Crystal study), most of them Phase III studies and all requiring GCP certification. Vincenzo Di Lazzaro has authored more than 300 peer-reviewed publications, 8 book chapters, with about 21.000 citations and an h-index of 66.
Dr. Capasso (ALS Centre, Chieti) is the chief of a regional ALS reference centre for Abruzzo Region and follows currently more than 100 patients. The centre is involved in clinical trials and basic research in collaboration with the ITALSGEN Consortium.
Dr. Cantisani (Perugia Hospital) is Trials' Investigator and Chief of Neurophysiopathology Unit; Dr. Brunori (ALS Centre, Perugia) is the chief of the Regional ALS Reference Centre for Umbria and with her multidisciplinary équipe follows currently more than 85 patients.
Prof. Giannini (ALS Center, Siena)) is the chief of a Regional ALS Reference Centre for Toscana Region, has great expertise in ALS genetics and clinical trials, and with his multidisciplinary équipe follows currently more than 100 patients.
The analysis of biomarkers will be centralized and performed in 2 internationally renowned laboratories for the use of advance techniques for the study of GM.

Dr. Niccolai is a PhD that has focused her studies on immune response in cancer patients. Actually her current topic is the evaluation of the relation between gut microbiota and intestinal inflammatory respons in colorectal cancer. Now she is a PhD fellowship holder for the determination of cytokines and characterization of T cell subsets in biological samples obtained

from patients with CRC at the Department of Experimental and Clinical Medicine, University of Florence.
Prof. Amedei is Associate Professor at the Department of Experimental and Clinical Medicine, University of Florence. In the last years, the Prof. Amedei has focused his scientific interests on the cancer, studying, in detail, the anti-cancer immune response and the role of cancer microenvironment. He is member of the International cancer project "HALIFAX" and Inventor of Patent Amedei A. and al: Use of the Neutrophil activating protein of Helicobacter pylori (HP-NAP) and/or of its portions as adjuvants for the development of T helper type 1 (Th1) immune responses. Publication Number: WO/2007/039451. His current scientific topic is the evaluation of the correlation between the microbiome and the immune response in the genesis of diseases inflammatory-correlated; in detail colorectal cancer, celiac disease, Inflammatory Bowel Diseases, Amyotrophic Lateral Sclerosis and Becet Disease.
Prof. Spanu (2nd Division Analysis (Virology and Microbiology), Department of Diagnostic and Laboratory Medicine, Faculty of Medicine " Agostino Gemelli " Catholic University of the Sacred Heart, Rome) research is focused on the study of microbiology, on the resistance to antibacterial drugs, and on epidemiology of resistant organisms, on the assessment of the clinical course of infections, and on the development of rapid diagnostic strategies of systemic infections. Her colleague, Prof Masucci has been working as medical doctor in charge for parasitology and gastric-enteric infections, for microbiology procedures of intestinal microbiota solution and screening of donors for Fecal Microbiota Transplantation (FMT) and for surgery at the 2nd Division Analysis (Virology and Microbiology) relating to the Department of Diagnostic and Laboratory Medicine - (UCSC) Fondazione Policlinico Universitario "A. Gemelli". His research interests are focused on development of molecular methods for clinical diagnosis in microbiology.
Prof. Masucci is a member of the team of researchers involved in two European projects: "ClosER" a Pan-European Longitudinal Surveillance Study to Monitor antibiotic resistance and Prevalent Clostridium difficile Ribotypes and "EuCLID" – European, multi-centre, prospective bi-annual point prevalence study of *C. difficile* Infection in hospitalised patients with diarrhoea. Recently he focused his research activity on the characterization of microbiota in human gastrointestinal pathologies and on FMT in *C. difficile* and Inflammatory Bowel Diseases: 280 infusions have been performed.
The team of the Institute of Microbiology is regarded as a leading group in the international microbiology field. The Laboratory of Microbiology (1,100 mq) is fully equipped to perform all microbiological procedures. BSL-1, BSL-2 and a BSL- 3 room and facilities are available. The Laboratory of Microbiology perform>650,000 analyses/year. Laminar flow biohazard cabinets (Steryl VBH), centrifuges and ultracentrifuges (Beckman Coutier 22R), BSL-3 animal facility, MALDI-TOFMSmass spectrometry apparatus (Bruker Daltonics), Affymetrix Microarray Facility, illumina® Sequencing System. Quality management system is certified by Det Norske Veritas to conform to quality management standard UNI EN ISO 9001:2018.

Prof. Cammarota, is Associate Professor of Internal Medicine, Internal Medicine, Gastroenterology and Liver Unit, at Agostino Gemelli University Hospital, Catholic University of Rome, Italy. He is responsible for the Simple Operative Unit of "Digestive Endoscopy, Breath Test and Gastroenterological Diagnostics". He has developed research on the involvement of Helicobacter pylori infection in the natural history of gastroduodenal pathologies. He also investigated the host-environment interaction in the gastro-enteric tract, especially in patients with autoimmune diseases, or those suffering from celiac disease. He has conducted studies on the pathophysiology, diagnosis

Via Giardini, 1355
Baggiovara
41126 Modena
tel. : 059396111
fax.: 0593962409

and clinical of celiac disease, especially in relation to the associated extraintestinal diseases. A particular interest has turned to the study of the relationships of the chronic infection with the hepatitis C virus and the pathogenesis of the gastric lymphoma. His recent studies on the pathophysiology of gastro-oesophageal reflux disease have helped to highlight the relationship with laryngeal carcinogeesi. Other contributions have been made on the use of new instruments with high resolution imaging in the diagnosis of diseases of the gastro-intestinal tract. Recently he focused his research on gut microbiota and on fecal transplantation contributing to many papers among which consensus conferences, reviews and guidelines on this very up-to-date topic.

Statistics Unit: Prof D'Amico is a statistician with great experience on RCT project and management, and on data analysis. He is the Director Cochrane Italy, and he has been involved in planning the study design and will be in charge of data management and analysis.

According to different roles of partners in the study, the work plan will be divided into 4 WPs: WP1 (study preparation); WP2 (RCT conduction); WP3 (laboratory studies), WP4 (data extraction, statistical analyses and result presentation).

6.3.1 WP1: Management and Project Coordination

Unit 1 and Unit 7
WP1 has been designed to manage and coordinate the proposed research project, through monitoring activities of all Centres, facilitating communication, promoting exchange of ideas and methodological approach, stimulating the analysis and the integration of results.

Kick off meeting and regular meetings with partners
The PI will organize the kick off meeting, regular meetings with partners, and on time correspondence of the trial activities: a first meeting will be organized to share the study protocol and rules, to discuss methodology and possible pitfalls of the study. Participating Centers will discuss and contribute to finalize the protocol, and will participate to investigator meetings.
Regular meetings will be organized, also using videoconferences to limit expenses for travels, and also to ensure the maximum participation of all partners at meetings.
During regular meetings exchange of ideas and methodological approach will be ensured as well as problem discussion and solving, and sharing of the work in progress.
The final meetings will be devoted to analysis and discussion of results.

Ethical Committee requirements
Submission to Ethical Committees of the Participating Centres is expected within month 2. An independent Contract Research Organization (CRO) will be selected for study monitoring. A Trial Manager will oversee the conduct of the study alongside the PI from the beginning of the study.

CRFs creation
The Statistics Unit of the University of Modena (Prof. D'Amico, Unit 7), together with the coordinating Centre will create, the eCRF based on the format of recently used eCRF for other RCT. The eCRF will be released and tested by the coordinating Centre, to be therefore provided to each participating centre before the beginning of the study.

Establishment of an independent Data and Safety Monitoring Board (DSMB)

An independent DSMB will be established, to address safety and efficacy concerns that could arise during the study. Reports including all relevant clinical data of patients will be sent by the Statistics Unit of the University of Modena to an independent DSMB for scheduled DSMB meetings (also through Skype) when 14 patients have done the second treatment and subsequently after every 6 months.

Establishment of an Independent Ethics Advisory Board (IEAB)

An independent DSMB will be established, to address ethics concerns that could arise during the study, with particular attention to informed consent form. The informed consent form (ICF) will be obtained by a neurologist not involved in the care relationship with the patient. In the patient information sheet that will be given to the patient at least two weeks before ICF collection and screening, the patient could find a telephone number of the IEAB, that he/she could call in the event of need of further information from an independent person. On the same day when the neurologist will give the patient information sheet, the investigator will advise the IEAB members of a potential enrollment to allow IEAB members to participate to ICF collection. The IEAB will send to the Ethics Committee a report within 2 months from study actual starting date and another report at the end of the study. Standard Operating procedures for DSMB and IEAB have been set up.

6.3.2 WP2: RCT conduction

Units 1, 2, 3, 4, 5,6, 10

This study is a randomized, double-blind, controlled, multicenter, clinical trial (RCT) to test FMT in ALS patients. The study will include 42 ALS patients (EL Escorial Revised Criteria, sporadic and familial) with 2:1 allocation in 2 groups (FMT 28 patients: 14 controls); treatment will be double blinded to patients and caring physicians and will be done at baseline and at month 6. Patients will undergo upper GI endoscopy with small-intestine biopsies, blood, feces and CSF (optional) collection. Active treatment: allogenic infusion of collected feces from donors (FMT) in the duodenum-jejunum; feces will be obtained by abitual healthy donors for *C. difficile* infection (see section 6.3.3 for feces preparation). The infusion will be performed through a standard nasojejunal tube, that will be placed during endoscopy. Infusion of feces will be repeated at 6 months. The same donor will give stool sample both for the first (baseline) and the second (month 6) infusion. Upper GI endoscopy 12 months after FMT will be performed only to identify specific microbioma and mucosal immunological evaluation. Salivary, Fecal samples and small intestine biopsy samples will be obtained from patients to perform the gut microbiota typing also at baseline, and 6 and 12months. Control group patients will not undergo any sham procedure, but will undergo to intestinal biopsy. Treatment will be blinded to patients and to caring neurologists. Computerized randomization will be stratified by ΔFS (</≥0.7). Randomization time: 18 months. Treatment: 6 months. Post-treatment follow up: 6 months. Overall study duration: 36 months.

The PI together with clinical partners will enroll patients; they will send patients to Policlinico Gemelli in Rome for the two treatment sections (baseline and month 6) and will follow up patients with 3 months-visits. They will be responsible for patients follow up and AE recording. They will follow patients with a multidisciplinary approach; they will record data on disease progression, procedure, side effects. They will manage with great attention every possible side effect due to the treatment. The partners will organize sample collection and shipping together with Partners in

Via Giardini, 1355
Baggiovara
41126 Modena
tel. : 059396111
fax.: 0593962409

charge for biological activity studies who will be responsible for management of all samples and biological assessments.

The statistic Unit will be in charge of computerized randomization, will contribute to trial coordination and to send data reports to an independent DSMB after the 14th subject have completed the second treatment and subsequently after every 6 months.

The following aims will be pursued:

a) **The primary aim is to assess whether FMT increases Tregs number in blood/gut tissue of ALS patients compared to the control arm and measured at baseline and at month 6 (treatment end).** To assess immunological changes induced by FMT in ALS patients blood collection will be performed before starting treatment, then at month 1-3-6-9-12. Fresh blood samples will be sent to University of Florence Laboratory of Immunology and examined within 24 hours. The "immunological response" is defined as the proportion of patients exhibiting a Tregs number (%) increase of at least 20% comparing baseline and treatment end (second FMT) between FMT and control arm. Tregs will be defined as $CD4^{+}CD25^{+}CD127^{-}FoxP3^{+}$ T cells.

   Other biological assessments: other immunological changes to be examined will be changes in the distribution of the T cells' subsets (in blood and gut tissue), changes in inflammatory/immune cytokines (serum, fecal and salivary) comparing baseline and treatment end between FMT and control arm.

   Blood withdrawal will be performed from a venous site in the upper limbs when possible. Patients will undergo blood withdrawal for biological activity assessment at baseline and at month 1-3-6-9-12.

   Fecal and saliva microbiota will be studied **at** baseline and at month 6 and 12 to evaluate the difference between treated and untreated patients for oral and intestinal microbiota.

   CSF will be obtained through lumbar puncture at baseline and month 6. Lumbar puncture will be optional for patients and we will assess CSF cytokines, NFL, and other biomarkers of disease progression

   This complex biological assessment including immunological cells in different sites (CSF, blood, gut) and over different time points is necessary due to the absence of established reference values of Treg in subjects with ALS, and the current lack of knowledge of the kind and extent of interaction between inflammation and immunity at the intestinal, systemic and central nervous system levels in ALS patients. Intestinal biopsies will allow to evaluate the immune cells (especially the Tregs) at the level of the intestinal mucosa, as well as blood sample will allow to assess Treg cells at the systemic level; the execution of lumbar puncture will allow to assess cytokines network in the CNS as well as at intestinal and systemic level.

   Moreover, currently it is not clear when a supposed immunological change induced by fecal transplantation may occur in patients with ALS or if the disease itself and its progression are associated, even in the absence of treatments, to immunological changes. For this reason the study includes endoscopic procedures with biopsy at three time points (0, 6, 12 months) both in treated patients and in the control group, as they will show possible "late" effects of the treatment or vice versa immunological changes linked to the disease in the most advanced phase. The comparison between treated and untreated patients and, for treated patients, the

Via Giardini, 1355
Baggiovara
41126 Modena
tel. : 059396111
fax.: 0593962409

comparison before and after FMT, will allow to have comprehensive information about the immune system and gut-brain axis in both treated and untreated patients.

To limit the number of invasive procedures, mucosal biopsies will be performed on small intestine, the site of FMT, considering that FMT in the distal colon may make difficult for some patients to retain the donor material and it may require multiple treatments (Kelly et al., 2015).

Less invasive methods, such as nasointestinal infusion may be safer in patients who are frail at the time of FMT. Although most of the mucosal immune system is concentrated in the colon, in every tract of the digestive system there is an immune response that can be modulated by the microenvironment i.e. at gastric level (Amedei et al., 2003), pancreatic (Amedei et al., 2013), and in the duodenum (namely the site we choose), this last site with an important role of the Tregs (Graves et al., 2017). Even though most of the microbial species are concentrated in the colon, starting from the mouth each tract of the digestive tract is characterized by its own microflora, more or less abundant depending on the district, with a sharing of more or less marked species, depending also on the pathology, as recently shown in the irritable bowel syndrome (Li et al., 2018)

Finally, lumbar puncture and CSF neurofilaments (that are among the most promising disease progression biomarkers) will also allow to have further markers of the biological response to the treatment. Neurofilaments are indeed markers of neurodegeneration and their assessment together with the study of the immunological component at various levels will allow to evaluate also the effect of a possible immunological change on motorneurons degeneration.

b) FMT safety and tolerability in a cohort of ALS patients

All patients will be accurately screened for any possible contraindication to FMT through a validated questionnaire for FMT. At every scheduled visit patients will be clinically monitored with particular attention to all possible side effects (total and severe adverse events) including the most common represented by diarrhea, abdominal cramping and pain, nausea, constipation.

The existing studies suggest that FMT is a safe therapy, with few serious adverse events reported. Adverse events attributed to FMT are uncommon. There have been individual reports of peripheral neuropathy, Sjogren syndrome, idiopathic thrombocytopenic purpura, microscopic colitis, contact dermatitis, rheumatoid arthritis, obesity, bacteremia, and ulcerative colitis flare after FMT. In the elderly, adverse events have included diarrhea, constipation. In one meta-analysis, the overall incidence rate of adverse events after FMT was 28.5%. The commonest FMT-attributable adverse event was abdominal discomfort. (Brand et al., 2012; Kelly et al. 2014; Baxter and Colville, 2016; Wang et al., 2016)

All AEs will be monitored with attention. Analysis will be performed considering treatment period and observational period for early and late AE. To monitor early and late AE clinical examinations and routine laboratory work will be performed during treatment and follow up to avoid any kind of toxicity. In particular, patients will undergo blood withdrawal at screening, baseline, week 4 and month 3-6-9-12 for safety purposes.

AE reporting will be performed according to Italian Regulation (D.Lgs. 191/2007, D.Lgs 16/2010).

Tracheostomy free survival in patients treated with active treatment and controls will also be assessed at study end. An independent DSMB will be established to address safety or other concerns that may arise during the study, with frequent meetings.

Via Giardini, 1355
Baggiovara
41126 Modena
tel. : 059396111
fax.: 0593962409

c) **Clinical assessments** will include overall survival from randomization to date of documented death or tracheostomy. The ALSFRS-R will be measured by neurologists experienced in ALS treatment at every visit. The ALSFRS-R is widespread used to assess disease progression in clinical practice and also in clinical trials for ALS as primary or secondary outcome measure. It is a validated instrument for assessment in the domains of gross and fine motor function, bulbar symptoms and breathing ability in patients with ALS. The scale does not rely upon physical examinations or instruments, measures function from the patient's perspective, shows a high inter-rater and intra-rater reliability, has excellent internal consistency and test–retest reliability, and can be reliably administered over the telephone. ALSFRS-R correlates with measures of muscle strength and survival (Kauffmann et al., 2007). The European Medical Agency (EMA) Guideline on clinical investigation of medicinal products for the treatment of ALS, requires either survival time or ALSFRS-R to be used as primary outcome measures to pursue the objectives of increasing disease survival or delaying ALS progression (http://www.ema.europa.eu/docs/en_GB/document_library/Scientific_guideline/2015/12/WC500199241.pdf).
   Forced vital capacity (FVC) score from baseline to month 3,6,9,12 and Quality of Life assessment through ALSAQ-40 scale.

6.3.3 WP3: Preparation of fecal material

Unit 9
Active treatment under study is represented by allogenic infusion of collected feces from donors (FMT) in the duodenum-jejunum; feces will be obtained by abitual healthy donors for C. difficile infection (see section 7.4 for inclusion and exclusion criteria for donors).
The same donor will give stool sample both for the first (baseline) and the second (month 6) FMT.
Before preparation, sample donation will be analyzed by rapid molecular test to detect intestinal pathogens.
The following rules will be applied for fresh fecal material preparation:
- ✓ Fresh stool will be used within 6 hours after defecation
- ✓ To protect anaerobic bacteria, the storage and preparation will be as brief as possible
- ✓ Until further processing, the stool sample can be stored at ambient temperature (20°C–30°C)
- ✓ The processing of the fresh stool should be performed under a hood.
- ✓ Anaerobic storage and processing will be applied
- ✓ An amount of 60 g of faeces will be used.
- ✓ Faecal material will be suspended in 200 mL NaCl 0.9% solution using a blender or manual effort and sieved in order to avoid the clogging of infusion syringes and tubes
- ✓ A dedicated space, disinfected using measures effective against sporulating bacteria, will be used
- ✓ Protective gloves and facial masks will be used during preparation
- ✓ Materials used for preparation and bags with final product have to be sterile.
- ✓ The bag containing the final product must be labelled with the following information: donor identification, date of preparation, time limit for administration, storage temperature.
- ✓ 5 g of donated material should be stored (-80° C) for possible future controls as well as a sample of final product.

Once fresh fecal material will be prepared, on the same day the infusion will be performed through a standard nasojejunal tube.

Via Giardini, 1355
Baggiovara
41126 Modena
tel. : 059396111
fax.: 0593962409

The following rules will be applied for frozen fecal material preparation:

- ✓ To protect anaerobic bacteria, the storage and preparation will be as brief as possible
- ✓ The processing of the stool should be performed under a hood.
- ✓ An amount of 60 g of faeces will be used.
- ✓ Faecal material will be suspended in 150 mL glycerol 10% solution
- ✓ Until further processing, the stool sample will be stored at -80°C
- ✓ Anaerobic storage and processing will be applied
- ✓ A dedicated space, disinfected using measures effective against sporulating bacteria, will be used
- ✓ Protective gloves and facial masks will be used during preparation
- ✓ Materials used for preparation and bags with final product have to be sterile.
- ✓ The bag containing the final product must be labelled with the following information: donor identification, date of preparation, time limit for administration, storage temperature.
- ✓ Before transplantation faecal material will be suspended in 200 mL NaCl 0.9% solution
- ✓ 5 g of donated material should be stored (-80° C) for possible future controls as well as a sample of final product.

6.3.4 WP4: Laboratory Assessment

Units 8, 9

A. **Immunological studies** will be performed at the time-points indicated in WP2 (at baseline and months 3,6,9,12). The following immunological aims will be pursued:

1) To identify the blood and mucosal changes in the absolute number, percentage and phenotype of Tregs, comparing baseline and study end between different treatment doses and control group. Tregs will be defined as $CD4^{+}CD25^{+}CD127^{-}FoxP3+$ T cells. Method: polychromatic flow cytometry (PFCM), using a combination of specific mAbs 2) To identify changes in distribution of T cell substes especially the ratio Tregs/Th1or Treg/Th17. In detail, evaluation of the functional role of T cell subsets in peripheral blood e gut mucosa: T cells will be isolated from peripheral blood and gut mucosa. We will isolate T cells through anti human CD3 microbeads (Miltenyi, Germany) from the tissue samples processed with the GentleMACS tissue dissociator. The distribution of the various T cell sub-sets will be evaluated by FACS analysis.

3) To identify changes in levels of pro-inflammatory cytokines and cytokines linked to T cell proliferation and differentiation. Methods: quantification by luminex technology of the cytokine profile of serum, salivary and stool samples. In detail, we will evaluate the samples for MIP1a, IL-27, IL-1β, IL-2, IL-4, IL-5, IP-10, IL-6, IL-8, IL-10, IL-12p70, IL-13, IL-17A, IFNγ, GM-CSF, TNFα, IFNα, MCP-1, IL-9, P-selectin, IL-1α, IL-23, IL-18, IL-21, sICAM-1, IL-22, E-selectin content using specifically assembled kits (Custom Mix&Match panel Human Panel- 27 Plex) for Luminex Screening Assays (Affymetrix, eBioscience)

4) Evaluation and correlation of immune products in the fecal and serum samples
The statistical analysis will be performed using STATISTICA software. If the data will not be normally distributed, medians and interquar-tile ranges (Q1 and Q3) will be calculated for all

Via Giardini, 1355
Baggiovara
41126 Modena
tel. : 059396111
fax.: 0593962409

groups. Principal components analysis (PCA) will be used for exploration of the data and tentative classification of the case volunteers. For assessing significant differences between low-and high-cytokine-values subgroups before intervention, the non-parametric Mann-Whitney U test will be performed. For assessing significant differences before and after the intervention period for any group/subgroup, the non-parametric Wilcoxon test will be applied. In both cases, $P<0.05$ will considered to be significant.

5) To assess biomarker changes, namely plasma/CSF neurofilament heavy/light chain protein (NF).
Methods: an aliquot of the withdrawn blood will be processed and stored at -80°C together with CSF samples until NF measurements; CSF sample collection and analysis to quantify NF will be performed before and after treatment.

Unit 1,2,3,4,5,6,8,9 and coordinating Unit will be responsible for sample collection at scheduled visits. Partner 8 and 9 will receive all biological samples from partners 1,2,3,4,5,6, the day after (within 9 a.m.) the blood sample (which will be withdrawn in the afternoon); they will process blood and CSF and perform all of the aforementioned analysis leading to assess immunological profile of treated and not-treated patients.

B. **Intestinal microbiota studies** will be performed at the time-points indicated in WP2 (at baseline and months 6,12). Characterization of intestinal microbiota and bacterial SCFA composition from feaces, saliva and gut tissue in ALS patients. The following microbiological aims will be pursued:

1) Bacterial DNA processing and Analysis through a "Next-Generation-Sequencing" (NGS) approach
Gut microbiota caracterization will be performed at the Department of Clinical and Experimental Medicine, University of Florence, Florence. Total DNA will be extracted from saliva, blood and fecal samples through PowerSoil®DNA Isolation Kit (Mobio Laboratories inc.). DNA, extracted by microbiologists of Department of Microbiology – Policlinico Universitario "A. Gemelli", Rome, will be used for PCR amplification of the V3-V4 variable regions of the bacterial 16S gene, to ensure the necessary information for the taxonomic classification of gut microbiota microbial communities. The amplification products will be processed by the Illumina MiSeq NGS platform (Illumina Inc.).
Data analysis will be carried out through specific softwares. In a first step, the sequences generated by massive sequencing technologies will be subjected to a quality control by eliminating low quality nucleotides. The obtained sequences will be used as input for a clustering algorithm that will assign them to "Operational Taxonomic Units" (OTUs) based on their level of nucleotide sequence identity. Also, in parallel, the presence of chimeric sequences will be checked and corrected using two standard approaches, one unsupervised approach based on the content of replicated sequences in the sample and a supervised one based on publically available 16rDNA databases. The last step will be the identification of the most likely target taxonomic annotation of each sequence by comparing them with sequences in taxonomy-oriented databases. The data obtained for each sample (patient) will be compared with each other in order to assess possible differences in the composition of the intestinal

Via Giardini, 1355
Baggiovara
41126 Modena
tel. : 059396111
fax.: 0593962409

flora. This last step will involve multivariate statistical techniques and the creation of mathematical models based on the bacterial composition obtained. The most common bioinformatics tools (eg FastQChttp://www.bioinformatics.babraham.ac.uk/projects/fastqc/ and SolexaQAhttp://solexaqa.sourceforge.net/) will be used to control the quality of the massive sequencing data. The controlled sequences will be subjected to the process of "gene calling" to identify the coding regions. This process will be conducted with intrinsic and extrinsic methods. Intrinsic methods are based on identifications of start codons or valid reading frame that lead back to the presence of a gene on the DNA fragment analysed. The extrinsic methods require the use of specific sequence similarity-based tools for probing the unknown sequence (query) specific databases embedding already characterized sequences. In the presence of a positive match, gene based information (e.g. functional annotation) can be transferred from the database sequence to the query. Two types of algorithms will be used to identify the main taxonomic signatures present in the analysed samples. The first type is based on the comparison of DNA sequences obtained with a raw bacterial database containing specific taxonomically sequences.

This method will assign a sequence to a specific organism with a high level of reliability and specificity, making it possible to generate high resolution taxonomic profiles (containing species or strains). The second method that will be used is based on an algorithm called "Lowest common ancestor" (LCA). This approach is based on the comparison of metagenomic sequences against a nucleotide reference database, to generate a list of possible taxonomical annotations in the form of a hierarchical structure (a taxonomic tree). From this structured list it will be possible to assign each analysed sequence to a single taxonomic category climbing the taxonomic tree in the search of a node that contains all the previously identified hits. The two methods will provide two different taxonomic overview, a less detailed but more comprehensive one that can provide information on general bacterial categories (Phylum, Families or classes), and a second one, less comprehensive but very detailed, with a lower number of assignments. This, in turn, can provide details on the bacterial composition in terms of species and/or strains. The two descriptions obtained will be compared in order to ensure the agreement and the presence of no discrepancies in the number and / or the type of assignments found.

2) Quantitative/qualitative analysis of SCFAs in fecal samples and correlation with gut microbiota

Analysis of Short Chain Fatty Acids (SCFAs) will be performed at the Department of Experimental and Clinical Medicine, University of Florence, Florence. The fecal determination of SCFA (mainly acetic, propionic, butyric acids, isobutyric, valeric, isovaleric acids) will be performed using a gas-chromatography coupled with mass spectrometry (GC-MS) method. Furthermore, to increase the quantitative reliability of the SCFA assay, we will use the isotopic dilution (ID) technique. The ID involves the employ of an isotopologue of the analyte as internal standard (ISTD), allowing the correct analyte monitoring, during the analytical sample manipulations, and its better quantitation, in detection phase. The use of the GC-MS/ID approach joins the sensitivity and specificity of the revelation to the reliability of the quantitative determination also small amounts of the analytes present in the sample. The SCFA will be extracted from the samples and analyzed by GC-MS in free form employing a polyethylene glycol column (WAX) that allows ease the separation of the SCFA series. The parameters setup will be carried out using a mixture of SCFA between C1-C5 terms (the most

Via Giardini, 1355
Baggiovara
41126 Modena
tel. : 059396111
fax.: 0593962409

representative compounds), but the proposed method allows the determination also the heavier homologous (C6-C10....). Moreover, the use of GC-MS guarantees the revelation also of branched isomers of SCFA that can be important to differentiate the active microorganism strains in the samples.The correlations between metabolites and bacterial composition will be determined using the correlation Pearson index "r" in which r less than or equal to 0.50 will be considered a moderate correlation, while at a major or equal to 0.70 will be considered as a strong correlation. The bacterial communities obtained by analyzing metagenomic sequences will be analyzed to assess differences between different cohorts of patients (at baseline, month 6 and 12). For this purpose, multivariate statistical techniques will be used such as principal component analysis, canonical correlation analysis and hierarchical clustering. Once the statistical dependence between the bacterial communities identified and various diseases will be assessed, it will be possible to analyze the correlations within communities using biological network, generated through the use of correlation matrices such as Pearson and Spearman. Sorting algorithms will be applied to identify which parts of the community are more interconnected and which are not related by displaying network. In the end it will be possible to identify a highly represented bacterial "core" interconnected in all patients and a community "accessory" feature instead only of certain diseases. The identification of these communities will be used for the production of a database and taxonomies functions bacterial correlate with the presence of particular disease states.

3) Isolation of bacteria in fecal samples by colturomics

For many years, several bacteria were considered non-culturable and so cultures were not used to study gut microbiota, but in the last years, the cultural approach is having a rebirth with culturomics.

Culturomics is a labourious and time-consuming approach which is considered very useful to study living bacteria. Using culture methods, 90 new strains were isolated (Langier et al. 2015)

Colturomics will be performed by Dr. Luca Masucci – Rome. 17 cultural conditions will be used in order to isolate bacteria in feces. Thirty grams of each fecal sample will be dissolved and re-suspended in 30 ml of saline solution. After the homogenization, there is a filtration step using patented filters, present in the Parasep® Fecal Parasite Concetrators kit (Apacor Limited – Sapphire Center – Wokingham, England). In these filters, large particles are rejected without occluding the 425µm pores, according to manufacture instructions.

The filtered 30 ml will be divided into three aliquots of nearly 10 ml each and then will be centrifuged at 3500g for 10 minutes. Supernatants will be discarded and each one of the three pellets will be resuspended one in 5 ml of Brain Hearth Infusion (BHI, Oxoid Deutschland GmbH – Wesel, Germany), one in 5 ml of supplemented Brucella Broth (BB, Remel INC. – Lenexa, USA) and the last in 5 ml of rumen fluid and they will be cultivated as below:

1.	Pre-incubation in paediatric blood culture bottle with rumen fluid and then 5% sheep blood, Chocolate and McConkey agar under aerobic, aerobe with 2.5% CO_2 and microaerophilic conditions at 37°C
2.	Pre-incubation in anaerobic blood culture bottle with rumen fluid and then Columbia and Schaedler agar under anaerobic conditions at 37°C
3.	Pre-incubation in paediatric blood culture bottle with Brucella broth and then 5% sheep blood, Chocolate and McConkey agar under aerobic, aerobe with 2.5% CO_2 and microaerophilic conditions at 37°C agar under aerobic conditions at 37°C
4.	Pre-incubation in anaerobic blood culture bottle with Brucella broth and then Columbia and Schaedler agar under anaerobic conditions at 37°C
5.	Pre-incubation under aerobic conditions in BHI and then 5% sheep blood, Chocolate and McConkey agar under aerobic, aerobe with 2.5% CO_2 and microaerophilic conditions at 37°C agar under aerobic conditions at 37°C
6.	Pre-incubation in anaerobic blood culture bottle with stool filtered at 5 m and then Columbia and Schaedler agar under anaerobic conditions at 37°C
7.	Pre-incubation in paediatric culture bottle with stool filtered at 5 m and then 5% sheep blood under aerobic conditions at 37°C
8.	Pre-incubation in paediatric blood culture bottle with 5 mL BHI and then 5% sheep blood, Chocolate and McConkey agar under aerobic, aerobe with 2.5% CO_2 and microaerophilic conditions at 37°C agar under aerobic conditions at 37°C
9.	Pre-incubation in anaerobic blood culture bottle with 5 mL BHI and then Columbia and Schaedler agar under anaerobic conditions at 37°C

The first step for analysis of microbiota will be performed by MALDI-TOF, Mass Spectrometry, on a Bruker MicroFlex LT (Bruker Daltonics, Billerica, MA).
For plate extractions, 1 to 4 colonies from subcultures, after growth according to routine protocols (≤24 hours), will be spotted on the target plate. Each spot was overlaid with 1 µl of alpha-cyano-4-hydroxycinnamic acid (α-CHCA).
Spectra will be acquired from 1,000 laser shots at a minimum intensity threshold of 30 arbitrary units (A.U.) Plate calibration will be performed on each run before spectral acquisition using a mixture of two proteins contained in BTS (Bruker Daltonics).
The BTS has been developed for the Quality Control process of the MALDI Biotyper system. Its specific composition covers the entire mass range of proteins used by MALDI Biotyper for precise identification of microorganisms, making BTS ideal for use with this mass spectrometry system. When BTS is used, MALDI Biotyper performs an automatic QC before each identification run. The QC process includes calibration of the mass spectrometer, a laser setting check and evaluation of spectrum quality. System performance is finally confirmed by achieving *Escherichia coli* identification which must pass minimum performance criteria. The result is documented on each identification run report.
Plate calibration met acceptable specifications when at least seven of the eight proteins were detected within 150 ppm of the “in last fit” parameter. Flex Analysis software (Version 3.4; Bruker Daltonics).
Isolated bacteria will be frozen at – 80 °C in 0.9% saline solution+10%glycerol

Via Giardini, 1355
Baggiovara
41126 Modena
tel. : 059396111
fax.: 0593962409

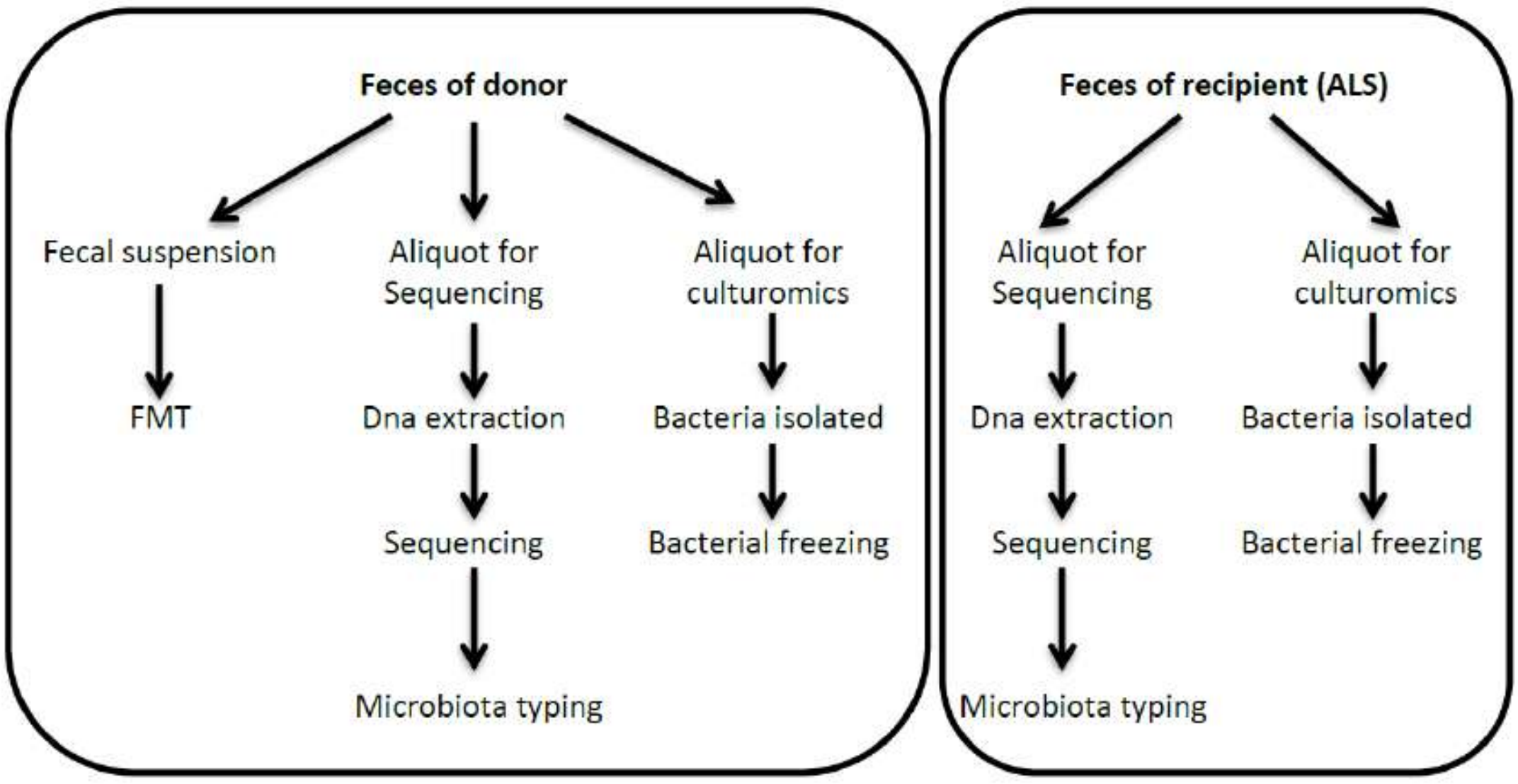

6.3.5 WP5: Analysis of data and results dissemination

All Units involved, coordination of this part by Unit 7

Data extraction:
The investigators will collect data by entering them directly into the trial database, through electronic case report forms ad hoc developed. At the end of the RCT, database will be locked and an expert Statistician (Prof. R. D'Amico) will check the database and extract all data for statistical analyses.

Statistical analysis: The analyses will be performed by intention-to-treat. Per protocol analysis will be carried out after excluding non-compliers (patients who will do the second round of FMT) and drop-out for any reason. Statistics will be tabulated by treatment arm and time. Results with p-values less than 0.05 will be considered as statistically significant. Statistical analyses will be carried out using Stata 12 software (Stata Corporation, College Station, Texas). Randomization and statistical analysis will be performed by independent persons who are not involved with patients.

Presentation and dissemination of results.
Results will be presented as appropriate effect estimates (mean differences, relative risks, hazard ratios) along with their relative 95% confidence intervals. The report on final results will be first communicated to MoH.

The PI and all the partners will guarantee the dissemination and exploitation of the scientific results within the consortium and externally (international conferences, publications, etc).

Dissemination of results will include national and international neurological and ALS meetings and workshops. We will organize the participation to conferences, where we will present our work, allowing to the units to achieve visibility.

We will schedule, plan and coordinate the writing of scientific papers (so that delays in manuscript preparation will be avoided). Results will be published in peer-reviewed international journals.

Any publication of results will report MoH contribution.

7. STUDY POPULATION

The study will include probable laboratory-supported, clinically probable, or definite ALS according to revised El Escorial criteria (sporadic and familiar).
To reduce phenotypic variability, we will stratify patients according to progression rate (ΔFS> or < to 0.7) [Kimura et al., 2006].
The study will be performed on 42 outpatients enrolled in 2 groups of 28 (FMT) and 14 (controls) subjects. Enrollment will be performed in the 6 ALS centers participating to the study. Enrollment period will last 18 months. Treatment duration will be 6 months and follow up will be 6 months.
The study will be carried out in accordance with the Declaration of Helsinki, as amended by the 64th WMA General Assembly, Fortaleza, Brazil, October 2013.
All subjects must sign and personally date an approved Informed Consent Form (ICF) after receiving detailed written and verbal information about the reason, the nature, the required procedures, the intended duration and the possible risks and benefits and any discomfort associated with the study. He/She should be informed that the subject's participation in the study is voluntary and that he/she may refuse to participate or withdraw from the trial, at any time, without penalty or loss of benefits to which the subject is otherwise entitled. The language used in the oral and written information about the trial, including the written Informed Consent Form, should be as non-technical as practical and should be understandable to the subject or the subject's legally acceptable representative and the impartial witness, where applicable. The subject must be given ample time to read and to understand the Patient Information Sheet and opportunity to inquire and ask any clarification about the trial before signing the ICF. For this reason patient information sheet will be given to the candidate patient at least 15 days before the collection of the ICF. In the patient information sheet the patient will find a telephone number of the IEAB that he/she could call to have independent information. Furthermore, when the neurologist will give the patient information sheet to the patient, the investigator will advise IEAB of a possible enrollment to allow IEAB member to be present at the moment of the ICF collection. Prior to a subject's participation in the trial, the written Informed Consent Form should be signed and personally dated by the subject or by the subject's legally acceptable representative, and by the a neurologist not involved in the care relationship with the patient, in addition to the physician who conducted the informed consent discussion. When applicable the Investigator may conduct the informed consent discussion in presence of an impartial witness, who should sign and personally date the Informed Consent Form (e.g. a member of the IEAB).
No study procedure can be performed before the written informed consent has been provided. The informed consent procedure must be done according to the guidelines provided in the Declaration of Helsinki and the ICH E6 Guideline for Good Clinical Practice. The subject must be made aware and agree that personal information may be scrutinized during audit by competent authorities and properly authorized persons. However, personal information will be treated as strictly confidential and will not be publicly available. By signing the Investigator Statement the Investigator assures the Sponsor that Informed Consent will be obtained. Both the Patient Information Sheet and the Informed Consent Form must be approved by the Ethics Committee with the study protocol.

Via Giardini, 1355
Baggiovara
41126 Modena
tel. : 059396111
fax.: 0593962409

Patient withdrawals and dropouts

Any study subject may be withdrawn from the study at any time either at the discretion of the investigator or at the request of the study subject.
The reason for doing so should be clearly documented in the Case Report Form (CRF).
However, the study subject will not be under any obligation to provide a reason for withdrawal.

7.1 INCLUSION CRITERIA

- Patients diagnosed with a laboratory supported, clinically "probable" or "definite" amyotrophic lateral sclerosis according to the Revised El Escorial criteria (Brooks, 2000)
- Sporadic or familial ALS
- Female or male patients aged between 18 and 70 years old
- Disease duration from symptoms onset no longer than 18 months at the screening visit
- Patients treated with a stable dose of Riluzole (100 mg/day) for at least 30 days prior to screening
- Patients with a weight > 50 kg and a BMI ≥18
- Patients with a FVC (Forced Vital Capacity) equal or more than 70% predicted normal value for gender, height, and age at the screening visit
- Patients able and willing to comply with study procedures as per protocol
- Patients able to understand, and capable of providing informed consent at screening visit prior to any protocol-specific procedures
- Use of effective contraception both for males and females

7.2 EXCLUSION CRITERIA

- Known organic gastrointestinal disease
- History of gastrointestinal malignancy; ongoing malignancies
- Use of immunosuppressive or chemotherapy within the past 2 years
- Celiac disease and/or food (e.g.lactose) intolerance
- Previous gastrointestinal surgery
- Any condition that would make endoscopic procedures contraindicated
- Acute infections requiring antibiotics
- Antimicrobial treatment or probiotics 4 weeks prior to screening
- Severe comorbidities (heart, renal, liver failure); severe renal (eGFR< 30ml/min/1.73m2), or liver failure or liver aminotransferase (ALT/AST > 2x Upper limit of normal),
- Autoimmune diseases, inflammatory disorders (SLE, Rheumatoid arthritis, connective tissue disorder) or chronic infections (HIV, hepatitis B or C infection)
- Abuse of alcohol or drugs
- HIV, TBC, hepatitis
- Participation in clinical trials <30 days before screening
- Existing blood dyscrasia (e.g., myelodysplasia)
- White blood cells<4,000/mm^3, platelets count<100,000/mm^3, hematocrit<30%
- Patients who underwent non invasive ventilation, tracheotomy and /or gastrostomy

- Women who are pregnant or breastfeeding

7.3 PATIENT IDENTIFICATION

All patients who have signed the informed consent document will receive a patient number that serves to identify the patient throughout the study.
Patients will be identified by a numeric code including the centre number, followed by a chronological inclusion number for that centre (XXX-YY), the first two letters of the last name and first name.
All subjects will receive a unique subject identification number at screening visit when signing the informed consent by the owner and before any study procedures are performed. This number will be used to identify the subject throughout the study and must be used on all study documentation related to that subject. The subject identification number must remain constant throughout the entire study; it must not be changed at the time of enrolment, or randomization.

7.4 DONORS SELECTION

Feces will be obtained by habitual healthy donors for *C. difficile* infection. The same donor will give a stool sample both for the first (baseline) and the second (month 6) FMT.
Wherever possible and according to CNT dispositions on FMT, fresh feces will be preferred for transplantation; in case of high risk for infections, frozen feces will be used according to CNT directives (see paragraph "E" of "BLOOD AND STOOL TESTING TO CHECK DONORS FOR ANY POTENTIALLY TRANSMITTABLE DISEASE" section).
Habitual healthy donors have been selected following the next key issues:

EXCLUSION CRITERIA:

A) INFECTIOUS DISEASES RISK

‣ History of, or known exposure to, HIV, HBV or HCV, syphilis, human T-lymphotropic virus I and II, malaria, trypanosomiasis, tuberculosis
‣ Known systemic infection not controlled at the time of donation
‣ Use of illegal drugs
‣ Risky sexual behaviour (anonymous sexual contacts; sexual contacts with prostitutes, drug addicts, individuals with HIV, viral hepatitis, syphilis; work as prostitute; history of sexually transmittable disease)
‣ Previous reception of tissue/organ transplant
‣ Previous (<12 months) reception of blood products
‣ Recent (<6 months) needle stick accident
‣ Recent (<6 months) body tattoo, piercing, earring, acupuncture
‣ Recent medical treatment in poorly hygienic conditions
‣ Risk of transmission of diseases caused by prions
‣ Recent parasitosis or infection from rotavirus, Giardia lamblia and other microbes with GI involvement

Via Giardini, 1355
Baggiovara
41126 Modena
tel. : 059396111
fax.: 0593962409

‣ Recent (<6 months) travel in tropical countries, countries at high risk of communicable diseases or traveller's diarrhoea
‣ Recent (<6 months) history of vaccination with a live attenuated virus, if there is a possible risk of transmission
‣ Healthcare workers (to exclude the risk of transmission of multidrug-resistant organisms)
‣ Individual working with animals (to exclude the risk of transmission of zoonotic infections)

B) GASTROINTESTINAL (GI), METABOLIC AND NEUROLOGICAL DISORDERS
‣ History of inflammatory bowel syndrome or disease, functional chronic constipation, coeliac disease, other chronic GI disorders
‣ History of chronic, systemic autoimmune disorders with GI involvement
‣ History of, or high risk for, GI cancer or polyposis
‣ Recent appearance of diarrhoea, hematochezia
‣ History of neurological/neurodegenerative disorders
‣ History of psychiatric conditions
‣ Overweight and obesity (body mass index >25)

C) DRUGS THAT CAN IMPAIR GUT MICROBIOTA COMPOSITION
‣ Recent (<3 months) exposure to antibiotics, immunosuppressants, chemotherapy
‣ Chronic therapy with proton pump inhibitors

ISSUES TO ADDRESS ON THE SAME DAY OF DONATION TO CHECK ANY RECENTLY ONSET OF HARMFUL EVENTS

The following issues, if present, contraindicate donation on the same day on which they are assessed:
‣Newly appeared GI signs and symptoms, for example, diarrhoea, nausea, vomiting, abdominal pain, jaundice
‣ Newly appeared illness or general signs as fever, throat pain, swollen lymph nodes
‣ Use of antibiotics or other drugs that may impair gut microbiota, new sexual partners or travels abroad since the last screening
‣ Recent ingestion of a substance that may result harmful for the recipients
‣ Travel in tropical areas—contact with human blood (sting, wound, showing, piercings, tattoos)—sexual high-risk behaviour
‣ Diarrhoea (more than three loose or liquid stools per day) among members of the entourage (including children) of the donor

BLOOD AND STOOL TESTING TO CHECK DONORS FOR ANY POTENTIALLY TRANSMITTABLE DISEASE

A) GENERAL BLOOD TESTING
‣ Cytomegalovirus
‣ Epstein-Barr virus

Via Giardini, 1355
Baggiovara
41126 Modena
tel. : 059396111
fax.: 0593962409

- Hepatitis A
- HBV
- HCV
- Hepatitis E virus
- Syphilis
- HIV-1 and HIV-2
- Entamoeba histolytica
- Complete blood cell count with differential
- C-reactive protein and erythrocyte sedimentation rate
- Albumin
- Creatinine and electrolytes
- Aminotransferases, bilirubin, gamma-glutamyltransferase, alkaline phosphatase

B) BLOOD TESTING IN SPECIFIC SITUATIONS
- Human T-lymphotropic virus types I and II antibodies
- Strongyloides stercoralis

C) GENERAL STOOL TESTING
- Detection of Clostridium difficile
- Detection of enteric pathogens, including Salmonella, Shigella
- Campylobacter, Escherichia coli O157 H7, Yersinia, vancomycin-resistant enterococci, methicillin-resistant Staphylococcus aureus, Gram-negative multidrug-resistant bacteria
- Norovirus
- Antigens and/or acid fast staining for Giardia lamblia and Criptosporidium parvum
- Protozoa (including Blastocystis hominis) and helminths
- Faecal occult blood testing

D) STOOL TESTING IN SPECIFIC SITUATIONS
- Detection of Vibrio cholera and Listeria monocytogenes
- Antigens and/or acid fast staining for Isospora and Microsporidia
- Calprotectin
- Helicobacter pylori faecal antigen
- Rotavirus

E) Before preparation, sample donation will be always analyzed by rapid molecular test to detect intestinal pathogensISSUES TO BE ADDRESS TO PREVENT COVID-19 INFECTION
This study protocol will be performed in accordance with CNT directives on FMT that may change together with the national trend of COVID-19 infection and during different time periods.
According to current CNT dispositions, the following measures have to be addressed unless different indications:
- donor evaluation has to be strengthened by checking if previous (30 days) SARS-COv-2 positivity was detected by pharyngeal swab, and asking for symptoms compatible with Covid-19 infection

(including fever, tiredness, dry cough , myalgia, dyspnoea and headache) and asking for close contact with infected or suspected infected individuals.
- execution of the specific test for SARS-Cov-2 on respiratory secretions (pharyngeal swab) taken on the day of the donation and freezing of the feces, in derogation from the National FMT Program.
- before using feces for transplantation, in addition to the test results, it will be necessary to repeat a donor assessment regarding the appearance of symptoms compatible with SARS-COv-2 infection within 30 days after the donation (post donation information). In case of positivity or appearance of symptoms, the donor must be considered unsuitable and the National Transplant Center must be informed on this.

8. STUDY TREATMENT

8.1. STUDY TREATMENT DEFINITION

The study will include 42ALS patients with 2:1 allocation in 2 groups of subjects (28 FMT vs 14 controls); computerized randomization will be stratified by ΔFS (</≥0.7)[21]. Randomization time: 18 months. Treatment will be double blinded to patients and physicians and will be done at baseline and at month 6. Active treatment will be FMT. Post-treatment follow up: 6 months.
ALS patients will undergo upper GI endoscopy with small-intestine biopsies (n° 4 biopsies of small intestine, performed with a standard biopsy forces) at baseline and after 6 and 12 months. At baseline patients will be randomized (2:1) to either an allogenic (from donors) infusion of collected feces (60 grams) (fecal microbiota transplantation, FMT) in the duodenum-jejunum or no treatment (controls). The infusion will be performed through a standard nasojejunal tube, that will be placed during endoscopy. Infusion of feces will be repeated at month 6. When possible, the same donor will give a stool sample both for the first (baseline) and the second (month 6) infusion. Control group patients will not receive any treatment (at baseline or at month 6), but will remain blind to treatment because of deep sedation (with anesthetist) used for small-intestine biopsies. Fecal microbiome will be diluted in saline solution 200 ml and infused at 30 ml/minute speed (whole amount of the performance: 15 minutes).
Every endoscopic procedure will be performed with deep sedation of the patient. Feces for FMT will be obtained by habitual healthy donors for C. difficile infection.
Procedures that are usually performed for the selection of donors for transplantation of feces: potential donors stool had been chosen in healthy volunteers that have been given a questionnaire with questions about lifestyle, health status, current therapy, etc., significant clinical symptoms of gastrointestinal disease, etc.. (For details, see. Questionnaire Annex). Based on this questionnaire, the potential donor has been considered eligible if were excluded: I) Habits of life and risk behaviors, II) Concomitant significant known disorders, III) chronic or recent use of concomitant medications that may interfere with the state of the intestinal microflora (eg, antibiotics), IV) Clinical symptoms indicative of gastrointestinal disease or other diseases of importance, V) Personal or family medical history known of neurodegenerative diseases or other autoimmune diseases.

Each suitable potential donor has been subjected to the following screening tests: I)Examination of stool for *Clostridium difficile* bacterial pathogens and protozoa and helminths of the small intestine and colon, VRE (Vancomycin-resistant Enterococci), MRSA (Methicillin-resistant Staphylococcus aureus), Gram-negative MDR (Multidrug-Resistant Organisms), *H. pylori* fecal antigen II) Serological screening for HAV, HBV, HCV, HIV 1-2, Treponema pallidum, blood count with differential, dose transaminasemia, creatinine and C-reactive protein.
Potential donors negative for this screening have been considered definitively suitable and will be invited to give a stool sample for FMT. The preparation of faeces (60 grams; homogenization in 200 mL of saline solution) for infusion will be performed at the UOC of Analysis 2 ° (Virology and Microbiology).
Fresh or frozen feces will be used according to CNT directives.
Analysis of T cell sub-populations will be performed both in peripheral blood and gut mucosa: T Regolatory cells (Tregs), Th17 cells, effector and central memory cells, NK and B-cells.

8.2. PATIENT COMPLIANCE

Compliance will be assessed by the investigator through adherence to both treatment rounds, and by adherence to follow up visits from baseline to month 12. Compliance of riluzole will be assessed too.

8.3. CONCOMITANT MEDICATIONS

All medications taken by the patients at the onset of study and all medication given during the study are regarded as concomitant medications.

8.3.1. Mandatory Concomitant Treatment

Patients must have been treated for a minimum of 1 month with a stable dose of riluzole (100 mg/day) at baseline. Safety issues related to riluzole should be managed according to usual practice.

8.3.2. Prohibited Concomitant Treatment

There are no prohibited concomitant medication except for:
- Probiotics of any kind
- Experimental drugs of any kind (including experimental drugs for ALS)
- Treatments with stem cells, antisense oligonucleotide or any other kind of advanced cellular treatment

Special warnings and precautions for use

Antibiotics and laxatives can alter fecal microbiota; they should be taken with caution and only if clinically appropriate

8.4 RANDOMIZATION

Eligible patients will be sequentially allocated to the 2 arms with a 2:1 ratio by using a computer-generated list of random number that will be centrally generated in the Statistical Unit based at the University of Modena. After the investigators will obtain the person's consent, they will connect to a website dedicated to the study, in which the randomisation sequence is concealed.

All patients will receive a unique patient identification number at screening visit when signing the informed consent and before any study procedures are performed. This number will be used to identify the patient throughout the study and must be used on all study documentation related to that patient. The patient identification number must remain constant throughout the entire study; it must not be changed at the time of enrolment, or randomisation.
42 patients will be randomized in the following three groups:

- 28 patients: FMT + Riluzole
- 14 patients: Riluzole

Stratification
Randomization will be performed on line. Patients will be stratified according to ΔFS (</≥0.7)[21], with 2:1 allocation in 2 arms. The investigator will randomize patients directly on line. Endoscopic treatment must be done within 21 days from randomization. In case of discontinuation from the study, the randomization number will not be reused. Enrolment time: 18 months.

8.5. BLINDING PROCEDURES

Eligible patients will be randomized by means of a computerized central randomization system. The automated system will assign the appropriate treatment for each patient. The Statistics Unit based at the University of Modena will supply the investigators with user guides for the automated system.
This study is a double-blind study: during the entire study duration neither the patient nor the neurologist will know what kind of treatment will be given to the patient. The investigator will be provided with technical options and password information to selectively break the code for an individual patient by telephone, facsimile transmission, or through electronic message transfers.
The premature breaking of the code should be confined to emergency cases in which knowledge of the treatment is necessary for adequate treatment. Whenever possible, the Statistics Unit based at the University of Modena should be contacted before breaking the blinded emergency code. Should any code be broken, the respective patient will be withdrawn from further participation in the study and a written explanation must be given by the investigator.

8.6. MANAGEMENT OF TOXICITY

8.6.1. Procedures to manage adverse events potentially related to study treatment

Adverse Events

According to Directive 2004/23/EC (implemented in Italy with D.Lgs 191/2007):

- "Serious adverse event" (SAE) means any untoward occurrence associated with the procurement, testing, processing, storage and distribution of tissues and cells that might lead to the transmission of a communicable disease, to death or life-threatening, disabling or incapacitating conditions for patients or which might result in, or prolong, hospitalisation or morbidity;
- "Serious adverse reaction" (SAR) means an unintended response, including a communicable disease, in the donor or in the recipient associated with the procurement or human application of tissues and cells that is fatal, life-threatening, disabling, incapacitating or which results in, or prolongs, hospitalisation or morbidity.

Centres are requested to report immediately from their awareness any SAE or SAR occurring during the trial.A report form will be available on the online CRF.
Completed forms should be forwarded by email to the coordinating centre and principal investigator (j.mandrioli@ausl.mo.it) and to Italian National Transplantation Centre (CNT) (cnt@iss.it, letizia.lombardini@iss.it e cristina.pintus@iss.it)

The coordinating Centre will be responsible for appropriate AE reporting to the regulatory authorities (CNT) every six months; investigators will be responsible for reporting to appropriate Ethic Committee. In case of death a clinical report will be prepared by the caring investigator together with SAE form; in case of autopsy, autopsy report will be added to study documentation.
Safety Data Collection will include classification of the event (serious or non-serious AE), description of signs or symptoms, diagnosis (where possible), onset and stop (date and time), intensity, correlation between the study agent and the adverse event as follows (probable, possible, not related, unknown), action taken (none; change in treatment administration; drug treatment required; non-drug treatment required; hospitalisation or prolonged hospitalisation; diagnostic or clinical test(s); discontinuation from the study), subject outcome.

Adverse events reported with FMT

A recent review (Wang et al., 2016) examined 50 publications, for a total of 1089 patients that were treated with FMT (age range: 1–95 years). Among them, 831 patients were affected by refractory or recurrent CDI, of whom 106 had concomitant IBD; 235 were affected by independent IBD; 1 affected by early onset colitis; 9 affected by antibiotic-associated diarrhea (AAD), of whom 6 had concomitant CDI; and 13 affected by IBS.
Overall, 78 kinds of AEs were reported to happen on 310 patients during or after FMT. The majority of AEs were presented as mild symptoms such as abdominal discomfort, diarrhea, transient fever, nausea, vomiting and constipation.
The overall incidence of AEs was 28.5% (310/1089).

Table 1: Attributable adverse events for upper gastrointestinal route FMT

First Author, Year	AEs	N. of pts with AEs	Sample size	Causality between AEs and FMT	Routes of infusion
Upper gastrointestinal routes					
Vermeire, 2012	Fever; Abdominal tenderness	3	4	Probably	Nasojejunal tube
Cui, 2014	Fever; Increased diarrhea	7	30	Probably	Gastroscopy (mid-gut)
Van Nood, 2013	Belching; Nausea; Abdominal cramps; Diarrhea; Abdominal pain; Infection; Dizziness combined with diarrhea; Constipation	27	29	Probably	Nasoduodenal tube
Aas, 2003	Death from peritonitis	1	18	Possibly	Nasogastric tube
MacConnachie, 2009	Upper gastrointestinal hemorrhage	1	15	Possibly	Nasogastric tube
Kronman, 2015	Vomiting; Mucoid stools	1	10	Probably	Nasogastric, nasoduodenal or nasojejunal tube
Pinn, 2014	Flatulence	1	13	Probably	Esophagogastroduodenoscopy
Wang, 2013	Diarrhea	5	16	Probably	Gastroscopy
Suskind, 2015	Rhinorrhea; Sore throat; Abdominal pain; Bloating; Diarrhea; Flatulence	5	9	Probably	Nasogastric tube
Suskind, 2015	Nasal stuffiness; Flatulence	1	4	Probably	Nasogastric tube
Rossen, 2015	Discomfort tube placement; Fever; Nausea; Malaise; Increase of stool frequency/diarrhea; Headache; Vomited fecal infusion; Vomited bowel prep; Vomiting; Abdominal cramps; Abdominal pain; Abdominal murmurs; Dizziness; Mild constipation	34	48	Probably	Nasoduodenal tube
Borody, 2003	Sore throat	3	8	Definitely	Nasojejunal tube
Lower gastrointestinal routes					
Kump, 2013	Fever; Temporary increase of CRP and IL-6; Increase in stool frequency	1	6	Probably	Colonoscopy
Zhang, 2013	Severe cold	1	1	Possibly	Colonoscopy
Quera, 2013	Fever; Bacteremia	1	1	Probably	Colonoscopy
Kunde, 2013	Fever; Chills; Abdominal fullness	2	10	Probably	Enema
Gustafsson, 1998	Diarrhea	3	9	Probably	Enema
Lee, 2014	Transient constipation; Excess flatulence	9	94	Probably	Enema
Hamilton, 2012	Irregularity of bowel movements; Excessive flatulence	14	43	Probably	Colonoscopy

Via Giardini, 1355
Baggiovara
41126 Modena
tel. : 059396111
fax.: 0593962409

Khoruts, 2010	Constipation; Irregularity of bowel movements	1	1	Probably	Colonoscopy
Pierog, 2014	Appendicitis	1	6	Possibly	Colonoscopy
Silverman, 2010	Urinary tract infections	2	7	Possibly	Enema
Hohmann, 2014	Cytomegalovirus colitis	1	1	Probably	Enema
De Leon, 2013	Transient relapse of UC	1	1	Probably	Colonoscopy
Schwartz, 2013	Norovirus Gastroenteritis	1	13	Probably	Colonoscopy

Safety rules

For the above mentioned toxicities investigators must treat symptoms according to the usual clinical practice. In case of symptoms that could be considered severe or life threating, the investigator must inform the sponsor immediately (within 24 hours by fax using a Serious Adverse Event form) and scond FMT round will be avoided accordingly to the local investigator's judgment. In accordance with the legislative requirements (D.Lgs. 191/2007 e dal D.Lgs 16/2010) Centres are requested to report immediately from their awareness any SAE or SAR occurring during the trial, and coordinating centre will send Completed forms by email to Italian National Transplantation Centre (CNT) (cnt@iss.it, letizia.lombardini@iss.it e cristina.pintus@iss.it).

9. EVALUATION CRITERIA

9.1. PRIMARY ENDPOINT

Change from baseline to month 6 in Tregs number in ALS patients treated with FMT compared to the control arm.

9.2. SECONDARY ENDPOINTS

9.2.1. BIOLOGICAL ACTIVITY ASSESSMENT

The following secondary variables will be assessed:

- Change from baseline to each time point (month 3, 6, 9, 12) of the T cell distribution especially the ratio Tregs/Th1 or Tregs/Th17comparing FMT arm and control group.
- Changes from baseline to each time point (month 3, 6, 9, 12) in inflammatory status (citokine profile) comparing FMT and control group.
- Changes from baseline to each time point (month 3, 6, 9, 12) in different biomarkers (including peripheral and CSF biomarkers i.e. creatinine and albumin, CK, vitamin D, plasma/CSF neurofilament heavy/light chain protein) comparing FMT arm and control group.

- Changes from baseline to each time point (month 6 and 12) in the composition of saliva, gut and fecal microbiota comparing FMT and control group.

9.2.2. SAFETY

The Safety will be assessed on the following variables:
Occurrence of adverse events (AEs) and per-treatment arising changes in physical examination, vital signs (blood pressure, pulse rate and body temperature), body weight, and clinical laboratory tests (biochemistry, haematology). We will refer to "ICH guidance for Clinical Safety Data Management: Definitions and Standards for Expedited Reporting" to report AEs and for safety outcome.

9.2.2.1. Adverse Events

An adverse event (AE) is defined as any modification of the clinical status of the patient, i.e. any emergence of a disease, sign or symptom, or modification of sign, symptom or concomitant disease, regardless of its relationship to study treatment.
All adverse events will be actively collected at each visit, from spontaneous declarations of the patient as well as from oral inquiry and clinical examination.
All AE should be noted in the case report form on the ad hoc "Adverse events" form. The investigator should specify its nature, date of onset, duration, outcome, actions taken, date of disappearance or stabilization. The investigator should evaluate the event in terms of severity, relationship to study treatment and seriousness.
Definitions for adverse event (AE) and serious adverse event/reaction (SAE/SAR), as well as procedures to follow in case of SAE are presented in Appendix 17.1.

9.2.2.2. Concomitant Treatments

All concomitant medications and/or therapies should be documented in the patient file and reported in the electronic Case Report Form (eCRF).

9.2.2.3. Laboratory Tests

The following parameters will be assessed:
Haematology
Haematology includes count of red blood cells, hemoglobin, hematocrit, total white blood cells count, platelet count, and a differential count including neutrophils, lymphocytes, monocytes, eosinophils, and basophils, and will be performed according to the Visit Schedules (see study flowcharts).
Biochemistry
Biochemistry includes urea, creatinine, albumin, protein electrophoresis, CK, AST (SGOT), ALT (SGPT), gamma GT, amylase, bilirubin, LDH, cholesterol, triglycerides, sodium, potassium, glucose, PCR, urinalysis, fecal calprotectin, will be performed according to the Visit Schedules (see study flowcharts).

Screening for infectious diseases
Screening for infectious diseases includes HIV, Hepatitis B and Hepatitis C.
Pregnancy test will be performed at screening (women of childbearing potential)

Via Giardini, 1355
Baggiovara
41126 Modena
tel. : 059396111
fax.: 0593962409

9.2.2.4. Physical Examination/Vital Signs

A physical examination including vital signs (systolic and diastolic arterial pressure, heart rate) will be performed according to the Visit Schedule. Information about the physical examination and vital signs must be present in the source documentation at the study site. Significant findings present prior to the start of study treatment must be included in the Medical History and Current Conditions CRF page. Significant findings made after the start of study treatment which meet the definition of an adverse event must be recorded on the Adverse Event CRF page.

9.2.1.5. Body Weight

Measurements of body weight will be performed according to the Visit Schedule. Body weight measurements performed during visits physical examination, will be captured on CRFs, which will calculate BMI.

9.2.1.6. Other Safety Parameters

Electrocardiogram and Chest X ray
An electrocardiogram and chest X-ray (only Posterior-Anterior view) will be performed at screening (unless done at diagnosis or in the previous 12 months).

Forced Vital capacity
The forced vital capacity (FVC) (percent of predicted normal) is the vital capacity (VC) measured when the patient is exhaling with maximal speed and effort. It will be determined, using the slow VC method. The VC can be measured using conventional spirometers that have had a calibration check prior to testing. A printout from the spirometer of all VC trials will be retained. Three VC trials are required for each testing session, and the highest VC recorded is utilized for eligibility.

9.2.3. CLINICAL ASSESSMENT

The following clinical variables will be assessed:

- Change from baseline to each time point (month 1, 3, 6, 9, 12) of ALSFRS-R
- Survival defined as the time from randomisation to the date of documented death or tracheotomy
- Change of Forced Vital Capacity (FVC) from baseline to each time point (month 1, 3, 6, 9, 12)
- Frequency of procedures (PEG, NIV, IV) from baseline to month 3,6,9,12

9.2.4. QUALITY OF LIFE

Quality of life will be assessed on the change in absolute value and percentage of following variables:

- Absolute and relative change from baseline in ALSAQ-40 (Amyotrophic Lateral Sclerosis Specific Assessment Questionnaire at month 6 and 12)

Via Giardini, 1355
Baggiovara
41126 Modena
tel. : 059396111
fax.: 0593962409

10. ASSESSMENT SCHEDULE

10.1. SCREENING VISIT

This visit will be performed in one of the recruiting Neurological ALS Centres (Modena, Firenze, Rome, Chieti, Perugia, Siena). Prior to any study activities, the patient will be asked to read the patient information sheet and sign an informed consent form that has been approved by the Independent Ethics Committees and which complies with regulatory requirements. Patients will be given adequate time to consider any information concerning the study given to them by the investigator. As part of the informed consent procedure, patients will be given the opportunity to ask the investigator any question regarding potential risks and benefits of a participation in the study. Patients will also have a telephone number of the IEAB to contact an independent person to ask further information as needed. Informed consent form will be collected and signed by an "independent" neurologist, who does not cure the patient. During the ICF collection a member of the IEAB (that would have been informed by email on the same day of the patient information sheet supply) could be present and sign the ICF too.

The following assessments and investigations will be conducted during this visit:

- Written informed consent
- Give information form for general practitioner
- Document the relevant past medical history, any planned surgery and current medical conditions not related to the diagnosis of ALS
- ALS history and diagnosis (date of diagnosis, signs and symptoms…)
- Relevant medical history and procedure history (start and end dates…)
- ALS medication history
- Concomitant medications and/or non-drug therapies, including the reason for administration
- Check of the patient's current practice for effective contraception
- Physical examination including vital signs, weight, height (BMI)
- Neurological examination
- Perform MRC, ALSFRS-R and FVC measurement
- Urine will be collected for urinalysis
- Blood sample will be collected for haematology and biochemistry
- Serum pregnancy test in females of child-bearing potential
- Screening for infectious diseases: Hepatitis B, Hepatitis C, HIV
- 12-lead ECG; in case a 12-lead ECG has been performed at diagnosis or within 12 months prior to screening, it might be used as baseline ECG
- Chest X-Ray (only Posterior-Anterior view); in case a chest X-Ray has been performed at diagnosis or within 12 months prior to screening, it might be used as baseline X-Ray
- Check of inclusion/exclusion criteria
- Recording of symptoms present at screening
- Appointment for the next visit

Via Giardini, 1355
Baggiovara
41126 Modena
tel. : 059396111
fax.: 0593962409

10.2. BASELINE VISIT

This visit will be performed in one of the recruiting Neurological ALS Centers (Modena, Firenze, Rome, Chieti, Perugia, Siena)
The baseline visit can occur from 1 day and up to 3 weeks after the screening visit when all results from the screening evaluation are available, and study treatment appointment at Gemelli Policlinico in Rome is available.

The following measures should be conducted before the randomization:

- Check of the patient's current practice for effective contraception
- Check of inclusion/exclusion criteria
- Physical examination including vital signs, weight, height (BMI)
- Blood sample will be collected for haematology and blood chemistry (analysis will not be repeated if Visit 2 occurs less than 7 days after Visit 1)
- Urinalysis
- Neurological examination
- Perform MRC, ALSFRS-R and FVC measurement
- ALSAQ – 40 (Amyotrophic Lateral Sclerosis Assessment Questionnaire)
- Perform lumbar puncture (LP) for biomarkers studies (collected in polypropylene tubes, centrifuged at 450 x g for 10 min at room temperature and stored at -80° C until analysis – see biological methods section)
- Blood sample for biological assessment and peripheral biomarkers
- Recording of menstrual cycles (non menopausal female patients)
- Query for medical procedures, hospitalizations, changes in concomitant medications and therapies
- Randomization and treatment allocation

The following measures should be conducted after randomization:

- Appointment for FMT to be performed at Gemelli Policlinico in Rome (within 4 weeks from screening)
- Ask the patient to come back 4 week later.

10.3 TREATMENT 1

This visit will be performed at Policlinico Gemelli in Rome.
This visit can occur from 1 day and up to 4 weeks after the screening visit when all results from the screening evaluation are available.

The following measures should be conducted before treatment:

- Check of the patient's current practice for effective contraception
- Physical examination including vital signs, weight, height (BMI)
- Blood, gut tissue (biopsies), saliva and feces samples collection
- Query for medical procedures, hospitalizations, changes in concomitant medications and therapies

Via Giardini, 1355
Baggiovara
41126 Modena
tel. : 059396111
fax.: 0593962409

Treatment will be then performed; the procedure will last approximately 15 minutes, will consist in deep sedation (with anesthetist) followed by gastroscopy and fecal infusion via naso-duodenal tube. Patients in the control group will not undergo any treatment, but they will be blind to treatment because of deep sedation used for small-intestine biopsies.
After the procedure the patient will be observed for 3 hours and then he could come back home; the appointment for the next treatment will be given (at six months).

10.4 VISIT MONTH 1 TREATMENT PERIOD

This visit will be performed in one of the recruiting Neurological ALS Centres (Modena, Firenze, Rome, Chieti, Perugia, Siena)
This visit will be performed at 1 month from treatment.
The following assessments and investigations will be conducted at each visit:

- Check of the patient's current practice for effective contraception
- Recording of adverse events
- Recording of menstrual cycles (non menopausal female patients)
- Query for medical procedures, hospitalizations, changes in concomitant medications and therapies
- Physical examination including vital signs, weight, height (BMI)
- Neurological examination
- Perform MRC, ALSFRS-R and FVC measurement
- Blood sample will be collected for haematology and blood chemistry
- Urinalysis
- Recording of dispensed treatment number(s) on CRF
- Appointment for the next visit

10.5. VISIT MONTH 3 TREATMENT PERIOD

This visit will be performed in one of the recruiting Neurological ALS Centres (Modena, Firenze, Rome, Chieti, Perugia, Siena)
This visit will be performed at 3 months from treatment.
The following assessments and investigations will be conducted at each visit:

- Check of the patient's current practice for effective contraception
- Recording of adverse events
- Recording of menstrual cycles (non menopausal female patients)
- Query for medical procedures, hospitalizations, changes in concomitant medications and therapies
- Physical examination including vital signs, weight, height (BMI)
- Blood sample will be collected for haematology and blood chemistry
- Urinalysis
- Neurological examination
- Perform MRC, ALSFRS-R and FVC measurement

Via Giardini, 1355
Baggiovara
41126 Modena
tel. : 059396111
fax.: 0593962409

- Blood sample for biological assessment and peripheral biomarkers
- Appointment for the next visit

10.6. VISIT MONTH 6

This visit will be performed in one of the recruiting Neurological ALS Centres (Modena, Firenze, Rome, Chieti, Perugia, Siena)
This visit will be performed 1 week to 1 day before the second treatment.
.
The following measures should be conducted:

- Check of the patient's current practice for effective contraception
- Physical examination including vital signs, weight, height (BMI)
- Blood sample will be collected for haematology and blood chemistry
- Urinalysis
- Neurological examination
- Perform MRC, ALSFRS-R and FVC measurement
- ALSAQ – 40 (Amyotrophic Lateral Sclerosis Assessment Questionnaire)
- Perform lumbar puncture (LP) for biomarkers studies (collected in polypropylene tubes, centrifuged at 450 x g for 10 min at room temperature and stored at -80° C until analysis – see biological methods section)
- Blood sample for biological assessement and peripheral biomarkers
- Recording of menstrual cycles (non menopausal female patients)
- Query for medical procedures, hospitalizations, changes in concomitant medications and therapies
- Ask the patient to come back 4 week later.

The following conditions will be considered for deciding to rule out the second FMT treatment:

- Patient's reduction of FVC with FVC<30% of predicted normal value for gender, height, and age (except for bulbar impairment conditioning spirometry reliability on clinical judgement)
- Severe ALS worsening with PEG positioning, invasive ventilation, or non invasive ventilation >14 h/day
- Any clinical condition that would make endoscopic procedures contraindicated
- Any laboratory condition that would make the procedure contraindicated, with particular attention to: platelets count<60,000/mm³, hematocrit<25%, hemoglobin <7.5 g/dl.

In case of contraindicating conditions that are potentially reversible (eg. acute infections requiring antibiotics, or potentially transient laboratory conditions), FMT may be delayed of a maximum of 4 weeks.

10.7 TREATMENT 2

This visit will be performed at Policlinico Gemelli in Rome.
This visit can occur from 1 day and up to 1 week after visit month 6.

Via Giardini, 1355
Baggiovara
41126 Modena
tel. : 059396111
fax.: 0593962409

The following measures should be conducted before treatment:
- Check of the patient's current practice for effective contraception
- Physical examination including vital signs, weight, height (BMI)
- Blood, gut tissue (biopsies), saliva and feces samples collection
- Query for medical procedures, hospitalizations, changes in concomitant medications and therapies

Treatment will be then performed; the procedure will last approximately 15 minutes, will consist in deep sedation (with anesthetist) followed by gastroscopy and fecal infusion via naso-duodenal tube. Patients of the control group will not undergo sham FMT, but will remain blind to treatment because of deep sedation used for small-intestine biopsies. After the procedure the patient will be observed for 3 hours and then he could come back home; the appointment for the next visit will be given (at six months).

10.8. FOLLOW UP VISITS, MONTH 7

This visit will be performed in one of the recruiting Neurological ALS Centres (Modena, Firenze, Rome, Chieti, Perugia, Siena)
This visit will be performed 1 month after the second treatment.
The following assessments and investigations will be conducted at each visit:
- Check of the patient's current practice for effective contraception
- Recording of adverse events
- Recording of menstrual cycles (non menopausal female patients)
- Query for medical procedures, hospitalizations, changes in concomitant medications and therapies
- Physical examination including vital signs, weight, height (BMI)
- Neurological examination
- Perform MRC, ALSFRS-R and FVC measurement
- Appointment for the next visit

10.9. FOLLOW UP VISIT, MONTH 9

This visit will be performed in one of the recruiting Neurological ALS Centres (Modena, Firenze, Rome, Chieti, Perugia, Siena)
These visits will be performed at month 9.
The following assessments and investigations will be conducted at each visit:
- Check of the patient's current practice for effective contraception
- Recording of adverse events
- Recording of menstrual cycles (non menopausal female patients)
- Query for medical procedures, hospitalizations, changes in concomitant medications and therapies
- Physical examination including vital signs, weight, height (BMI)

- Neurological examination
- Perform MRC, ALSFRS-R and FVC measurement
- Blood sample will be collected for haematology and blood chemistry
- Urinalysis
- Blood sample for biological assessement and peripheral biomarkers
- Appointment for the next visit

10.10 FOLLOW UP VISIT, MONTH 12

This visit will be performed at Policlinico Gemelli in Rome.
This visit will be performed at 12 month from the first treatment.
The following measures should be conducted before treatment:

- Check of the patient's current practice for effective contraception
- Physical examination including vital signs, weight, height (BMI)
- Blood, gut tissue (biopsies) saliva and feces samples collection
- Query for medical procedures, hospitalizations, changes in concomitant medications and therapies

After the procedure the patient will be observed for 3 hours and then he could come back home; the appointment for the next visit will be given (at six months).

10.11 END OF STUDY VISIT (MONTH 12)

This visit will be performed in one of the recruiting Neurological ALS Centres (Modena, Firenze, Rome, Chieti, Perugia, Siena)
This visit will be performed 1 week to 1 month after follow up visit at month 12.
The following assessments and investigations will be conducted at each visit:

- Check of the patient's current practice for effective contraception
- Recording of adverse events
- Recording of menstrual cycles (non menopausal female patients)
- Query for medical procedures, hospitalizations, changes in concomitant medications and therapies
- Physical examination including vital signs, weight, height (BMI)
- Neurological examination
- Perform MRC, ALSFRS-R and FVC measurement
- ALSAQ – 40 (Amyotrophic Lateral Sclerosis Assessment Questionnaire)
- Blood sample will be collected for haematology and blood chemistry
- Urinalysis
- Blood sample for biological assessement and peripheral biomarkers

10.12 WITHDRAWAL OF PATIENTS

Patient may withdraw from the study for any of the following reasons:
- Withdrawal of consent
- Adverse or intercurrent event considered intolerable by the patient or incompatible with continuation of the study according to the investigator
- Protocol violation (e.g., noncompliance with treatment administration, prohibited treatment needed)
- Worsening of the patient's disease status requiring a change in treatment, as determined by the investigator
- Suspected pregnancy or positive pregnancy test result. Any suspected pregnancy must be immediately confirmed by a serum pregnancy test

If the patient discontinues the study, the reason must be given on the study completion form as one of the following:
- Adverse Event
- Patient’s request / Unwillingness to continue
- Lost to follow-up
- Documented progressive disease
- Protocol Deviation
- Death
- Other (to be specified by the investigator)

The investigator should record the main reason for discontinuation of treatment in the eCRF and single out the primary reason if more than one applies.
If a patient discontinues the study for an adverse event, s/he must be followed up weekly for 4 weeks, or until resolution or stabilization of the event, whichever comes first.
In case of premature withdrawal, the patient will undergo all examinations scheduled for the last trial visit.
A final visit will be performed within 2 weeks of last study treatment intake on patients who are withdrawn from the study prematurely, at any time after receiving the first dose of study medication.

11. STATISTICAL METHODS

11.1. SAMPLE SIZE JUSTIFICATION

The sample size has been estimated considering as the primary outcome measure the proportion of positive response (Tregs number increase) at treatment end (6 MONTHS) in pts treated with FMT vs controls.

ALS pts have a slight reduction of Treg% (mean±SD:2.1±0.7) with respect to healthy controls (2.6±0.6)(Treg% calculated on total lymphocytes; normal values of total lymphocytes: 1000-4500/mmc; normal values of total Treg: 71.5±17/mmc) (Mantovani et al., 2009).

Via Giardini, 1355
Baggiovara
41126 Modena
tel. : 059396111
fax.: 0593962409

Slowly progressive ALS pts have a number of Tregs that is equal to healthy controls, whereas fast progressors have 31% fewer Tregs than slowly progressing pts, and Treg % is inversely correlated with the rate of disease progression(Beers,2011). These data indicates that ALS pts have 60±17 Treg/mmc (fast progressors: 49.3Treg/mmc; slow progressors: 71.5Treg/mmc). As a result, a "positive response" can be considered an increases of the proportion of Tregs by at least 20%. The null hypothesis is that FMT does not increase significantly the proportion of positive responses in treated pts after the second FMT round compared to their baseline stage and to control group. The alternative hypothesis is that FMT determines a proportion of positive responses in at least 50% of treated patients compared to a maximum 5% of patients in the control group. The study has been designed to reject the null hypothesis with an alpha error of 0.05 (in order to take into account a multiple comparison with a control arm) and a power of 0.80. For this purpose, a sample of 39 pts randomized in 2 treatment arms would be needed. Considering an average drop out of 10% then a recruitment of 42 patients will be necessary.

11.2 STATISTICAL ANALYSIS

Data management and statistical plan: Unit 7 will be in charge for this work (details in WP4). An independent Data and Safety Monitoring Committee will be established with several meetings aimed at preserving safety (see safety evaluation box).

Statistical methods:
Separate analyses will be performed in:
1. All randomized subjects receiving at least 1 FMT (**Intention-to-treat population**);
2. All randomized subjects excluding protocol deviations (**Per protocol, PP population**).

Descriptive statistics will be performed comparing the groups of FMT treatment and controls. Continuous variables will be described using mean and S.D. or median and I.Q. range; categorical variables will be described as counts and percentages.

Biological activity: Immune response to FMT will be analyzed as the difference in positive response to FMT (mean Tregs increase >20%) between the control group and the FMT groups. This will be calculated with the use of Treg data obtained at baseline and at month 6.
We will compare the mean values of of different T, B, NK cell subpopulations, of biomarkers, inflammasome, cytokines, comparing baseline and treatment end (month 6) between FMT and control groups. Mean differences in plasma concentrations from baseline to month 6 in the 2 treatment arms will be calculated and compared using t-test or Wilcoxon-Mann-Whitney test.
The mean change over time for the same variables as above will be assessed using repeated measures ANOVA, with treatment as between-subjects factor and time as within-subjects factor. Different models will be used, each with a different biomarker of activity as the dependent variable. Models will be adjusted for any unbalanced distribution of the main prognostic factors (e.g. age) between the two treatment arms.

Analysis of microbiota will be performed with specific softwares.

Safety analysis will be performed in all subjects receiving at least one FMT round. All AEs and SAEs will be recorded according to ICH Guidelines and compared in the treatment arms at any follow-up visit and at study end.
Differences in tracheostomy-free survival (Kaplan-Meier method) between the treated groups and control group will be compared using the log-rank test. Cox's proportional hazard model would be used to adjust for any possible unbalanced prognostic factors. Statistical significance will be set at 0.05 level for a two-tailed test. Missing data will be handled using the last observation carried forward.

FMT efficacy: The comparison of clinical endpoint among arms will be carried out by using the logistic regression model. Results will be presented as odds ratios (OR) and 95%C.I.. FMT will be considered effective in relation to controls whether the OR of positive results will provide a p-value<0.05.

11.3 STOPPING RULES FOR SAFETY REASONS

Adverse events
Adverse Events (AE) (serious and non serious AE-SAE and NSAE), Adverse Reaction (AR), Unexpected AR, will be defined accordingly to according to Italian Regulation (D.Lgs. 191/2007, D.Lgs 16/2010)
All AE occurring between the first study-related procedure and the last study-related procedure will be reported. Those meeting the definition of SAE will be reported in an ad hoc SAE Form; they will be reported to coordinating centre within 24 hours. All AE will be recorded in the eCRF with a diagnosis (whenever possible), and together with investigator’s opinion concerning the relationship of the AE to study treatment. The coordinating Centre will be responsible for appropriate AE reporting to the regulatory authorities (CNT) according to Italian Regulation (D.Lgs. 191/2007, D.Lgs 16/2010); investigators will be responsible for reporting to appropriate Ethic Committee. In case of death a clinical report will be prepared by the caring investigator together with SAE form; in case of autopsy, autopsy report will be added to study documentation. Safety Data Collection is described below in the data collection section.

Stopping rules for safety reasons:
To establish stopping rules, we will consider the following list of toxicities as acceptable: diarrhea, abdominal cramping and pain, nausea, vomiting, flatulence, fever, constipation, dizziness, sore throat, rhinhorrea, bloating, nasal stuffiness, urinary infections, headache, mild/moderate blood cells alterations; elevated CRP, rashes, urticaria, dermatoses, dermatitis (mild/moderate severity), elevated AST/ALT/ALP, loss of appetite, colitis/gastroenteritis.
A procedure consisting of stopping the trial in case of excess of AE recording the treating group in case of 30% of the occurrence of the following: peritonitis, upper gastrointestinal hemorrhage, sepsis, bacteriemia.

Interim analysis
Given the short duration of the study, an interim analysis of efficacy data is not scheduled. Nevertheless, to address safety concerns, a report including all relevant clinical data of patients will be sent by the Statistics Unit to an independent Data and Safety Monitoring Board for scheduled

DSMB meetings (also through Skype) when 14 patients would have completed the second treatment and then every 6 months.

12. DATA RECORDING AND STUDY MONITORING

All data will be recorded by an electronic CRF.
The study will be monitored by a certified contract research organization (CRO).
DSMB meetings will be scheduled when 14 patients would have completed the second treatment and then every 6 months.
An IEAB will sent a report to the EC within 2 months from actual study start and at study end.

13. DATA MANAGEMENT

Data from the CRFs are entered into the study database using EDC (Electronic Data Capture).
Subsequently, the information entered into the database is systematically checked:

- On line with automatic checks
- Off line by Data Management staff (Statistics Unit based at University of Modena)

using error messages from validation programs or database listings.
Error message will be entered on Data Clarification Forms and entered into the data-base by the investigator using EDC. Investigator will sign the final eCRF with electronic signature. This process follows until the lock of the data-base. Quality control audits of all key safety and efficacy data in the database will be made by the data-manager before locking the data-base.
Concomitant medications entered into the database will be coded using the WHO Drug Reference List which employs the Anatomical Therapeutic Chemical classification system. Coexistent diseases and adverse events will be coded using MedDRA.
When the database has been declared to be complete and accurate, the database will be locked.

14.ETHICS

14.1 ETHICAL CONSIDERATIONS

The study will be carried out in accordance with the Declaration of Helsinki (Appendix 17.2), as amended by the 64th WMA General Assembly, Fortaleza, Brazil, October 2013.

14.2 SUBJECT INFORMATION AND INFORMED CONSENT

All subjects must sign and personally date an approved Informed Consent Form after receiving detailed written and verbal information about the reason, the nature, the required procedures, the intended duration and the possible risks and benefits and any discomfort associated with the study. He/She should be informed that the subject's participation in the study is voluntary and that he/she may refuse to participate or withdraw from the trial, at any time, without penalty or loss of benefits to which the subject is otherwise entitled. The language used in the oral and written information

Via Giardini, 1355
Baggiovara
41126 Modena
tel. : 059396111
fax.: 0593962409

about the trial, including the written Informed Consent Form (ICF), should be as non technical as practical and should be understandable to the subject or the subject's legally acceptable representative and the impartial witness, where applicable. The subject must be given ample time to read and to understand the Patient Information Sheet and opportunity to inquire and ask any clarification about the trial before signing the Informed Consent Form.
The patient information sheet will be given to the patient at least 2 weeks befor he can sign the ICF. In the patient information sheet the patient will find a telephone number of the IEAB, that he/she could call to recive further and independent information. The same day when the investigator will supply the patient information sheet, the investigator will advise the IEAB by email of a possible enrollment, to allow iEAB members to be present at the moment of the ICF collection.
The written Informed Consent Form should be signed and personally dated by the subject or by the subject's legally acceptable representative, by the physician who conducted the informed consent discussion, and by a neurologist who does not cure the patient. When applicable the Investigator may conduct the informed consent discussion in presence of an impartial witness of the IEAB, who should sign and personally date the Informed Consent Form too.
No study procedure can be performed before the written informed consent has been provided.
The informed consent procedure must be done according to the guidelines provided in the Declaration of Helsinki and the ICH E6 Guideline for Good Clinical Practice.
The subject must be made aware and agree that personal information may be scrutinized during audit by competent authorities and properly authorized persons. However, personal information will be treated as strictly confidential and will not be publicly available.
By signing the Investigator Statement (Appendix) the Investigator assures the Sponsor that Informed Consent will be obtained.
Both the Patient Information Sheet and the Informed Consent Form must be approved by the Ethics Committee with the study protocol.

14.3 PATIENT WITHDRAWALS AND DROPOUTS

Any study subject may be withdrawn from the study at any time either at the discretion of the investigator or at the request of the study subject.
The reason for doing so should be clearly documented in the CRF.
However, the study subject is not under any obligation to provide a reason for withdrawal.

14.4 ETHIC COMMITTEE APPROVAL

The protocol, Subject Information Sheet, Informed Consent Form and any advertisement for the recruitment of subjects must be reviewed and approved by an appropriately constituted Ethic Committee, as required in chapter 3 of the ICH E6 Guideline.
Written EC approval must be obtained by the Sponsor prior to shipment of study agent or subject enrollment.
The Investigator is committed in accordance with local requirements to inform the IRB/IEC of any emergent problem, serious adverse events, and/or protocol amendments.

Via Giardini, 1355
Baggiovara
41126 Modena
tel. : 059396111
fax.: 0593962409

14.5 INDEPENDENT BOARDS

An Independent Data and Safety Monitoring Board (DSMB) without direct involvement in the conduct of the study, will be set up and notified to the Ethical Committee of the coordinating centre specifically to monitor safety data throughout the duration of the study to determine if continuation is appropriate both scientifically and ethically. All adverse events occurring during the trial will be forwarded to the Committee.
An independent DSMB will be established, to address ethics concerns that could arise during the study, with particular attention to informed consent form. The IEAB will send to the Ethics Committee a report within 2 months from study actual starting date and another report at the end of the study.

15. ADMINISTRATIVE CONSIDERATIONS

Regulatory Requirements–Sponsor/Investigator Obligations
This study will be conducted in accordance with the Declaration of Helsinki and the ICH E6 Guideline (Good Clinical Practice). To ensure compliance the Investigator agrees, by written consent to this protocol, to fully cooperate with compliance checks by allowing access to all documentation, including patient hospital files (the source documents), by authorised individuals and competent authorities.

Protocol Amendments
Protocol directions must be strictly adhered to. In case of need a written Protocol Amendment will be prepared. The Amendment will be approved and signed by the involved parties (Investigator and Sponsor) according to the same procedure followed for the study protocol.
The Amendment will not be implemented before the approval of the Ethics Committee.

Curriculum Vitae
The Investigator and any co-Investigator(s) must provide the coordinating centre with current copies of their own curriculum vitae.

Investigator's Statement
This document, signed and dated by the Principal Investigator, describes the Investigator's obligations.

Via Giardini, 1355
Baggiovara
41126 Modena
tel. : 059396111
fax.: 0593962409

Role of participating centres

Each centre is expected:

1. to randomize at least 7 patients fulfilling including and excluding criteria in a period of 18 months;
2. to provide one principal investigator to evaluate including and excluding criteria, and assess primary and secondary outcomes.
3. to formally adhere to the practice parameters of the European Federation of Neurological Societes concerning the standardization of the management of the patient in terms of ventilatory support and nutrition.

Monitoring procedures

Study monitoring

The study monitor indicated by the coordinating centre will be in contact with the investigators and will conduct a visit to each Centre to discuss and/or collect data. The Monitor will conduct a visit before the start of the study to discuss the protocol and obligations of the investigators and sponsor. Investigators of each centre are required to allow the Monitor to conduct the site visit, the study-end visit and the site closure visit.

The purposes of these visits are:

– Make sure that the written informed consent was obtained for each subject before participation in the study
– Assess the progress of the study.
– Ensure compliance with the study protocol.
– Check whether all adverse events were correct relationship.
– Make sure that the investigator retains the essential documents of the study.
– To discuss any problem that arises.
– Review the CRF for readability, accuracy and completeness of the compilation.
– Validating Data entered in the CRF versus the source files.

The investigators are required to make available for inspection the original medical documents. Information contained therein will be treated as confidential.

The Monitor will conduct a final audit to close the centers to the end of the study.

Electronic Case Report Form (eCRF)

All information about the study will be collected in the eCRF.

If requested, copies of the eCRF are to be made available to the appropriate regulatory agencies.

Auditing

The Investigators will make all pertinent records available including original medical documents for inspection by regulatory authorities. This information will be considered as confidential.

Archiving of Records

Copies of the protocol, subject identification codes, electronic Case Report Form, source data, Informed Consent Form and other documents pertaining to the study conduction and support the data collected from each subject must be kept for the maximum period of time as required by the study centres.

No study document should be destroyed.

Via Giardini, 1355
Baggiovara
41126 Modena
tel. : 059396111
fax.: 0593962409

Originals of all documentation and copies of outgoing correspondence concerning the study will be stored and retained by the Sponsor in a safe area in the Trial Master File for the lifetime of the product. In particular, the final report must be retained by the Sponsor, or the subsequent owner, for five years beyond the lifetime of the study agent.

Role of funding

This is an academic, independent clinical research project funded by Minister of Health (MoH).
Participating units and patients will not be paid.

Use and Publication of Study Results

The results of the study may be presented during scientific symposia or published in scientific journals only after review and written approval by the involved parties in full respect of the privacy of the participating subjects. None of the investigators at the participating centres can make use of any information or data before, during and after the study without the written approval from the principal investigator (Dr. Jessica Mandrioli).

Insurance Policy

An insurance company will provide insurance coverage for damages to patients involved in the trial. The principal Investigator will be supplied with all data concerning the insurance company and policy number.

16. REFERENCES

ALSUntangled Group. ALS Untangled No. 21: Fecal transplants. ALS FTD 2013;14:482-5.
Amedei A., C. Della Bella, E. Niccolai, N. Stanflin, M. Benagiano, R. Duranti, G. Del Prete, TF. Murphy, M.M. D'Elios. Moraxella catarrhalis - specific th1 cells in bal fluids of copd patients. Int J Immunopathol Pharmacol. 2009; 22(4):979-90
Amedei A, Bergman MP, Appelmelk BJ, Azzurri A, Benagiano M, Tamburini C, et al. Molecular mimicry between Helicobacter pylori antigens and H+, K+ --adenosine triphosphatase in human gastric autoimmunity. J Exp Med 2003;198:1147-56.
Amedei A, Niccolai E, Benagiano M, Della Bella C, Cianchi F, Bechi P, et al. Ex vivo analysis of pancreatic cancer-infiltrating T lymphocytes reveals that ENO-specific Tregs accumulate in tumor tissue and inhibit Th1/Th17 effector cell functions. Cancer Immunol Immunother 2013;62:1249-60. doi: 10.1007/s00262-013-1429-3.
Backhed F., Ley R.E., Sonnenburg J.L., Peterson D.A., Gordon J.I. Hostbacterial mutualism in the human intestine. Science 2005;307:1915-20.
Baxter M. & Colville A. Adverse events in faecal microbiota transplant: a review of the literature. J. Hosp. Infect. 2016;92:117-127. doi: 10.1016/j.jhin.2015.10.024

Via Giardini, 1355
Baggiovara
41126 Modena
tel. : 059396111
fax.: 0593962409

Beers D.R., Henkel J.S., Zhao W., et al. Endogenous regulatory T lymphocytes ameliorate amyotrophic lateral sclerosis in mice and correlate with disease progression in patients with amyotrophic lateral sclerosis. Brain. 2011;134(5):1293-314. doi: 10.1093/brain/awr074.

Bienenstock J., Kunze W., Forsythe P. Microbiota and the gut-brain axis. Nutr. Rev. 2015;73(Suppl. 1): 28-31. doi: 10.1093/nutrit/nuv019

Boillée S., Yamanaka K., Lobsiger C.S., et al. Onset and progression in inherited ALS determined by motor neurons and microglia.Science 2006;312(5778):1389-92.

Boillée S, VandeVelde C, Cleveland DW. ALS: a disease of motor neurons and their non neuronal neighbours. Neuron. 2006;52:39–59. (b)

Borody TJ, Khoruts A. Fecal microbiota transplantation and emerging applications. Nat Rev Gastroenterol Hepatol 2011;9(2):88-96. doi: 10.1038/nrgastro.2011.244

Bradley W.G., Borenstein A.R., Nelson L.M., et al. Is exposure to cyanobacteria an environmental risk factor for amyotrophic lateral sclerosis and other neurodegenerative diseases? Amyotroph Lateral Scler Frontotemporal Degener. 2013 Sep;14(5-6):325-33.

Brandt L.J. Fecal microbiota transplant: respice, adspice, prospice. J. Clin. Gastroenterol. 2015; 49(Suppl 1): 65–68.

Brandt L.J., Brandt L.J., Aroniadis O.C., et al. Long-term follow-up of colonoscopic fecal microbiota transplant for recurrent Clostridium difficile infection. Am.J. Gastroenterol. 2012;107(7):1079-87.

Braniste V, Al-Asmakh M, Kowal C, Anuar F, Abbaspour A, Tóth M, Korecka A, Bakocevic N, Ng LG, Kundu P, Gulyás B, Halldin C, Hultenby K, Nilsson H, Hebert H, Volpe BT, Diamond B, Pettersson S. The gut microbiota influences blood-brain barrier permeability in mice. Sci Transl Med. 2014 Nov 19;6(263):263ra158

Cammarota G., Masucci L., Ianiro G., et al. Randomised clinical trial: faecal microbiota transplantation by colonoscopy vs. vancomycin for the treatment of recurrent Clostridium difficile infection. Aliment Pharmacol Ther 2015;41(9):835-43. doi: 10.1111/apt.13144.

Conway PL. Development of intestinal microbiota. In: Mackie R.I.,White B.A., Isaacson R.E., editors. Gastrointestinal microbiology. Vol.2 New York: Chapman & Hall Microbiology Series;1997:3-38.

Eckburg P.B., Bik E.M., Bernstein C.N., et al. Diversity of the human intestinal microbial flora. Science 2005;308:1635-8.

Eiseman B., Silen W., Bascom G.S., Kauvar A.J. Fecal enema as an adjunct in the treatment of pseudomembranous enterocolitis. Surgery 1958;44(5):854–859.

Erny D., Hrabě de Angelis A.L., Jaitin D., et al. Host microbiota constantly control maturation and function of microglia in the CNS. Nat Neurosci 2015;18(7):965-77.

Fang X. Potential role of gut microbiota and tissue barriers in Parkinson's disease and amyotrophic lateral sclerosis. Int J Neurosci 2016;126:771–6. doi:10.3109/00207454.2015.1096271.

Fang X, Wang X, Yang S, Meng F, Wang X, Wei H, et al. Evaluation of the microbial diversity in amyotrophic lateral sclerosis using high-throughput sequencing. Front Microbiol 2016;7:1479. doi:10.3389/fmicb.2016.01479

Frank D.N., St Amand A.L., Feldman R.A., Boedeker E.C., Harpaz N., Pace N.R. Molecular-phylogenetic characterization of microbial community imbalances in human inflammatory bowel diseases PNAS 2007;104(34):13780-5.

Fung TC, Olson CA, Hsiao EY. Interactions between the microbiota, immune and nervous systems in health and disease. Nat Neurosci 2017; 20:145–155. https ://doi.org/10.1038/nn.4476.

Furness JB. The enteric nervous system and neurogastroenterology. Nat Rev Gastroenterol Hepatol

2012; 9:286–294. https ://doi.org/10.1038/nrgas tro.2012.32
Gopalakrishnan V, Spencer CN, Nezi L, Reuben A, Andrews MC, Karpinets TV, et al. Gut microbiome modulates response to anti-PD-1 immunotherapy in melanoma patients. Science 2018; 359:97–103. doi:10.1126/science.aan4236
Graves CL, Li J, LaPato M, Shapiro MR, Glover SC, Wallet MA, Wallet SM. Intestinal Epithelial Cell Regulation of Adaptive Immune Dysfunction in Human Type 1 Diabetes. Front Immunol. 2017;7:679. doi: 10.3389/fimmu.2016.00679.
Hardiman O., van den Berg LH. Edaravone: a new treatment for ALS on the horizon? Lancet Neurol. 2017 Jul;16(7):490-491.
Henkel J.S., Beers D.R., Wen S., et al. Regulatory T-lymphocytes mediate amyotrophic lateral sclerosis progression and survival. EMBO Mol Med. 2013;5(1):64-79
Huisman MB, Seelen M, Van Doormaal PC. Effect of presymptomatic body mass index and consumption of fat and alcohol on amyotrophic lateral scle-rosis. JAMA Neurol 2015; 72:1155–62. doi:10.1001/jamaneurol.2015.1584
Kassam Z., Lee C.H., Yuan Y., Hunt R.H. Fecal microbiota transplantation for Clostridium difficile infection: systematic review and meta-analysis. Am. J. Gastroenterol. 2013;108:500–508. doi: 10.1038/ajg.2013.59. Epub 2013 Mar 19.
Kelly CR, Kahn S, Kashyap P, Laine L, Rubin D, Atreja A, Moore T, Wu G. Update on Fecal Microbiota Transplantation 2015: Indications, Methodologies, Mechanisms, and Outlook. Gastroenterology 2015;149:223-37. doi: 10.1053/j.gastro.2015.05.008.
Kelly C.R., Ihunnah C., Fischer M. et al. Fecal microbiota transplant for treatment of Clostridium difficile infection in immunocompromised patients. Am. J.Gastroenterol. 2014;109:1065-71
Khanna S. Microbiota Replacement Therapies: Innovation in Gastrointestinal Care. Clin Pharmacol Ther. 2018 Jan;103(1):102-111. doi: 10.1002/cpt.923.
Kimura F, Fujimura C, Ishida S, et al. Progression rate of ALSFRS-R at time of diagnosis predicts survival time in ALS. Neurology 2006;66:265-7.
Kadowaki A., Miyake S., Saga R., Chiba A., Mochizuki H., Yamamura T. Gut environment-induced intraepithelial autoreactive CD4(+) T cells suppress central nervous system autoimmunity via LAG-3. Nat Commun 2016;7:11639.
Lee Y.K., Menezes J.S., Umesaki Y., Mazmanian S.K. Proinflammatory T-cell responses to gut microbiota promote experimental autoimmune encephalomyelitis. PNAS 2011;108(1):4615-22.
Li G, Yang M, Jin Y, Li Y, Qian W, Xiong H, Song J, Hou X. Involvement of shared mucosal-associated microbiota in the duodenum and rectum in diarrhea-predominant irritable bowel syndrome. J Gastroenterol Hepatol. 2018;33:1220-1226. doi: 10.1111/jgh.14059.
Longstreth W.T. Jr, Meschke J.S., Davidson S.K., Smoot L.M., Smoot J.C., Koepsell T.D. Hypothesis: a motor neuron toxin produced by a clostridial species residing in gut causes ALS. Med Hypotheses. 2005;64(6):1153-6.
Louveau A, Smirnov I, Keyes TJ, Eccles JD, Rouhani SJ, Peske JD et al. Structural and functional features of central nervous system lymphatic vessels. Nature 2015; 523:337–341. https ://doi. org/10.1038/natur e1443 2
Mantovani S., Garbelli S., Pasini A., et al. Immune system alterations in sporadic amyotrophic lateral sclerosis patients suggest an ongoing neuroinflammatory process. J Neuroimmunol 2009;210:73-9. doi: 10.1016/j.jneuroim.2009.02.012.

Matson V, Fessler J, Bao R, Chongsuwat T, Zha Y, Alegre M-L, et al. The commensal microbiome is associated with anti-PD-1 efficacy in metastatic melanoma patients. Science 2018;359:104–8. doi:10.1126/science.aao3290

Mayer E. A., Tillisch K., Gupta A. Gut/brain axis and the microbiota. J. Clin. Invest. 2015;125:926-938. doi:10.1172/JCI76304

Maynard CL, Elson CO, Hatton RD, Weaver CT. Reciprocal interactions of the intestinal microbiota and immune system.Nature 2012;489:231–241. https ://doi.org/10.1038/natur e1155 1

Moreno-Igoa M., Calvo A.C., Penas C., et al. Fragment C of tetanus toxin, more than a carrier. Novel perspectives in non-viral ALS gene therapy. J Mol Med (Berl). 2010 Mar;88(3):297-308.

Nayak D, Roth TL, McGavern DB. Microglia development and function. Annu Rev Immunol 2014;32:367–402. https ://doi. org/10.1146/annur ev-immun ol-03271 3-12024 0

Pecere S., Sabatelli M., Fantoni M., Ianiro G., Gasbarrini A., Cammarota G. Letter: faecal microbiota transplantation in combination with fidaxomicin to treat severe complicated recurrent Clostridium difficile infection. Aliment Pharmacol Ther. 2015;42(8):1030. doi: 10.1111/apt.13362.

Piccione E.A., Sletten D.M., Staff N.P. Low P.A.Autonomic system and ALS. Muscle Nerve 2015;51(5):676-9.

Quigley EMM. Microbiota-brain-gut axis and neurodegenerative diseases. Curr Neurol Neurosci Rep 2017; 17:94. https ://doi.org/10.1007/s1191 0-017-0802-6.

Rooks MG, Garrett WS. Gut microbiota, metabolites and host immunity. Nat Rev Immunol 2016; 16:341–352. https ://doi.org/10.1038/nri.2016.42

Round J.L., Mazmanian S.K. Inducible Foxp3+ regulatory T-cell development by a commensal bacterium of the intestinal microbiota.PNAS USA 2010;107(27):12204-9.

Routy B, Le Chatelier E, Derosa L, Duong CPM, Alou MT, Daillère R, et al. Gut microbiome influences efficacy of PD-1-based immunotherapy against epithelial tumors. Science 2018;359:91–7. doi:10.1126/science. aan3706

Rowin J, Xia Y, Jung B, Sun J. Gut inflammation and dysbiosis in human motor neuron disease. Physiol Rep 2017;5:e13443. doi:10.14814/phy2.13443

Sabel CE, Boyle PJ, Loytonen M, et al. Spatial clustering of amyotrophic lateral sclerosis in Finland at place of birth and place of death. Am J Epidemiol. 2003;157:898–905

Sampson T.R., Debelius J.W., Thron T., et al. Gut microbiota regulate motor deficits and neuroinflammation in a model of Parkinson's disease. Cell 2016;167:1469-1480.e12.

Saresella M., Piancone F., Tortorella P., et al. T helper-17 activation dominates the immunologic milieu of both amyotrophic lateral sclerosis and progressive multiple sclerosis. Clin Immunol. 2013;148(1):79-88. doi: 10.1016/j.clim.2013.04.010.

Scheperjans F. Can microbiota research change our understanding of neurodegenerative diseases? Neurodegener Dis Manag. 2016; 6(2):81-5.

Scheperjans F., Aho V., Pereira P.A., et al. Gut microbiota are related to Parkinson's disease and clinical phenotype. Mov Disord 2015;30(3):350–358.

Schwan A., Sjolin S., Trottestam U., Aronsson, B. Relapsing clostridium difficile enterocolitis cured by rectal infusion of homologous faeces. Lancet 1983;2 (8354):845.

Shi N, Li N, Duan X, Niu H. Interaction between the gut microbiome and mucosal immune system. Mil Med Res 2017; 4:14. https ://doi.org/10.1186/s4077 9-017-0122-9.

Via Giardini, 1355
Baggiovara
41126 Modena
tel. : 059396111
fax.: 0593962409

Smith PM, Howitt MR, Panikov N, Michaud M, Gallini CA,Bohlooly YM et al. The microbial metabolites, shortchain fatty acids, regulate colonic Treg cell homeostasis. Science 2013;341:569–573. https ://doi.org/10.1126/science.12411 65

Tiemessen M.M., Jagger A.L., Evans H.G., van Herwijnen M.J., John S., Taams L.S. CD4+CD25+Foxp3+ regulatory T cells induce alternative activation of human monocytes/macrophages. PNAS 2007;104(49):19446-51.

Tremlett H., Bauer K.C., Appel-Cresswell S., Finlay B.B., Waubant E. The gut microbiome in human neurological disease: A review. Ann Neurol. 2017 Mar;81(3):369-382. doi: 10.1002/ana.24901.

Tremlett H, Fadrosh DW, Faruqi AA, et al. Gut microbiota composition and relapse risk in pediatric MS: a pilot study. J Neurol Sci 2016;363:153–157.

van Es M.A., Hardiman O., Chio A., Al-Chalabi A., Pasterkamp RJ., Veldink JH., van den Berg LH. Amyotrophic lateral sclerosis. Lancet. 2017 Nov 4;390(10107):2084-2098).

van Nood E, Vrieze A, Nieuwdorp M, et al. Duodenal infusion of donor feces for recurrent Clostridium difficile. N Engl J Med. 2013 Jan 31;368(5):407-15. doi: 10.1056/NEJMoa1205037.

Wang, S., Xu M., Wang W., et al. Systematic review: adverse events of fecal microbiota transplantation. PLoS One 2016;11(8): e0161174

Wu HJ, Wu E. The role of gut microbiota in immune homeostasis and autoimmunity. Gut microbes 2012; 3:4–14. https ://doi.org/10.4161/gmic.19320

Wu S, Yi J, Zhang YG, Zhou J, Sun J. Leaky intestine and impaired microbiome in an amyotrophic lateral sclerosis mouse model. Physiol Rep 2015;3(4). doi:10.14814/phy2.12356

Zhang F, Luo W, Shi Y, Fan Z, Ji G. Should we standardize the 1,700-year-old fecal microbiota transplantation? Am. J. Gastroenterol. 2012;107, 1755–1756. doi: 10.1038/ajg.2012.251

Zhang YG, Wu S, Yi J, Xia Y, Jin D, Zhou J, et al. Target intestinal microbiota to alleviate disease progression in amyotrophic lateral sclerosis. Clin Ther 2017;39:322–36. doi:10.1016/j.clinthera.2016.12.014

Zhao W, Xie W, Xiao Q, Beers DR, Appel SH. Protective effects of an anti-inflammatory cytokine, interleukin-4, on motoneuron toxicity induced by activated microglia. J Neurochem. 2006 Nov;99(4):1176-87.

17. APPENDIXES

17.1. ADVERSE EVENTS

17.1.1. DEFINITION OF ADVERSE EVENT

An adverse event (AE) is defined as any untoward medical occurrence in a patient or subject which does not necessarily have a causal relationship with any medical treatment. An AE can therefore be any unfavourable and unintended sign (e.g., an abnormal laboratory finding), symptom or disease temporally associated with a treatment, whether or not considered causally related to the treatment. This includes all intercurrent diseases (newly diagnosed concomitant diseases or symptoms), accidents, clinically relevant deteriorations of pre-existing diseases or clinically relevant deteriorations in clinically evaluated variables (e.g., laboratory, ECG, or physical examination). An event does not have to be documented as an AE in the CRF if the following holds true:

- Untoward medical findings that occur prior to the treatment are not considered to be AEs if they occur in the scope of investigations that are performed to check the inclusion or exclusion criteria
- Deviations in clinically evaluated variables are not considered to be AEs if similar deviations were already present prior to or at the baseline visit. These values should be reported as baseline conditions, if clinically relevant, and exclusion criteria must be obeyed
- Surgeries and other invasive procedures that are planned prior to the start of the study do not have to be documented as AEs. Planned procedures will be recorded in the CRF by the investigator at the baseline visit

17.1.2. DEFINITION OF SERIOUS ADVERSE EVENT

Serious adverse events (SAEs) are a subgroup of all AEs which fulfil internationally agreed upon definitions. They require special attention by the investigator and sponsor.
A SAE is defined in the legislation as 'any undesired and unexpected occurrence associated with any stage of the chain from donation to transplantation (supply, control, processing, storing, distribution of human cells or tissues):

- that might lead to the transmission of a communicable disease;
- that might lead to death or life-threatening, disabling or incapacitating conditions for patients; or
- which results in, or prolongs, hospitalisation or morbidity.

If there is any doubt about the seriousness of an AE, the investigator should contact the study coordinator.

17.1.3. DEFINITION OF SERIOUS ADVERSE REACTION

A serious adverse reaction (SAR) is defined in the legislation as 'an unintended response, including a communicable disease, in the living donor or in the recipient that might be associated with any stage of the chain from donation to transplantation (supply, control, processing, storing, distribution of human cells or tissues):

- that is fatal, life-threatening, disabling, incapacitating, or
- which results in, or prolongs, hospitalisation or morbidity.

17.1.4. REPORTING OF ADVERSE EVENTS

Information about all adverse events, whether volunteered by the patient, discovered through questioning by the investigator, or detected through physical examination, laboratory test or other means, will be collected and recorded on the "Adverse Event Case Report Form" and followed as appropriate.
As far as possible, each AE will also be described by:
Its duration (start and end dates)
Its severity
Its relationship to the study treatment (suspected/not suspected/not assessable)
The action(s) taken
Any AE occurring by the time of study completion (within two weeks of last treatment) must be recorded on the AE CRF page.

17.1.5. REPORTING OF SERIOUS ADVERSE EVENTS/REACTIONS

In accordance with the legislative requirements (D.Lgs. 191/2007 e dal D.Lgs 16/2010) Centres are requested to report immediately from their awareness any SAE or SAR occurring during the trial, in particular:
- SAE(s) that may influence the quality and safety of transplantation and is attributable to the donation, testing, characterisation, procurement, preservation, transport or transplantation activities;
- SAR(s) observed during or after transplantation which may be attributed to such activities;
- Living donor reactions – any SAR(s) in the living donor that may result from the donation.

A report form will be available on the online CRF.
Completed forms should be forwarded by email to Italian National Transplantation Centre (CNT) (cnt@iss.it, letizia.lombardini@iss.it e cristina.pintus@iss.it) and to the coordinating centre and principal investigator (j.mandrioli@ausl.mo.it)

SAR/E reporting

The date and time of occurrence of the SAR/E and the date and time of SAR/E detection will be supplied. These times are not necessarily the same in all cases. It is possible that some time may have elapsed before the SAR/E was detected. Distinguishing between the two allows for a further characterisation of the type of event/reaction to be investigated and facilitates the development of risk/cause analysis.

The SAR/E may have implications for other recipients/potential recipients. For example, complications with one donor may have implications for many potential recipients. Details as to whether fresh/frozen feces obtained from this donor had been transplanted to other recipients will be provided to allow for notification to other sites of any potential risks that may be associated with

Via Giardini, 1355
Baggiovara
41126 Modena
tel. : 059396111
fax.: 0593962409

this donor. Policlinico Gemelli in Rome will notify relevant parties and competent authorities as appropriate.

All available information will be included on the form. A unique report ID number to support traceability at the reporting establishment will be assigned;

Donor/Recipient details

Fresh/frozen feces procured, allocated and transplanted will be fully traceable from donor to the recipient and vice versa in order to safeguard the health of both the donors and recipients. In order to facilitate traceability throughout the entire transplantation chain, a number of identification details will be provided, as applicable, to the CNT when submitting SAR/E reports.

Serious Adverse Reactions (SAR) details

SARs are unintended responses or effects in a recipient or living donor to anything that occurs in the transplantation chain. Unintended responses, in this context, entail any reaction by any patient during the chain that may not be considered by the professional medical community as 'calculated risk'.

Clinical symptoms or situations suggesting that any of the following reactions might have occurred in a recipient should be seen as triggers for an SAR report. When completing the form, the reporter(s) will be asked to select from a number of options listed below:

- Infection possibly transferred from the donor to the recipient (e.g. viral, bacterial, prion) that was not known at the time of transplantation (e.g. due to incorrect donor characterization information).
- Infections possibly transferred due to contamination or cross-contamination by an infectious agent on the feces or associated materials from procurement to transplantation.
- Malignant disease possibly transferred from the donor to the recipient that was not known at the time of transplantation.
- Any unintended consequence for the recipient, including recurrence of recipient illness, early graft failure or delayed graft function.
- Unexpected immunological reactions that were not anticipated and may be connected to the testing, characterization, procurement, preservation and transport of feces.

Serious adverse reactions in living donors

Donor adverse reactions with a possible direct effect on the quality and safety of the donated feces, or that may have resulted from the donation must be reported as a serious adverse reaction.
These may be immediate, i.e. occurring at the time of donation or within a few days post-donation, or they may be delayed, i.e. identified after the donation. Suspected SAR must be reported the within 24 hours of its discovery.

Via Giardini, 1355
Baggiovara
41126 Modena
tel. : 059396111
fax.: 0593962409

Serious Adverse Events (SAE) details

A brief description of the type of event is to be provided. This should include the suspected reasons as to why the event may have occurred and if any corrective or preventative actions have been put in place in order to facilitate and close out the investigation.
An assessment is made based on the likelihood of recurrence using the following scale:

- Rare (difficult to believe it could happen again)
- Unlikely (not expected to happen again)
- Possible (may occur occasionally)
- Likely (expected to happen but not persistent)
- Almost certain (expected to happen on many occasions)

It is important to indicate the stage at which the event occurred (donation, testing, characterisation, procurement, preservation, transport, transplantation or other) and the specifics of the SAE, for example if it was an equipment failure, human error or other.

17.1.6. CAUSALITY ASSESSMENT

The investigator has to assess the causal relation of the AE to the treatment. The investigator should base the assessment of a causal relationship on the following scale:
Not suspected

- There is evidently another explanation for the AE:
- The AE is obviously explained by the patient's disease(s) or
- The AE is in accordance with the effect or adverse effect of a concomitant medication or
- The AE had occurred already prior to treatment in comparable intensity and/or frequency or
- The AE started before the first treatment

Suspected

- If there is a reasonable temporal relationship between the AE and treatment, there are plausible reasons that point to a causal relationship with treatment
- Not assessable
- Due to conflicting medical information and/or patient status, no causal relationship can be stated.

17.1.7. INTENSITY OF ADVERSE EVENTS

Grade refers to the severity of the AE.
For oncology studies, The NCI CTCAE v4.0 (published May 2009) displays Grades 1 through 5 with clinical description of severity for each AE.
For non-oncology study, the severity of adverse event is described as follows:

- Mild adverse event: awareness of event but easily tolerated
- Moderate adverse event: discomfort enough to cause interference with usual activity
- Severe adverse event: inability to carry out usual activity.

17.1.8. ACTIONS TAKEN IN RESPONSE TO AN ADVERSE EVENT

The actions taken in response to an AE are described on a numerical scale, from 1 to 4 that covers the various possibilities. One of these has to be selected:
1 = No action taken
2 = Study treatment reduced
3 = Study treatment temporary interrupted
4 = Study treatment permanently discontinued due to this adverse event

17.1.9. FOLLOW-UP ON ONGOING ADVERSE EVENTS

Patients discontinuing the study with reported AEs that have not yet completely resolved must return for one or more follow-up visit(s).

- Adverse events will be followed-up until their resolution or until stabilization stated by the investigator.
- Particular attention should be given to:
- SAEs/SARs
- Ongoing non-serious AEs likely or definitely related to the treatment according to the investigator's causality assessment
- Ongoing AEs leading to the patient's premature discontinuation
- Any laboratory value or vital signs being beyond the sponsor-defined alert limit

The AE is monitored until either normalization, return to the baseline value or identification of a permanent change. In case of minor AEs a phone call to the patient may be acceptable. If necessary, additional safety investigations and queries will be done during and after this time period.
Follow-up information on the outcome must be recorded on the respective AE page in the CRF or in the data clarification form. All efforts to achieve follow-up information must be documented in the source data. Source data information has to be available upon request.
Follow-up investigations may also be necessary according to the investigator's medical judgment even if the patient has no AE at the end of the study. However, information related to these investigations does not have to be documented in the CRF but must be recorded in the source documentation.

In addition, the study sponsor must send periodic reports on the patient follow-up and on the progress of the clinical trial to the CNT as the competent authority for issuing the authorization of the present protocol.

Via Giardini, 1355
Baggiovara
41126 Modena
tel. : 059396111
fax.: 0593962409

17.1.10. FOLLOW-UP FORM TO BE SENT EVERY SIX MONTHS TO ITALIAN NATIONAL TRANSPLANTATION CENTRE

Centro Nazionale Trapianti

FOLLOW UP PAZIENTI sottoposti a trapianto sperimentale autorizzato dal Centro Nazional Trapianti

Compilare con le informazioni richieste ed allegare una relazione clinica riassuntiva sugli aspetti di sicurezza ed efficacia dei pazienti trattati

Titolo protocollo:
Nome responsabile<br><br>della sperimentazione:
Centro clinico:

Data del presente aggiornamento	
Data approvazione CNT	
Data approvazione Comitato Etico	
Nr. pazienti trattati/nr. pazienti previsti<br><br>(se del caso, specificare quanti con trattamento sperimentale e quanti con trattamento di confronto)	

Via Giardini, 1355
Baggiovara
41126 Modena
tel. : 059396111
fax.: 0593962409

Informazioni sui singoli pazienti sottoposti al trapianto sperimentale

Codice paziente	Data del trapianto	Mese di follow up	Tipo di risposta<br>(completa/parziale/non risposta)	Reazioni avverse gravi*

* Con reazione grave s'intende: una risposta non voluta nel donatore o nel ricevente, compresa una malattia trasmissibile, connessa con l'approvvigionamento o l'applicazione sull'uomo di tessuti o cellule, che provochi la morte, metta in pericolo la vita o produca invalidità o incapacità dell'interessato, o ne produca o prolunghi l'ospedalizzazione o lo stato di malattia.

* Con reazione grave s'intende: una risposta non voluta nel donatore o nel ricevente, compresa una malattia trasmissibile, connessa con l'approvvigionamento o l'applicazione sull'uomo di tessuti o cellule, che provochi la morte, metta in pericolo la vita o produca invalidità o incapacità dell'interessato, o ne produca o prolunghi l'ospedalizzazione o lo stato di malattia.

Informazioni sui singoli pazienti sottoposti al trattamento di confronto (se del caso)

Codice paziente	Data del trapianto	Mese di follow up	Tipo di risposta<br>(completa/parziale/non risposta)	Reazioni avverse gravi*

NOTA:

Inviare le su elencate informazioni a cnt@iss.it , letizia.lombardini@iss.it e cristina.pintus@it insieme ad una relazione clinica riassuntiva sugli aspetti di sicurezza ed efficacia dei pazienti trattati e allegando eventuali pubblicazioni correlate alla sperimentazione clinica.

Via Giardini, 1355
Baggiovara
41126 Modena
tel. : 059396111
fax.: 0593962409

17.2 DECLARATION OF HELSINKI – ETHICAL PRINCIPLES FOR MEDICAL RESEARCH INVOLVING HUMAN SUBJECTS

World Medical Association

Adopted by the 18th World Medical Assembly, Helsinki, Finland, June 1964
and amended by the
29th World Medical Assembly, Tokyo, Japan, October 1975,
35th World Medical Assembly, Venice, Italy, October 1983,
41st World Medical Assembly, Hong Kong, September 1989
and the
48th General Assembly, Somerset West, Republic of South Africa, October 1996
52nd WMA General Assembly, Edinburgh, Scotland, October 2000
53rdWMA General Assembly,Washington, DC, USA, October 2002 (Note of Clarification added)
55th WMA General Assembly, Tokyo, Japan, October 2004 (Note of Clarification added)
59thWMA General Assembly, Seoul, Republic of Korea, October 2008
64th WMA General Assembly, Fortaleza, Brazil, October 2013

Preamble

1. TheWorldMedical Association (WMA) has developed the Declaration of Helsinki as a statement of ethical principles for medical research involving human subjects, including research on identifiable human material and data. The Declaration is intended to be read as a whole and each of its constituent paragraphs should be applied with consideration of all other relevant paragraphs.
2. Consistent with the mandate of theWMA, the Declaration is addressed primarily to physicians. The WMA encourages others who are involved in medical research involving human subjects to adopt these principles.

General Principles

3. The Declaration of Geneva of the WMA binds the physician with the words, “The health of my patient will be my first consideration,” and the International Code of Medical Ethics declares that, “A physician shall act in the patient's best interest when providing medical care.”
4. It is the duty of the physician to promote and safeguard the health, well-being and rights of patients, including those who are involved in medical research. The physician's knowledge and conscience are dedicated to the fulfilment of this duty.
5. Medical progress is based on research that ultimately must include studies involving human subjects.
6. The primary purpose of medical research involving human subjects is to understand the causes, development and effects of diseases and improve preventive, diagnostic and therapeutic interventions (methods, procedures and treatments). Even the best proven interventions must be evaluated continually through research for their safety, effectiveness, efficiency, accessibility and quality.
7. Medical research is subject to ethical standards that promote and ensure respect for all human subjects and protect their health and rights.
8. While the primary purpose of medical research is togenerate new knowledge, this goal can never take precedence over the rights and interests of individual research subjects.

Via Giardini, 1355
Baggiovara
41126 Modena
tel. : 059396111
fax.: 0593962409

9. It is the duty of physicians who are involved in medical research to protect the life, health, dignity, integrity, right to self determination, privacy, and confidentiality of personal information of research subjects. The responsibility for the protection of research subjects must always rest with the physician or other health care professionals and never with the research subjects, even though they have given consent.
10. Physicians must consider the ethical, legal and regulatory norms and standards for research involving human subjects in their own countries as well as applicable international norms and standards. No national or international ethical, legal or regulatory requirement should reduce or eliminate any of the protections for research subjects set forth in this Declaration.
11. Medical research should be conducted in a manner that minimizes possible harm to the environment.
12. Medical research involving human subjects must be conducted only by individuals with the appropriate ethics and scientific education, training and qualifications. Research on patients or healthy volunteers requires the supervision of a competent and appropriately qualified physician or other health care professional.
13. Groups that are underrepresented in medical research should be provided appropriate access to participation in research.
14. Physicians who combine medical research with medical care should involve their patients in research only to the extent that this is justified by its potential preventive, diagnostic or therapeutic value and if the physician has good reason to believe that participation in the research study will not adversely affect the health of the patients who serve as research subjects.
15. Appropriate compensation and treatment for subjects who are harmed as a result of participating in research must be ensured.

Risks, Burdens and Benefits
16. In medical practice and in medical research, most interventions involve risks and burdens. Medical research involving human subjects may only be conducted if the importance of the objective outweighs the risks and burdens to the research subjects.
17. All medical research involving human subjects must be preceded by careful assessment of predictable risks and burdens to the individuals and groups involved in the research in comparison with foreseeable benefits to them and to other individuals or groups affected by the condition under investigation. Measures to minimise the risks must be implemented. The risks must be continuously monitored, assessed and documented by the researcher.
18. Physicians may not be involved in a research study involving human subjects unless they are confident that the risks have been adequately assessed and can be satisfactorily managed. When the risks are found to outweigh the potential benefits or when there is conclusive proof of definitive outcomes, physicians must assess whether to continue, modify or immediately stop the study.

Vulnerable Groups and Individuals
19. Some groups and individuals are particularly vulnerable and may have an increased likelihood of being wronged or of incurring additional harm. All vulnerable groups and individuals should receive specifically considered protection.
20. Medical research with a vulnerable group is only justified if the research is responsive to the health needs or priorities of this group and the research cannot be carried out in a non vulnerable group. In addition, this group should stand to benefit from the knowledge, practices or interventions that result from the research.

Via Giardini, 1355
Baggiovara
41126 Modena
tel. : 059396111
fax.: 0593962409

Scientific Requirements and Research Protocols

21. Medical research involving human subjects must conform to generally accepted scientific principles, be based on a thorough knowledge of the scientific literature, other relevant sources of information, and adequate laboratory and, as appropriate, animal experimentation. The welfare of animals used for research must be respected.

22. The design and performance of each research study involving human subjects must be clearly described and justified in a research protocol. The protocol should contain a statement of the ethical considerations involved and should indicate how the principles in this Declaration have been addressed. The protocol should include information regarding funding, sponsors, institutional affiliations, potential conflicts of interest, incentives for subjects and information regarding provisions for treating and/or compensating subjects who are harmed as a consequence of participation in the research study.

In clinical trials, the protocol must also describe appropriate arrangements for post-trial provisions.

Research Ethics Committees

23. The research protocol must be submitted for consideration, comment, guidance and approval to the concerned research ethics committee before the study begins. This committee must be transparent in its functioning, must be independent of the researcher, the sponsor and any other undue influence and must be duly qualified. It must take into consideration the laws and regulations of the country or countries in which the research is to be performed as well as applicable international norms and standards but these must not be allowed to reduce or eliminate any of the protections for research subjects set forth in this Declaration. The committee must have the right to monitor ongoing studies. The researcher must provide monitoring information to the committee, especially information about any serious adverse events. No amendment to the protocol may be made without consideration and approval by the committee. After the end of the study, the researchers must submit a final report to the committee containing a summary of the study's findings and conclusions.

Privacy and Confidentiality

24. Every precaution must be taken to protect the privacy of research subjects and the confidentiality of their personal information.

Informed Consent

25. Participation by individuals capable of giving informed consent as subjects in medical research must be voluntary. Although it may be appropriate to consult family members or community leaders, no individual capable of giving informed consent may be enrolled in a research study unless he or she freely agrees.

26. In medical research involving human subjects capable of giving informed consent, each potential subject must be adequately informed of the aims, methods, sources of funding, any possible conflicts of interest, institutional affiliations of the researcher, the anticipated benefits and potential risks of the study and the discomfort it may entail, post-study provisions and any other relevant aspects of the study. The potential subject must be informed of the right to refuse to participate in the study or to withdraw consent to participate at any time without reprisal. Special attention should be given to the specific information needs of individual potential subjects as well as to the methods used to deliver the information. After ensuring that the potential subject has

Via Giardini, 1355
Baggiovara
41126 Modena
tel. : 059396111
fax.: 0593962409

understood the information, the physician or another appropriately qualified individual must then seek the potential subject's freely-given informed consent, preferably in writing. If the consent cannot be expressed in writing, the non-written consent must be formally documented and witnessed. All medical research subjects should be given the option of being informed about the general outcome and results of the study.

27. When seeking informed consent for participation in a research study the physician must be particularly cautious if the potential subject is in a dependent relationship with the physician or may consent under duress. In such situations the informed consent must be sought by an appropriately qualified individual who is completely independent of this relationship.

28. For a potential research subject who is incapable of giving informed consent, the physician must seek informed consent from the legally authorised representative. These individuals must not be included in a research study that has no likelihood of benefit for them unless it is intended to promote the health of the group represented by the potential subject, the research cannot instead be performed with persons capable of providing informed consent, and the research entails only minimal risk and minimal burden.

29. When a potential research subject who is deemed incapable of giving informed consent is able to give assent to decisions about participation in research, the physician must seek that assent in addition to the consent of the legally authorised representative. The potential subject's dissent should be respected.

30. Research involving subjects who are physically or mentally incapable of giving consent, for example, unconscious patients, may be done only if the physical or mental condition that prevents giving informed consent is a necessary characteristic of the research group. In such circumstances the physician must seek informed consent from the legally authorised representative. If no such representative is available and if the research cannot be delayed, the study may proceed without informed consent provided that the specific reasons for involving subjects with a condition that renders them unable to give informed consent have been stated in the research protocol and the study has been approved by a research ethics committee. Consent to remain in the research must be obtained as soon as possible from the subject or a legally authorised representative.

31. The physician must fully inform the patient which aspects of their care are related to the research. The refusal of a patient to participate in a study or the patient's decision to withdraw from the study must never adversely affect the patient-physician relationship.

32. For medical research using identifiable human material or data, such as research on material or data contained in biobanks or similar repositories, physicians must seek informed consent for its collection, storage and/or reuse. There may be exceptional situations where consent would be impossible or impracticable to obtain for such research. In such situations the research may be done only after consideration and approval of a research ethics committee.

Use of Placebo

33. The benefits, risks, burdens and effectiveness of a new intervention must be tested against those of the best proven intervention(s), except in the following circumstances: Where no proven intervention exists, the use of placebo, or no intervention, is acceptable; or Where for compelling and scientifically sound methodological reasons the use of any intervention less effective than the best proven one, the use of placebo, or no intervention is necessary to determine the efficacy or safety of an intervention and the patients who receive any intervention less effective than the best proven one, placebo, or no intervention will not be subject to additional risks of serious or

Via Giardini, 1355
Baggiovara
41126 Modena
tel. : 059396111
fax.: 0593962409

irreversible harm as a result of not receiving the best proven intervention. Extreme care must be taken to avoid abuse of this option.

Post-Trial Provisions

34. In advance of a clinical trial, sponsors, researchers and host country governments should make provisions for post-trial access for all participants who still need an intervention identified as beneficial in the trial. This information must also be disclosed to participants during the informed consent process.

Research Registration and Publication and Dissemination of Results

35. Every research study involving human subjects must be registered in a publicly accessible database before recruitment of the first subject.

36. Researchers, authors, sponsors, editors and publishers all have ethical obligations with regard to the publication and dissemination of the results of research. Researchers have a duty to make publicly available the results of their research on human subjects and are accountable for the completeness and accuracy of their reports. All parties should adhere to accepted guidelines for ethical reporting. Negative and inconclusive as well as positive results must be published or otherwise made publicly available. Sources of funding, institutional affiliations and conflicts of interest must be declared in the publication. Reports of research not in accordance with the principles of this Declaration should not be accepted for publication.

Unproven Interventions in Clinical Practice

37. In the treatment of an individual patient, where proven interventions do not exist or other known interventions have been ineffective, the physician, after seeking expert advice, with informed consent from the patient or a legally authorised representative, may use an unproven intervention if in the physician's judgement it offers hope of saving life, re-establishing health or alleviating suffering. This intervention should subsequently be made the object of research, designed to evaluate its safety and efficacy. In all cases, new information must be recorded and, where appropriate, made publicly available.

Dipartimento Integrato di Neuroscienze
Unità Operativa di Neurologia
Ospedale Civile Sant'Agostino Estense

SERVIZIO SANITARIO REGIONALE
EMILIA-ROMAGNA
Azienda Ospedaliero - Universitaria di Modena

Via Giardini, 1355
Baggiovara
41126 Modena
tel. : 059396111
fax.: 0593962409

17.3 ITALIAN REGULATION ON CELLS/TISSUES TRANSPLANTATION

9-11-2007 *Supplemento ordinario* alla GAZZETTA UFFICIALE *Serie generale* - n. **261**

LEGGI ED ALTRI ATTI NORMATIVI

DECRETO LEGISLATIVO 6 novembre 2007, n. **191**.

Attuazione della direttiva 2004/23/CE sulla definizione delle norme di qualità e di sicurezza per la donazione, l'approvvigionamento, il controllo, la lavorazione, la conservazione, lo stoccaggio e la distribuzione di tessuti e cellule umani.

IL PRESIDENTE DELLA REPUBBLICA

Visti gli articoli 76 e 87 della Costituzione;

Vista la legge 21 dicembre 1999, n. 526, recante disposizioni per l'adempimento di obblighi derivanti dall'appartenenza dell'Italia alle Comunità europee - legge comunitaria 1999;

Vista la direttiva 2004/23/CE del Parlamento europeo e del Consiglio, del 31 marzo 2004, sulla definizione di norme di qualità e di sicurezza per la donazione, l'approvvigionamento, il controllo, la lavorazione, la conservazione, lo stoccaggio e la distribuzione di tessuti e cellule umani;

Vista la legge 25 gennaio 2006, n. 29, recante disposizioni per l'adempimento di obblighi derivanti dall'appartenenza dell'Italia alle Comunità europee - legge comunitaria 2005, ed in particolare l'articolo 1, commi 1 e 3, e l'allegato A;

Vista la legge 1° aprile 1999, n. 91, recante disposizioni in materia di prelievi e di trapianti di organi e di tessuti;

Vista la legge 21 ottobre 2005, n. 219, recante nuova disciplina delle attività trasfusionali e della produzione nazionale degli emoderivati;

Visto il decreto del Presidente della Repubblica in data 14 gennaio 1997, recante approvazione dell'atto di indirizzo e coordinamento alle regioni e alle province autonome di Trento e di Bolzano, in materia di requisiti strutturali, tecnologici ed organizzativi minimi per l'esercizio delle attività sanitarie da parte delle strutture pubbliche e private, pubblicato nel supplemento ordinario alla *Gazzetta Ufficiale* n. 42 del 20 febbraio 1997;

Visto il decreto legislativo 19 giugno 1999, n. 229;

Visto il decreto del Presidente del Consiglio dei Ministri in data 1° settembre 2000, recante approvazione dell'atto di indirizzo e coordinamento in materia di requisiti strutturali, tecnologici ed organizzativi minimi per l'esercizio delle attività sanitarie relative alla medicina trasfusionale, pubblicato nella *Gazzetta Ufficiale* n. 274 del 23 novembre 2000;

Visto l'accordo 21 marzo 2002 tra il Ministro della salute, le regioni e le province autonome di Trento e di Bolzano, concernente linee guida per le attività di coordinamento per il reperimento di organi e di tessuti in ambito nazionale ai fini di trapianto;

Visto l'accordo 21 marzo 2002 tra il Ministro della salute, le regioni e le province autonome di Trento e di Bolzano, concernente linee guida per il prelievo, la conservazione e l'utilizzo di tessuto muscolo-scheletrico;

Visto il decreto del Ministro della salute in data 2 dicembre 2004, recante modalità per il rilascio delle autorizzazioni all'esportazione o all'importazione di organi e tessuti, pubblicato nella *Gazzetta Ufficiale* n. 27 del 3 febbraio 2005;

Visto l'accordo 23 settembre 2004 tra il Ministro della salute, le regioni e le province autonome di Trento e di Bolzano, su linee guida sulle modalità di disciplina delle attività di reperimento, trattamento, conservazione e distribuzione di cellule e tessuti umani a scopo di trapianto, in attuazione dell'articolo 15, comma 1, della legge 1° aprile 1999, n. 91, pubblicato nella *Gazzetta Ufficiale* n. 248 del 21 ottobre 2007;

Visto l'accordo 10 luglio 2003 tra il Ministro della salute, le regioni e le province autonome di Trento e di Bolzano sul documento recante linee guida in tema di raccolta, manipolazione e impiego clinico delle cellule staminali emopoietiche (CSE), pubblicato nella *Gazzetta Ufficiale* n. 227 del 30 settembre 2007;

Visto il decreto legislativo 19 agosto 2005, n. 191, recante attuazione della direttiva 2002/98/CE, che stabilisce norme di qualità e di sicurezza per la raccolta, il controllo, la lavorazione, la conservazione e la distribuzione del sangue umano e dei suoi componenti, e successive modificazioni;

Vista la legge 19 febbraio 2004, n. 40, recante norme in materia di procreazione medicalmente assistita;

Vista la preliminare deliberazione del Consiglio dei Ministri, adottata nella riunione del 27 luglio 2007;

Acquisito il parere della Conferenza permanente per i rapporti tra lo Stato, le regioni e le province autonome di Trento e di Bolzano nella seduta del 20 settembre 2007;

Acquisiti i pareri delle competenti Commissioni della Camera dei deputati e del Senato della Repubblica;

Vista la deliberazione del Consiglio dei Ministri, adottata nella riunione del 23 ottobre 2007;

Sulla proposta del Ministro per le politiche europee e del Ministro della salute, di concerto con i Ministri degli affari esteri, della giustizia, dell'economia e delle finanze e per gli affari regionali e le autonomie locali;

EMANA

il seguente decreto legislativo:

Capo I

DISPOSIZIONI GENERALI

Art. 1.

Obiettivi

1. Il presente decreto stabilisce norme di qualità e di sicurezza per i tessuti e le cellule umani destinati ad applicazioni sull'uomo, al fine di assicurare un elevato livello di protezione della salute umana.

Dipartimento Integrato di Neuroscienze
Unità Operativa di Neurologia
Ospedale Civile Sant'Agostino Estense

SERVIZIO SANITARIO REGIONALE
EMILIA-ROMAGNA
Azienda Ospedaliero - Universitaria di Modena

Via Giardini, 1355
Baggiovara
41126 Modena
tel. : 059396111
fax.: 0593962409

9-11-2007 *Supplemento ordinario* alla GAZZETTA UFFICIALE *Serie generale* - n. **261**

Art. 2.

Ambito di applicazione

1. Il presente decreto si applica alla donazione, all'approvvigionamento, al controllo, alla lavorazione, alla conservazione, allo stoccaggio e alla distribuzione di tessuti e cellule umani destinati ad applicazioni sull'uomo, nonché ai prodotti fabbricati derivati da tessuti e cellule umani destinati ad applicazioni sull'uomo. Qualora tali prodotti fabbricati siano disciplinati da altre norme di derivazione europea, il presente decreto si applica soltanto alla donazione, all'approvvigionamento e al controllo degli stessi.

2. Le disposizioni di cui al presente decreto legislativo si applicano anche alla donazione, alla raccolta, al controllo, alla conservazione di cellule staminali emopoietiche, ferme restando le disposizioni previste dalla normativa vigente in materia di attività trasfusionali.

3. Alle cellule riproduttive, ai tessuti e alle cellule fetali e alle cellule staminali embrionali, si applicano le disposizioni vigenti in materia. Per la conservazione si applicano le disposizioni di cui al presente decreto in quanto compatibili.

4. Il presente decreto non si applica a:

a) tessuti e cellule utilizzati per un trapianto autologo nell'ambito dello stesso intervento chirurgico;

b) sangue e suoi componenti secondo la definizione del decreto legislativo 19 agosto 2005, n. 191, e successive modificazioni;

c) organi o parti di organi, qualora la loro funzione sia quella di essere utilizzati per lo stesso scopo dell'organo intero nel corpo umano.

Art. 3.

Definizioni

1. Ai fini del presente decreto si intende per:

a) «cellule»: le cellule umane singole o un insieme di cellule umane non collegate da alcuna forma di tessuto connettivo;

b) «tessuto»: tutte le parti costituenti del corpo umano formate da cellule;

c) «donatore»: qualsiasi fonte umana, vivente o deceduta, di cellule o tessuti umani;

d) «donazione»: l'atto di donare tessuti o cellule umani destinati ad applicazioni sull'uomo;

e) «organo»: una parte differenziata e vitale del corpo umano, formata da diversi tessuti, che mantiene la propria struttura, vascolarizzazione e capacità di sviluppare funzioni fisiologiche con un sensibile livello di autonomia;

f) «approvvigionamento»: il processo che rende disponibile il tessuto o le cellule;

g) «lavorazione»: tutte le operazioni connesse con la preparazione, la manipolazione, la conservazione e l'imballaggio dei tessuti o delle cellule destinati ad applicazioni sull'uomo;

h) «conservazione»: l'uso di agenti chimici, alterazioni delle condizioni ambientali o altri mezzi impiegati durante la lavorazione per impedire o ritardare il deterioramento biologico o fisico di cellule o tessuti;

i) «quarantena»: lo stato dei tessuti o cellule prelevati, o di un tessuto isolato fisicamente o tramite altri mezzi appropriati, in attesa di una decisione sulla loro accettazione o sul loro rifiuto;

l) «stoccaggio»: il mantenimento del prodotto in condizioni adeguate e controllate fino alla sua distribuzione;

m) «distribuzione»: il trasporto e la consegna di tessuti o cellule destinati ad applicazioni sull'uomo;

n) «applicazione sull'uomo»: l'uso di tessuti o cellule su o in un ricevente umano, nonché le applicazioni extra-corporee;

o) «evento avverso grave»: qualunque evento negativo collegato con l'approvvigionamento, il controllo, la lavorazione, lo stoccaggio e la distribuzione di tessuti e cellule, che possa provocare la trasmissione di malattie trasmissibili, la morte o condizioni di pericolo di vita, di invalidità o incapacità dei pazienti, o ne produca o prolunghi l'ospedalizzazione o lo stato di malattia;

p) «reazione avversa grave»: una risposta non voluta nel donatore o nel ricevente, compresa una malattia trasmissibile, connessa con l'approvvigionamento o l'applicazione sull'uomo di tessuti o cellule, che provochi la morte, metta in pericolo la vita o produca invalidità o incapacità dell'interessato, o ne produca o prolunghi l'ospedalizzazione o lo stato di malattia;

q) «istituto dei tessuti»: una banca dei tessuti, come prevista dalla legge 1° aprile 1999, n. 91, o una unità di un ospedale pubblico, o un settore di un servizio trasfusionale come previsto dal decreto legislativo 19 agosto 2005, n. 191, o una struttura sanitaria senza fini di lucro, in cui si effettuano attività di lavorazione, conservazione, stoccaggio o distribuzione di tessuti e cellule umani, o una struttura sanitaria autorizzata ai sensi della legge 19 febbraio 2004, n. 40, per le attività compatibili con la legge medesima. Fatte salve le competenze delle regioni e province autonome in materia di autorizzazione al funzionamento degli istituti, il Ministero della salute, le regioni e le province autonome di Trento e di Bolzano, di seguito denominate «province autonome», mediante accordo in Conferenza permanente per i rapporti tra lo Stato, le regioni e le province autonome di Trento e di Bolzano, emanano le linee guida per l'accreditamento delle attività di tali istituti, conformemente alla normativa vigente in materia, sulla base delle indicazioni fornite dal Centro nazionale trapianti, dal Centro nazionale sangue e dall'Istituto superiore di Sanità secondo i rispettivi ambiti di competenza. L'Istituto dei tessuti può inoltre essere incaricato dell'approvvigionamento o del controllo, comprendente anche l'esecuzione degli esami analitici, dei tessuti e delle cellule;

s) «uso allogenico»: cellule o tessuti prelevati da una persona ed applicati ad un'altra;

t) «uso autologo»: cellule o tessuti prelevati da una persona ed applicati sulla stessa persona.

Art. 4.

Attuazione

1. Il Ministero della salute e le regioni e le province autonome sono le autorità responsabili competenti per l'attuazione dei requisiti di cui al presente decreto.

Capo II

OBBLIGHI DELLE AUTORITÀ COMPETENTI

Art. 5.

Vigilanza sull'approvvigionamento dei tessuti e delle cellule umani

1. L'approvvigionamento e il controllo dei tessuti e delle cellule sono effettuati da personale in possesso dei requisiti richiesti dalla normativa vigente e che abbia formazione ed esperienza adeguate e si svolgono secondo modalità a tale fine autorizzate dalle regioni e dalle province autonome.

2. Con decreto del Ministro della salute, previa intesa con le regioni e le province autonome, sono impartite le disposizioni per assicurare che l'approvvigionamento dei tessuti e delle cellule sia attuato nel rispetto della normativa vigente nel settore e in coerenza ai requisiti di cui all'articolo 28, comma 1, lettere *b)*, *e)* e *f)*. I controlli, compresi gli esami analitici, richiesti per i donatori sono effettuati dalle strutture a tali fini specificamente individuate, autorizzate e accreditate secondo le modalità previste dalle regioni o dalle province autonome.

Art. 6.

Autorizzazione e accreditamento degli istituti dei tessuti e dei procedimenti di preparazione dei tessuti e delle cellule

1. Con accordo in sede di Conferenza permanente per i rapporti tra lo Stato, le regioni e le province autonome di Trento e di Bolzano, entro sei mesi dalla data di entrata in vigore del presente decreto, sono definiti i requisiti minimi organizzativi, strutturali e tecnologici degli istituti dei tessuti e le linee-guida per l'accreditamento, sulla base delle indicazioni all'uopo fornite dal CNT, dal CNS e dalla Conferenza dei Presidenti delle regioni e delle province autonome, per le rispettive competenze.

2. Ai sensi della normativa vigente e del presente decreto, gli istituti dei tessuti in cui si svolgono attività di controllo, lavorazione, conservazione, stoccaggio o distribuzione di tessuti e cellule umani destinati ad applicazioni sull'uomo sono autorizzati e accreditati dalle regioni e dalle province autonome, ai fini dello svolgimento di tali attività.

3. La regione o la provincia autonoma, previo accertamento della conformità dell'istituto dei tessuti ai requisiti previsti dalla normativa vigente e dal presente decreto, con particolare riferimento all'articolo 28, comma 1, lettera *a)*, lo autorizza e lo accredita, ed indica le attività di cui è consentito l'esercizio, prevedendone le condizioni. La regione o la provincia autonoma autorizza ed accredita le attività relative ai procedimenti di preparazione dei tessuti e delle cellule, che l'istituto dei tessuti può svolgere nel rispetto della normativa vigente nel settore e dei requisiti di cui all'articolo 28, comma 1, lettera *g)*. Gli accordi previsti all'articolo 24, conclusi fra un istituto dei tessuti e terzi sono esaminati nell'ambito di tale procedura.

4. L'istituto dei tessuti non apporta modifiche sostanziali alle proprie attività senza il rilascio della preventiva autorizzazione da parte della regione o della provincia autonoma interessata.

5. La regione o la provincia autonoma competente può sospendere o revocare l'autorizzazione e l'accreditamento di un istituto dei tessuti o di un procedimento di preparazione dei tessuti e delle cellule qualora l'ispezione o le misure di controllo attuate dimostrino che tale istituto o procedimento non soddisfano i requisiti previsti.

6. Con decreto del Ministro della salute, d'intesa con la Conferenza permanente per i rapporti tra lo Stato, le regioni e le province autonome di Trento e di Bolzano, sono individuati, nel rispetto dei requisiti di cui all'articolo 28, comma 1, lettera *i)*, i tessuti e le cellule specifici che possono essere distribuiti direttamente per il trapianto immediato al ricevente, a condizione che il fornitore abbia ottenuto per tale attività l'autorizzazione e l'accreditamento da parte delle regioni o delle province autonome competenti.

Art. 7.

Ispezioni e misure di controllo

1. La regione o la provincia autonoma organizza ispezioni e adeguate misure di controllo presso gli istituti dei tessuti, avvalendosi per gli specifici ambiti di competenza del supporto del CNT o del CNS, per verificarne la rispondenza ai requisiti previsti dalla normativa vigente e dal presente decreto.

2. La regione o la provincia autonoma assicura, inoltre, l'adozione di appropriate misure di controllo per quanto riguarda l'approvvigionamento di tessuti e cellule umani.

3. Le ispezioni e le misure di controllo, di cui ai commi 1 e 2, sono attuate a intervalli di tempo regolari e comunque non superiori a due anni.

4. Tali ispezioni e misure di controllo sono effettuate da personale incaricato che ha il potere di:

a) ispezionare gli istituti dei tessuti e le strutture dei terzi indicati all'articolo 24;

b) valutare e verificare le procedure e le attività svolte negli istituti dei tessuti, nonché nelle strutture dei terzi sopraindicati;

c) esaminare qualsiasi documento o altre registrazioni connessi a quanto richiesto dalla normativa vigente e dal presente decreto.

5. Con accordo in sede di Conferenza permanente per i rapporti tra lo Stato, le regioni e le province autonome di Trento e di Bolzano sono stabiliti, anche in conformità alle indicazioni fornite dagli organismi europei, i criteri relativi allo svolgimento delle ispezioni

UNIMORE
UNIVERSITÀ DEGLI STUDI DI MODENA E REGGIO EMILIA

Dipartimento Integrato di Neuroscienze
Unità Operativa di Neurologia
Ospedale Civile Sant'Agostino Estense

SERVIZIO SANITARIO REGIONALE
EMILIA-ROMAGNA
Azienda Ospedaliero - Universitaria di Modena

Via Giardini, 1355
Baggiovara
41126 Modena
tel. : 059396111
fax.: 0593962409

e delle misure di controllo, e quelli inerenti alla formazione ed alla qualificazione del personale interessato, al fine di raggiungere livelli omogenei di competenza e rendimento.

6. La regione o la provincia autonoma organizza ispezioni ed attua misure di controllo adeguate in caso di reazioni o eventi avversi gravi, avvalendosi per gli specifici ambiti di competenza del supporto del CNT o del CNS. In tale caso sono inoltre organizzate ispezioni e attuate misure di controllo su richiesta debitamente motivata da parte delle autorità competenti di un altro Stato membro.

7. Con accordo in sede di Conferenza permanente per i rapporti tra lo Stato, le regioni e le province autonome di Trento e di Bolzano sono stabiliti modalità e tempi per corrispondere alla richiesta della Commissione europea o di un altro Stato dell'Unione europea in ordine ai risultati delle ispezioni e delle misure di controllo attuate in relazione ai disposti del presente decreto.

Art. 8.

Tracciabilità

1. Con apposito decreto di recepimento di direttive tecniche europee adottato dal Ministro della salute, di concerto con il Ministro dell'economia e delle finanze, sono fissate le disposizioni necessarie a garantire per tutti i tessuti e le cellule prelevati, lavorati, stoccati o distribuiti sul territorio nazionale la tracciabilità del percorso dal donatore al ricevente e viceversa. Tale tracciabilità riguarda anche le informazioni concernenti prodotti e materiali che entrano in contatto con i medesimi tessuti e cellule.

2. Con il medesimo decreto di cui al comma 1 è istituito un sistema di individuazione dei donatori, che assegna un codice unico a ciascuna donazione e a ciascuno dei prodotti da essa derivati.

3. Tutti i tessuti e le cellule sono resi identificabili tramite un'etichetta contenente le informazioni o i riferimenti che ne consentono il collegamento con le fasi di cui all'articolo 28, comma 1, lettere *f)* e *h)*.

4. Gli istituti dei tessuti conservano i dati necessari ad assicurare la tracciabilità in tutte le fasi. I dati richiesti ai fini della completa tracciabilità sono conservati per un periodo minimo di trenta anni dopo l'uso clinico. L'archiviazione dei dati può avvenire anche in forma elettronica.

5. Con il decreto di cui al comma 1 sono anche fissati, nel rispetto della normativa vigente e delle indicazioni formulate in sede europea, i requisiti di tracciabilità per tessuti e cellule, così come per prodotti e materiali che entrano in contatto con i predetti tessuti e cellule e che possono influenzarne qualità e sicurezza.

6. Con apposito decreto di recepimento di direttive tecniche europee adottato dal Ministro della salute, di concerto con il Ministro dell'economia e delle finanze, è data attuazione alle procedure volte a garantire la tracciabilità a livello comunitario, formulate in sede europea.

Art. 9.

Importazione ed esportazione di tessuti e cellule umani

1. Con decreto del Ministro della salute, adottato di concerto con il Ministro dell'economia e delle finanze, d'intesa con la Conferenza permanente per i rapporti tra lo Stato, le regioni e le province autonome di Trento e di Bolzano, sono individuate tutte le misure necessarie per garantire che ogni importazione di tessuti e cellule da Paesi terzi sia effettuata da istituti dei tessuti accreditati o designati o autorizzati o titolari di licenza, ai fini dello svolgimento delle proprie attività e che i tessuti e le cellule importati siano rintracciabili dal donatore al ricevente e viceversa, come previsto dall'articolo 8. Gli istituti dei tessuti che ricevono tali importazioni da Paesi terzi ne assicurano la conformità a norme di qualità e sicurezza equivalenti a quelle fissate dalla normativa vigente e dal presente decreto.

2. Con il decreto di cui al comma 1 sono fissate, altresì, tutte le misure necessarie per garantire che ogni esportazione di tessuti e cellule verso Paesi terzi sia effettuata da istituti dei tessuti autorizzati e accreditati, e che la stessa sia conforme ai requisiti previsti dalla normativa vigente e dal presente decreto.

3. Il succitato decreto regola anche:

a) l'importazione e l'esportazione di tessuti e cellule specifici, di cui all'articolo 6, comma 6;

b) l'importazione o l'esportazione di tessuti e cellule specifici, di cui all'articolo 6, comma 6, in caso di emergenza;

c) l'adozione di tutte le misure necessarie per assicurare che le importazioni e le esportazioni dei tessuti e cellule di cui alle lettere *a)* e *b)* siano conformi a norme di qualità e di sicurezza equivalenti a quelle fissate dalla normativa vigente e dal presente decreto.

4. Con apposito decreto del Ministro della salute sono recepite le procedure, stabilite in sede europea, volte alla verifica del rispetto delle norme di qualità e di sicurezza equivalenti.

Art. 10.

Registro degli istituti dei tessuti e obbligo di presentare relazioni

1. Gli istituti dei tessuti istituiscono e conservano un registro delle loro attività, nel quale sono riportati anche i tipi e le quantità di tessuti e/o di cellule prelevati, controllati, conservati, lavorati, stoccati e distribuiti o altrimenti utilizzati, e l'origine e la destinazione dei tessuti e delle cellule destinati ad applicazioni sull'uomo, in conformità alle norme vigenti e ai disposti di cui all'articolo 28, comma 1, lettera *f)*. Gli istituti dei tessuti presentano, alle regioni o alle province autonome e al CNT, al CNS e all'Istituto superiore di sanità (ISS), secondo le rispettive competenze, una relazione annuale sulle attività svolte, accessibile al pubblico.

2. Le regioni e le province autonome istituiscono e aggiornano un registro degli istituti dei tessuti, accessi-

UNIMORE
UNIVERSITÀ DEGLI STUDI DI MODENA E REGGIO EMILIA

Dipartimento Integrato di Neuroscienze
Unità Operativa di Neurologia
Ospedale Civile Sant'Agostino Estense

SERVIZIO SANITARIO REGIONALE
EMILIA-ROMAGNA
Azienda Ospedaliero - Universitaria di Modena

Via Giardini, 1355
Baggiovara
41126 Modena
tel. : 059396111
fax.: 0593962409

bile al pubblico, nel quale sono specificate le attività per le quali ciascun istituto è stato autorizzato e accreditato.

3. Presso il CNT o il CNS o l'ISS, in relazione alle rispettive competenze, è istituito ed aggiornato un registro nazionale degli istituti dei tessuti in cui sono specificate le attività per le quali ciascun istituto è stato autorizzato e accreditato.

3. A livello europeo è istituita la rete dei registri nazionali degli istituti dei tessuti.

Art. 11.

Notifica di eventi e reazioni avversi gravi

1. Con apposito decreto di recepimento di direttive tecniche europee adottato dal Ministro della salute, di concerto con il Ministro dell'economia e delle finanze, viene definito un sistema atto a notificare, controllare, registrare e trasmettere le informazioni riguardanti eventi e reazioni avversi gravi che possono influire sulla qualità e la sicurezza di tessuti e cellule e che possono essere connessi all'approvvigionamento, al controllo, alla lavorazione, allo stoccaggio e alla distribuzione dei tessuti e delle cellule, nonché qualsiasi altra reazione avversa grave, inclusa la cessazione della funzione desiderata del tessuto valutata mediante follow up, osservata nel corso o a seguito dell'applicazione clinica, che possa essere in rapporto con la qualità e la sicurezza dei tessuti e delle cellule.

2. Il medico o la struttura sanitaria che utilizza tessuti e cellule umani, disciplinati dalla normativa vigente e dal presente decreto, comunica ogni informazione pertinente a tale utilizzo agli istituti coinvolti nella donazione, approvvigionamento, controllo, lavorazione, stoccaggio e distribuzione dei medesimi tessuti e cellule umani, per facilitare la tracciabilità e garantire il controllo della qualità e della sicurezza.

3. La persona responsabile dell'istituto dei tessuti, così come definita dall'articolo 17, garantisce che la regione o la provincia autonoma e il CNT o il CNS, per gli specifici ambiti di competenza, siano informati degli eventi o reazioni avversi gravi di cui al comma 1, e che ricevano una relazione analitica delle relative cause e conseguenze.

4. La procedura di notifica di eventi e reazioni avversi gravi è stabilita, in base alle indicazioni formulate in sede europea, con apposito decreto di recepimento di direttive tecniche europee adottato dal Ministro della salute.

5. Ciascun istituto dei tessuti stabilisce una procedura accurata, rapida e verificabile per ritirare dalla distribuzione qualsiasi prodotto che possa essere connesso a eventi o reazioni avverse.

Capo III

SELEZIONE E VALUTAZIONE DEI DONATORI

Art. 12.

Principi della donazione di tessuti e cellule

1. La donazione di tessuti e cellule è volontaria e gratuita.

2. Il Ministero della salute, d'intesa con le regioni e le province autonome e con il CNT o il CNS, secondo le rispettive competenze, presenta alla Commissione europea relazioni in ordine alle iniziative intraprese in tale senso anteriormente al 30 giugno 2008 e in seguito ogni tre anni.

3. Il Ministero della salute, le regioni e le province autonome, il CNT o il CNS, in collaborazione con le rispettive associazioni e federazioni dei donatori volontari adottano, nei limiti degli ordinari stanziamenti di bilancio, tutte le misure necessarie per assicurare che le attività di promozione e pubblicità a favore della donazione di tessuti e cellule umani siano conformi agli orientamenti e alle disposizioni legislative vigenti.

Art. 13.

Consenso ed espressione di volontà

1. L'approvvigionamento di tessuti o cellule umani è consentito solo se sono soddisfatti tutti i requisiti previsti dalla normativa vigente in ordine al consenso informato o all'espressione di volontà o all'autorizzazione alla donazione.

2. Con decreto del Ministro della salute, d'intesa con la Conferenza permanente per i rapporti tra lo Stato, le regioni e le province autonome di Trento e di Bolzano, sono fissate le misure necessarie per garantire che i donatori, i loro congiunti o le persone che danno l'autorizzazione per conto dei donatori, ricevano, oltre a tutte le informazioni previste dalle norme vigenti, anche quelle di cui all'allegato al presente decreto.

Art. 14.

Protezione dei dati e tutela della riservatezza

1. Tutti i dati, comprese le informazioni genetiche, raccolti ai sensi delle disposizioni vigenti e del presente decreto ed ai quali abbiano accesso terzi, sono resi anonimi in modo tale che né il donatore né il ricevente siano identificabili.

2. A tale fine è garantito che:

a) siano adottate misure di protezione dei dati e misure di tutela volte ad evitare aggiunte, soppressioni o modifiche dei dati non autorizzate negli archivi riguardanti i donatori o nei registri dei donatori esclusi, o qualunque trasferimento di informazioni;

b) siano istituite procedure volte a risolvere le divergenze tra i dati;

c) non avvenga alcuna divulgazione non autorizzata di tali informazioni, garantendo nel contempo la tracciabilità delle donazioni.

UNIMORE
UNIVERSITÀ DEGLI STUDI DI MODENA E REGGIO EMILIA

Dipartimento Integrato di Neuroscienze
Unità Operativa di Neurologia
Ospedale Civile Sant'Agostino Estense

SERVIZIO SANITARIO REGIONALE
EMILIA-ROMAGNA
Azienda Ospedaliero - Universitaria di Modena

Via Giardini, 1355
Baggiovara
41126 Modena
tel. : 059396111
fax.: 0593962409

3. Nel rispetto delle disposizioni vigenti in materia, l'identità del o dei riceventi non è rivelata al donatore o alla sua famiglia e viceversa.

Art. 15.

Selezione, valutazione e approvvigionamento.

1. Le attività connesse all'approvvigionamento dei tessuti sono effettuate in modo da assicurare che la valutazione e la selezione del donatore si svolgano nel rispetto delle disposizioni vigenti e di quanto previsto dall'articolo 28, comma 1, lettere *d)* ed *e)*, e che i tessuti e le cellule siano prelevati, confezionati e trasportati in conformità alla normativa vigente e ai disposti di cui all'articolo 28, comma 1, lettera *f)*.

2. In caso di donazione autologa, i criteri di adeguatezza sono stabiliti conformemente ai requisiti di cui all'articolo 28, comma 1, lettera *d)*.

3. I risultati delle procedure di valutazione e controllo del donatore sono documentati e qualsiasi risultato anomalo significativo è comunicato al medesimo ai sensi della normativa vigente e dell'allegato 1 al presente decreto.

4. La persona responsabile dell'istituto dei tessuti garantisce che tutte le attività connesse con l'approvvigionamento dei tessuti si svolgano nel rispetto della normativa vigente e dei disposti dell'articolo 28, comma 1, lettera *f)*.

Capo IV

DISPOSIZIONI RELATIVE ALLA QUALITÀ E SICUREZZA DEI TESSUTI E DELLE CELLULE

Art. 16.

Gestione della qualità

1. Con apposito decreto di recepimento di direttive tecniche europee adottato dal Ministro della salute, di concerto con il Ministro dell'economia e delle finanze, sono individuate tutte le misure perché ciascun istituto dei tessuti istituisca e aggiorni un sistema di qualità, fondato sui principi di buona prassi.

2. Con provvedimento analogo sono recepite le norme e le specifiche comunitarie di cui all'articolo 28, comma 1, lettera *c)*, per le attività relative al sistema di qualità.

3. Gli istituti dei tessuti adottano tutte le misure necessarie per assicurare che il sistema di qualità comprenda almeno la seguente documentazione:

a) procedure operative standard;

b) linee-guida;

c) manuali di formazione e di riferimento;

d) moduli per le relazioni;

e) dati relativi ai donatori;

f) informazioni sulla destinazione finale dei tessuti o delle cellule.

4. Gli istituti dei tessuti assicurano la disponibilità della documentazione di cui al comma 3 nel momento dell'ispezione da parte dell'autorità competente o delle autorità competenti, ai sensi dell'articolo 7.

5. Gli istituti dei tessuti conservano i dati necessari per garantire la tracciabilità come previsto dall'articolo 8.

Art. 17.

Persona responsabile

1. L'Ente titolare dell'autorizzazione e accreditamento, in funzione delle attività svolte dall'istituto dei tessuti, ne designa il responsabile che soddisfa almeno le seguenti condizioni:

a) possesso del diploma di laurea in medicina e chirurgia o in scienze biologiche; nel caso in cui la persona responsabile sia un biologo, gli deve essere comunque assicurata la possibilità di avvalersi di una professionalità medica per gli aspetti di competenza;

b) esperienza pratica di almeno due anni nei settori pertinenti.

2. La persona designata ai sensi del comma 1 ha le seguenti responsabilità:

a) garantire che i tessuti e le cellule umani destinati ad applicazioni sull'uomo, nell'ambito dell'istituto di cui è responsabile, siano prelevati, controllati, lavorati, stoccati e distribuiti ai sensi delle disposizioni vigenti e del presente decreto;

b) fornire alla regione o alla provincia autonoma le informazioni richieste ai sensi dell'articolo 6;

c) attuare, relativamente alle attività svolte, le disposizioni previste agli articoli 7, 10, 11, 15, 16 ed agli articoli da 18 a 24.

3. L'Ente titolare dell'autorizzazione e accreditamento comunica alla regione o alla provincia autonoma il nome della persona responsabile dell'istituto dei tessuti. Qualora la persona responsabile debba essere temporaneamente o permanentemente sostituita, il sopraindicato Ente comunica alla regione o alla provincia autonoma il nome del nuovo responsabile e la data di assunzione delle funzioni.

Art. 18.

Personale

1. Il personale degli istituti dei tessuti che interviene direttamente nelle attività connesse con l'approvvigionamento, la lavorazione, la conservazione, lo stoccaggio e la distribuzione di tessuti e cellule possiede le qualifiche necessarie secondo la normativa vigente per svolgere tali funzioni e riceve adeguata formazione professionale, in conformità anche ai disposti di cui all'articolo 28, comma 1, lettera *c)*.

Via Giardini, 1355
Baggiovara
41126 Modena
tel. : 059396111
fax.: 0593962409

Art. 19.

Ricevimento dei tessuti e delle cellule

1. Gli istituti dei tessuti garantiscono che tutte le donazioni di tessuti e cellule umani siano stati sottoposti ai controlli previsti dalla normativa vigente e alle disposizioni di cui all'articolo 28, comma 1, lettera *e)*, e che la selezione e l'accettazione dei tessuti e delle cellule siano risultate conformi alle disposizioni vigenti e a quelle di cui all'articolo 28, comma 1, lettera *f)*.

2. Gli istituti dei tessuti garantiscono la conformità dei tessuti e delle cellule umani e della documentazione relativa, alle norme vigenti e alle disposizioni di cui all'articolo 28, comma 1, lettera *f)*.

3. Gli istituti dei tessuti verificano e registrano la conformità delle condizioni di confezionamento dei tessuti e delle cellule umani ricevuti alle norme vigenti e ai requisiti di cui all'articolo 28, comma 1, lettera *f)*. I tessuti e le cellule non conformi a tali disposizioni sono scartati.

4. L'accettazione o il rifiuto dei tessuti o delle cellule ricevuti sono documentati.

5. Gli istituti dei tessuti garantiscono la corretta e costante identificazione dei tessuti e delle cellule umani. Ad ogni consegna o partenza di tessuti o cellule è attribuito un codice d'identificazione, ai sensi dell'articolo 8.

6. I tessuti e le cellule sono tenuti in quarantena sino al soddisfacimento dei requisiti relativi al controllo e all'informazione del donatore, ai sensi dell'articolo 15.

Art. 20.

Lavorazione dei tessuti e delle cellule

1. Gli istituti dei tessuti includono nelle proprie procedure operative standard ogni processo di lavorazione che incida sulla qualità e la sicurezza ed assicurano che tali processi si svolgano in condizioni controllate. Gli istituti dei tessuti assicurano che il materiale utilizzato, l'ambiente di lavoro, nonché l'organizzazione, la convalida e le condizioni di controllo dei processi siano conformi alle disposizioni vigenti e ai requisiti di cui all'articolo 28, comma 1, lettera *h)*.

2. Qualunque modifica dei processi utilizzati nella preparazione dei tessuti e delle cellule è resa parimenti conforme ai criteri indicati al comma 1.

3. Nelle proprie procedure operative standard gli istituti dei tessuti adottano speciali disposizioni per la manipolazione dei tessuti e delle cellule da scartare, al fine di impedire la contaminazione di altri tessuti o cellule, dell'ambiente in cui avviene la lavorazione o del personale.

Art. 21.

Condizioni di stoccaggio dei tessuti e delle cellule

1. Gli istituti dei tessuti garantiscono che tutti i procedimenti connessi con lo stoccaggio di tessuti e cellule siano documentati dalle procedure operative standard e che le condizioni di stoccaggio siano conformi alla normativa vigente e ai requisiti di cui all'articolo 28, comma 1, lettera *h)*.

2. Gli istituti dei tessuti garantiscono lo svolgimento di tutti i processi di stoccaggio in condizioni controllate.

3. Gli istituti dei tessuti istituiscono ed attuano procedure per il controllo dei reparti di confezionamento e stoccaggio, al fine di prevenire qualunque evento che possa compromettere la funzione o l'integrità dei tessuti e delle cellule.

4. I tessuti e le cellule lavorati non sono distribuiti fino a quando i requisiti previsti dalla normativa vigente e dal presente decreto non sono stati soddisfatti.

5. Con accordo in sede di Conferenza permanente per i rapporti tra lo Stato, le regioni e le province autonome di Trento e di Bolzano sono stabilite le indicazioni per gli accordi e le procedure che gli istituti dei tessuti, in caso di cessazione dell'attività per qualsivoglia ragione, pongono in atto per garantire che i tessuti e le cellule stoccati, in conformità al consenso informato o l'espressione di volontà o l'autorizzazione alla donazione acquisiti, siano trasferiti ad altro istituto o istituti dei tessuti autorizzati e accreditati ai sensi dell'articolo 6.

Art. 22.

Etichettatura, documentazione e confezionamento

1. Gli istituti dei tessuti garantiscono la conformità dell'etichettatura, della documentazione e del confezionamento ai requisiti previsti dalla normativa vigente e dall'articolo 28, comma 1, lettera *f)*.

Art. 23.

Trasporto e distribuzione

1. Gli istituti dei tessuti garantiscono la qualità dei tessuti e delle cellule durante la distribuzione, che avviene nel rispetto delle condizioni previste dalle norme vigenti e dei requisiti di cui all'articolo 28, comma 1, lettera *h)*.

Art. 24.

Rapporti fra istituti dei tessuti e terzi

1. Gli istituti dei tessuti, previa autorizzazione della regione o provincia autonoma, concludono accordi scritti con terzi, ogniqualvolta vi sia un intervento esterno e tale attività influisca sulla qualità e la sicurezza dei tessuti e delle cellule lavorati, in particolare nei seguenti casi:

a) qualora l'istituto dei tessuti affidi a terzi una fase della lavorazione dei tessuti o delle cellule;

b) qualora i terzi forniscano beni e servizi incidenti sulla garanzia di qualità e di sicurezza dei tessuti o cellule, inclusa la loro distribuzione;

c) qualora un istituto dei tessuti distribuisca tessuti o cellule lavorati da terzi.

2. Ove i terzi intervengano in una fase della lavorazione dei tessuti e delle cellule, preliminarmente alla stipula dell'accordo, sono sottoposti, da parte del CNT o del CNS, secondo le specifiche competenze, alla verifica del possesso dei requisiti prescritti dalla normativa vigente e dal presente decreto per lo svolgimento della specifica attività. Alla verifica del possesso dei requisiti segue il rilascio da parte del Ministero della salute di un nulla osta all'esercizio di detta attività di lavorazione a favore di istituti dei tessuti.

Via Giardini, 1355
Baggiovara
41126 Modena
tel. : 059396111
fax.: 0593962409

3. Gli istituti dei tessuti conservano un elenco completo degli accordi di cui al comma 1 che essi hanno concluso con terzi.

4. Gli accordi conclusi fra un istituto dei tessuti e terzi specificano in particolare le responsabilità che gravano sui terzi e le relative procedure dettagliate.

5. Gli istituti dei tessuti forniscono copia degli accordi conclusi con terzi alla regione o provincia autonoma e, in relazione alle specifiche competenze, al CNT o al CNS.

Capo V

SCAMBIO DI INFORMAZIONI, RELAZIONI E SANZIONI

Art. 25.

Codifica delle informazioni

1. Con il provvedimento di cui all'articolo 8, comma 1, è istituito un sistema per l'identificazione dei tessuti e delle cellule umani, al fine di garantirne la completa tracciabilità, ai sensi dell'articolo 8.

2. A livello europeo viene elaborato un unico sistema di codificazione, al fine di fornire informazioni sulle caratteristiche e proprietà fondamentali dei tessuti e delle cellule.

Art. 26.

Relazioni

1. Il Ministero della salute presenta alla Commissione europea, anteriormente al 7 aprile 2009 e, successivamente, ogni tre anni, una relazione sulle attività svolte in riferimento alle disposizioni del presente decreto, comprendente un rendiconto delle misure adottate in materia di ispezione e di controllo.

2. Entro sei mesi dalla data di entrata in vigore del presente decreto, con apposito accordo in sede di Conferenza permanente per i rapporti tra lo Stato, le regioni e le province autonome di Trento e di Bolzano, sono stabiliti modalità e tempi per corrispondere ai disposti di cui al comma 1.

Art. 27.

Sanzioni

1. Salvo che il fatto costituisca più grave reato, chiunque preleva, procura, raccoglie, conserva, lavora, distribuisce cellule e tessuti destinati all'applicazione sull'uomo, nonché prodotti fabbricati derivati da tessuti e cellule umani destinati ad applicazioni sull'uomo al di fuori degli istituti di cui all'articolo 3, comma 1, lettera *q)*, o in assenza o in violazione delle prescritte autorizzazioni è punito con l'arresto da uno a tre anni e con l'ammenda da euro 5.000 ad euro 15.000. Se il colpevole è persona che esercita una professione sanitaria, alla condanna segue l'interdizione dall'esercizio della professione per uguale periodo.

2. Salvo che il fatto costituisca più grave reato, la persona responsabile di un istituto dei tessuti, come definito dall'articolo 3, comma 1, lettera *q)*, o il suo legittimo delegato, che non svolge una o più delle funzioni di competenza di cui all'articolo 17, comma 2, è punito con l'arresto da due a sei mesi, o con l'ammenda da euro 5.000 ad euro 15.000. Salvo che il fatto costituisca più grave reato, chiunque pone la persona responsabile di un istituto dei tessuti, in condizione di non poter svolgere una o più delle funzioni di competenza di cui all'articolo 17, comma 2, è punito con l'arresto da due a sei mesi, o con l'ammenda da euro 5.000 ad euro 15.000.

3. La persona che interviene nella raccolta, nel controllo, nella lavorazione, nella conservazione, nella distribuzione di cellule e tessuti destinati all'applicazione sull'uomo, senza possedere le qualificazioni previste dalla normativa vigente per svolgere tali attività, è punita con l'arresto da due a sei mesi o con l'ammenda da euro 5.000 ad euro 15.000.

4. Chiunque impedisca oppure ostacoli, in qualsiasi modo, il compimento da parte del personale incaricato delle attività di cui all'articolo 7, comma 4, o non assicuri la dovuta collaborazione, è punito con la sanzione amministrativa da euro 5.000 ad euro 30.000. Se si tratta di pubblico dipendente si applica l'interdizione dal pubblico ufficio per un periodo non inferiore a giorni novanta.

Capo VI

DISPOSIZIONI FINALI

Art. 28.

Requisiti tecnici e loro adeguamento al progresso scientifico e tecnico

1. Con appositi decreti del Ministero della salute sono recepite le disposizioni, emanate a livello europeo, per l'adeguamento al progresso tecnico e scientifico dei requisiti tecnici nelle materie di seguito elencate:

a) requisiti per l'autorizzazione e l'accreditamento degli istituti dei tessuti;

b) requisiti per l'approvvigionamento di tessuti o cellule umani;

c) sistema di qualità, compresa la formazione;

d) criteri di selezione dei donatori di tessuti e/o cellule;

e) esami di laboratorio richiesti per i donatori;

f) procedure per l'approvvigionamento di cellule e/o tessuti e ricevimento all'istituto dei tessuti;

g) requisiti per i procedimenti di preparazione di tessuti e cellule;

h) lavorazione, stoccaggio e distribuzione di tessuti e cellule;

i) requisiti per la distribuzione diretta al ricevente di tessuti e cellule specifici.

Art. 29.

Clausola di invarianza degli oneri di spesa

1. Le amministrazioni interessate provvedono alle attività previste dal presente decreto con le risorse umane, finanziarie e strumentali disponibili a legislazione vigente.

Via Giardini, 1355
Baggiovara
41126 Modena
tel. : 059396111
fax.: 0593962409

2. Dall'attuazione del presente decreto non devono derivare nuovi o maggiori oneri a carico della finanza pubblica.

Art. 30.

Clausola di cedevolezza

1. In relazione a quanto disposto dall'articolo 117, quinto comma, della Costituzione e dall'articolo 16, comma 3, della legge 4 febbraio 2005, n. 11, le disposizioni del presente decreto legislativo riguardanti ambiti di competenza legislativa delle regioni e delle province autonome si applicano, nell'esercizio del potere sostituivo dello Stato e con carattere di cedevolezza, a decorrere dalla scadenza del termine stabilito per l'attuazione della/e direttiva/e oggetto del presente decreto legislativo, nelle regioni e nelle province autonome nelle quali non sia ancora stata adottata la normativa di attuazione regionale o provinciale e perdono comunque efficacia dalla data di entrata in vigore di quest'ultima, fermi restando i principi fondamentali ai sensi dell'articolo 117, comma terzo, della Costituzione.

Il presente decreto, munito del sigillo dello Stato, sarà inserito nella Raccolta ufficiale degli atti normativi della Repubblica italiana. È fatto obbligo a chiunque spetti di osservarlo e di farlo osservare.

Dato a Roma, addì 6 novembre 2007

NAPOLITANO

PRODI, *Presidente del Consiglio dei Ministri*

BONINO, *Ministro per le politiche europee*

TURCO, *Ministro della salute*

D'ALEMA, *Ministro degli affari esteri*

MASTELLA, *Ministro della giustizia*

PADOA SCHIOPPA, *Ministro dell'economia e delle finanze*

LANZILLOTTA, *Ministro per gli affari regionali e le autonomie locali*

Visto, *il Guardasigilli:* MASTELLA

ALLEGATO I
I (previsto dall'articolo 15, comma 3)

INFORMAZIONI DA FORNIRE SULLA DONAZIONE DI TESSUTI E/O DI CELLULE

A. *Donatori viventi.*

1. La persona responsabile del processo di donazione garantisce che il donatore, o i soggetti legittimati ad esprimere il consenso alla donazione, siano stati adeguatamente informati almeno degli aspetti relativi al processo di donazione e approvvigionamento di cui al punto 3. Le informazioni sono fornite prima dell'approvvigionamento.

2. Le informazioni sono fornite da personale sanitario appositamente formato, capace di comunicarle in modo chiaro e adeguato, usando termini facilmente comprensibili per il donatore o per i soggetti legittimati ad esprimere il consenso alla donazione.

3. Dette informazioni comprendono:

scopo e natura dell'approvvigionamento, sue conseguenze e rischi; esami analitici, se effettuati; registrazione e protezione dei dati del donatore, riservatezza medica; scopo terapeutico e potenziali benefici, nonché informazioni sulle garanzie applicabili volte a tutelare il donatore.

4. Il donatore ed i soggetti legittimati ad esprimere il consenso alla donazione sono informati del diritto ad ottenere i risultati confermati degli esami analitici ed a ricevere spiegazioni chiare in merito a tali risultati.

5. Sono inoltre fornite informazioni sulla necessità di acquisire, a seconda dei casi, il consenso informato o l'espressione di volontà o l'autorizzazione previsti dalle disposizioni vigenti affinché si possa procedere all'approvvigionamento di tessuti e/o cellule.

B. *Donatori deceduti.*

1. Sono acquisite tutte le informazioni necessarie ed avere ottenuto i necessari consensi e le autorizzazioni, come previsto dalla legislazione vigente.

2. I risultati della valutazione del donatore, una volta confermati, sono comunicati e spiegati chiaramente alle persone interessate, conformemente alla legislazione vigente.

NOTE

AVVERTENZA:

Il testo delle note qui pubblicato è stato redatto dall'amministrazione competente per materia ai sensi dell'art. 10, comma 3, del testo unico delle disposizioni sulla promulgazione delle leggi, sull'emanazione dei decreti del Presidente della Repubblica e sulle pubblicazioni ufficiali della Repubblica italiana, approvato con D.P.R. 28 dicembre 1985, n. 1092, al solo fine di facilitare la lettura delle disposizioni di legge alle quali è operato il rinvio. Restano invariati il valore e l'efficacia degli atti legislativi qui trascritti.

Via Giardini, 1355
Baggiovara
41126 Modena
tel. : 059396111
fax.: 0593962409

Per regolamenti e direttive CE vengono forniti gli estremi di pubblicazione nella Gazzetta ufficiale dell'Unione europea (GUUE).

Note alle premesse:

— L'art. 76 della Costituzione delega l'esercizio della funzione legislativa al Governo, per un periodo di tempo limitato e per oggetti definiti, previa determinazione di principi e criteri direttivi.

— L'art. 87 della Costituzione conferisce, tra l'altro, al Presidente della Repubblica il potere di promulgare le leggi e di emanare i decreti aventi valore di legge ed i regolamenti.

— La legge 21 dicembre 1999, n. 526 «Disposizioni per l'adempimento di obblighi derivanti dall'appartenenza dell'Italia alle Comunità europee - legge comunitaria 1999», è pubblicata nel suppl. ord. alla *Gazzetta Ufficiale* n. 13 del 18 gennaio 2000.

— La Dir. 31 marzo 2004 n. 2004/23/CE «Direttiva del Parlamento europeo e del Consiglio sulla definizione di norme di qualità e di sicurezza per la donazione, l'approvvigionamento, il controllo, la lavorazione, la conservazione, lo stoccaggio e la distribuzione di tessuti e cellule umani», pubblicata nella G.U.U.E. 7 aprile 2004, n. L 102.

— L'art. 1, commi 1 e 3, e l'allegato A, della legge 25 gennaio 2006, n. 29, pubblicato nel suppl. ord. alla *Gazzetta Ufficiale* n. 32 dell'8 febbraio 2006, così recitano:

«Art. 1 *(Delega al Governo per l'attuazione di direttive comunitarie).* — 1. Il Governo è delegato ad adottare, entro il termine di diciotto mesi dalla data di entrata in vigore della presente legge, i decreti legislativi recanti le norme occorrenti per dare attuazione alle direttive comprese negli elenchi di cui agli allegati A e B.

2. *(Omissis).*

3. Gli schemi dei decreti legislativi recanti attuazione delle direttive comprese nell'elenco di cui all'allegato B, nonché, qualora sia previsto il ricorso a sanzioni penali, quelli relativi all'attuazione delle direttive elencate nell'allegato A, sono trasmessi, dopo l'acquisizione degli altri pareri previsti dalla legge, alla Camera dei deputati e al Senato della Repubblica perché su di essi sia espresso il parere dei competenti organi parlamentari. Decorsi quaranta giorni dalla data di trasmissione, i decreti sono emanati anche in mancanza del parere. Qualora il termine per l'espressione del parere parlamentare di cui al presente comma, ovvero i diversi termini previsti dai commi 4 e 9, scadano nei trenta giorni che precedono la scadenza dei termini previsti ai commi 1 o 5 o successivamente, questi ultimi sono prorogati di novanta giorni.».

«ALLEGATO A
(art. 1, commi 1 e 3)

2004/10/CE del Parlamento europeo e del Consiglio, dell'11 febbraio 2004, concernente il ravvicinamento delle disposizioni legislative, regolamentari ed amministrative relative all'applicazione dei principi di buona pratica di laboratorio e al controllo della loro applicazione per le prove sulle sostanze chimiche.

2004/23/CE del Parlamento europeo e del Consiglio, del 31 marzo 2004, sulla definizione di norme di qualità e di sicurezza per la donazione, l'approvvigionamento, il controllo, la lavorazione, la conservazione, lo stoccaggio e la distribuzione di tessuti e cellule umani.

2004/41/CE del Parlamento europeo e del Consiglio, del 21 aprile 2004, che abroga alcune direttive recanti norme sull'igiene dei prodotti alimentari e le disposizioni sanitarie per la produzione e la commercializzazione di determinati prodotti di origine animale destinati al consumo umano e che modifica la direttiva 89/662/CEE e la direttiva 92/118/CEE del Consiglio e la decisione 95/408/CE del Consiglio.

2004/68/CE del Consiglio, del 26 aprile 2004, che stabilisce norme di polizia sanitaria per le importazioni e il transito nella Comunità di determinati ungulati vivi, che modifica la direttiva 90/426/CEE e la direttiva 92/65/CEE e che abroga la direttiva 72/462/CEE.

2004/107/CE del Parlamento europeo e del Consiglio, del 15 dicembre 2004, concernente l'arsenico, il cadmio, il mercurio, il nickel e gli idrocarburi policiclici aromatici nell'aria ambiente.

2004/114/CE del Consiglio, del 13 dicembre 2004, relativa alle condizioni di ammissione dei cittadini di paesi terzi per motivi di studio, scambio di alunni, tirocinio non retribuito o volontariato.

2004/117/CE del Consiglio, del 22 dicembre 2004, che modifica la direttiva 66/401/CEE, la direttiva 66/402/CEE, la direttiva 2002/54/CE, la direttiva 2002/55/CE e la direttiva 2002/57/CE per quanto riguarda gli esami eseguiti sotto sorveglianza ufficiale e l'equivalenza delle sementi prodotte in paesi terzi.

2005/1/CE del Parlamento europeo e del Consiglio, del 9 marzo 2005, che modifica la direttiva 73/239/CEE, la direttiva 85/611/CEE, la direttiva 91/675/CEE, la direttiva 92/49/CEE e la direttiva 93/6/CEE del Consiglio e la direttiva 94/19/CE, la direttiva 98/78/CE, la direttiva 2000/12/CE, la direttiva 2001/34/CE, la direttiva 2002/83/CE e la direttiva 2002/87/CE al fine di istituire una nuova struttura organizzativa per i comitati del settore dei servizi finanziari.

2005/29/CE del Parlamento europeo e del Consiglio, dell'11 maggio 2005, relativa alle pratiche commerciali sleali tra imprese e consumatori nel mercato interno e che modifica la direttiva 84/450/CEE del Consiglio e le direttive 97/7/CE, 98/27/CE e 2002/65/CE del Parlamento europeo e del Consiglio e il regolamento (CE) n. 2006/2004 del Parlamento europeo e del Consiglio («direttiva sulle pratiche commerciali sleali»).

2005/50/CE della Commissione, dell'11 agosto 2005, relativa alla riclassificazione delle protesi articolari dell'anca, del ginocchio e della spalla nel quadro della direttiva 93/42/CEE concernente i dispositivi medici.".».

— La legge 1° aprile 1999, n. 91, «Disposizioni in materia di prelievi e di trapianti di organi e di tessuti» è pubblicata nella *Gazzetta Ufficiale* del 15 aprile 1999, n. 87.

— La legge 21 ottobre 2005, n. 219 «Nuova disciplina delle attività trasfusionali e della produzione nazionale degli emoderivati», è pubblicata nella *Gazzetta Ufficiale* del 27 ottobre 2005, n. 251.

— Il decreto legislativo 19 giugno 1999, n. 229, reca: «Norme per la razionalizzazione del Servizio sanitario nazionale, a norma dell'art. 1 della legge 30 novembre 1998, n. 419.».

— L'art. 15 della legge 1° aprile 1999, n. 91, «Disposizioni in materia di prelievi e di trapianti di organi e di tessuti», pubblicata nella *Gazzetta. Ufficiale* del 15 aprile 1999, n. 87, così recita:

«Art. 15 *(Strutture per la conservazione dei tessuti prelevati).* — 1. Le regioni, sentito il centro regionale o interregionale, individuano le strutture sanitarie pubbliche aventi il compito di conservare e distribuire i tessuti prelevati, certificandone la idoneità e la sicurezza.

2. Le strutture di cui al comma 1 sono tenute a registrare i movimenti in entrata ed in uscita dei tessuti prelevati, inclusa l'importazione, secondo le modalità definite dalle regioni.».

— Il decreto legislativo 19 agosto 2005, n. 191 «Attuazione della direttiva 2002/98/CE che stabilisce norme di qualità e di sicurezza per la raccolta, il controllo, la lavorazione, la conservazione e la distribuzione del sangue umano e dei suoi componenti» è pubblicato nella *Gazzetta Ufficiale* del 22 settembre 2005, n. 221.

— La Direttiva 2002/98/CE Parlamento europeo e del Consiglio del 27 gennaio 2003 che stabilisce norme di qualità e di sicurezza per la raccolta, il controllo, la lavorazione, la conservazione e la distribuzione del sangue umano e dei suoi componenti e che modifica la direttiva 2001/83/CE è pubblicata nella Gazzetta ufficiale dell'Unione europea L 33/30 IT dell'8 febbraio 2003.

— La legge 19 febbraio 2004, n. 40 «Norme in materia di procreazione medicalmente assistita» è pubblicata nella *Gazzetta Ufficiale* del 24 febbraio 2004, n. 45.

Via Giardini, 1355
Baggiovara
41126 Modena
tel. : 059396111
fax.: 0593962409

Nota all'art. 2:

— Per il decreto legislativo 19 agosto 2005, n. 191, vedi le note alle premesse.

Nota all'art. 3:

— Per la legge 1° aprile 1999, n. 91, il decreto legislativo 19 agosto 2005, n. 191 e la legge 19 febbraio 2004, n. 40, vedi note alle premesse.

Note all'art. 30:

— L'art. 117 della Cost. 27 dicembre 1947 «Costituzione della Repubblica italiana» così recita:

«Art. 117. — La potestà legislativa è esercitata dallo Stato e dalle regioni nel rispetto della Costituzione, nonché dei vincoli derivanti dall'ordinamento comunitario e dagli obblighi internazionali .

Lo Stato ha legislazione esclusiva nelle seguenti materie:

a) politica estera e rapporti internazionali dello Stato; rapporti dello Stato con l'Unione europea; diritto di asilo e condizione giuridica dei cittadini di Stati non appartenenti all'Unione europea;

b) immigrazione;

c) rapporti tra la Repubblica e le confessioni religiose;

d) difesa e Forze armate; sicurezza dello Stato; armi, munizioni ed esplosivi;

e) moneta, tutela del risparmio e mercati finanziari; tutela della concorrenza; sistema valutario; sistema tributario e contabile dello Stato; perequazione delle risorse finanziarie;

f) organi dello Stato e relative leggi elettorali; referendum statali; elezione del Parlamento europeo;

g) ordinamento e organizzazione amministrativa dello Stato e degli enti pubblici nazionali;

h) ordine pubblico e sicurezza, ad esclusione della polizia amministrativa locale;

i) cittadinanza, stato civile e anagrafi;

l) giurisdizione e norme processuali; ordinamento civile e penale; giustizia amministrativa;

m) determinazione dei livelli essenziali delle prestazioni concernenti i diritti civili e sociali che devono essere garantiti su tutto il territorio nazionale;

n) norme generali sull'istruzione;

o) previdenza sociale;

p) legislazione elettorale, organi di governo e funzioni fondamentali di comuni, province e città metropolitane;

q) dogane, protezione dei confini nazionali e profilassi internazionale;

r) pesi, misure e determinazione del tempo; coordinamento informativo statistico e informatico dei dati dell'amministrazione statale, regionale e locale; opere dell'ingegno;

s) tutela dell'ambiente, dell'ecosistema e dei beni culturali.

Sono materie di legislazione concorrente quelle relative a: rapporti internazionali e con l'Unione europea delle regioni; commercio con l'estero; tutela e sicurezza del lavoro; istruzione, salva l'autonomia delle istituzioni scolastiche e con esclusione della istruzione e della formazione professionale; professioni; ricerca scientifica e tecnologica e sostegno all'innovazione per i settori produttivi; tutela della salute; alimentazione; ordinamento sportivo; protezione civile; governo del territorio; porti e aeroporti civili; grandi reti di trasporto e di navigazione; ordinamento della comunicazione; produzione, trasporto e distribuzione nazionale dell'energia; previdenza complementare e integrativa; armonizzazione dei bilanci pubblici e coordinamento della finanza pubblica e del sistema tributario; valorizzazione dei beni culturali e ambientali e promozione e organizzazione di attività culturali; casse di risparmio, casse rurali, aziende di credito a carattere regionale; enti di credito fondiario e agrario a carattere regionale. Nelle materie di legislazione concorrente spetta alle Regioni la potestà legislativa, salvo che per la determinazione dei principi fondamentali, riservata alla legislazione dello Stato.

Spetta alle regioni la potestà legislativa in riferimento ad ogni materia non espressamente riservata alla legislazione dello Stato.

Le regioni e le province autonome di Trento e di Bolzano, nelle materie di loro competenza, partecipano alle decisioni dirette alla formazione degli atti normativi comunitari e provvedono all'attuazione e all'esecuzione degli accordi internazionali e degli atti dell'Unione europea, nel rispetto delle norme di procedura stabilite da legge dello Stato, che disciplina le modalità di esercizio del potere sostitutivo in caso di inadempienza.

La potestà regolamentare spetta allo Stato nelle materie di legislazione esclusiva, salva delega alle regioni. La potestà regolamentare spetta alle regioni in ogni altra materia. I comuni, le province e le città metropolitane hanno potestà regolamentare in ordine alla disciplina dell'organizzazione e dello svolgimento delle funzioni loro attribuite.

Le leggi regionali rimuovono ogni ostacolo che impedisce la piena parità degli uomini e delle donne nella vita sociale, culturale ed economica e promuovono la parità di accesso tra donne e uomini alle cariche elettive.

La legge regionale ratifica le intese della regione con altre regioni per il migliore esercizio delle proprie funzioni, anche con individuazione di organi comuni.

Nelle materie di sua competenza la regione può concludere accordi con Stati e intese con enti territoriali interni ad altro Stato, nei casi e con le forme disciplinati da leggi dello Stato.».

— L'art. 16 della legge 4 febbraio 2005, n. 11 «Norme generali sulla partecipazione dell'Italia al processo normativo dell'Unione europea e sulle procedure di esecuzione degli obblighi comunitari», pubblicata nella *Gazzetta Ufficiale* 15 febbraio 2005, n. 37, così recita:

«Art. 16 *(Attuazione delle direttive comunitarie da parte delle regioni e delle province autonome).* — 1. Le regioni e le province autonome, nelle materie di propria competenza, possono dare immediata attuazione alle direttive comunitarie. Nelle materie di competenza concorrente la legge comunitaria indica i principi fondamentali non derogabili dalla legge regionale o provinciale sopravvenuta e prevalenti sulle contrarie disposizioni eventualmente già emanate dalle regioni e dalle province autonome.

2. I provvedimenti adottati dalle regioni e dalle province autonome per dare attuazione alle direttive comunitarie, nelle materie di propria competenza legislativa, devono recare nel titolo il numero identificativo della direttiva attuata e devono essere immediatamente trasmessi in copia conforme alla Presidenza del Consiglio dei Ministri - Dipartimento per le politiche comunitarie.

3. Ai fini di cui all'art. 117, quinto comma, della Costituzione, le disposizioni legislative adottate dallo Stato per l'adempimento degli obblighi comunitari, nelle materie di competenza legislativa delle regioni e delle province autonome, si applicano, per le regioni e le province autonome, alle condizioni e secondo la procedura di cui all'art. 11, comma 8, secondo periodo.

4. Nelle materie di cui all'art. 117, secondo comma, della Costituzione, cui hanno riguardo le direttive, il Governo indica i criteri e formula le direttive ai quali si devono attenere le regioni e le province autonome ai fini del soddisfacimento di esigenze di carattere unitario, del perseguimento degli obiettivi della programmazione economica e del rispetto degli impegni derivanti dagli obblighi internazionali. Detta funzione, fuori dai casi in cui sia esercitata con legge o con atto avente forza di legge o, sulla base della legge comunitaria, con i regolamenti previsti dall'art. 11, è esercitata mediante deliberazione del Consiglio dei Ministri, su proposta del Presidente del Consiglio dei Ministri o del Ministro per le politiche comunitarie, d'intesa con i Ministri competenti secondo le modalità di cui all'art. 8 della legge 15 marzo 1997, n. 59.».

07G0221

Dipartimento Integrato di Neuroscienze
Unità Operativa di Neurologia
Ospedale Civile Sant'Agostino Estense

SERVIZIO SANITARIO REGIONALE
EMILIA-ROMAGNA
Azienda Ospedaliero - Universitaria di Modena

Via Giardini, 1355
Baggiovara
41126 Modena
tel. : 059396111
fax.: 0593962409

18-2-2010 GAZZETTA UFFICIALE DELLA REPUBBLICA ITALIANA *Serie generale* - n. **40**

LEGGI ED ALTRI ATTI NORMATIVI

DECRETO LEGISLATIVO 25 gennaio 2010, n. **16**.

Attuazione delle direttive 2006/17/CE e 2006/86/CE, che attuano la direttiva 2004/23/CE per quanto riguarda le prescrizioni tecniche per la donazione, l'approvvigionamento e il controllo di tessuti e cellule umani, nonché per quanto riguarda le prescrizioni in tema di rintracciabilità, la notifica di reazioni ed eventi avversi gravi e determinate prescrizioni tecniche per la codifica, la lavorazione, la conservazione, lo stoccaggio e la distribuzione di tessuti e cellule umani.

IL PRESIDENTE DELLA REPUBBLICA

Visti gli articoli 76 e 87 della Costituzione;

Vista la legge 21 dicembre 1999, n. 526;

Visto il decreto legislativo 6 novembre 2007, n. 191, recante attuazione della direttiva del Parlamento europeo e del Consiglio 2004/23/CE del 31 marzo 2004, sulla definizione di norme di qualità e di sicurezza per la donazione, l'approvvigionamento, il controllo, la lavorazione, la conservazione, lo stoccaggio e la distribuzione di tessuti e cellule umani, con particolare riguardo per i suoi articoli 8, 11, comma 4, e 28, comma 1, lettere *a), b) c), d), e), f), g), h)* ed *i)*;

Vista la direttiva 2006/17/CE della Commissione dell'8 febbraio 2006 che attua la direttiva 2004/23/CE del Parlamento europeo e del Consiglio per quanto riguarda determinate prescrizioni tecniche per la donazione, l'approvvigionamento e il controllo di tessuti e cellule umani;

Vista la direttiva 2006/86/CE della Commissione del 24 ottobre 2006 che attua la direttiva 2004/23/CE del Parlamento europeo e del Consiglio per quanto riguarda le prescrizioni in tema di rintracciabilità, la notifica di reazioni ed eventi avversi gravi e determinate prescrizioni tecniche per la codifica, la lavorazione, la conservazione, lo stoccaggio e la distribuzione di tessuti e cellule umani;

Vista la legge 7 luglio 2009, n. 88, recante disposizioni per l'adempimento di obblighi derivanti dall'appartenenza dell'Italia alle Comunità europee - legge comunitaria 2008;

Vista la legge 1° aprile 1999, n. 91, recante disposizioni in materia di trapianti di organi e di tessuti;

Vista la legge 21 ottobre 2005, n. 219, recante nuova disciplina delle attività trasfusionali e della produzione nazionale degli emoderivati, e successive modificazioni;

Vista la legge 6 marzo 2001, n. 52, recante riconoscimento del registro nazionale italiano dei donatori di midollo osseo;

Visto il decreto del Presidente della Repubblica 14 gennaio 1997, pubblicato nella *Gazzetta Ufficiale* n. 42 del 20 febbraio 1997, recante approvazione dell'atto di indirizzo e coordinamento alle regioni e alle province autonome di Trento e di Bolzano, in materia di requisiti strutturali, tecnologici ed organizzativi minimi per l'esercizio delle attività sanitarie da parte delle strutture pubbliche e private;

Visto il decreto legislativo 30 dicembre 1992, n. 502, come modificato dalla legge 30 novembre 1998, n. 419, recante delega al Governo per la razionalizzazione del Servizio sanitario nazionale;

Visto il decreto del Presidente del Consiglio dei Ministri in data 1° settembre 2000, pubblicato nella *Gazzetta Ufficiale* n. 274 del 23 novembre 2000, recante approvazione dell'atto di indirizzo e coordinamento in materia di requisiti strutturali, tecnologici ed organizzativi minimi per l'esercizio delle attività sanitarie relative alla medicina trasfusionale;

Vista la legge 12 agosto 1993, n. 301, recante norme in materia di prelievo ed innesti di cornea;

Visto il decreto legislativo 24 febbraio 1997, n. 46, recante attuazione della direttiva 93/42/CEE concernente i dispositivi medici;

Visto il decreto legislativo 8 settembre 2000, n. 332, recante attuazione della direttiva 98/79/CE relativa ai dispositivi medico-diagnostici in vitro;

Visto l'Accordo 21 marzo 2002 tra il Ministero della salute, le regioni e le province autonome di Trento e di Bolzano, concernente linee guida per le attività di coordinamento per il reperimento di organi e di tessuti in ambito nazionale ai fini di trapianto;

Visto l'Accordo 21 marzo 2002 tra il Ministero della salute, le regioni e le province autonome di Trento e di Bolzano, concernente linee guida per il prelievo, la conservazione e l'utilizzo di tessuto muscolo-scheletrico;

Visto il decreto del Ministro della salute in data 2 dicembre 2004, pubblicato nella *Gazzetta Ufficiale* n. 27 del 3 febbraio 2005, recante modalità per il rilascio delle autorizzazioni all'esportazione o all'importazione di organi e tessuti;

Vista la legge 19 febbraio 2004, n. 40, recante norme in materia di procreazione medicalmente assistita;

Visto il decreto del Ministro della salute in data 11 aprile 2008, pubblicato nella *Gazzetta Ufficiale* n. 101 del 30 aprile 2008, recante linee guida in materia di procreazione medicalmente assistita;

UNIMORE
UNIVERSITÀ DEGLI STUDI DI MODENA E REGGIO EMILIA

Dipartimento Integrato di Neuroscienze
Unità Operativa di Neurologia
Ospedale Civile Sant'Agostino Estense

SERVIZIO SANITARIO REGIONALE
EMILIA-ROMAGNA
Azienda Ospedaliero - Universitaria di Modena

Via Giardini, 1355
Baggiovara
41126 Modena
tel. : 059396111
fax.: 0593962409

Visto l'Accordo 23 settembre 2004 tra il Ministero della salute, le regioni e le province autonome di Trento e di Bolzano, sul documento recante linee guida sulle modalità di disciplina delle attività di reperimento, trattamento, conservazione e distribuzione di cellule e tessuti umani a scopo, in attuazione dell'articolo 15, comma 1, della legge 1° aprile 1999, n. 91;

Visto l'Accordo 10 luglio 2003 tra il Ministero della salute, le regioni e le province autonome di Trento e di Bolzano, sul documento recante linee guida in tema di raccolta, manipolazione e impiego clinico delle cellule staminali emopoietiche (CSE);

Visto l'Accordo 5 ottobre 2006 tra il Ministero della salute, le regioni e le province autonome di Trento e di Bolzano in materia di ricerca e reperimento di cellule staminali emopoietiche presso registri e banche italiane ed estere;

Visto il decreto legislativo 20 dicembre 2007, n. 261, di revisione del decreto legislativo 19 agosto 2005, n. 191, recante attuazione della direttiva 2002/98/CE, che stabilisce norme di qualità e di sicurezza per la raccolta, il controllo, la lavorazione, la conservazione e la distribuzione del sangue umano e dei suoi componenti;

Vista la preliminare deliberazione del Consiglio dei Ministri, adottata nella riunione del 2 ottobre 2009;

Acquisito il parere della Conferenza permanente per i rapporti tra lo Stato, le regioni e le province autonome di Trento e di Bolzano nella seduta del 29 ottobre 2009;

Acquisiti i pareri delle competenti Commissioni della Camera dei deputati e del Senato della Repubblica;

Vista la deliberazione del Consiglio dei Ministri, adottata nella riunione del 13 gennaio 2010;

Sulla proposta del Ministro per le politiche europee e del Ministro della salute, di concerto con i Ministri degli affari esteri, della giustizia, dell'economia e delle finanze e per i rapporti con le regioni;

EMANA

il seguente decreto legislativo:

Art. 1.

Campo d'applicazione

1. Il presente decreto disciplina determinate prescrizioni tecniche per la donazione, l'approvvigionamento e il controllo di tessuti e cellule umani, nonché la codifica, la lavorazione, la conservazione, lo stoccaggio e la distribuzione di:

a) tessuti e cellule umani, destinati ad applicazioni sull'uomo;

b) prodotti fabbricati, derivati da tessuti e cellule umani destinati ad applicazioni sull'uomo, qualora tali prodotti non siano disciplinati da altre direttive.

2. Le disposizioni degli articoli da 10 a 14 del presente decreto, relative alla rintracciabilità e alla notifica di reazioni ed eventi avversi gravi, si applicano anche alla donazione, all'approvvigionamento e al controllo di tessuti e cellule umani.

3. Ai fini dell'attuazione delle disposizioni recate in materia di cellule riproduttive dal decreto legislativo 6 novembre 2007, n. 191, e dal presente decreto, il Ministero della salute e le Regioni si avvalgono della collaborazione del Centro nazionale trapianti, in seguito indicato come «CNT». Sono fatte salve le competenze dell'Istituto superiore di sanità di cui alla legge 19 febbraio 2004, n. 40.

Art. 2.

Definizioni

1. Ai fini del presente decreto si intendono per:

a) cellule riproduttive: tutti i tessuti e le cellule destinati ad essere utilizzati ai fini della riproduzione assistita, nel rispetto delle disposizioni vigenti in materia di procreazione medicalmente assistita;

b) donazione da parte di un partner: la donazione di cellule riproduttive tra un uomo e una donna che rispettino i requisiti soggettivi di cui all'articolo 5 della legge 19 febbraio 2004, n. 40;

c) impiego diretto: qualsiasi procedura in base alla quale le cellule donate vengono utilizzate senza essere conservate;

d) sistema di qualità: la struttura organizzativa, le responsabilità, le procedure, i processi e le risorse destinati ad attuare la gestione della qualità, comprese tutte le attività che direttamente o indirettamente contribuiscono alla qualità;

e) gestione della qualità: le attività coordinate per dirigere e controllare un'organizzazione sul piano della qualità;

f) procedure operative standard (POS): istruzioni scritte che descrivono le fasi di un determinato processo nonché i materiali e i metodi da utilizzare e il prodotto finale previsto;

g) convalida, o qualifica in caso di attrezzature o ambienti: la produzione di prove documentate, in grado di garantire con un elevato livello di certezza che determinati procedimenti, attrezzature o ambienti diano luogo a un prodotto conforme alle specifiche e alle caratteristiche qualitative prestabilite; un procedimento è convalidato al fine di valutare se il sistema funziona efficacemente in rapporto all'impiego previsto;

Via Giardini, 1355
Baggiovara
41126 Modena
tel. : 059396111
fax.: 0593962409

h) rintracciabilità: la possibilità di ricostruire il percorso di tessuti o cellule in ogni fase dell'approvvigionamento, della lavorazione, del controllo e dello stoccaggio fino alla distribuzione al ricevente o allo smaltimento, compresa la possibilità di risalire all'identificazione del donatore, dell'istituto dei tessuti o del centro di produzione che ricevono, o lavorano o stoccano i tessuti o le cellule, nonché, a livello delle strutture sanitarie, la possibilità di individuare i responsabili che applicano i tessuti o le cellule sui riceventi. Tale rintracciabilità riguarda anche la possibilità di reperire e identificare tutti i dati pertinenti relativi ai prodotti e ai materiali che vengono a contatto con detti tessuti o cellule;

i) critico: che ha potenzialmente effetto sulla qualità e o la sicurezza di tessuti e cellule o è a contatto con i predetti;

l) organizzazione per l'approvvigionamento: una struttura sanitaria o un'unità ospedaliera in cui si effettuano prelievi di tessuti e cellule umani, che può non essere autorizzata e accreditata come Istituto dei tessuti, fatto salvo quanto previsto dalla legge 12 agosto 1993, n. 301, recante norme in materia di prelievo ed innesti di cornea;

m) organizzazione responsabile dell'applicazione sull'uomo: una struttura sanitaria o un'unità ospedaliera che esegue applicazioni sull'uomo di tessuti e cellule.

Art. 3.

Approvvigionamento di tessuti e cellule umani

1. Ad eccezione della donazione da parte di un partner di cellule riproduttive destinate all'impiego diretto, l'approvvigionamento di tessuti e cellule umani è autorizzato solo qualora siano rispettate le prescrizioni di cui ai commi da 2 a 12.

2. Il prelievo di tessuti e cellule umani è effettuato da personale qualificato e adeguatamente formato, ai sensi della normativa vigente, a svolgere tali attività.

3. L'istituto dei tessuti o l'organizzazione per l'approvvigionamento conclude con il personale qualificato o l'equipe clinica responsabile della selezione del donatore che non faccia parte dello stesso istituto o organizzazione, accordi scritti in ordine alle procedure da seguire per garantire la conformità dei requisiti ai criteri di selezione dei donatori di cui all'allegato I.

4. L'istituto dei tessuti o l'organizzazione per l'approvvigionamento conclude con il personale qualificato o l'equipe clinica responsabile della selezione del donatore che non faccia parte dello stesso istituto o organizzazione, accordi scritti in ordine al tipo di tessuti, di cellule o di campioni da prelevare nonché ai protocolli da seguire.

5. Il responsabile dell'istituto dei tessuti, in accordo con l'organizzazione per l'approvvigionamento definisce procedure operative standard, in seguito indicate come «POS», al fine di verificare:

a) l'identità del donatore;

b) la documentazione relativa al consenso informato, o all'espressione di volontà o all'autorizzazione alla donazione da parte del donatore o della sua famiglia;

c) la valutazione dei criteri di selezione dei donatori di cui all'articolo 4;

d) la valutazione degli esami di laboratorio richiesti per i donatori di cui all'articolo 5.

6. Il responsabile dell'istituto dei tessuti, in accordo con l'organizzazione per l'approvvigionamento definisce altresì POS relative all'approvvigionamento, confezionamento, etichettatura e trasporto dei tessuti e delle cellule fino alla destinazione presso l'istituto dei tessuti o, in caso di distribuzione diretta di tali materiali, presso l'equipe clinica responsabile della loro applicazione, ovvero, in caso di campioni di tessuti e cellule, presso il laboratorio per il controllo, in conformità all'articolo 5.

7. L'approvvigionamento è eseguito presso strutture adeguate, ponendo in atto procedure mirate a ridurre il rischio di contaminazione batterica o di altro tipo dei tessuti e delle cellule prelevati ai sensi dell'articolo 6.

8. I materiali e le attrezzature utilizzati per l'approvvigionamento sono gestiti conformemente alle norme e alle specifiche di cui all'allegato IV, sezione 1.3, tenendo debitamente conto delle regolamentazioni, normative e linee guida nazionali ed internazionali, relative alla sterilizzazione di medicinali e dispositivi medici. Per il prelievo di tessuti e cellule sono impiegati appositi strumenti e dispositivi qualificati, sterili.

9. Il prelievo di tessuti e cellule da donatore vivente è effettuato in un contesto che ne garantisca la salute, la sicurezza e la tutela dei dati personali.

10. Nel caso di donatore cadavere è assicurata la disponibilità di personale e attrezzature per la ricomposizione del corpo che deve essere effettuata in modo completo ed efficace.

11. Le procedure relative al prelievo di tessuti e cellule sono attuate in conformità alle disposizioni di cui all'articolo 6.

12. Nel corso del prelievo o presso l'istituto dei tessuti viene assegnato un codice di identificazione unico al donatore ed ai tessuti e alle cellule donati, in modo da garantire un'adeguata identificazione del donatore e la tracciabilità dei materiali donati, nel rispetto delle norme per la tutela della riservatezza. I codici e i dati correlati sono annotati in un registro predisposto a tale fine.

13. La documentazione relativa al donatore è conservata conformemente a quanto previsto dall'allegato IV, punto 1.4.

Via Giardini, 1355
Baggiovara
41126 Modena
tel. : 059396111
fax.: 0593962409

Art. 4.

Criteri di selezione dei donatori

1. Il responsabile dell'organizzazione per l'approvvigionamento o dell'istituto dei tessuti garantisce che la selezione dei donatori sia effettuata in conformità ai criteri di selezione di cui:

a) all'allegato I per i donatori di tessuti e cellule;

b) all'allegato III per i donatori di cellule riproduttive.

2. Per i donatori di cellule staminali emopoietiche midollari e periferiche, ivi incluse quelle da sangue cordonale, si applicano anche le disposizioni previste dalla normativa vigente in materia di attività trasfusionali e di trapianto di midollo.

Art. 5.

Esami di laboratorio

1. Il responsabile dell'organizzazione per l'approvvigionamento o dell'istituto dei tessuti garantisce che:

a) i donatori di tessuti e di cellule, ad eccezione dei donatori di cellule riproduttive, vengano sottoposti agli esami di cui all'allegato II, punto 1;

b) tali esami siano effettuati secondo le prescrizioni stabilite nell'allegato II, punto 2.

2. Il responsabile dell'organizzazione per l'approvvigionamento o dell'istituto dei tessuti garantisce che:

a) i donatori di cellule riproduttive siano sottoposti agli esami di cui all'allegato III, punti 1 e 2;

b) gli esami di cui alla lettera *a)* siano effettuati secondo le prescrizioni stabilite nell'allegato III, punto 3.

3. Per i donatori di cellule staminali emopoietiche periferiche, ivi incluse quelle da sangue cordonale, si applicano anche le disposizioni previste dalla normativa vigente in materia di attività trasfusionali.

Art. 6.

Donazione e approvvigionamento di tessuti e/o di cellule e ricevimento presso l'istituto dei tessuti

1. Il responsabile dell'organizzazione per l'approvvigionamento o dell'istituto dei tessuti garantisce la conformità alle prescrizioni di cui all'allegato IV delle procedure relative alla donazione e all'approvvigionamento di tessuti o cellule nonché al ricevimento degli stessi presso l'istituto dei tessuti.

Art. 7.

Distribuzione diretta ai riceventi di determinati tessuti e cellule

1. Il responsabile dell'organizzazione per l'approvvigionamento o dell'istituto dei tessuti, acquisito il parere del Centro nazionale trapianti, può autorizzare la distribuzione diretta di determinati tessuti e cellule dal luogo in cui è effettuato il prelievo a un centro di assistenza sanitaria ai fini di un trapianto immediato.

Art. 8.

Prescrizioni per l'autorizzazione e l'accreditamento degli Istituti dei tessuti

1. L'allegato V al presente decreto riporta le indicazioni e le prescrizioni da soddisfare ai fini del rilascio, da parte dell'Autorità regionale competente, dell'autorizzazione e dell'accreditamento.

Art. 9.

Prescrizioni per l'autorizzazione e l'accreditamento allo svolgimento dei procedimenti di preparazione di tessuti e cellule

1. L'allegato VI al presente decreto riporta le indicazioni e le prescrizioni da soddisfare ai fini del rilascio, da parte dell'Autorità regionale competente, dell'autorizzazione allo svolgimento dei procedimenti di preparazione di tessuti e cellule.

Art. 10.

Notifica di reazioni avverse gravi

1. In conformità ai disposti di cui all'articolo 11 del decreto legislativo 6 novembre 2007, n. 191:

a) l'organizzazione per l'approvvigionamento predispone procedure per conservare le registrazioni dei dati relativi a tessuti e cellule prelevati e per notificare tempestivamente all'istituto dei tessuti di riferimento ogni reazione avversa grave nel donatore vivente che possa influire sulla qualità e sicurezza di tessuti e cellule;

b) l'organizzazione responsabile dell'applicazione sull'uomo di tessuti e cellule predispone procedure per conservare le registrazioni dei dati di tessuti e cellule applicati sull'uomo e per notificare tempestivamente all'istituto dei tessuti di riferimento ogni reazione avversa grave osservata nel corso o a seguito dell'applicazione clinica, che possa essere in rapporto con la qualità e la sicurezza dei tessuti e delle cellule utilizzate;

c) l'istituto dei tessuti che distribuisce tessuti e cellule per applicazioni sull'uomo fornisce all'organizzazione responsabile dell'applicazione sull'uomo di tessuti e cellule, coerentemente a quanto previsto alla lettera *b)*, informazioni sulle modalità per la notifica delle reazioni avverse gravi.

2. In conformità ai disposti di cui all'articolo 11 del decreto legislativo 6 novembre 2007, n. 191, l'istituto dei tessuti:

a) predispone procedure per comunicare tempestivamente alla rispettiva autorità regionale e al CNT o al Centro nazionale sangue, in seguito indicato come «CNS», o all'Istituto superiore di sanità, secondo i rispettivi ambiti di competenza, tutte le informazioni disponibili attinenti alle presunte reazioni avverse gravi di cui al comma 1, lettere *a)* e *b)*;

b) predispone procedure per comunicare tempestivamente alla rispettiva autorità regionale e al CNT o al CNS o all'Istituto superiore di sanità, secondo i rispettivi ambiti di competenza, le conclusioni dell'indagine per analizzare le cause e il conseguente esito.

3. Conformemente ai disposti di cui all'articolo 11 del decreto legislativo 6 novembre 2007, n. 191:

a) la persona responsabile di cui all'articolo 17 dello stesso decreto legislativo, comunica alla rispettiva autorità regionale e al CNT o al CNS, secondo i rispettivi ambiti di competenza, le informazioni incluse nel modello di notifica di cui alla parte A dell'allegato VII;

b) l'istituto dei tessuti notifica alla rispettiva Autorità regionale e al CNT o al CNS, secondo l'ambito di competenza, i provvedimenti adottati per quanto riguarda altri tessuti e cellule interessati, distribuiti a fini di applicazioni sull'uomo;

c) l'istituto dei tessuti notifica alla rispettiva Autorità regionale e al CNT o al CNS, secondo l'ambito di competenza, le conclusioni dell'indagine, fornendo almeno le informazioni di cui alla parte B dell'allegato VII.

Art. 11.

Notifica di eventi avversi gravi

1. In conformità ai disposti di cui all'articolo 11 del decreto legislativo 6 novembre 2007, n. 191:

a) l'organizzazione per l'approvvigionamento e l'istituto dei tessuti predispongono procedure per conservare le registrazioni dei dati e per notificare tempestivamente ogni evento avverso grave che si verifichi durante l'approvvigionamento e possa influire sulla qualità e sicurezza dei tessuti e cellule;

b) l'organizzazione responsabile dell'applicazione sull'uomo di tessuti e cellule predispone procedure per notificare tempestivamente all'istituto dei tessuti di riferimento ogni evento avverso grave che possa influire sulla qualità e sicurezza dei tessuti e cellule;

c) l'istituto dei tessuti fornisce all'organizzazione responsabile dell'applicazione sull'uomo informazioni sulle modalità per notificargli eventi avversi gravi che possano influire sulla qualità e sicurezza dei tessuti e cellule.

2. In materia di riproduzione assistita si considera evento avverso grave ogni tipo di errore d'identificazione o di scambio di gameti o embrioni. La persona interessata o l'organizzazione per l'approvvigionamento o l'organizzazione responsabile dell'applicazione sull'uomo riferiscono tali eventi all'istituto dei tessuti fornitore ai fini dell'indagine e notifica all'autorità competente.

3. In conformità ai disposti di cui all'articolo 11 del decreto legislativo 6 novembre 2007, n. 191:

a) l'istituto dei tessuti predispone procedure per comunicare tempestivamente alla rispettiva autorità regionale e al CNT o al CNS o all'Istituto Superiore di Sanità, secondo l'ambito di competenza, tutte le informazioni disponibili attinenti ai presunti eventi avversi gravi di cui al comma 1, lettere *a)* e *b)*;

b) l'istituto dei tessuti o le autorità regionali valutano e comunicano al CNT o CNS, o all'Istituto Superiore di Sanità, secondo l'ambito di competenza, l'eventuale implicazione di altre cellule e tessuti, e gli eventuali provvedimenti intrapresi;

c) l'istituto dei tessuti predispone procedure per comunicare tempestivamente alla rispettiva autorità regionale e al CNT o al CNS o all'Istituto Superiore di Sanità, secondo l'ambito di competenza, le conclusioni dell'indagine per analizzare le cause e il conseguente esito;

4. In conformità ai disposti di cui all'articolo 11 del decreto legislativo 6 novembre 2007, n. 191:

a) il responsabile dell'istituto dei tessuti notifica alla rispettiva autorità regionale e al CNT o al CNS, secondo l'ambito di competenza, le informazioni incluse nel modello di notifica di cui alla parte A dell'allegato VIII;

b) l'istituto dei tessuti valuta gli eventi avversi gravi per individuarne le cause evitabili nell'ambito del procedimento;

c) l'istituto dei tessuti notifica alla rispettiva autorità regionale e al CNT o al CNS, secondo l'ambito di competenza, le conclusioni dell'indagine, fornendo almeno le informazioni di cui alla parte B dell'allegato VIII.

Art. 12.

Relazioni annuali

1. Il Ministero della salute entro il 30 giugno di ogni anno presenta alla Commissione una relazione annuale sulle notifiche delle reazioni e degli eventi avversi gravi, relative all'anno precedente, ricevute dalle Autorità competenti. Il Ministero della salute mette tale relazione a disposizione degli istituti dei tessuti.

2. La trasmissione dei dati si attiene alle specifiche del modello di scambio di dati di cui all'allegato IX, parti A e B, e fornisce tutte le informazioni necessarie ad identificare il mittente e a conservare i suoi dati di riferimento.

Art. 13.

Scambio di informazioni fra Autorità competenti

1. Il Ministero della salute pone in atto le iniziative necessarie ad assicurare la comunicazione delle informazioni del caso ai rispettivi organismi degli Stati membri in relazione a reazioni ed eventi avversi gravi, al fine di assicurare l'adozione dei necessari adeguati provvedimenti.

Art. 14.

Rintracciabilità

1. L'istituto dei tessuti pone in atto sistemi efficaci ed accurati per identificare ed etichettare individualmente cellule e tessuti ricevuti e distribuiti.

2. L'istituto dei tessuti e l'organizzazione responsabile dell'applicazione sull'uomo conservano per almeno trenta anni i dati di cui all'allegato X, avvalendosi di un sistema di registrazione adeguato e leggibile.

Art. 15.

Sistema europeo di codifica

1. Conformemente alle previsioni di cui all'articolo 8, comma 6, del decreto legislativo n. 191 del 2007, a ciascun materiale donato è attribuito un codice d'identificazione unico europeo, al fine di garantire un'adeguata identificazione del donatore e la rintracciabilità di tutti i materiali donati, nonché di fornire informazioni sulle caratteristiche e proprietà fondamentali dei tessuti e cellule. Il codice comprende almeno le informazioni di cui all'allegato XI.

Art. 16.

Recepimento

1. Le regioni e le province autonome di Trento e Bolzano attuano con proprio provvedimento le disposizioni di cui al presente decreto.

2. Con accordo da sancire in sede di Conferenza Stato-regioni sono stabilite le modalità e procedure necessarie a conformarsi alle prescrizioni dell'articolo 15.

Art. 17.

Clausola di cedevolezza

1. In relazione a quanto disposto dall'articolo 117, quinto comma, della Costituzione e dall'articolo 16, comma 3, della legge 4 febbraio 2005, n. 11, le disposizioni del presente decreto legislativo riguardanti ambiti di competenza legislativa delle regioni e delle province autonome di Trento e di Bolzano si applicano, nell'esercizio del potere sostitutivo dello Stato e con carattere di cedevolezza, a decorrere dalla data di entrata in vigore del presente decreto legislativo nelle regioni e nelle province autonome nelle quali non sia ancora adottata la normativa di attuazione regionale o provinciale e perdono comunque efficacia dalla data di entrata in vigore di quest'ultima, fermi restando i principi fondamentali ai sensi dell'articolo 117, comma terzo, della Costituzione.

Art. 18.

Disposizioni finanziarie

1. Ai nuovi o maggiori oneri di cui all'articolo 5 del presente decreto — pari a euro 1.080.000 annui a decorrere dall'anno 2010 — si provvede a valere sulle disponibilità del Fondo di rotazione di cui all'articolo 5 della legge 16 aprile 1987, n. 183, che, a tal fine, vengono versate all'entrata del bilancio dello Stato, per essere successivamente riassegnate ai pertinenti capitoli dello stato di previsione del Ministero della salute.

2. Il Ministro dell'economia e delle finanze è autorizzato ad apportare, con propri decreti, le occorrenti variazioni di bilancio.

3. Le attività previste dalle restanti norme del presente decreto sono svolte dalle amministrazioni competenti con le risorse umane, strumentali e finanziarie disponibili a legislazione vigente.

Il presente decreto, munito del sigillo dello Stato, sarà inserito nella Raccolta ufficiale degli atti normativi della Repubblica italiana. È fatto obbligo a chiunque spetti di osservarlo e di farlo osservare.

Dato a Roma, addì 25 gennaio 2010

NAPOLITANO

BERLUSCONI, *Presidente del Consiglio dei Ministri*

RONCHI, *Ministro per le politiche europee*

FAZIO, *Ministro della salute*

FRATTINI, *Ministro degli affari esteri*

ALFANO, *Ministro della giustizia*

TREMONTI, *Ministro dell'economia e delle finanze*

FITTO, *Ministro per i rapporti con le regioni*

Visto, *il Guardasigilli:* ALFANO

UNIMORE
UNIVERSITÀ DEGLI STUDI DI MODENA E REGGIO EMILIA

Dipartimento Integrato di Neuroscienze
Unità Operativa di Neurologia
Ospedale Civile Sant'Agostino Estense

SERVIZIO SANITARIO REGIONALE
EMILIA-ROMAGNA
Azienda Ospedaliero - Universitaria di Modena

Via Giardini, 1355
Baggiovara
41126 Modena
tel. : 059396111
fax.: 0593962409

17.4 ITALIAN VERSION OF THE ALSFRS-R

1. LINGUAGGIO

Normale processo fonatorio	4
Alterazione evidenziabile del linguaggio	3
Intelligibile con ripetizioni	2
Linguaggio associato a comunicazione non vocale	1
Perdita di linguaggio utile	0

2. **SALIVAZIONE**

Normale	4
Lieve ma definito eccesso di saliva nella bocca; può avere una perdita notturna	3
Saliva moderatamente eccessiva; può avere una perdita minima	2
Marcato eccesso di saliva con una certa perdita	1
Marcata perdita; richiede costantemente l'uso di fazzoletti	0

3. **DEGLUTIZIONE**

Normali abitudini alimentari	4
Iniziali problemi alimentari – occasionalmente va per traverso	3
Modificazioni della consistenza della dieta	2
Necessita di alimentazione enterale supplementare	1
Non in grado di deglutire (alimentazione esclusivamente parenterale o enterale)	0

4 SCRIVERE A MANO (si consideri la mano dominante prima dell'esordio della SLA)

Normale	4
Rallentato o approssimato: tutte le parole sono leggibili	3
Non tutte le parole sono leggibili	2
In grado di afferrare la penna ma incapace di scrivere	1
Incapace di afferrare la penna	0

5a.TAGLIARE IL CIBO E USARE UTENSILI (pazienti senza gastrostomia)

Normale	4
Talvolta rallentato e goffo, ma non richiede aiuto	3
Può tagliare la maggior parte dei cibi, anche se in modo rallentato e goffo; è necessario un certo aiuto	2
Il cibo deve essere tagliato da altri, ma riesce ancora a portarsi il cibo alla bocca da solo	1
Deve essere nutrito	0

5b.TAGLIARE IL CIBO E USARE UTENSILI (pazienti con gastrostomia)

Via Giardini, 1355
Baggiovara
41126 Modena
tel. : 059396111
fax.: 0593962409

Normale	4
Maldestro ma in grado di eseguire tutte le manipolazioni da solo	3
Necessario un certo aiuto con dispositivi di fissaggio	2
Fornisce una minima assistenza a chi lo aiuta	1
Incapace di eseguire qualsiasi aspetto di questi compiti	0

6 VESTIRSI E IGIENE

Funzione normale	4
Bada a se stesso in modo indipendente e completo con sforzo e ridotta efficienza	3
Assistenza intermittente o metodi sostitutivi	2
Necessita di aiuto per la cura del sé	1
Dipendenza totale	0

7. GIRARSI NEL LETTO E AGGIUSTARE LE COPERTE

Normale	4
Talvolta rallentato e goffo; ma non è necessario aiuto	3
Può girarsi da solo o mettere a posto le coperte ma con grande difficoltà	2
Può iniziare il movimento, ma non girarsi o mettere a posto le coperte da solo	1
Necessita di aiuto totale	0

8. CAMMINARE

Normale	4
Iniziali difficoltà di deambulazione	3
Cammina con assistenza (qualsiasi ausilio per la deambulazione comprese ortesi per la caviglia)	2
Solo movimenti funzionali che non portano alla deambulazione	1
Nessun movimento utile degli arti inferiori	0

9. SALIRE LE SCALE

Normale	4
Rallentato	3
Lieve instabilità o fatica	2
Necessita di assistenza (compreso il mancorrente)	1
Non può farlo	0

10. DISPNEA

Nessuna	4
Dispnea quando cammina	3
Dispnea nelle attività della vita quotidiana (mangiare, lavarsi vestirsi)	2
Dispnea a riposo, difficoltà a respirare da seduti o sdraiati	1
Dispnea rilevante, considerare l'uso di supporto respiratorio meccanico	0

Via Giardini, 1355
Baggiovara
41126 Modena
tel. : 059396111
fax.: 0593962409

11. ORTOPNEA

Nessuna	4
Qualche difficoltà nel dormire la notte, non usa più di due cuscini	3
Necessità di un cuscino aggiuntivo per dormire (più di due cuscini)	2
Può dormire solo seduto	1
Non riesce a dormire	0

12. INSUFFICIENZA RESPIRATORIA

Nessuna	4
Uso intermittente di BiPAP	3
Uso continuo di BiPAP la notte	2
Uso continuo di BiPAP la notte e il giorno	1
Ventilazione meccanica invasiva mediante intubazione o tracheostomia	0

TOTALE ____/48

17.5 ITALIAN VERSION OF ALSAQ-40

Le seguenti affermazioni si riferiscono ad alcune difficoltà che lei potrebbe aver riscontrato durante le ultime due settimane. Indichi per favore, ponendo una crocetta sulla casella appropriata, come si è sentito riguardo le seguenti affermazioni.
Se lei non può compiere affatto l'azione indicata dall'affermazione, la preghiamo di segnare la casella corrispondente a: ***Sempre / Non posso farlo affatto***

Quanto spesso durante le ultime due settimane ha pensato che le seguenti affermazioni fossero vere?

Ponga per favore una crocetta nella casella corrispondente ad ogni affermazione

	Mai	Raramente	Qualche Volta	Spesso	Sempre / non posso farlo affatto
1) Ho avuto difficoltà nel camminare anche per brevi distanze, per esempio in casa	|__|	|__|	|__|	|__|	|__|
2) Sono caduto/a e mentre camminavo	|__|	|__|	|__|	|__|	|__|
3) Sono inciampato/a mentre camminavo	|__|	|__|	|__|	|__|	|__|
4) Ho perso l'equilibrio mentre camminavo	|__|	|__|	|__|	|__|	|__|
5) Ho dovuto concentrarmi nel camminare	|__|	|__|	|__|	|__|	|__|
6) Camminare mi ha stancato moltissimo	|__|	|__|	|__|	|__|	|__|
7) Ho sentito male alle gambe mentre camminavo	|__|	|__|	|__|	|__|	|__|
8) Ho avuto difficoltà nel salire e scendere le scale	|__|	|__|	|__|	|__|	|__|
9) Ho trovato difficile stare in piedi	|__|	|__|	|__|	|__|	|__|
10) Ho avuto difficoltà nell'alzarmi dalle sedie	|__|	|__|	|__|	|__|	|__|

Via Giardini, 1355
Baggiovara
41126 Modena
tel. : 059396111
fax.: 0593962409

11) Ho avuto difficoltà nell' usare le braccia e le mani	|__|	|__|	|__|	|__|	|__|
12) Ho avuto difficoltà nel girarmi e muovermi nel letto	|__|	|__|	|__|	|__|	|__|
13) Ho avuto difficoltà nell'afferrare gli oggetti	|__|	|__|	|__|	|__|	|__|
14) Ho avuto difficoltà nel tenere in mano libri o giornali,o girarne le pagine	|__|	|__|	|__|	|__|	|__|
15) Ho avuto difficoltà nello scrivere con chiarezza	|__|	|__|	|__|	|__|	|__|
16) Ho avuto difficoltà nel fare i lavori in casa	|__|	|__|	|__|	|__|	|__|
17) Ho avuto difficoltà nel mangiare con le posate	|__|	|__|	|__|	|__|	|__|
18) Ho avuto difficoltà nel pettinarmi o nel lavarmi i denti	|__|	|__|	|__|	|__|	|__|
19) Ho avuto difficoltà nel vestirmi	|__|	|__|	|__|	|__|	|__|
20) Ho avuto difficoltà nel lavarmi sul lavandino del bagno	|__|	|__|	|__|	|__|	|__|
21) Ho avuto difficoltà nel deglutire	|__|	|__|	|__|	|__|	|__|
22) Ho avuto difficoltà nel mangiare cibi solidi	|__|	|__|	|__|	|__|	|__|
23) Ho avuto difficoltà nel bere bevande liquide	|__|	|__|	|__|	|__|	|__|
24) Ho avuto difficoltà nel partecipare alle conversazioni	|__|	|__|	|__|	|__|	|__|
25) Credo che non sia stato facile capirmi quando parlavo	|__|	|__|	|__|	|__|	|__|

Via Giardini, 1355
Baggiovara
41126 Modena
tel. : 059396111
fax.: 0593962409

26) Ho balbettato o detto parole non comprensibili |__| |__| |__| |__| |__|

27) Ho dovuto parlare molto lentamente |__| |__| |__| |__| |__|

28) Ho parlato meno di quanto fossi solito/a fare |__| |__| |__| |__| |__|

29) Mi sono sentito/a frustrato/a a causa del mio modo di parlare |__| |__| |__| |__| |__|

30) Mi sono sentito/a a disagio a causa del mio modo di parlare |__| |__| |__| |__| |__|

31) Mi sono sentito/a solo/a |__| |__| |__| |__| |__|

32) Mi sono annoiato/a |__| |__| |__| |__| |__|

33) Mi sono sentito/a imbarazzato/a in alcune situazioni sociali |__| |__| |__| |__| |__|

34) Mi sono sentito/a scoraggiato/a per il futuro |__| |__| |__| |__| |__|

35) Ho temuto di essere un peso per gli altri |__| |__| |__| |__| |__|

36) Mi sono sentito/a senza motivazioni per andare avanti |__| |__| |__| |__| |__|

37) Ho provato rabbia a causa della mia malattia |__| |__| |__| |__| |__|

38) Mi sono sentito depresso/a |__| |__| |__| |__| |__|

39) Mi sono preoccupato/a di come la malattia potrebbe essere nel futuro |__| |__| |__| |__| |__|

40) Mi sono sentito/a privato/a della mia libertà |__| |__| |__| |__| |__|

Per favore si assicuri di aver segnato una casella per ciascuna domanda.

GRAZIE DI AVER COMPILATO QUESTO QUESTIONARIO.

Dipartimento Integrato di Neuroscienze
Unità Operativa di Neurologia
Ospedale Civile Sant'Agostino Estense

SERVIZIO SANITARIO REGIONALE
EMILIA-ROMAGNA
Azienda Ospedaliero - Universitaria di Modena

Via Giardini, 1355
Baggiovara
41126 Modena
tel. : 059396111
fax.: 0593962409

17.6 ITALIAN VERSION OF MRC

**Arti superiori**	Dx	Sn
Deltoide		
Bicipite brachiale		
Tricipite brachiale		
Flessione polso		
Estensione polso		
Opposizione pollice		
Flessione dita		
Estensione dita		

**Arti inferiori**	Dx	Sn
Ileopsoas		
Quadricipite		
Bicipite femorale		
Tibiale anteriore		
Gemelli		
Flessione dita		
Estensione dita		

**Collo**	
Flessione	
Estensione	

PUNTEGGI:
5/5 alla scala MRC: movimento possibile contro resistenza massima;
4/5 alla scala MRC: movimento possibile solo contro resistenza minima;
3/5 alla scala MRC: movimento possibile solo contro gravità;
2/5 alla scala MRC: movimento possibile solo in assenza di gravità;
1/5 alla scala MRC: accenno al movimento;
0/5 alla scala MRC: assenza di movimento;
