# Supplementary material for: Immune effects of faecal microbiota transplantation in amyotrophic lateral sclerosis: a pilot multicentre, randomised, masked, sham-controlled, proof-of concept trial
Source: eBioMedicine. 2026 Oct 8;133:106509. doi: 10.1016/j.ebiom.2026.106509 (PMC13676721; doi:10.1016/j.ebiom.2026.106509)
Supplement: Supplementary file 2 [file mmc1.docx]

# Supplementary appendix

This appendix formed part of the original submission and has been peer reviewed. We post it as supplied by the authors.

**Supplement to: Zucchi E, Niccolai E, De Maio F, et al.**

**Immune effects of faecal microbiota transplantation in amyotrophic lateral sclerosis: a pilot multicentre, randomised, double blind controlled, proof-of-concept trial**

**Immune effects of faecal microbiota transplantation in amyotrophic lateral sclerosis: a pilot multicentre, randomised, double blind controlled, proof-of-concept trial**

Elisabetta Zucchi^1,2^*, Elena Niccolai^3^*, Flavio De Maio^4^*, Riccardo Cuoghi Costantini^5,6^*, Gianluca Quaranta^4^, Stefano Bibbò^7^, Ilaria Martinelli^2^, Giulia Gianferrari^1,2^, Cecilia Simonini^8^, Giulia Nannini^3^, Matteo Lulli^9^, Simone Baldi^3^, Gianluca Bartolucci^10^, Roberto Vicini^5^, FETR-ALS Study Group, Margherita Capasso^11^, Giulia Bisogni^12^, Amelia Conte^12^, Paola Brunori^13^, Giovanni Cammarota^7^, Sabrina Paganoni^14-15^, Roberto D’Amico^5,6#^, Amedeo Amedei^3#^, Luca Masucci^4,16#^, Jessica Mandrioli^1,2#§^

^1^Department of Biomedical, Metabolic and Neural Sciences, University of Modena and Reggio Emilia, Modena, Italy.

^2^Neurology Unit, Department of Neuroscience, Azienda Ospedaliero Universitaria Di Modena, Modena, Italy.

^3^Department of Experimental and Clinical Medicine, University of Florence, Florence, Italy.

^4^Department of Laboratory Sciences and Haematology, Fondazione Policlinico Universitario Gemelli IRCCS, Rome, Italy.

^5^Unit of Statistical and Methodological Support to Clinical Research, Azienda Ospedaliero-Universitaria, Modena 41121, Italy.

^6^Department of Medical and Surgical Sciences for Children and Adults, University of Modena and Reggio Emilia, Modena 41124, Italy.

^7^Department of Medical and Surgical Sciences, UOC Gastroenterologia, Fondazione Policlinico Universitario Gemelli IRCCS, Rome, Italy.

^8^Transfusion Medicine Unit, Azienda USL-IRCCS di Reggio Emilia, Reggio Emilia, Italy.

^9^Department of Experimental and Clinical Biomedical Sciences, Biochemical Sciences Section, University of Florence, Florence, Italy

^10^Department of Neurosciences, Psychology, Drug Research and Child Health Section of Pharmaceutical and Nutraceutical Sciences, University of Florence, Florence, Italy.

^11^Neuromuscular Center, Clinica Neurologica, Ospedale "S.S. Annunziata", Chieti, Italy.

^12^ Centro Clinico Nemo “Armida Barelli”- Fondazione Serena Onlus; Fondazione Policlinico Universitario Agostino Gemelli IRCCS, Rome, Italy.

^13^Department of Neurophysiopathology, Perugia Hospital, Perugia, Italy.

^14^Department of Neurology, Sean M. Healey and AMG Center for ALS and the Neurological Clinical Research Institute, Massachusetts General Hospital, Harvard Medical School, Boston, Massachusetts, USA.

^15^Spaulding Rehabilitation Hospital, Boston, Massachusetts, USA

^16^Department of Basic, Clinical, Intensive and Perioperative Biotechnology Sciences, Fondazione Policlinico Universitario Agostino Gemelli IRCCS, Rome, Italy.

*Contributed equally

^#^Contributed equally

^§^Corresponding author

# Table of contents

1. FETR-ALS study group	5

Clinical trial sites and investigators	5

FETR-ALS study group	5

2. Supplementary tables	6

Table 1. Biological features (blood and CSF samples) of patients at baseline	6

Table 2 Biological features (stool, saliva and intestinal samples) of patients at baseline	8

Table 3. Patients showing a positive response comparing baseline and month 6

between FMT and SP arms	10

Table 4. Changes from baseline to month 6 and 12 in Treg cells across treatment arms	10

Table 5. Average monthly variations in circulating and tissue Treg in FMT and SP

Patients	10

Table 6. Primary and secondary efficacy outcomes at month 6 in the intention-to-treat population	11

Table 7 Average ALSFRS-r (total scores and subscores) per treatment arm during

FETR-ALS trial in intention-to-treat population..	12

Table 8 Mean differences between FMT and SP arms in ALSFRS-r total scores and subscores at month 6 and month 12 (assessed as changes with respect to baseline scores).

Intention-to-treat analysis.	12

Table 9 Average monthly variations in ALSFRS-r and its subscores in FMT and SP arms, and the mean differences over time between the two arms, in

intention-to-treat population.	13

Table 10. Average decline in ALSFRS-r total score and subscores in segmented

repeated-measures analysis in SP and FMT arms, from baseline to month 12	14

Table 11. Average monthly decline in ALSFRS-r total score in segmented

repeated-measures analysis in SP and FMT arms, from onset to month 12	15

Table 12. Mean differences between FMT and SP arms in clinical outcome measures

from baseline to month 6 and month 12. Intention-to-treat analysis.	15

Table 13. Average monthly variations in clinical outcome measures in FMT and

SP arms, and the mean differences over time between the two arms	16

Table 14. Treatment-emergent adverse events between month 6 and month 12, by treatment arm

and categorized according to MedDRA classification. Intention-to treat population.	17

Table 15: Treatment-emergent adverse events between baseline and month 12, by treatment arm

and categorized according to MedDRA classification. Intention-to treat population.	18

Table 16 Mean changes in biological outcomes between FMT and SP arm from baseline

to month 6 an d12. Intention-to-treat analysis	19

Table 17 Average variations of neurofilaments over time in FMT and SP arms, and

the mean difference between the two arms, as estimated by longitudinal models.	19

Table 18. Changes from baseline to month 6 and 12 in Treg fractions over Th1 and Th17

immune cells subpopulations across treatment arms	20

Table 19. Mean changes in circulating Treg/Th1 and Treg/Th17 ratios in FMT and SP arms	20

Table 20. Changes from baseline to month 6 and 12 of all circulating immune cell

subpopulations across treatment arms	21

Table 21. Mean variations over time of circulating T cell subpopulations in FMT and SP arms	23

Table 22. Changes from baseline to month 6 and 12 of intestinal immune cell

subpopulations across treatment arms	25

Table 23. Mean variations of intestinal immune cells subpopulations at baseline

and over time, in FMT and SP arms, and from the comparison of the treatment arms	27

Table 24. Changes from baseline to month 6 and 12 of serum cytokines across treatment arms	29

Table 25. Average monthly changes of serum cytokines in the SP and the FMT arms,

and their ratio over time	31

Table 26. Changes from baseline to month 6 and 12 of salivary cytokines across treatment arms	33

Table 27. Average monthly changes of salivary cytokines in the SP and FMT arms,

and their ratio over time	35

Table 28. Changes from baseline to month 6 and 12 of homogenized intestinal tissue

cytokines across treatment arms. Intention to treat analysis	37

Table 29. Average monthly changes of homogenized intestinal tissue sample cytokines

in the SP and FMT arms, and their ratio over time	39

Table 30. Changes from baseline to month 6 and 12 of faecal cytokines across treatment arms	41

Table 31. Average monthly changes of faecal cytokines in the SP and FMT arms, and

their ratio over time	43

Table 32 Mean changes in serum free fatty acids from baseline to month 6 and month

12 across FMT and SP arms.	45

Table 33. Average monthly changes of serum free fatty acid in the SP and FMT arms,

and their mean difference over time	47

Table 34. Relative abundances at Phylum level at baseline and month 6, in FMT and SP arms	48

Table 35. Relative abundances at Genus level (top 20 genera) in FMT and SP arms	49

3. Supplementary figures	50

Figure 1. Variations in Tregs over time in the two treatment arms	50

Figure 2. Variations in ALSFRS-R and FVC over time in the two treatment arms .	51

Figure 3. Variations in BMI and ALSAQ40 over time in the two treatment arms	52

Figure 4. Tracheostomy-free survival rates in the two treatment arms	53

Figure 5. Safety analyses up to 6 months	54

Figure 6. Safety analyses over the full 12-month follow-up	55

Figure 7. Variations in neurofilament over time in the two treatment arms	56

Figure 8: Variations in T cells over time in the two treatment arms	57

Figure 9: Variations in the ratios of circulating T cells over time in the two treatment arms	58

Figure 10. Comparison of gut microbiota alpha diversity metrics at baseline and at month 6 in SP

and FMT arms	59

Figure 11. Comparison of Bray-Curtis beta diversity of gut microbiota composition in SP and FMT

arms at baseline and at month 6	60

Figure 12. Assessment of dispersion of gut microbiota communities at baseline and at month 6 in

SP and FMT arms	61

Figure 13. Relative abundance of the bacterial phyla at baseline and at month 6 in SP and FMT arms	62

Figure 14. Relative abundance of the top 20 genera at baseline and at month 6 in SP and FMT arms	63

Figure 15. Differential abundance analysis of bacterial taxa at baseline and month 6 in FMT and

SP arms	64

Figure 16. Microbial community composition differences across donors and SP and FMT arms	65

#### 1. FETR-ALS clinical trial sites and investigators

| **Trial sites and location** | **Principal**  **investigators** | **Sub-investigators and collaborators** |
| --- | --- | --- |
| **Modena** |  |  |
| ALS Center, Azienda Ospedaliero-Universitaria di Modena, Modena, Italy | Jessica Mandrioli | Nicola Fini, Ilaria Martinelli, M.D. Elisabetta Zucchi, M.D.  Giulia Gianferrari, M.D. Andrea Ghezzi M.D. Cecilia Simonini, BSc Roberta Bedin, BSc Annalisa Gessani, S.L.P.  Francesca Prompicai, R.N. Silvia Parisi, R.N. |
| **Rome** |  |  |
| Centro Clinico NeMO Roma. Rome, Italy | Mario Sabatelli | Amelia Conte, Giulia Bisogni |
| **Perugia** |  |  |
| Azienda Ospedaliera di Perugia, Perugia, Italy | Paola Brunori | Teresa Anna Cantisani, Rossella Papetti |
| **Chieti** |  |  |
| Neuromuscular Unit, Ospedale "SS Annunziata" di Chieti, Chieti, Italy | Margherita Capasso | Laura Ferri, Paola Ajdinaj |

#### FETR-ALS study group:

1) ALS Center, Neurology Unit, Azienda Ospedaliero Universitaria di Modena; Department of Biomedical, Metabolic and Neural Sciences, University of Modena and Reggio Emilia, Modena, Italy: Jessica Mandrioli, Nicola Fini, Ilaria Martinelli, Elisabetta Zucchi, Giulia Gianferrari, Andrea Ghezzi, Cecilia Simonini, Roberta Bedin, Annalisa Gessani, Francesca Prompicai, Silvia Parisi
2) Unit of Statistical and Methodological Support to Clinical Research, Azienda Ospedaliero-Universitaria di Modena; Department of Medical and Surgical Sciences for Children and Adults, University of Modena and Reggio Emilia, Modena, Italy: Riccardo Cuoghi Costantini, Roberto Vicini, Roberrto D’Amico
3) Laboratory of Immunology, Department of Experimental and Clinical Medicine, University of Florence, Florence: Elena Niccolai, Amedeo Amedei, Giulia Nannini, Matteo Lulli, Simone Baldi, Gianluca Bartolucci
4) Department of Laboratory and Haematological Sciences, Fondazione Policlinico Universitario A. Gemelli IRCCS, Rome, Italy Luca Masucci, Gianluca Quaranta, Maurizio Sanguinetti, Alessandra Guarnaccia, Flavio De Maio, Giovanni Fancello, Chiara Agrillo, Vanessa Di Pietro, Desy De Lorenzis, Roberto Rosato, Francesca Romana Monzo
5) Department of Medical and Surgical Sciences, UOC Gastroenterologia, Fondazione Policlinico Universitario Gemelli IRCCS, Rome, Italy Giovanni Cammarota, Stefano Bibbò,Antonio Gasbarrini, Irene Venturini, Debora Rondinella, Nausicaa Antonini, Chiara Chiarini, Regina Manica, Micaela Angeletti
6) Department of Emergency, Intensive Care Medicine and Anesthesia, Fondazione Policlinico Universitario A. Gemelli IRCCS, Rome, Italy: Marco Rossi, Tommaso Cosimo Caputo
7) Centro Clinico Nemo “Armida Barelli”- Fondazione Serena Onlus, Fondazione Policlinico A. Gemelli, Rome; Institute of Neurology, Catholic University of the Sacred Heart, Fondazione Policlinico A. Gemelli, Rome: Mario Sabatelli, Amelia Conte, Giulia Bisogni
8) U.O. Neurofisiopatologia, Azienda Ospedaliera di Perugia: Teresa Anna Cantisani, Paola Brunori, Rosella Papetti
9) Neuromuscular Center, Clinica Neurologica, Ospedale "S.S. Annunziata", Chieti: Margherita Capasso, Laura Ferri, Paola Ajdinaj

# 2. Supplementary Tables

#### Supplementary Table 1. Biological features (blood and CSF samples) of patients enrolled in the two trial arms at baseline.·

|  | | **SP** | | | **FMT** | | | |
| --- | --- | --- | --- | --- | --- | --- | --- | --- |
|  |  | **(n = 14)** | | | **(n = 28)** | | | |
|  |  | Sample type | n | mean | SD | n | mean | SD |
| Treg (%CD4+CD25+CD127-FoxP3+) | % | PBMC | 14 | 4·84 | 2·03 | 28 | 4·81 | 2·74 |
| CD45+ (% over live) | % | PBMC | 14 | 67·76 | 11·63 | 28 | 63·19 | 12·92 |
| CD3+ (% over CD45+) | % | PBMC | 14 | 52·85 | 20·74 | 28 | 61·61 | 17·32 |
| CD4+ (% over CD45+) | % | PBMC | 14 | 38·14 | 11·06 | 28 | 47·40 | 14·61 |
| CD8+CD4+ (% over CD3) | % | PBMC | 14 | 0·49 | 0·41 | 28 | 0·87 | 1·24 |
| CD4+ (% over CD3) | % | PBMC | 14 | 63·37 | 11·07 | 28 | 65·99 | 14·28 |
| Th0 (% over CD4+) | % | PBMC | 14 | 4·06 | 3·66 | 28 | 4·46 | 7·57 |
| CxCR3+ (% over CD4+) | % | PBMC | 14 | 3·17 | 2·91 | 28 | 4·73 | 11·12 |
| Th1 (% over CD4+) | % | PBMC | 14 | 2·33 | 2·12 | 28 | 1·87 | 3·68 |
| Th17 (% over CD4+) | % | PBMC | 14 | 16·56 | 24·92 | 28 | 16·9 | 23·20 |
| Th1/Th17 (% over CD4+) | % | PBMC | 14 | 0·47 | 0·54 | 28 | 0·43 | 0·70 |
| Ratio of circulating Treg/Th1 |  | PBMC | 14 | 12·82 | 33·06 | 28 | 38·33 | 129·67 |
| Ratio of circulating Treg/Th17 |  | PBMC | 14 | 0·61 | 0·40 | 28 | 1·06 | 1·47 |
| CCR4+ (% over CD4+) | % | PBMC | 14 | 60·03 | 26·16 | 28 | 56·68 | 27·28 |
| Th2 (% over CD4+) | % | PBMC | 14 | 41·12 | 24·9 | 28 | 36·57 | 25·60 |
| Th9 (% over CD4+) | % | PBMC | 14 | 3·27 | 2·89 | 28 | 5·69 | 5·66 |
| CCR6+ CCR4+ (% over CD4+) | % | PBMC | 14 | 18·51 | 24·67 | 28 | 20·66 | 25·40 |
| Th22 (% over CD4+) | % | PBMC | 14 | 1·44 | 1·08 | 28 | 1·54 | 1·19 |
| CD8+ (% over CD3) | % | PBMC | 14 | 27·87 | 9·20 | 28 | 26·98 | 11·32 |
| Tc0 (% over CD8+) | % | PBMC | 14 | 6·23 | 5·69 | 28 | 5·31 | 7·84 |
| CXCR3 (% over CD8+) | % | PBMC | 14 | 2·82 | 3·00 | 28 | 6·26 | 12·28 |
| Tc1/Tc17 (% over CD8+) | % | PBMC | 14 | 1·28 | 1·83 | 28 | 1·67 | 2·60 |
| Tc1 (% over CD8+) | % | PBMC | 14 | 1·13 | 1·14 | 28 | 2·33 | 7·80 |
| CCR4+ (% over CD8+) | % | PBMC | 14 | 56·53 | 33·11 | 28 | 55·36 | 34·42 |
| Tc17 (% over CD8+) | % | PBMC | 14 | 26·11 | 30·12 | 28 | 28·92 | 27·10 |
| Tc2 (% over CD8+) | % | PBMC | 14 | 27·58 | 29·46 | 28 | 24·12 | 30·07 |
| Tc9 (% over CD8+) | % | PBMC | 14 | 26·1 | 34·48 | 28 | 25·56 | 29·45 |
| CCR4+ CCR6+ (% over CD8+) | % | PBMC | 14 | 34·64 | 29·81 | 28 | 35·18 | 29·42 |
| Tc22 (% over CD8+) | % | PBMC | 14 | 0·66 | 0·65 | 28 | 0·58 | 0·54 |
| MIP-1β | pg/ml | serum | 14 | 29·07 | 26·18 | 28 | 32·08 | 23·63 |
| IL-6 | pg/ml | serum | 14 | 0·36 | 0·39 | 28 | 0·26 | 0·35 |
| IFNg | pg/ml | serum | 14 | 0·41 | 0·33 | 28 | 0·55 | 0·53 |
| IL-5 | pg/ml | serum | 14 | 0·08 | 0·31 | 28 | <LLOQ^§^ | <LLOQ^§^ |
| GM-CSF | pg/ml | serum | 14 | <LLOQ^§^ | <LLOQ^§^ | 28 | 0·11 | 0·6 |
| TNFalfa | pg/ml | serum | 14 | 2·35 | 2·05 | 28 | 2·23 | 2·44 |
| IL-2 | pg/ml | serum | 14 | 0·01 | 0·05 | 28 | <LLOQ^§^ | <LLOQ^§^ |
| IL-1β | pg/ml | serum | 13 | 0·01 | 0·04 | 28 | 0·06 | 0·1 |
| IL-13 | pg/ml | serum | 14 | <LLOQ^§^ | <LLOQ^§^ | 28 | <LLOQ^§^ | <LLOQ^§^ |
| IL-4 | pg/ml | serum | 14 | <LLOQ^§^ | <LLOQ^§^ | 28 | <LLOQ^§^ | <LLOQ^§^ |
| MCP-1 | pg/ml | serum | 14 | 28·35 | 11·31 | 28 | 34·08 | 17·1 |
| IL-8 | pg/ml | serum | 14 | 57·67 | 113·44 | 28 | 24·4 | 46·4 |
| IL-10 | pg/ml | serum | 14 | 0·39 | 1·04 | 26 | 0·11 | 0·13 |
| G-CSF | pg/ml | serum | 14 | 0·24 | 0·89 | 27 | 0·1 | 0·51 |
| IL-7 | pg/ml | serum | 14 | 2·33 | 1·83 | 26 | 2·41 | 3·44 |
| IL-12p70 | pg/ml | serum | 14 | 0·48 | 0·78 | 27 | 2·31 | 9·34 |
| IL-17 | pg/ml | serum | 13 | 0·03 | 0·07 | 25 | 0·17 | 0·63 |
| VEGF | pg/ml | serum | 14 | <LLOQ^§^ | <LLOQ^§^ | 28 | <LLOQ^§^ | <LLOQ^§^ |
| MIP-1α | pg/ml | serum | 14 | 4·46 | 7·46 | 28 | 2·82 | 2·07 |
| Creatinine | mg/dl | serum | 14 | 0·71 | 0·16 | 28 | 0·63 | 0·15 |
| CRP | mg/dl | serum | 14 | 0·52 | 0·06 | 28 | 0·54 | 0·37 |
| Albumin | g/dl | serum | 14 | 4·32 | 0·17 | 28 | 4·30 | 0·32 |
| Total Cholesterol | mg/dl | serum | 14 | 241·21 | 49·54 | 28 | 241·75 | 55·61 |
| LDL Cholesterol | mg/dl | serum | 14 | 154·00 | 40·63 | 28 | 162·26 | 42·11 |
| Triglycerides | mg/dl | serum | 14 | 130·14 | 46·41 | 28 | 122·54 | 58·34 |
| Vitamin D | ng/ml | serum | 14 | 22·76 | 11·55 | 28 | 28·98 | 21·66 |
| pNfH | pg/ml | serum | 14 | 3528·51 | 2950·53 | 28 | 2797·04 | 27·42·84 |
| NfL | pg/ml | serum | 14 | 153·13 | 88·15 | 28 | 149·16 | 119·81 |
| pNfH | pg/ml | CSF | 11 | 5964·00 | 5990·75 | 24 | 8321·25 | 5597·03 |
| NfL | pg/ml | CSF | 11 | 6590·50 | 4592·20 | 24 | 6669·75 | 2384·57 |
| Acetic Acid | μmol/L | serum | 14 | 166·21 | 45·60 | 28 | 166·72 | 51·07 |
| Propionic Acid | μmol/L | serum | 14 | 7·55 | 5·61 | 28 | 6·40 | 2·83 |
| IsoButiric Acid | μmol/L | serum | 14 | 14·85 | 3·56 | 28 | 16·01 | 5·75 |
| Butiric Acid | μmol/L | serum | 14 | 0·88 | 0·22 | 28 | 1·18 | 0·64 |

|  | | **SP** | | | **FMT** | | | |
| --- | --- | --- | --- | --- | --- | --- | --- | --- |
|  |  | **(n = 14)** | | | **(n = 28)** | | | |
|  |  | Sample type | n | mean | SD | n | mean | SD |
| isoValeric Acid | μmol/L | serum | 14 | 28·88 | 10·51 | 28 | 31·37 | 14·25 |
| 2MetilButiric Acid | μmol/L | serum | 14 | 9·71 | 4·22 | 28 | 10·58 | 5·85 |
| Hexanoic Acid | μmol/L | serum | 14 | 0·95 | 0·24 | 28 | 0·65 | 0·00 |
| Octanoic Acid | μmol/L | serum | 14 | 16·54 | 38·81 | 28 | 0·67 | 0·00 |
| Decanoic Acid | μmol/L | serum | 14 | 7·02 | 16·26 | 28 | 1·01 | 0·32 |
| Dodecanoic Acid | μmol/L | serum | 14 | 2·04 | 1·31 | 28 | 7·99 | 21·43 |
| Tetradecanoic Acid | μmol/L | serum | 14 | 25·49 | 10·02 | 28 | 3·70 | 9·19 |
| Hexadecanoic Acid | μmol/L | serum | 14 | 576·29 | 181·71 | 28 | 1·92 | 1·34 |
| Octadecanoic Acid | μmol/L | serum | 14 | 454·97 | 131·44 | 28 | 21·14 | 8·11 |

Values are reported as means with standard deviations between parentheses.

§: Below the lower limit of quantification (LLOQ) of the assay.

Abbreviations: SP: Sham Procedure; FMT: faecal microbiota transplantation; CRP: C-reactive protein; IL: interleukin; MCP-1: monocyte- chemoattractant protein 1. NfL: neurofilament light chain, NfH: neurofilament heavy chain; Treg: T regulatory cells; Th1: T helper type 1; Th17: T helper type 17.

#### Supplementary Table 2. Biological features (stool, saliva and intestinal samples) of patients enrolled in the two trial arms at baseline.

|  | | **SP** | | | | **FMT** | | |
| --- | --- | --- | --- | --- | --- | --- | --- | --- |
|  |  | **(n = 14)** | | | | **(n = 28)** | | |
|  |  | **Sample type** | **n** | **mean** | **SD** | **n** | **mean** | **SD** |
| FOXP3/b2M expression* | % | Dissociated intestinal tissue | 10 | 1·64 | 2·21 | 25 | 1·22 | 1·67 |
| CD3+ (% over CD45+) | % | Dissociated intestinal tissue | 13 | 15·10 | 16·12 | 26 | 13·93 | 11·02 |
| CD8+CD4+ (% over CD3) | % | Dissociated intestinal tissue | 13 | 2·50 | 4·12 | 19 | 1·62 | 2·20 |
| CD4+(% over CD3) | % | Dissociated intestinal tissue | 13 | 71·69 | 20·77 | 26 | 67·55 | 23·33 |
| Th0 (% over CD4+) | % | Dissociated intestinal tissue | 13 | 17·37 | 13·20 | 26 | 17·60 | 11·31 |
| CxCR3+ (% over CD4+) | % | Dissociated intestinal tissue | 13 | 4·70 | 6·84 | 26 | 4·20 | 10·14 |
| Th1/Th17 (% over CD4+) | % | Dissociated intestinal tissue | 13 | 0·21 | 0·38 | 26 | 0·21 | 0·33 |
| Th1 (% over CD4+) | % | Dissociated intestinal tissue | 13 | 2·90 | 3·80 | 26 | 2·44 | 4·49 |
| CCR4+ (% over CD4+) | % | Dissociated intestinal tissue | 13 | 45·38 | 23·75 | 26 | 48·21 | 23·08 |
| Th17 (% over CD4+) | % | Dissociated intestinal tissue | 13 | 4·66 | 7·86 | 26 | 8·39 | 15·12 |
| Th2 (% over CD4+) | % | Dissociated intestinal tissue | 13 | 42·02 | 22·18 | 26 | 40·99 | 20·01 |
| Th9 (% over CD4+) | % | Dissociated intestinal tissue | 13 | 1·37 | 1·96 | 25 | 1·74 | 2·05 |
| CCR6+ CCR4+ (% over CD4+) | % | Dissociated intestinal tissue | 13 | 11·87 | 12·26 | 25 | 16·26 | 17·09 |
| Th22 (% over CD4+) | % | Dissociated intestinal tissue | 13 | 0·33 | 0·54 | 25 | 0·54 | 0·95 |
| CD8+ (% over CD3) | % | Dissociated intestinal tissue | 13 | 16·83 | 18·14 | 26 | 22·13 | 21·59 |
| Tc0 (% over CD8+) | % | Dissociated intestinal tissue | 13 | 13·50 | 8·68 | 25 | 11·09 | 8·57 |
| CXCR3 (% over CD8+) | % | Dissociated intestinal tissue | 13 | 1·41 | 2·21 | 25 | 2·41 | 7·29 |
| Tc1/Tc17 (% over CD8+) | % | Dissociated intestinal tissue | 13 | 0·04 | 0·13 | 24 | 0·13 | 0·59 |
| Tc1 (% over CD8+) | % | Dissociated intestinal tissue | 13 | 0·64 | 0·79 | 25 | 0·71 | 1·85 |
| CCR4+ (% over CD8+) | % | Dissociated intestinal tissue | 13 | 43·41 | 22·96 | 25 | 49·10 | 27·19 |
| Tc17 %(% over CD8+) | % | Dissociated intestinal tissue | 13 | 5·33 | 11·39 | 25 | 12·13 | 19·59 |
| Tc2 (% over CD8+) | % | Dissociated intestinal tissue | 13 | 35·30 | 22·41 | 25 | 34·73 | 23·92 |
| Tc9 (% over CD8+) | % | Dissociated intestinal tissue | 13 | 5·50 | 5·74 | 25 | 5·61 | 6·89 |
| CCR4+ CCR6+ %(% over CD8+) | % | Dissociated intestinal tissue | 13 | 20·90 | 15·33 | 25 | 25·72 | 23·19 |
| Tc22 (% over CD8+) | % | Dissociated intestinal tissue | 13 | 1·05 | 1·14 | 25 | 1·08 | 1·84 |
| MIP-1β | pg/ml | feces | 7 | <LLOQ^§^ | <LLOQ^§^ | 14 | <LLOQ^§^ | <LLOQ^§^ |
| IL-6 | pg/ml | feces | 7 | <LLOQ^§^ | <LLOQ^§^ | 14 | 8·52 | 31·79 |
| IFNg | pg/ml | feces | 7 | 0·80 | 2·03 | 14 | 0·51 | 1·67 |
| IL-5 | pg/ml | feces | 7 | 3·18 | 8·4 | 14 | <LLOQ^§^ | <LLOQ^§^ |
| GM-CSF | pg/ml | feces | 7 | 0·58 | 0·74 | 14 | 7·55 | 26·8 |
| TNFalpha | pg/ml | feces | 7 | 2·58 | 5·67 | 14 | 1·73 | 3·69 |
| IL-2 | pg/ml | feces | 7 | 2·17 | 5·75 | 13 | 2·97 | 5·8 |
| IL-1β | pg/ml | feces | 7 | 24·46 | 63·66 | 14 | 1·03 | 1·89 |
| IL-13 | pg/ml | feces | 7 | <LLOQ^§^ | <LLOQ^§^ | 14 | 0·43 | 1·6 |
| IL-4 | pg/ml | feces | 7 | <LLOQ^§^ | <LLOQ^§^ | 14 | <LLOQ^§^ | <LLOQ^§^ |
| MCP-1 | pg/ml | feces | 7 | 0·56 | 1·49 | 14 | 2·01 | 5·96 |
| IL-8 | pg/ml | feces | 7 | 0·02 | 0·03 | 14 | 0·24 | 0·64 |
| IL-10 | pg/ml | feces | 7 | 0·17 | 0·32 | 14 | 0·75 | 2·08 |
| G-CSF | pg/ml | feces | 5 | 2·15 | 4·29 | 10 | 0·64 | 1·18 |
| IL-7 | pg/ml | feces | 5 | <LLOQ^§^ | <LLOQ^§^ | 10 | <LLOQ^§^ | <LLOQ^§^ |
| IL-12p70 | pg/ml | feces | 7 | 0·27 | 0·66 | 14 | 0·99 | 2·77 |
| IL-17 | pg/ml | feces | 7 | 0·35 | 0·82 | 14 | 3·59 | 12·99 |
| VEGF | pg/ml | feces | 5 | <LLOQ^§^ | <LLOQ^§^ | 10 | <LLOQ^§^ | <LLOQ^§^ |
| MIP-1α | pg/ml | feces | 5 | <LLOQ^§^ | <LLOQ^§^ | 10 | 0·04 | 0·09 |
| IL-15 | pg/ml | feces | 5 | <LLOQ^§^ | <LLOQ^§^ | 10 | <LLOQ^§^ | <LLOQ^§^ |
| MIP-1apha | pg/ml | Homogenized intestinal tissue | 14 | 7·04 | 5·40 | 24 | 5·78 | 6·14 |
| IL-6 | pg/ml | Homogenized intestinal tissue | 14 | 1·14 | 0·86 | 24 | 0·85 | 0·63 |
| IFNg | pg/ml | Homogenized intestinal tissue | 14 | 5·67 | 2·05 | 24 | 5·51 | 2·74 |
| IL-5 | pg/ml | Homogenized intestinal tissue | 14 | 10·15 | 6·27 | 24 | 13·6 | 7·00 |
| GM-CSF | pg/ml | Homogenized intestinal tissue | 14 | 9·23 | 4·14 | 24 | 9·27 | 4·37 |
| TNFalfa | pg/ml | Homogenized intestinal tissue | 14 | 8·66 | 4·07 | 24 | 9·15 | 4·81 |
| IL-2 | pg/ml | Homogenized intestinal tissue | 14 | 1·20 | 0·55 | 24 | 1·45 | 1·31 |
| IL-1β | pg/ml | Homogenized intestinal tissue | 14 | 1·75 | 0·61 | 24 | 2·08 | 1·36 |
| IL-13 | pg/ml | Homogenized intestinal tissue | 14 | 5·72 | 2·87 | 24 | 5·55 | 2·41 |
| IL-4 | pg/ml | Homogenized intestinal tissue | 14 | 0·33 | 0·24 | 24 | 0·35 | 0·30 |
| MCP-1 | pg/ml | Homogenized intestinal tissue | 14 | 19·62 | 12·34 | 24 | 15·85 | 12·30 |
| IL-8 | pg/ml | Homogenized intestinal tissue | 14 | 3·33 | 1·77 | 24 | 2·99 | 2·25 |
| IL-10 | pg/ml | Homogenized intestinal tissue | 14 | 1·59 | 1·04 | 24 | 1·79 | 1·15 |
| G-CSF | pg/ml | Homogenized intestinal tissue | 14 | 16·99 | 13·70 | 24 | 14·84 | 7·73 |
| IL-7 | pg/ml | Homogenized intestinal tissue | 14 | 45·29 | 14·02 | 24 | 47·61 | 18·72 |
| IL-12p70 | pg/ml | Homogenized intestinal tissue | 14 | 12·49 | 6·70 | 24 | 13·47 | 7·65 |
| IL-17 | pg/ml | Homogenized intestinal tissue | 14 | 1·85 | 0·76 | 24 | 1·90 | 0·96 |
| VEGF | pg/ml | Homogenized intestinal tissue | 14 | 41·72 | 37·77 | 26 | 36·34 | 43·14 |
| MIP-1β | pg/ml | Homogenized intestinal tissue | 14 | 51·05 | 38·58 | 26 | 44·44 | 40·60 |
| IL-15 | pg/ml | Homogenized intestinal tissue | 14 | 15·22 | 34·88 | 26 | 32·66 | 49·77 |
| MIP-1β | pg/ml | Saliva | 14 | 2·00 | 2·11 | 22 | 3·86 | 8·38 |

|  | | **SP** | | | | **FMT** | | | |
| --- | --- | --- | --- | --- | --- | --- | --- | --- | --- |
|  |  | **(n = 14)** | | | | **(n = 28)** | | | |
|  |  | **Sample type** |  | **n** | **mean** | **SD** | **n** | **mean** | **SD** |
| IL-6 | pg/ml |  | Saliva | 14 | 1·63 | 2·11 | 22 | 1·56 | 3·46 |
| IFNg | pg/ml |  | Saliva | 14 | 0·59 | 0·59 | 22 | 0·50 | 0·49 |
| IL-5 | pg/ml |  | Saliva | 14 | 0·32 | 0·73 | 22 | 0·19 | 0·69 |
| GM-CSF | pg/ml |  | Saliva | 14 | 0·30 | 0·72 | 22 | 0·13 | 0·34 |
| TNFalpha | pg/ml |  | Saliva | 14 | 14·32 | 15·62 | 22 | 9·84 | 16·05 |
| IL-2 | pg/ml |  | Saliva | 14 | 0·35 | 0·39 | 22 | 0·34 | 0·43 |
| IL-1β | pg/ml |  | Saliva | 14 | 160·01 | 138·32 | 22 | 278·19 | 561·63 |
| VEGF | pg/ml |  | Saliva | 14 | 147·52 | 131·73 | 22 | 223·34 | 280·22 |
| IL-13 | pg/ml |  | Saliva | 14 | 0·08 | 0·07 | 22 | 0·06 | 0·08 |
| IL-4 | pg/ml |  | Saliva | 14 | 0·15 | 0·14 | 22 | 0·15 | 0·19 |
| MCP-1 | pg/ml |  | Saliva | 14 | 64·48 | 64·59 | 22 | 73·39 | 70·25 |
| IL-8 | pg/ml |  | Saliva | 14 | 969·05 | 874·11 | 21 | 669·93 | 815·92 |
| MIP-1α | pg/ml |  | Saliva | 14 | 0·31 | 0·57 | 22 | 0·31 | 0·48 |
| IL-10 | pg/ml |  | Saliva | 14 | 0·23 | 0·31 | 22 | 0·48 | 1·21 |
| G-CSF | pg/ml |  | Saliva | 14 | 16·29 | 13·24 | 22 | 29·38 | 64·43 |
| IL-15 | pg/ml |  | Saliva | 14 | 21·77 | 29·23 | 22 | 13·24 | 18·96 |
| IL-7 | pg/ml |  | Saliva | 14 | 0·90 | 1·64 | 22 | 1·92 | 2·98 |
| IL-12p70 | pg/ml |  | Saliva | 14 | 0·41 | 0·59 | 21 | 0·24 | 0·29 |
| IL-17 | pg/ml |  | Saliva | 14 | 1·74 | 1·26 | 21 | 1·79 | 2·26 |

Values are reported as means with standard deviations between parentheses.

Abbreviations: FMT: faecal microbiota transplantation; NfL: neurofilament light chain, NfH: neurofilament heavy chain; Treg: T regulatory cells; Th1: T helper type 1; Th17: T helper type 17; CRP: C-reactive protein; IL: interleukin; MCP-1: monocyte-chemoattractant protein 1.

* Data are expressed as fold change relative to FOXP3/b2M expression in a reference sample

§: Below the lower limit of quantification (LLOQ) of the assay.

#### Supplementary Table 3. Patients showing a positive response (increase in Treg of at least 20%), comparing baseline and month 6 (second planned procedure, FMT/SP) between FMT and SP arms.

|  | Positive  response | | not positive response | | Effect of FMT on circulating Treg | | | | | |
| --- | --- | --- | --- | --- | --- | --- | --- | --- | --- | --- |
|  | **n** | **%** | **n** | **%** | **RR** | **CI** | **P value** | **OR** | **CI** | **P value** |
| SP arm | 2 | 20·00 | 8 | 80·00 | - | - | - | - | - | - |
| FMT arm | 8 | 44·44 | 10 | 55·56 | 2·22 | 0·56 - 14·72 | 0·31 | 3·20 | 0·59 - 25·36 | 0·21 |
|  | **Positive**  **response** | | **not positive response** | | **Effect of FMT on intestinal tissue Treg** | | | | | |
|  | **n** | **%** | **n** | **%** | **RR** | **CI** | **P value** | **OR** | **CI** | **P value** |
| SP arm | 1 | 12·50 | 7 | 87·50 | - | - | - | - | - | - |
| FMT arm | 10 | 52·63 | 9 | 47·37 | 4·21 | 0·54 - 32·88 | 0·17 | 7·78 | 0·80 - 76·09 | 0·078 |

Relative risks and odds ratios of presenting a positive response, that is, an increase of at least 20% of circulating or intestinal Treg at month 6 compared to baseline, in both arms, are presented in the table, according to intention-to-treat analysis. Univariable robust Poisson regression models, with log link and sandwich variance estimator, were used to estimate relative risks, whereas univariable logistic regression models were used to estimate odds ratios. Wald tests were used to test the null hypothesis that regression coefficients were equal to 0 and obtained p-values reported. Abbreviations: CI: confidence interval; FMT: faecal microbiota transplantation; SP: sham procedure; OR: odds ratio; RR: relative risk

#### Supplementary Table 4. Changes from baseline to month 6 and 12 in Treg cells across treatment arms. Intention to treat analysis.

| **Biological outcome** | **Time point** | **Arm** |  | | | **absolute change from baseline** | | | |
| --- | --- | --- | --- | --- | --- | --- | --- | --- | --- |
|  |  |  | **N** | **Mean** | **SE** | **MD** | **95% CI** | | **P value** |
| **Circulating Treg**  (%CD4+CD25+CD127-FoxP3+) | Month 6 | SP | 10 | -1·39 | 0·90 | - | - | - | - |
|  |  | FMT | 18 | 0·96 | 0·66 | 2·36 | 0·19 | 4·55 | 0·042 |
|  | Month 12 | SP | 1 | -^§^ | -^§^ | - | - | - | - |
|  |  | FMT | 10 | -1·07 | 0·96 | -^§^ | -^§^ | -^§^ | -^§^ |
| **Tissue Treg**  **(**FOXP3/b2M expression) | Month 6 | SP | 8 | -0·15 | 1·30 | - | - | - | - |
|  |  | FMT | 19 | 1·07 | 0·84 | 1·22 | -1·77 | 4·27 | 0·44 |
|  | Month 12 | SP | 1 | -^§^ | -^§^ | - | - | - | - |
|  |  | FMT | 9 | -0·16 | 0·49 | -^§^ | -^§^ | -^§^ | -^§^ |

Mean absolute changes from baseline to month 6 and 12 are showed for each treatment group and comparisons were performed using linear mixed- effect regression models that include indicator variables for treatment arms, time and the interaction arm x time as independent variables. T tests were used to test the null hypothesis that regression coefficients were equal to 0 and obtained p-values reported. Satterthwaite approximation was used to estimate degrees of freedom.

Abbreviations: N: number of samples for analysis; MD = Mean Difference; se = Standard Error; CI = Confidence Interval; SP: Sham Procedure; FMT: Faecal Microbiota Transplantation.

^§^ these values couldn’t be calculated as only one observation per SP arm was available.

#### Supplementary Table 5. Average monthly variations in circulating and tissue Treg in FMT and SP patients, and the estimate of the mean difference at baseline and for every month between month 6 and baseline comparing the two arms.

| **Circulating Treg** |  | **Estimate** | **SE** | **95% CI** | | **P value** |
| --- | --- | --- | --- | --- | --- | --- |
| Treg (%) | Mean difference between FMT and SP | -0·03 | 0·84 | -1·65 | 1·60 | 0·97 |
|  | Monthly variation SP | -0·23 | 0·15 | -0·52 | 0·06 | 0·13 |
|  | Monthly variation FMT | 0·16 | 0·11 | -0·05 | 0·38 | 0·15 |
|  | Mean difference in monthly variation | 0·39 | 0·19 | 0·03 | 0·76 | 0·042 |

**Intestinal Treg	Estimate	SE	95% CI	P value**

| FOXP3/b2M expression | Mean difference between FMT and SP | -0·41 | 1·38 | -3·07 | 2·25 | 0·77 |
| --- | --- | --- | --- | --- | --- | --- |
|  | Monthly variation SP | -0·02 | 0·21 | -0·44 | 0·39 | 0·91 |
|  | Monthly variation FMT | 0·18 | 0·14 | -0·09 | 0·45 | 0·20 |
|  | Mean difference in monthly variation | 0·20 | 0·25 | -0·29 | 0·70 | 0·43 |

Monthly variations and differences between monthly variations in Treg, considered as percentage over total CD4 T cells, or in absolute concentrations, and intestinal Treg, were estimated using linear mixed-effects models. These models were specified with a random intercept term and included as covariates arm, time and their interaction. T tests were used to test the null hypothesis that regression coefficients were equal to 0 and obtained p-values reported. Satterthwaite approximation was used to estimate degrees of freedom.

Abbreviations: CI: confidence interval; SE: standard error. Statistically significant mean estimates were reported in bold characters.

#### Supplementary Table 6. Primary and secondary efficacy outcomes at month 6 in the intention-to-treat population. The table summarizes primary and secondary efficacy outcomes comparing FMT with SP at the primary study timepoint, month 6. Binary outcomes are reported as number and percentage of participants with the event, with odds ratios (ORs) or hazard ratios (HRs) and 95% confidence intervals (CIs), as appropriate. Continuous outcomes are reported as mean changes from baseline, values at month 6, or average monthly variations estimated using mixed-effects models, with corresponding mean differences (MDs) or ratios and 95% CIs. Positive MD values indicate results favouring FMT when they correspond to slower clinical decline or greater biological response compared with SP; negative MD values indicate lower values in the FMT group. Survival outcomes were analysed as time-to-event variables from randomization to death, tracheostomy, PEG placement, or initiation of non-invasive ventilation (NIV), as appropriate. T tests were used to test the null hypothesis that regression coefficients were equal to 0 and obtained p-values reported. In the primary analysis, Satterthwaite approximation was used to estimate degrees of freedom. Sensitivity analyses using the Kenward–Roger approximation for degrees of freedom are shown for selected mixed-effects models. Analyses were performed in the intention-to-treat population, including all randomised participants with available longitudinal assessments.

|  |  |  |  |  | **Kenward-Roger approximation** | | |
| --- | --- | --- | --- | --- | --- | --- | --- |
| **Outcome** | **SP arm** | **FMT arm** | **Effect estimate (95% CI)** | **p value** | **Effect estimate (95% CI)** | **p value** | **DF estimation** |
| **Primary endpoint** |  |  |  |  |  |  |  |
| Participants with ≥20% increase in circulating Tregs, n/N (%) | 2/10 (20·0%) | 8/18 (44·4%) | OR 3·20 (0·59 to 25·36) | 0·21 |  |  |  |
| Participants with any increase in circulating Tregs, n/N (%) | 3/10 (30·0%) | 12/18 (66·7%) | OR 4·67 (0·94 to 28·47) | 0·071 |  |  |  |
| Change in circulating Tregs from baseline, mean (SE) | –1·39 (0·90) | 0·96 (0·66) | MD 2·36 (0·19 to 4·55) | 0·042 | MD 2·36 (0·12 to 4·59) | 0·039 | KR |
| Change in intestinal Tregs from baseline, mean (SE) | –0·15 (1·30) | 1·07 (0·84) | MD 1·22 (–1·77 to 4·27) | 0·44 | MD 1·22 (–1·88 to 4·31) | 0·43 | KR |
| **Secondary clinical outcomes** |  |  |  |  |  |  |  |
| ALSFRS-R monthly decline (points/month), mean (SE) | –1·07 (0.08) | –0·76 (0.06) | MD 0·31 (0·11 to 0·51) | 0·002 | MD 0·31 (0·11 to 0·51) | 0·002 | KR |
| FVC at 6 months (%), mean (SD) | 77·30 (19·77) | 86·87 (19·61) | MD 9·57 (–5·62 to 24·75) | 0·21 |  |  |  |
| MRC at 6 months (proportion of maximum possible score), mean (SD) | 0·89 (0·08) | 0·75 (0·19) | MD –0·13 (–0·26 to 0·00) | 0·042 |  |  |  |
| BMI at 6 months (kg/m²), mean (SD) | 25·95 (2·61) | 24·44 (3·93) | MD –1·20 (–3·55 to 1·15) | 0·31 |  |  |  |
| ALSAQ-40 total score at 6 months, mean (SD) | 30·56 (22·61) | 44, ·13 (19·71) | MD 13·57 (–2·34 to 29·48) | 0·092 |  |  |  |
| PEG placement, n/N (%) | 3/14 (21·4%) | 2/28 (7·1%) | HR 0·26 (0·04 to 1·59) | 0·15 |  |  |  |
| NIV initiation, n/N (%) | 3/14 (21·4%) | 9/28 (32·1%) | HR 1·30 (0·35 to 4·80) | 0·69 |  |  |  |
| Tracheostomy-free survival events, n/N (%) |  |  | HR 0·15 (0·02 to 1·48) | 0·10 |  |  |  |
| **Secondary biological outcomes** |  |  |  |  |  |  |  |
| Treg/Th1 ratio change from baseline to month 6, mean ratio (SE) | 1·19 (1·20) | 3·07 (1·92) | Ratio 2·58 (0·46 to 13·78) | 0·28 | Ratio 2·58 (0·46 to 13·78) | 0·28 | KR |
| Treg/Th17 ratio change from baseline to month 6, mean ratio (SE) | 0·85 (0·51) | 1·23 (0·50) | Ratio 1·45 (0·43 to 4·79) | 0·55 | Ratio 1·45 (0·43 to 4·79) | 0·55 | KR |
| Serum NfH at 6 months, pg/ml, mean (SD) | 3755.39 (2637.86) | 2431.50 (2127.41) | MD –1323·89 (–3109·51 to 461·73) | 0·14 |  |  |  |
| Serum NfL at 6 months, pg/ml, mean (SD) | 145.35 (72.18) | 134.86 (93.21) | MD –10·49 (–78·59 to 57·61) | 0·75 |  |  |  |
| Creatinine at 6 months, mg/dl, mean (SD) | 0.69 (0.17) | 0.61 (0.18) | MD –0·08 (–0·21 to 0·05) | 0·23 |  |  |  |
| Albumin at 6 months, g/dl, mean (SD) | 4.36 (0.14) | 4.33 (0.29) | MD -0·03 (–0·22 to 0·17) | 0·76 |  |  |  |
| Vitamin D at 6 months, ng/ml, mean (SD) | 26.06 (8.08) | 27.16 (13.51) | MD 1·11 (–10·03 to 12·24) | 0·84p |  |  |  |

#### Supplementary Table 7. Average ALSFRS-r (total scores and subscores) per treatment arm during FETR-ALS trial in intention-to-treat population.

**]**

|  | **M1** | | | | **M3** | | | | **M6** | | | | **M7** | | | | **M9** | | | | **M12** | | | |
| --- | --- | --- | --- | --- | --- | --- | --- | --- | --- | --- | --- | --- | --- | --- | --- | --- | --- | --- | --- | --- | --- | --- | --- | --- |
|  | **SP (n=14)** | | **FMT (n=27)** | | **SP (n=14)** | | **FMT (n=26)** | | **SP (n=10)** | | **FMT (n=23)** | | **SP (n=10)** | | **FMT (n=20)** | | **SP (n=9)** | | **FMT (n=20)** | | **SP (n=8)** | | **FMT (n=17)** | |
| **Variable** | Mean | SD | Mean | SD | Mean | SD | Mean | SD | Mean | SD | Mean | SD | Mean | SD | Mean | SD | Mean | SD | Mean | SD | Mean | SD | Mean | SD |
| Progression Rate | 1·24 | 1·54 | 0·76 | 0·54 | 1·60 | 2·08 | 0·96 | 0·62 | 0·91 | 0·45 | 1·10 | 0·65 | 1·08 | 0·48 | 1·03 | 0·64 | 1·53 | 0·99 | 1·21 | 0·70 | 1·54 | 0·60 | 1·24 | 0·62 |
| ALSFRS-R Score | 37·64 | 7·57 | 37·96 | 6·62 | 34·86 | 9·89 | 35·08 | 8·18 | 37·7 | 5·38 | 34·00 | 9·01 | 35·80 | 5·41 | 35·4 | 7·86 | 31·78 | 6·65 | 33·45 | 8·58 | 29·50 | 6·82 | 31·59 | 9·55 |
| ALSFRS-R Items 1-3 | 10·43 | 1·95 | 10·70 | 2·03 | 9·43 | 2·68 | 10·58 | 1·68 | 9·10 | 3·45 | 10·13 | 2·49 | 8·70 | 3·89 | 10·25 | 2·29 | 8·22 | 4·15 | 9·75 | 2·81 | 9·13 | 2·85 | 10·00 | 2·62 |
| ALSFRS-R Items 4-6 | 7·86 | 2·98 | 7·63 | 3·53 | 7·14 | 3·23 | 6·31 | 4·15 | 8·50 | 2·42 | 6·09 | 4·39 | 8·00 | 2·21 | 6·65 | 4·12 | 7·00 | 3·16 | 5·95 | 4·12 | 5·25 | 2·92 | 5·18 | 4·11 |
| ALSFRS-R Items 7-9 | 7·86 | 3·84 | 7·81 | 3·11 | 7·29 | 4·10 | 7·00 | 3·24 | 8·40 | 3·34 | 6·83 | 3·42 | 7·90 | 3·75 | 7·10 | 3·35 | 6·11 | 3·10 | 6·90 | 3·54 | 4·88 | 2·90 | 5·88 | 3·97 |
| ALSFRS-R Items 10-12 | 11·5 | 1·61 | 11·81 | 0·79 | 11·00 | 2·91 | 11·19 | 1·96 | 11·70 | 0·95 | 10·96 | 1·82 | 11·20 | 1·75 | 11·4 | 1·14 | 10·44 | 2·55 | 10·85 | 2·35 | 10·25 | 2·55 | 10·53 | 2·70 |

Mean values and their standard deviations (SD) are shown in the sham and FMT-treated arm. Abbreviations: FMT: faecal micropbiota transplantation; SD: standard deviation.

#### Supplementary Table 8. Mean differences between FMT and SP arms in ALSFRS-r total scores and subscores at month 6 and month 12 (assessed as changes with respect to baseline scores). Intention-to-treat analysis.

|  | **At 6 months** | | | | | **At 12 months** | | | | |
| --- | --- | --- | --- | --- | --- | --- | --- | --- | --- | --- |
| **Outcome** | **MD** | **Std· Error** | **95% Conf· Interval** | | **P Value** | **MD** | **Std· Error** | **95% Conf· Interval** | | **P Value** |
| ALSFRS-R Total Score | -3·70 | 3·08 | -9·98 | 2·58 | 0·24 | 2·09 | 3·78 | -5·73 | 9·90 | 0·59 |
| ALSFRS-R Items 1-3 | 1·03 | 1·06 | -1·13 | 3·20 | 0·34 | 0·88 | 1·15 | -1·51 | 3·26 | 0·46 |
| ALSFRS-R Items 4-6 | -2·41 | 1·48 | -5·44 | 0·62 | 0·11 | -0·07 | 1·62 | -3·43 | 3·29 | 0·96 |
| ALSFRS-R Items 7-9 | -1·57 | 1·29 | -4·20 | 1·05 | 0·23 | 1·01 | 1·58 | -2·25 | 4·27 | 0·53 |
| ALSFRS-R Items 10-12 | -0·74 | 0·61 | -1·99 | 0·51 | 0·23 | 0·28 | 1·14 | -2·07 | 2·63 | 0·81 |

For these analysis, the mean differences between the average of the difference of each score in the FMT-treated and sham procedure arm between month 6 and baseline and month 12 and baseline were used, respectively, using univariable linear regression models. Wald tests were used to test the null hypothesis that regression coefficients were equal to 0 and obtained p-values reported. MD: mean difference between FMT versus SP arm.

Abbreviations: ALSFRS-r: Amyotrophic Lateral Sclerosis Functional Rating Scale, revised; SE: standard error; CI: confidence interval. FMT: faecal microbiota transplantation; SP: Sham Procedure.

#### Supplementary Table 9. Average monthly variations in ALSFRS-r and its subscores in FMT and SP arms, and the mean differences over time between the two arms, in intention-to-treat population.

| **Outcome** |  | **Estimate** | **Std· Error** | **95% Conf· Interval** | | **P Value** |
| --- | --- | --- | --- | --- | --- | --- |
| ALSFRS-R Total Score | Monthly variation SPs | -1·07 | 0·08 | -1·24 | -0·91 | <0·0001 |
|  | Monthly variation FMT | -0·76 | 0·06 | -0·87 | -0·65 | <0·0001 |
|  | **Mean difference in monthly variation** | 0·31 | 0·10 | 0·11 | 0·51 | 0·0020 |
| ALSFRS-R Items 1-3 | Monthly variation SPs | -0·2 | 0·03 | -0·26 | -0·15 | <0·0001 |
|  | Monthly variation FMT | -0·14 | 0·02 | -0·18 | -0·10 | <0·0001 |
|  | Mean difference in monthly variation | 0·06 | 0·03 | -0·01 | 0·13 | 0·073 |
| ALSFRS-R Items 4-6 | Monthly variation SPs | -0·37 | 0·04 | -0·44 | -0·30 | <0·0001 |
|  | Monthly variation FMT | -0·26 | 0·03 | -0·31 | -0·21 | <0·0001 |
|  | **Mean difference in monthly variation** | 0·11 | 0·04 | 0·02 | 0·20 | 0·012 |
| ALSFRS-R Items 7-9 | Monthly variation SPs | -0·34 | 0·03 | -0·41 | -0·28 | <0·0001 |
|  | Monthly variation FMT | -0·24 | 0·02 | -0·28 | -0·19 | <0·0001 |
|  | **Mean difference in monthly variation** | 0·11 | 0·04 | 0·03 | 0·19 | 0·0091 |
| ALSFRS-R Items 10-12 | Monthly variation SPs | -0·15 | 0·04 | -0·22 | -0·08 | <0·0001 |
|  | Monthly variation FMT | -0·12 | 0·02 | -0·17 | -0·07 | <0·0001 |
|  | Mean difference in monthly variation | 0·03 | 0·04 | -0·05 | 0·11 | 0·50 |

Monthly variations of ALSFRS-S total score and subscores and differences between monthly variations in the two treatment arms were estimated by linear mixed-effect models with random intercept. These models included as covariates arm, time and the interaction arm x time. T tests were used to test the null hypothesis that regression coefficients were equal to 0 and obtained p-values reported. Satterthwaite approximation was used to estimate degrees of freedom.

Abbreviations: ALSFRS-r: ALS functional rating scale, revised; FMT: faecal microbiota transplantation; SP: Sham Procedure.

#### Supplementary Table 10. Average decline in ALSFRS-r total score and subscores in segmented repeated-measures analysis during FETR-ALS trial, in SP and FMT arms, and their mean differences, considering immediately following first FMT (month 0-1), delayed after first FMT (month 1-6), immediately following the second FMT (month 6-7), delayed after the second FMT (month 7-12). Intention-to-treat analysis.

| **Outcome** |  | **Estimate** | **Std· Error** | **95% Conf· Interval** | | **P Value** |
| --- | --- | --- | --- | --- | --- | --- |
| ALSFRS-R Total Score | Monthly variation SP (months 0-1) | -2·73 | 0·62 | -3·92 | -1·53 | <0·0001 |
|  | Monthly variation SP (months 1-6) | -1·69 | 0·41 | -2·50 | -0·90 | <0·0001 |
|  | Monthly variation SP (months 6-7) | -1·69 | 0·40 | -2·49 | -0·91 | <0·0001 |
|  | Monthly variation SPs (months 7-12) | -1·87 | 0·40 | -2·67 | -1·09 | <0·0001 |
|  | Monthly variation FMT (months 0-1) | -0·95 | 0·44 | -1·8 | -0·09 | 0·033 |
|  | Monthly variation FMT (months 1-6) | -1·28 | 0·29 | -1·86 | -0·71 | <0·0001 |
|  | Monthly variation FMT (months 6-7) | -1·25 | 0·29 | -1·83 | -0·69 | <0·0001 |
|  | Monthly variation FMT (months 7-12) | -1·25 | 0·29 | -1·82 | -0·69 | <0·0001 |
|  | Mean difference in monthly variation (months 0-1) | 1·78 | 0·76 | 0·31 | 3·25 | 0·020 |
|  | Mean difference in monthly variation (months 1-6) | 0·41 | 0·50 | -0·56 | 1·40 | 0·41 |
|  | Mean difference in monthly variation (months 6-7) | 0·44 | 0·50 | -0·53 | 1·42 | 0·38 |
|  | Mean difference in monthly variation (months 7-12) | 0·62 | 0·49 | -0·34 | 1·59 | 0·22 |
| ALSFRS-R Items 1-3 | Monthly variation SP (months 0-1) | -0·22 | 0·20 | -0·61 | 0·17 | 0·27 |
|  | Monthly variation SP (months 1-6) | -0·35 | 0·10 | -0·54 | -0·15 | 0·0011 |
|  | Monthly variation SP (months 6-7) | -0·35 | 0·10 | -0·54 | -0·16 | 0·0011 |
|  | Monthly variation SPs (months 7-12) | -0·35 | 0·10 | -0·55 | -0·17 | 0·0011 |
|  | Monthly variation FMT (months 0-1) | -0·17 | 0·14 | -0·45 | 0·11 | 0·23 |
|  | Monthly variation FMT (months 1-6) | -0·19 | 0·07 | -0·33 | -0·06 | 0·010 |
|  | Monthly variation FMT (months 6-7) | -0·21 | 0·07 | -0·35 | -0·08 | 0·0040 |
|  | Monthly variation FMT (months 7-12) | -0·22 | 0·07 | -0·36 | -0·09 | 0·0032 |
|  | Mean difference in monthly variation (months 0-1) | 0·05 | 0·25 | -0·43 | 0·53 | 0·85 |
|  | Mean difference in monthly variation (months 1-6) | 0·15 | 0·12 | -0·08 | 0·39 | 0·21 |
|  | Mean difference in monthly variation (months 6-7) | 0·13 | 0·12 | -0·10 | 0·37 | 0·28 |
|  | Mean difference in monthly variation (months 7-12) | 0·14 | 0·12 | -0·10 | 0·37 | 0·26 |
| ALSFRS-R Items 4-6 | Monthly variation SP (months 0-1) | -1·29 | 0·33 | -1·92 | -0·65 | <0·0001 |
|  | Monthly variation SP (months 1-6) | -0·52 | 0·12 | -0·76 | -0·29 | <0·0001 |
|  | Monthly variation SP (months 6-7) | -0·45 | 0·11 | -0·69 | -0·23 | <0·0001 |
|  | Monthly variation SPs (months 7-12) | -0·51 | 0·11 | -0·74 | -0·30 | <0·0001 |
|  | Monthly variation FMT (months 0-1) | -0·27 | 0·23 | -0·73 | 0·18 | 0·25 |
|  | Monthly variation FMT (months 1-6) | -0·40 | 0·08 | -0·57 | -0·24 | <0·0001 |
|  | Monthly variation FMT (months 6-7) | -0·37 | 0·08 | -0·53 | -0·22 | <0·0001 |
|  | Monthly variation FMT (months 7-12) | -0·36 | 0·08 | -0·52 | -0·21 | <0·0001 |
|  | Mean difference in monthly variation (months 0-1) | 1·01 | 0·40 | 0·23 | 1·79 | **0·012** |
|  | Mean difference in monthly variation (months 1-6) | 0·12 | 0·15 | -0·17 | 0·41 | 0·43 |
|  | Mean difference in monthly variation (months 6-7) | 0·08 | 0·14 | -0·19 | 0·36 | 0·57 |
|  | Mean difference in monthly variation (months 7-12) | 0·15 | 0·13 | -0·11 | 0·42 | 0·27 |
| ALSFRS-R Items 7-9 | Monthly variation SP (months 0-1) | -0·64 | 0·27 | -1·15 | -0·12 | 0·016 |
|  | Monthly variation SP (months 1-6) | -0·37 | 0·10 | -0·56 | -0·17 | 0·0012 |
|  | Monthly variation SP (months 6-7) | -0·36 | 0·10 | -0·56 | -0·18 | 0·0011 |
|  | Monthly variation SPs (months 7-12) | -0·43 | 0·09 | -0·61 | -0·25 | <0·0001 |
|  | Monthly variation FMT (months 0-1) | -0·33 | 0·19 | -0·7 | 0·04 | 0·086 |
|  | Monthly variation FMT (months 1-6) | -0·34 | 0·07 | -0·47 | -0·20 | <0·0001 |
|  | Monthly variation FMT (months 6-7) | -0·32 | 0·07 | -0·45 | -0·19 | <0·0001 |
|  | Monthly variation FMT (months 7-12) | -0·30 | 0·06 | -0·43 | -0·18 | <0·0001 |
|  | Mean difference in monthly variation (months 0-1) | 0·31 | 0·33 | -0·32 | 0·95 | 0·34 |
|  | Mean difference in monthly variation (months 1-6) | 0·03 | 0·12 | -0·20 | 0·27 | 0·80 |
|  | Mean difference in monthly variation (months 6-7) | 0·05 | 0·12 | -0·18 | 0·28 | 0·68 |
|  | Mean difference in monthly variation (months 7-12) | 0·13 | 0·11 | -0·09 | 0·35 | 0·27 |
| ALSFRS-R Items 10-12 | Monthly variation SP (months 0-1) | -0·44 | 0·28 | -0·99 | 0·09 | 0·11 |
|  | Monthly variation SP (months 1-6) | -0·33 | 0·20 | -0·72 | 0·06 | 0·11 |
|  | Monthly variation SP (months 6-7) | -0·37 | 0·20 | -0·76 | 0·02 | 0·074 |
|  | Monthly variation SPs (months 7-12) | -0·41 | 0·20 | -0·80 | -0·03 | 0·045 |
|  | Monthly variation FMT (months 0-1) | -0·15 | 0·20 | -0·53 | 0·24 | 0·47 |
|  | Monthly variation FMT (months 1-6) | -0·34 | 0·14 | -0·62 | -0·06 | 0·025 |
|  | Monthly variation FMT (months 6-7) | -0·32 | 0·14 | -0·61 | -0·05 | 0·030 |
|  | Monthly variation FMT (months 7-12) | -0·33 | 0·14 | -0·62 | -0·06 | 0·026 |
|  | Mean difference in monthly variation (months 0-1) | 0·30 | 0·34 | -0·37 | 0·96 | 0·38 |
|  | Mean difference in monthly variation (months 1-6) | -0·01 | 0·25 | -0·49 | 0·47 | 0·98 |
|  | Mean difference in monthly variation (months 6-7) | 0·04 | 0·24 | -0·44 | 0·52 | 0·86 |
|  | Mean difference in monthly variation (months 7-12) | 0·08 | 0·24 | -0·40 | 0·56 | 0·74 |

For this analysis, four segments of time were considered: immediately following the first FMT (month 0-1), delayed effect after first FMT (month 1-6), immediately following the second FMT (month 6-7), delayed effects after the second FMT (month 7-12). The intention-to-treat cohort population was

included for this analysis. Monthly variations and differences in monthly variations between arms were estimated using random intercept segmented linear mixed-effect models, which included as covariates arm, time segments, and the interaction arm x time segment. T tests were used to test the null hypothesis that regression coefficients were equal to 0 and obtained p-values reported. Satterthwaite approximation was used to estimate degrees of freedom.

Abbreviations: ALSFRS-r: ALS functional rating scale, revised; FMT: faecal microbiota transplantation; SP: Sham Procedure.

#### Supplementary Table 11. Average monthly decline in ALSFRS-r total score in segmented repeated-measures analysis during FETR-ALS trial, in SP and FMT arms, and their mean differences, considering before baseline, immediately following first FMT (month 0-1), delayed after first FMT (month 1-6), immediately following the second FMT (month 6-7), delayed after the second FMT (month 7-12). Intention-to-treat analysis.

| **ALSFRS-R Total Score** | **Estimate** | **Std· Error** | **95% Conf· Interval** | | **P Value** |
| --- | --- | --- | --- | --- | --- |
| Monthly variation SP (onset to randomization) | -0·91 | 0·21 | -1·33 | -0·49 | <0·0001 |
| Monthly variation SP (months 0-1) | -2·77 | 0·65 | -4·03 | -1·52 | <0·0001 |
| Monthly variation SP (months 1-6) | -1·48 | 0·24 | -1·94 | -1·02 | <0·0001 |
| Monthly variation SP (months 6-7) | -1·39 | 0·23 | -1·83 | -0·95 | <0·0001 |
| Monthly variation SPs (months 7-12) | -1·55 | 0·22 | -1·98 | -1·13 | <0·0001 |
| Monthly variation FMT (eonset to randomization) | -0·66 | 0·15 | -0·96 | -0·37 | <0·0001 |
| Monthly variation FMT (months 0-1) | -0·82 | 0·46 | -1·72 | 0·08 | 0·079 |
| Monthly variation FMT (months 1-6) | -1·16 | 0·17 | -1·48 | -0·84 | <0·0001 |
| Monthly variation FMT (months 6-7) | -1·05 | 0·16 | -1·36 | -0·74 | <0·0001 |
| Monthly variation FMT (months 7-12) | -0·98 | 0·15 | -1·28 | -0·69 | <0·0001 |
| Mean difference in monthly variation (onset to randomization) | 0·24 | 0·26 | -0·27 | 0·76 | 0·36 |
| Mean difference in monthly variation (months 0-1) | 1·96 | 0·80 | 0·42 | 3·50 | 0·014 |
| Mean difference in monthly variation (months 1-6) | 0·32 | 0·29 | -0·24 | 0·89 | 0·26 |
| Mean difference in monthly variation (months 6-7) | 0·34 | 0·28 | -0·19 | 0·88 | 0·22 |
| Mean difference in monthly variation (months 7-12) | 0·57 | 0·26 | 0·06 | 1·09 | 0·037 |

For this analysis, five segments of time were considered: before randomization (from disease onset to month 0), immediately following the first FMT (month 0-1), delayed effect after first FMT (month 1-6), immediately following the second FMT (month 6-7), delayed effects after the second FMT (month 7-12). For each patient, ALSFRS-r total score was estimated to be 48 at disease onset. Monthly variations and differences in monthly variations between arms were estimated using random intercept segmented linear mixed-effect models, which included as covariates arm, time segments, and the interaction arm x time segment. T tests were used to test the null hypothesis that regression coefficients were equal to 0 and obtained p-values reported. Satterthwaite approximation was used to estimate degrees of freedom.

Abbreviations: ALSFRS-r: ALS functional rating scale, revised; FMT: faecal microbiota transplantation; SP: Sham Procedure. ITT: Intention-to-treat.

#### Supplementary Table 12. Mean differences between FMT and SP arms in clinical outcome measures from baseline to month 6 and month 12. Intention-to-treat analysis.

|  | **At 6 months** | | | | | **At 12 months** | | | | |
| --- | --- | --- | --- | --- | --- | --- | --- | --- | --- | --- |
| **Outcome** | **MD** | **Std· Error** | **95% Conf· Interval** | | **P**  **value** | **MD** | **Std· Error** | **95% Conf· Interval** | | **P value** |
| FVC (% of predicted normal value) | 9·57 | 7·45 | -5·62 | 24·75 | 0·21 | 14·23 | 9·68 | -5·97 | 34·43 | 0·16 |
| MRC (proportion of the maximum possible score) | -0·13 | 0·06 | -0·26 | 0·00 | 0·042 | -0·05 | 0·08 | -0·21 | 0·11 | 0·50 |
| BMI (kg/m2) | -1·20 | 1·16 | -3·55 | 1·15 | 0·31 | -0·43 | 1·75 | -4·04 | 3·19 | 0·81 |
| ALSAQ-40 Total Score | 13·57 | 7·80 | -2·34 | 29·48 | 0·092 | -6·57 | 9·30 | -25·81 | 12·67 | 0·49 |
| ALSAQ-40 Physical mobility | 9·40 | 12·03 | -15·33 | 34·14 | 0·44 | -11·63 | 15·53 | -43·93 | 20·68 | 0·46 |
| ALSAQ-40 Independence | 20·95 | 12·35 | -4·34 | 46·24 | 0·10 | -6·56 | 13·04 | -33·6 | 20·47 | 0·62 |
| ALSAQ-40 Eat and drink | -1·39 | 14·09 | -31·26 | 28·48 | 0·92 | -6·67 | 12·93 | -34·83 | 21·5 | 0·61 |
| ALSAQ-40 Communication | 2·64 | 15·14 | -29·17 | 34·44 | 0·86 | 3·62 | 15·22 | -28·82 | 36·06 | 0·81 |
| ALSAQ-40 Emotion | 11·01 | 8·93 | -7·25 | 29·26 | 0·23 | 2·10 | 9·94 | -18·48 | 22·67 | 0·83 |

For these analysis, the mean differences between each parameter in the FMT-treated and sham procedure (SP) arm between month 6 and baseline and month 12 and baseline were estimated by univariable linear regression models. Wald tests were used to test the null hypothesis that regression coefficients were equal to 0 and obtained p-values reported.

MD: mean difference between FMT versus SP arm.

Abbreviations: FVC: forced vital capacity; MRC: medical research council score; BMI: body mass index; ALSQ-40: ALS- quality of life questionnaire (40 items). FMT: faecal microbiota transplantation; SP: Sham Procedure.

#### Supplementary Table 13. Average monthly variations in clinical outcome measures in FMT and SP arms, and the mean differences over time between the two arms, in intention-to-treat population.

| **Outcome** |  | **Estimate** | **Std· Error** | **95% Conf· Interval** | | **p·value** |
| --- | --- | --- | --- | --- | --- | --- |
| FVC (% of predicted normal value) | Monthly variation SP | -2·09 | 0·31 | -2·7 | -1·48 | < 0·0001 |
|  | Monthly variation FMT | -1·74 | 0·21 | -2·14 | -1·33 | < 0·0001 |
|  | Mean difference in monthly variation | 0·35 | 0·37 | -0·38 | 1·08 | 0·34 |
| MRC (proportion of the maximum possible score) | Monthly variation SP | -0·01 | 0·00 | -0·02 | -0·01 | < 0·0001 |
|  | Monthly variation FMT | -0·01 | 0·00 | -0·01 | -0·01 | < 0·0001 |
|  | Mean difference in monthly variation | 0·00 | 0·00 | 0·00 | 0·00 | 0·71 |
| BMI (kg/m2) | Monthly variation SP | -0·12 | 0·02 | -0·16 | -0·08 | < 0·0001 |
|  | Monthly variation FMT | -0·02 | 0·01 | -0·05 | 0·00 | 0·10 |
|  | Mean difference in monthly variation | 0·10 | 0·03 | 0·04 | 0·15 | < 0·0001 |
| ALSAQ-40 Total Score | Monthly variation SP | 2·36 | 0·40 | 1·56 | 3·14 | < 0·0001 |
|  | Monthly variation FMT | 1·35 | 0·27 | 0·81 | 1·88 | < 0·0001 |
|  | Mean difference in monthly variation | -1·01 | 0·48 | -1·95 | -0·05 | 0·042 |
| ALSAQ-40 Subscales: Physical mobility | Monthly variation SP | 2·68 | 0·69 | 1·28 | 4·02 | < 0·0001 |
|  | Monthly variation FMT | 2·00 | 0·45 | 1·12 | 2·87 | < 0·0001 |
|  | Mean difference in monthly variation | -0·68 | 0·82 | -2·27 | 0·96 | 0·411 |
| ALSAQ-40 Subscales: Independence | Monthly variation SP | 3·27 | 0·62 | 2·02 | 4·47 | < 0·0001 |
|  | Monthly variation FMT | 1·50 | 0·40 | 0·71 | 2·28 | < 0·0001 |
|  | Mean difference in monthly variation | -1·77 | 0·74 | -3·20 | -0·30 | 0·020 |
| ALSAQ-40 Subscales: Eat and drink | Monthly variation SP | 2·45 | 0·82 | 0·83 | 4·02 | 0·0050 |
|  | Monthly variation FMT | 0·88 | 0·66 | -0·43 | 2·15 | 0·19 |
|  | Mean difference in monthly variation | -1·57 | 1·05 | -3·6 | 0·48 | 0·15 |
| ALSAQ-40 Subscales: Communication | Monthly variation SP | 2·29 | 0·91 | 0·42 | 4·04 | 0·017 |
|  | Monthly variation FMT | 1·60 | 0·74 | 0·13 | 3·04 | 0·038 |
|  | Mean difference in monthly variation | -0·68 | 1·18 | -2·95 | 1·66 | 0·57 |
| ALSAQ-40 Subscales: Emotion | Monthly variation SP | 1·70 | 0·5 | 0·71 | 2·67 | 0·001 |
|  | Monthly variation FMT | 0·63 | 0·33 | -0·03 | 1·28 | 0·063 |
|  | Mean difference in monthly variation | -1·07 | 0·6 | -2·24 | 0·11 | 0·082 |

Monthly variations and differences between monthly variations in the two treatment arms with respect to the analysed outcomes were estimated by linear mixed-effect models with random intercept. These models included as covariates arm, time and the interaction arm x time. T tests were used to test the null hypothesis that regression coefficients were equal to 0 and obtained p-values reported. Satterthwaite approximation was used to estimate degrees of freedom.

Abbreviations: FVC: forced vital capacity; MRC: medical research council score; BMI: body mass index; ALSQ-40: ALS- quality of life questionnaire (40 items). FMT: faecal microbiota transplantation; SP: Sham Procedure.

**Supplementary Table 14: Treatment-emergent adverse events during the FETR-ALS trial between month 6 and month 12, by treatment arm (FMT vs. SP) and categorized according to MedDRA classification. Intention-to treat population.**

| **Treatment-emergent adverse events, MedDRA preferred term** | **SP arm** | | **FMT arm** | | **Risk difference** | **95% CI** | |
| --- | --- | --- | --- | --- | --- | --- | --- |
|  | **(n = 10)** | | **(n = 23)** | |  |  |  |
|  | **N** | **%** | **N** | **%** |  |  |  |
| **Drop-outs** | 2 | 20·00 | 6 | 26·09 | 0·06 | -0·25 | 0·37 |
| Deaths/Tracheostomy | 1 | 10·00 | 0 | 0·00 | -0·10 | -0·29 | 0·09 |
| Withdrawals due to harms | 1 | 10·00 | 2 | 8·70 | -0·01 | -0·23 | 0·21 |
| **Nr participants at risk of harms at month 12** | 8 | 80·00 | 17 | 73·91 | -0·06 | -0·37 | 0·25 |
| **Nr patients with at least one AE** | 3 | 30·00 | 6 | 26·09 | -0·04 | -0·38 | 0·30 |
| **AEs** | 3 |  | 10 |  |  |  |  |
| **SAEs** | 2 |  | 2 |  |  |  |  |
| **Adverse Events of Special Interest** |  |  |  |  |  |  |  |
| ***Gastrointestinal disorders*** | **0** | 0·00 | **2** | 8·70 | **0·09** | **-0·03** | **0·20** |
| Constipation | 0 | 0·00 | 0 | 0·00 | 0·00 | -0·28 | 0·14 |
| Faecaloma | 0 | 0·00 | 1 | 4·35 | 0·04 | -0·04 | 0·13 |
| Diarrhoea - Nausea | 0 | 0·00 | 0 | 0·00 | 0·00 | -0·28 | 0·14 |
| Hypogastrium distension - Hypogastrium pain | 0 | 0·00 | 0 | 0·00 | 0·00 | -0·28 | 0·14 |
| Chronic gastritis | 0 | 0·00 | 0 | 0·00 | 0·00 | -0·28 | 0·14 |
| Enteritis | 0 | 0·00 | 1 | 4·35 | 0·04 | -0·04 | 0·13 |
| ***Infections and infestations*** | **0** | 0·00 | **2** | 8·70 | **0·09** | **-0·03** | **0·20** |
| Covid 19 | 0 | 0·00 | 0 | 0·00 | 0·00 | -0·28 | 0·14 |
| Peristomal infection | 0 | 0·00 | 1 | 4·35 | 0·04 | -0·04 | 0·13 |
| Gastroenteritis | 0 | 0·00 | 1 | 4·35 | 0·04 | -0·04 | 0·13 |
| **Others Adverse Events** |  |  |  |  |  |  |  |
| ***Metabolism and nutrition disorders*** | **0** | 0·00 | **1** | 4·35 | **0·04** | **-0·04** | **0·13** |
| Hypoglicemia | 0 | 0·00 | 0 | 0·00 | 0·00 | -0·28 | 0·14 |
| Dysphagia | 0 | 0·00 | 1 | 4·35 | 0·04 | -0·04 | 0·13 |
| ***Respiratory, thoracic, and mediastinal disorders*** | **4** | 40·00 | **3** | 13·04 | **-0·27** | **-0·60** | **0·06** |
| Cough | 0 | 0·00 | 0 | 0·00 | 0·00 | -0·28 | 0·14 |
| Dyspnoea | 0 | 0·00 | 0 | 0·00 | 0·00 | -0·28 | 0·14 |
| Pneumonia - Pulmonary embolism | 0 | 0·00 | 1 | 4·35 | 0·04 | -0·04 | 0·13 |
| Respiratory failure | 4 | 40·00 | 0 | 0·00 | -0·40 | -0·70 | -0·10 |
| ***General disorders and administration site conditions*** | **0** | 0·00 | **0** | 0·00 | **0·00** | **-0·28** | **0·14** |
| Pyrexia | 0 | 0·00 | 0 | 0·00 | 0·00 | -0·28 | 0·14 |
| ***Injury, poisoning, and procedural complications*** | **1** | 10·00 | **4** | 17·39 | **0·07** | **-0·17** | **0·32** |
| Fall | 0 | 0·00 | 2 | 8·70 | 0·09 | -0·03 | 0·20 |
| Foot fracture | 1 | 10·00 | 0 | 0·00 | -0·10 | -0·29 | 0·09 |
| Leg fracture | 0 | 0·00 | 1 | 4·35 | 0·04 | -0·04 | 0·13 |
| Rib contusion | 0 | 0·00 | 0 | 0·00 | 0·00 | -0·28 | 0·14 |
| Accidental removal of percutaneous endoscopic gastrostomy tube | 0 | 0·00 | 1 | 4·35 | 0·04 | -0·04 | 0·13 |
| ***Musculoskeletal and connective tissue disorders*** | **0** | 0·00 | **0** | 0·00 | **0·00** | **-0·28** | **0·14** |
| Arthralgia | 0 | 0·00 | 0 | 0·00 | 0·00 | -0·28 | 0·14 |
| ***Psychiatric disorders*** | **0** | 0·00 | **0** | 0·00 | **0·00** | **-0·28** | **0·14** |
| Insomnia - Anxiety - Agitation | 0 | 0·00 | 0 | 0·00 | 0·00 | -0·28 | 0·14 |
| ***Renal and urinary disorders*** | **0** | 0·00 | **0** | 0·00 | **0·00** | **-0·28** | **0·14** |
| Bladder distension and urinary catheterization | 0 | 0·00 | 0 | 0·00 | 0·00 | -0·28 | 0·14 |
| Dysuria | 0 | 0·00 | 0 | 0·00 | 0·00 | -0·28 | 0·14 |
| ***Skin and subcutaneous tissue disorders*** | **0** | 0·00 | **0** | 0·00 | **0·00** | **-0·28** | **0·14** |
| Allergic reaction erythema | 0 | 0·00 | 0 | 0·00 | 0·00 | -0·28 | 0·14 |

**Supplementary Table 15: Treatment-emergent adverse events during the FETR-ALS trial between baseline and month 12, by treatment arm (FMT vs. SP) and categorized according to MedDRA classification. Intention-to treat population.**

| **Treatment-emergent adverse events, MedDRA preferred term** | **SP arm** | | **FMT arm** | | **Risk difference** | **95% CI** | |
| --- | --- | --- | --- | --- | --- | --- | --- |
|  | **(n = 14)** | | **(n = 28)** | |  |  |  |
|  | **N** | **%** | **N** | **%** |  |  |  |
| **Drop-outs** | 6 | 42·86 | 11 | 39·29 | -0·04 | -0·35 | 0·28 |
| Deaths/Tracheostomy | 3 | 21·43 | 1 | 3·57 | -0·18 | -0·40 | 0·05 |
| Withdrawals due to harms | 1 | 7·14 | 2 | 7·14 | 0·00 | -0·17 | 0·17 |
| **Nr participants at risk of harms at month 12** | 8 | 57·14 | 17 | 60·71 | 0·04 | -0·28 | 0·35 |
| **Nr patients with at least one AE** | 7 | 50·00 | 12 | 42·86 | -0·07 | -0·39 | 0·25 |
| **AEs** | 10 |  | 28 |  |  |  |  |
| **SAEs** | 4 |  | 4 |  |  |  |  |
| **Adverse Events of Special Interest** |  |  |  |  |  |  |  |
| ***Gastrointestinal disorders*** | **1** | **7·14** | **8** | **28·57** | **0·21** | **0·00** | **0·43** |
| Constipation | 0 | 0·00 | 1 | 3·57 | 0·04 | -0·03 | 0·10 |
| Faecaloma | 0 | 0·00 | 2 | 7·14 | 0·07 | -0·02 | 0·17 |
| Diarrhoea - Nausea | 1 | 7·14 | 2 | 7·14 | 0·00 | -0·17 | 0·17 |
| Hypogastrium distension - Hypogastrium pain | 0 | 0·00 | 1 | 3·57 | 0·04 | -0·03 | 0·10 |
| Chronic gastritis | 0 | 0·00 | 1 | 3·57 | 0·04 | -0·03 | 0·10 |
| Enteritis | 0 | 0·00 | 1 | 3·57 | 0·04 | -0·03 | 0·10 |
| ***Infections and infestations*** | **0** | **0·00** | **4** | **14·29** | **0·14** | **0·01** | **0·27** |
| Covid 19 | 0 | 0·00 | 1 | 3·57 | 0·04 | -0·03 | 0·10 |
| Peristomal infection | 0 | 0·00 | 1 | 3·57 | 0·04 | -0·03 | 0·10 |
| Gastroenteritis | 0 | 0·00 | 2 | 7·14 | 0·07 | -0·02 | 0·17 |
| **Others Adverse Events** |  |  |  |  |  |  |  |
| ***Metabolism and nutrition disorders*** | **0** | **0·00** | **2** | **7·14** | **0·07** | **-0·02** | **0·17** |
| Hypoglicemia | 0 | 0·00 | 1 | 3·57 | 0·04 | -0·03 | 0·10 |
| Dysphagia | 0 | 0·00 | 1 | 3·57 | 0·04 | -0·03 | 0·10 |
| ***Respiratory, thoracic, and mediastinal disorders*** | **9** | **64·29** | **8** | **28·57** | **-0·36** | **-0·66** | **-0·06** |
| Cough | 0 | 0·00 | 1 | 3·57 | 0·04 | -0·03 | 0·10 |
| Dyspnoea | 1 | 7·14 | 0 | 0·00 | -0·07 | -0·21 | 0·06 |
| Pneumonia - Pulmonary embolism | 1 | 7·14 | 6 | 21·43 | 0·14 | -0·06 | 0·35 |
| Respiratory failure | 7 | 50·00 | 1 | 3·57 | -0·46 | -0·74 | -0·19 |
| ***General disorders and administration site conditions*** | **0** | **0·00** | **1** | **3·57** | **0·04** | **-0·03** | **0·10** |
| Pyrexia | 0 | 0·00 | 1 | 3·57 | 0·04 | -0·03 | 0·10 |
| ***Injury, poisoning, and procedural complications*** | **3** | **21·43** | **5** | **17·86** | **-0·04** | **-0·29** | **0·22** |
| Fall | 2 | 14·29 | 2 | 7·14 | -0·07 | -0·28 | 0·14 |
| Foot fracture | 1 | 7·14 | 0 | 0·00 | -0·07 | -0·21 | 0·06 |
| Leg fracture | 0 | 0·00 | 1 | 3·57 | 0·04 | -0·03 | 0·10 |
| Rib contusion | 0 | 0·00 | 1 | 3·57 | 0·04 | -0·03 | 0·10 |
| Accidental removal of percutaneous endoscopic gastrostomy tube | 0 | 0·00 | 1 | 3·57 | 0·04 | -0·03 | 0·10 |
| ***Musculoskeletal and connective tissue disorders*** | **0** | **0·00** | **1** | **3·57** | **0·04** | **-0·03** | **0·10** |
| Arthralgia | 0 | 0·00 | 1 | 3·57 | 0·04 | -0·03 | 0·10 |
| ***Psychiatric disorders*** | **0** | **0·00** | **1** | **3·57** | **0·04** | **-0·03** | **0·10** |
| Insomnia - Anxiety - Agitation | 0 | 0·00 | 1 | 3·57 | 0·04 | -0·03 | 0·10 |
| ***Renal and urinary disorders*** | **1** | **7·14** | **1** | **3·57** | **-0·04** | **-0·19** | **0·12** |
| Bladder distension and urinary catheterization | 1 | 7·14 | 0 | 0·00 | -0·07 | -0·21 | 0·06 |
| Dysuria | 0 | 0·00 | 1 | 3·57 | 0·04 | -0·03 | 0·10 |
| ***Skin and subcutaneous tissue disorders*** | **0** | **0·00** | **1** | **3·57** | **0·04** | **-0·03** | **0·10** |
| Allergic reaction erythema | 0 | 0·00 | 1 | 3·57 | 0·04 | -0·03 | 0·10 |

**Supplementary Table 16. Mean changes in biological outcomes between FMT and SP arm from baseline to month 6 an 12. Intention- to-treat analysis**

|  | **At 6 months** | | | | | **At 12 months** | | | | |
| --- | --- | --- | --- | --- | --- | --- | --- | --- | --- | --- |
| **Biological outcome** | **MD*** | **SE** | **95% CI** | | **P value** | **MD*** | **SE** | **95% CI** | | **P value** |
| NfH Serum (pg/ml) | -1355·54 | 899·84 | -3198·78 | 487·70 | 0·14 | -2155·26 | 1162·85 | -4580·93 | 270·4 | 0·079 |
| NfL Serum (pg/ml) | -20·06 | 33·35 | -88·36 | 48·25 | 0·55 | -45·16 | 29·36 | -106·4 | 16·08 | 0·14 |
| Creatinine (mg/dl) | -0·07 | 0·07 | -0·21 | 0·07 | 0·31 | 0·01 | 0·09 | -0·17 | 0·20 | 0·89 |
| CRP (mg/dl) | 0·06 | 0·22 | -0·38 | 0·50 | 0·79 | -0·13 | 0·17 | -0·48 | 0·22 | 0·45 |
| Albumin (g/dl) | 0·02 | 0·09 | -0·16 | 0·19 | 0·85 | 0·03 | 0·13 | -0·25 | 0·31 | 0·81 |
| Total Cholesterol (mg/dl) | 7·76 | 20·75 | -34·88 | 50·41 | 0·71 | 9·93 | 21·95 | -36·02 | 55·88 | 0·66 |
| LDL Cholesterol (mg/dl) | 15·94 | 15·37 | -15·73 | 47·60 | 0·31 | 4·89 | 15·28 | -27·21 | 36·99 | 0·75 |
| Triglycerides (mg/dl) | 6·76 | 33·56 | -62·23 | 75·75 | 0·84 | 12·93 | 21·18 | -31·4 | 57·26 | 0·55 |
| Vitamin D (ng/ml) | 1·02 | 5·57 | -10·45 | 12·50 | 0·86 | 2·20 | 12·57 | -24·32 | 28·72 | 0·86 |

For these analysis, the mean differences between each biological outcome in the FMT-treated and sham procedure arm were assessed using univariable linear regression models between month 6 and baseline and month 12 and baseline. *: difference between FMT versus SP arm. Wald tests were used to test the null hypothesis that regression coefficients were equal to 0 and obtained p-values reported.

Given the exploratory nature of these analyses, no adjustment for multiple comparisons was performed and nominal p-values were reported. Abbreviations: FMT: faecal microbiota transplantation; SP: Sham Procedure.SE: standard error; CI: confidence interval; P= P Values.

#### Supplementary Table 17. Average variations of neurofilaments over time in FMT and SP arms, and the mean difference between the two arms, as estimated by longitudinal models. Intention-to-treat analysis

| **Outcome** |  | **Estimate** | **SE** | **95% CI** | | **P value** |
| --- | --- | --- | --- | --- | --- | --- |
| NfH Serum, pg/ml | Monthly variation SP | 64·62 | 38·05 | -10·13 | 138·98 | 0·092 |
|  | Monthly variation FMT | 15·48 | 26·76 | -37·36 | 67·63 | 0·56 |
|  | Mean difference in monthly variation | -49·14 | 46·52 | -140·43 | 41·85 | 0·29 |
| NfH CSF, pg/ml | Monthly variation SP | 294·13 | 157·32 | 0·21 | 569·32 | 0·20 |
|  | Monthly variation FMT | 196·06 | 157·46 | -102·74 | 469·56 | 0·34 |
|  | Mean difference in monthly variation | -98·07 | 222·58 | -502·02 | 299·72 | 0·70 |
| NfL Serum, pg/ml | Monthly variation SP | 1·45 | 1·00 | -0·52 | 3·41 | 0·15 |
|  | Monthly variation FMT | 0·91 | 0·70 | -0·48 | 2·28 | 0·20 |
|  | Mean difference in monthly variation | -0·54 | 1·22 | -2·95 | 1·85 | 0·66 |
| NfL CSF, pg/ml | Monthly variation SP | 43·78 | 75·04 | -94·32 | 176·1 | 0·62 |
|  | Monthly variation FMT | -207·93 | 75·08 | -345·47 | -74·98 | 0·11 |
|  | Mean difference in monthly variation | -251·71 | 106·15 | -442·03 | -60·19 | 0·14 |

Monthly variations of NfH and NfL, measured in serum and cerebrospinal fluid (CSF), in Sham and FMT arm, and differences between the Monthly variations in the two treatment arms were estimated by linear mixed-effect models with random intercept. These models included as covariates arm, time and the interaction arm x time. T tests were used to test the null hypothesis that regression coefficients were equal to 0 and obtained p-values reported. Satterthwaite approximation was used to estimate degrees of freedom.

Abbreviations: CI: confidence interval; FMT: faecal microbiota transplantation; NfL: neurofilament light chain; NffH: neurofilament heavy chain; SE: standard error; SP: Sham Procedure.

#### Supplementary Table 18. Changes from baseline to month 6 and 12 in Treg fractions over Th1 and Th17 immune cells subpopulations across treatment arms. Intention to treat analysis.

| **Treg fractions** | **Time point** | **Arm** |  | | | **absolute change from baseline** | | | |
| --- | --- | --- | --- | --- | --- | --- | --- | --- | --- |
|  |  |  | **N** | **Mean ratio** | **SE** | **Ratio between arms** | **95% CI** | | **p** |
| **Treg/Th1** | Month 6 | SP | 10 | 1·19 | 1·20 | - | - | - | - |
|  |  | FMT | 18 | 3·07 | 1·92 | 2·58 | 0·46 | 13·78 | 0·28 |
|  | Month 12 | SP | 1 | -^§^ | -^§^ | - | - | - | - |
|  |  | FMT | 10 | 0·68 | 0·51 | -^§^ | -^§^ | -^§^ | -^§^ |
| **Treg/Th17** | Month 6 | SP | 10 | 0·85 | 0·51 | - | - | - | - |
|  |  | FMT | 17 | 1·23 | 0·50 | 1·45 | 0·43 | 4·79 | 0·55 |
|  | Month 12 | SP | 1 | -^§^ | -^§^ | - | - | - | - |
|  |  | FMT | 10 | 1·12 | 0·66 | -^§^ | -^§^ | -^§^ | -^§^ |

Mean absolute changes from baseline to month 6 and 12 are showed for each treatment group and comparison were performed using linear mixed- effects regression models that include indicator variables for treatment arms. time and the interaction time x arm as independent variables. T tests were used to test the null hypothesis that regression coefficients were equal to 0 and obtained p-values reported. Satterthwaite approximation was used to estimate degrees of freedom.

Abbreviations: N: number of samples for analysis; MD = Mean Difference; se = Standard Error; CI = Confidence Interval; SP: Sham Procedure; FMT: Faecal Microbiota Transplantation.

^§^ these values couldn’t be calculated as only one observation per SP arm was available.

#### Supplementary Table 19. Mean changes in circulating Treg/Th1 and Treg/Th17 ratios in FMT and SP arms, considering their baseline variations, mean monthly variations in the two arms, and mean monthly variations between the two arms, when estimated considering baseline and month 6. Intention-to-treat analysis.

| **Outcome** |  | **Estimate** | **SE** | **95% CI** | | **P value** |
| --- | --- | --- | --- | --- | --- | --- |
| **Treg/Th1** | Mean ratio between FMT and SP | 1·74 | 1·37 | 0·55 | 5·55 | 0·36 |
|  | Monthly variation in SP arm (mean ratio) | 1·03 | 0·12 | 0·82 | 1·30 | 0·81 |
|  | Monthly variation in FMT arm (mean ratio) | 1·21 | 0·10 | 1·02 | 1·42 | 0·035 |
|  | Mean ratio of monthly variation between FMT and SP | 1·17 | 0·18 | 0·88 | 1·55 | 0·28 |
| **Treg/Th17** | Mean ratio between FMT and SP | 1·13 | 0·52 | 0·51 | 2·48 | 0·76 |
|  | Monthly variation in SP arm (mean ratio) | 0·97 | 0·08 | 0·83 | 1·15 | 0·74 |
|  | Monthly variation in FMT arm (mean ratio) | 1·03 | 0·06 | 0·92 | 1·17 | 0·58 |
|  | Mean ratio of monthly variation between FMT and SP | 1·06 | 0·11 | 0·87 | 1·3 | 0·55 |

For these analyses, laboratory parameters were scaled using a logarithmic transformation, to handle their strongly left-skewed distributions. The term "mean ratio" indicates the ratio of the geometric mean of FMT arm and the geometric mean of the SP arm. Monthly variations and differences between monthly were estimated using linear mixed-effects models with random intercept. These models included as covariates arm, time and the interaction arm x time. T tests were used to test the null hypothesis that regression coefficients were equal to 0 and obtained p-values reported. Satterthwaite approximation was used to estimate degrees of freedom.

Abbreviations: CI: confidence interval; FMT: faecal microbiota transplantation; SP: sham procedure; SE: standard error; Treg: T regulatory; Th1: T helper -1; Th17: T-helper 17.

#### Supplementary Table 20. Changes from baseline to month 6 and 12 of all circulating immune cell subpopulations across treatment arms. Intention to treat analysis.

| **T cells** | **Time point** | **Arm** |  | | | **absolute change from baseline** | | | |
| --- | --- | --- | --- | --- | --- | --- | --- | --- | --- |
|  |  |  | **N** | **Mean** | **SE** | **MD** | **95% CI** | | **P value** |
| CD45+  (% over live) | Month 6 | SP | 10 | -12·95 | 4·58 | - | - | - | - |
|  |  | FMT | 19 | 4·21 | 3·30 | 17·16 | 6·16 | 28·21 | 0·0050 |
|  | Month 12 | SP | 1 | -^§^ | -^§^ | - | - | - | - |
|  |  | FMT | 10 | 4·68 | 4·60 | -^§^ | -^§^ | -^§^ | -^§^ |
| CD3+  (% over CD45+) | Month 6 | SP | 10 | 10·09 | 4·32 | - | - | - | - |
|  |  | FMT | 19 | 2·05 | 3·13 | -8·04 | -18·38 | 2·51 | 0·14 |
|  | Month 12 | SP | 1 | -^§^ | -^§^ | - | - | - | - |
|  |  | FMT | 10 | 8·60 | 4·60 | -^§^ | -^§^ | -^§^ | -^§^ |
| CD4+  (% over CD45+) | Month 6 | SP | 10 | 10·47 | 3·31 | - | - | - | - |
|  |  | FMT | 19 | -0·42 | 2·39 | -10·89 | -18·79 | -2·78 | 0·013 |
|  | Month 12 | SP | 1 | -^§^ | -^§^ | - | - | - | - |
|  |  | FMT | 10 | 1·63 | 2·76 | -^§^ | -^§^ | -^§^ | -^§^ |
| CD8+CD4+ (% over CD3) | Month 6 | SP | 10 | 0·03 | 1·00 | - | - | - | - |
|  |  | FMT | 19 | 1·03 | 0·72 | 1·01 | -1·38 | 3·40 | 0·42 |
|  | Month 12 | SP | 1 | -^§^ | -^§^ | - | - | - | - |
|  |  | FMT | 10 | 0·07 | 0·29 | -^§^ | -^§^ | -^§^ | -^§^ |
| CD4+  (% over CD3) | Month 6 | SP | 10 | 1·76 | 1·76 | - | - | - | - |
|  |  | FMT | 19 | 0·03 | 1·27 | -1·73 | -5·94 | 2·55 | 0·43 |
|  | Month 12 | SP | 1 | -^§^ | -^§^ | - | - | - | - |
|  |  | FMT | 10 | -3·27 | 3·38 | -^§^ | -^§^ | -^§^ | -^§^ |
| Th0  (% over CD4+) | Month 6 | SP | 10 | 1·64 | 3·11 | - | - | - | - |
|  |  | FMT | 19 | 1·74 | 2·23 | 0·10 | -7·29 | 7·50 | 0·98 |
|  | Month 12 | SP | 1 | -^§^ | -^§^ | - | - | - | - |
|  |  | FMT | 10 | 1·52 | 2·71 | -^§^ | -^§^ | -^§^ | -^§^ |
| CxCR3+  (% over CD4+) | Month 6 | SP | 10 | -0·48 | 3·19 | - | - | - | - |
|  |  | FMT | 19 | -2·06 | 2·29 | -1·58 | -9·19 | 6·09 | 0·69 |
|  | Month 12 | SP | 1 | -^§^ | -^§^ | - | - | - | - |
|  |  | FMT | 10 | 0·35 | 3·94 | -^§^ | -^§^ | -^§^ | -^§^ |
| Th1/Th17 | Month 6 | SP | 10 | -0·23 | 0·21 | - | - | - | - |
|  |  | FMT | 19 | -0·12 | 0·15 | 0·12 | -0·37 | 0·62 | 0·65 |
|  | Month 12 | SP | 1 | -^§^ | -^§^ | - | - | - | - |
|  |  | FMT | 10 | 0·02 | 0·26 | -^§^ | -^§^ | -^§^ | -^§^ |
| Th1  (% over CD4+) | Month 6 | SP | 10 | -0·56 | 1·05 | - | - | - | - |
|  |  | FMT | 19 | -0·65 | 0·75 | -0·08 | -2·58 | 2·45 | 0·95 |
|  | Month 12 | SP | 1 | -^§^ | -^§^ | - | - | - | - |
|  |  | FMT | 10 | -0·45 | 1·22 | -^§^ | -^§^ | -^§^ | -^§^ |
| CCR4+  (% over CD4+) | Month 6 | SP | 10 | 7·03 | 9·72 | - | - | - | - |
|  |  | FMT | 19 | 12·52 | 6·99 | 5·49 | -17·98 | 28·78 | 0·65 |
|  | Month 12 | SP | 1 | -^§^ | -^§^ | - | - | - | - |
|  |  | FMT | 10 | 3·31 | 9·91 | -^§^ | -^§^ | -^§^ | -^§^ |
| Th17  (% over CD4+) | Month 6 | SP | 10 | -0·16 | 8·55 | - | - | - | - |
|  |  | FMT | 19 | -5·71 | 6·14 | -5·55 | -25·86 | 14·77 | 0·60 |
|  | Month 12 | SP | 1 | -^§^ | -^§^ | - | - | - | - |
|  |  | FMT | 10 | -3·40 | 6·09 | -^§^ | -^§^ | -^§^ | -^§^ |
| Th2  (% over CD4+) | Month 6 | SP | 10 | 4·70 | 9·04 | - | - | - | - |
|  |  | FMT | 19 | 19·22 | 6·51 | 14·52 | -7·69 | 36·04 | 0·20 |
|  | Month 12 | SP | 1 | -^§^ | -^§^ | - | - | - | - |
|  |  | FMT | 10 | 8·22 | 9·01 | -^§^ | -^§^ | -^§^ | -^§^ |
| Th9  (% over CD4+) | Month 6 | SP | 10 | 0·25 | 2·18 | - | - | - | - |
|  |  | FMT | 19 | -2·29 | 1·57 | -2·54 | -7·76 | 2·75 | 0·35 |
|  | Month 12 | SP | 1 | -^§^ | -^§^ | - | - | - | - |
|  |  | FMT | 10 | -3·45 | 1·81 | -^§^ | -^§^ | -^§^ | -^§^ |
| CCR6+ CCR4+  (% over CD4+) | Month 6 | SP | 10 | 0·93 | 9·35 | - | - | - | - |
|  |  | FMT | 19 | -3·62 | 6·71 | -4·55 | -26·76 | 17·66 | 0·69 |
|  | Month 12 | SP | 1 | -^§^ | -^§^ | - | - | - | - |
|  |  | FMT | 10 | -5·85 | 7·58 | -^§^ | -^§^ | -^§^ | -^§^ |
| Th22  (% over CD4+) | Month 6 | SP | 10 | 0·15 | 0·41 | - | - | - | - |
|  |  | FMT | 19 | -0·37 | 0·29 | -0·52 | -1·48 | 0·48 | 0·31 |
|  | Month 12 | SP | 1 | -^§^ | -^§^ | - | - | - | - |
|  |  | FMT | 10 | -0·30 | 0·36 | -^§^ | -^§^ | -^§^ | -^§^ |
| CD8+  (% over CD3) | Month 6 | SP | 10 | -1·35 | 1·89 | - | - | - | - |
|  |  | FMT | 19 | -1·48 | 1·37 | -0·13 | -4·75 | 4·41 | 0·96 |
|  | Month 12 | SP | 1 | -^§^ | -^§^ | - | - | - | - |
|  |  | FMT | 10 | -0·44 | 1·74 | -^§^ | -^§^ | -^§^ | -^§^ |
| Tc0  (% over CD8+) | Month 6 | SP | 10 | 1·40 | 3·72 | - | - | - | - |
|  |  | FMT | 19 | 2·50 | 2·67 | 1·10 | -7·81 | 10·01 | 0·82 |
|  | Month 12 | SP | 1 | -^§^ | -^§^ | - | - | - | - |
|  |  | FMT | 10 | 4·19 | 2·87 | -^§^ | -^§^ | -^§^ | -^§^ |
| CXCR3  (% over CD8+) | Month 6 | SP | 10 | -0·33 | 3·41 | - | - | - | - |
|  |  | FMT | 19 | -4·23 | 2·45 | -3·90 | -12·00 | 4·19 | 0·35 |
|  | Month 12 | SP | 1 | -^§^ | -^§^ | - | - | - | - |
|  |  | FMT | 10 | -4·13 | 3·94 | -^§^ | -^§^ | -^§^ | -^§^ |
| Tc1/Tc17 | Month 6 | SP | 10 | -0·02 | 0·74 | - | - | - | - |
|  |  | FMT | 19 | -0·85 | 0·54 | -0·83 | -2·61 | 0·98 | 0·37 |
|  | Month 12 | SP | 1 | -^§^ | -^§^ | - | - | - | - |
|  |  | FMT | 10 | -1·10 | 0·84 | -^§^ | -^§^ | -^§^ | -^§^ |
| Tc1  (% over CD8+) | Month 6 | SP | 10 | -0·15 | 2·09 | - | - | - | - |
|  |  | FMT | 19 | -1·65 | 1·50 | -1·50 | -6·46 | 3·45 | 0·56 |
|  | Month 12 | SP | 1 | -^§^ | -^§^ | - | - | - | - |
|  |  | FMT | 10 | -0·10 | 0·78 | -^§^ | -^§^ | -^§^ | -^§^ |
| CCR4+  (% over CD8+) | Month 6 | SP | 10 | 16·21 | 10·81 | - | - | - | - |
|  |  | FMT | 19 | 19·09 | 7·79 | 2·87 | -23·33 | 28·80 | 0·83 |
|  | Month 12 | SP | 1 | -^§^ | -^§^ | - | - | - | - |
|  |  | FMT | 10 | 21·44 | 10·11 | -^§^ | -^§^ | -^§^ | -^§^ |
| Tc17  (% over CD8+) | Month 6 | SP | 10 | 16·16 | 12·10 | - | - | - | - |
|  |  | FMT | 19 | 13·36 | 8·68 | -2·80 | -31·54 | 25·93 | 0·85 |
|  | Month 12 | SP | 1 | -^§^ | -^§^ | - | - | - | - |
|  |  | FMT | 10 | 9·14 | 9·67 | -^§^ | -^§^ | -^§^ | -^§^ |
| Tc2  (% over CD8+) | Month 6 | SP | 10 | 0·06 | 11·77 | - | - | - | - |
|  |  | FMT | 19 | 4·30 | 8·45 | 4·23 | -23·72 | 32·19 | 0·77 |
|  | Month 12 | SP | 1 | -^§^ | -^§^ | - | - | - | - |
|  |  | FMT | 10 | 6·42 | 10·24 | -^§^ | -^§^ | -^§^ | -^§^ |
| Tc9  (% over CD8+) | Month 6 | SP | 10 | -15·94 | 9·81 | - | - | - | - |
|  |  | FMT | 19 | -14·29 | 7·07 | 1·66 | -21·99 | 25·32 | 0·89 |
|  | Month 12 | SP | 1 | -^§^ | -^§^ | - | - | - | - |
|  |  | FMT | 10 | -21·45 | 9·47 | -^§^ | -^§^ | -^§^ | -^§^ |
| CCR4+ CCR6+  (% over CD8+) | Month 6 | SP | 10 | 20·42 | 11·61 | - | - | - | - |
|  |  | FMT | 19 | 18·74 | 8·35 | -1·68 | -29·35 | 26·72 | 0·91 |
|  | Month 12 | SP | 1 | -^§^ | -^§^ | - | - | - | - |
|  |  | FMT | 10 | 15·54 | 10·97 | -^§^ | -^§^ | -^§^ | -^§^ |
| Tc22  (% over CD8+) | Month 6 | SP | 10 | 0·33 | 0·19 | - | - | - | - |
|  |  | FMT | 19 | 0·14 | 0·14 | -0·19 | -0·65 | 0·28 | 0·43 |
|  | Month 12 | SP | 1 | -^§^ | -^§^ | - | - | - | - |
|  |  | FMT | 10 | 1·21 | 0·46 | -^§^ | -^§^ | -^§^ | -^§^ |

Mean absolute changes from baseline to month 6 and 12 are showed for each treatment group and comparisons were performed using linear mixed- effects regression models that include indicator variables for treatment arms, time and the interaction time x arm as independent variables. T tests were used to test the null hypothesis that regression coefficients were equal to 0 and obtained p-values reported. Satterthwaite approximation was used to estimate degrees of freedom. Given the exploratory nature of these analyses, no adjustment for multiple comparisons was performed and nominal p-values were reported. Abbreviations: N: number of samples for analysis; MD = Mean Difference; se = Standard Error; CI = Confidence Interval; SP: Sham Procedure; FMT: Faecal Microbiota Transplantation.

^§^ these values couldn’t be calculated as only one observation per SP arm was available.

#### Supplementary Table 21. Mean variations of circulating T cell subpopulations at baseline and over time (from baseline to month 6), in FMT and SP arms, and from the comparison of the treatment arms over time. Intention- to-treat analysis

| **Immune cells populations** |  | **Estimate** | **Std· Error** | **95% Conf· Int·** | | **P value** | |
| --- | --- | --- | --- | --- | --- | --- | --- |
| CD45+ (% over live) | Mean difference between FMT and SP | -4·57 | 4·38 | -13·05 | 3·92 | 0·30 | |
|  | Monthly variation SP | -2·16 | 0·76 | -3·64 | -0·65 | 0·0080 | |
|  | Monthly variation FMT | 0·7 | 0·55 | -0·36 | 1·80 | 0·21 | |
|  | Mean difference in monthly variation | 2·86 | 0·94 | 1·03 | 4·70 | 0·0051 | |
| CD4+ (% over CD45+) | Mean difference between FMT and SP | 9·27 | 4·78 | -0·05 | 18·58 | 0·059 | |
|  | Monthly variation SP | 1·74 | 0·55 | 0·65 | 2·81 | 0·0042 | |
|  | Monthly variation FMT | -0·07 | 0·4 | -0·85 | 0·71 | 0·86 | |
|  | Mean difference in monthly variation | -1·81 | 0·68 | -3·13 | -0·46 | 0·013 | |
| CD3+ (% CD45+) | Mean difference between FMT and SP | 8·76 | 5·85 | -2·61 | 20·13 | 0·14 | |
|  | Monthly variation SP | 1·68 | 0·72 | 0·24 | 3·07 | 0·026 | |
|  | Monthly variation FMT | 0·34 | 0·52 | -0·68 | 1·36 | 0·52 | |
|  | Mean difference in monthly variation | -1·34 | 0·89 | -3·06 | 0·42 | 0·14 | |
| CD4+ (% CD3) | Mean difference between FMT and SP | 2·62 | 4·4 | -5·97 | 11·22 | 0·55 | |
|  | Monthly variation SP | 0·29 | 0·29 | -0·28 | 0·86 | 0·32 | |
|  | Monthly variation FMT | 0·01 | 0·21 | -0·41 | 0·43 | 0·98 | |
|  | Mean difference in monthly variation | -0·29 | 0·36 | -0·99 | 0·43 | 0·43 | |
| Th0 (% CD4+) | Mean difference between FMT and SP | 0·4 | 2·46 | -4·35 | 5·15 | 0·87 | |
|  | Monthly variation SP | 0·27 | 0·52 | -0·73 | 1·28 | 0·60 | |
|  | Monthly variation FMT | 0·29 | 0·37 | -0·43 | 1·01 | 0·44 | |
|  | Mean difference in monthly variation | 0·02 | 0·64 | -1·22 | 1·25 | 0·98 | |
| CxCR3+ (% CD4+) | Mean difference between FMT and SP | 1·57 | 2·55 | -3·35 | 6·49 | 0·54 | |
|  | Monthly variation SP | -0·08 | 0·53 | -1·11 | 0·95 | 0·88 | |
|  | Monthly variation FMT | -0·34 | 0·38 | -1·08 | 0·40 | 0·37 | |
|  | Mean difference in monthly variation | -0·26 | 0·65 | -1·53 | 1·01 | 0·69 | |
| Th1/Th17 (%CD4+) | Mean difference between FMT and SP | -0·04 | 0·22 | -0·47 | 0·38 | 0·84 | |
|  | Monthly variation SP | -0·04 | 0·03 | -0·11 | 0·03 | 0·27 | |
|  | Monthly variation FMT | -0·02 | 0·02 | -0·07 | 0·03 | 0·44 | |
|  | Mean difference in monthly variation | 0·02 | 0·04 | -0·06 | 0·10 | 0·65 | |
| Th1 (% CD4+) | Mean difference between FMT and SP | -0·46 | 0·97 | -2·33 | 1·41 | 0·64 | |
|  | Monthly variation SP | -0·09 | 0·17 | -0·43 | 0·25 | 0·59 | |
|  | Monthly variation FMT | -0·11 | 0·13 | -0·35 | 0·14 | 0·40 | |
|  | Mean difference in monthly variation | -0·01 | 0·21 | -0·43 | 0·41 | 0·95 | |
| CCR4+ (% CD4+) | Mean difference between FMT and SP | -3·35 | 8·26 | -19·3 | 12·6 | 0·69 | |
|  | Monthly variation SP | 1·17 | 1·62 | -1·99 | 4·33 | 0·47 | |
|  | Monthly variation FMT | 2·09 | 1·16 | -0·21 | 4·35 | 0·082 | |
|  | Mean difference in monthly variation | 0·91 | 1·99 | -3·00 | 4·80 | 0·65 | |
| Th17 (% CD4+) | Mean difference between FMT and SP | 0·34 | 6·76 | -12·71 | 13·39 | 0·96 | |
|  | Monthly variation SP | -0·03 | 1·43 | -2·78 | 2·72 | 0·98 | |
|  | Monthly variation FMT | -0·95 | 1·02 | -2·93 | 1·02 | 0·36 | |
|  | Mean difference in monthly variation | -0·92 | 1·75 | -4·31 | 2·46 | 0·60 | |
| Th2 (% CD4+) | Mean difference between FMT and SP | -4·55 | 7·93 | -19·86 | 10·76 | 0·57 | |
|  | Monthly variation SP | 0·78 | 1·51 | -2·13 | 3·79 | 0·61 | |
|  | Monthly variation FMT | 3·2 | 1·08 | 1·08 | 5·31 | 0·0061 | |
|  | Mean difference in monthly variation | 2·42 | 1·86 | -1·28 | 6·01 | 0·20 | |
| Th9 (% CD4+) | Mean difference between FMT and SP | 2·42 | 1·80 | -1·05 | 5·89 | 0·18 | |
|  | Monthly variation SP | 0·04 | 0·36 | -0·67 | 0·75 | 0·91 | |
|  | Monthly variation FMT | -0·38 | 0·26 | -0·89 | 0·13 | 0·15 | |
|  | Mean difference in monthly variation | -0·42 | 0·45 | -1·29 | 0·46 | 0·35 | |
| CCR6+ CCR4+ (% CD4+) | Mean difference between FMT and SP | 2·15 | 7·39 | -12·12 | 16·41 | 0·77 | |
|  | Monthly variation SP | 0·15 | 1·56 | -2·85 | 3·16 | 0·92 | |
|  | Monthly variation FMT | -0·6 | 1·12 | -2·76 | 1·56 | 0·59 | |
|  | Mean difference in monthly variation | -0·76 | 1·92 | -4·46 | 2·94 | 0·69 | |
| Th22 (% CD4+) | Mean difference between FMT and SP | 0·1 | 0·36 | -0·6 | 0·79 | 0·79 | |
|  | Monthly variation SP | 0·02 | 0·07 | -0·11 | 0·16 | 0·71 | |
|  | Monthly variation FMT | -0·06 | 0·05 | -0·16 | 0·03 | 0·22 | |
|  | Mean difference in monthly variation | -0·09 | 0·08 | -0·25 | 0·08 | 0·31 | |
| CD8+ (% CD3) | Mean difference between FMT and SP | -0·9 | 3·55 | -7·81 | 6·02 | 0·80 | |
|  | Monthly variation SP | -0·23 | 0·32 | -0·84 | 0·40 | 0·48 | |
|  | Monthly variation FMT | -0·25 | 0·23 | -0·7 | 0·20 | 0·29 | |
|  | Mean difference in monthly variation | -0·02 | 0·39 | -0·79 | 0·74 | 0·96 | |
| Tc0 (% CD8+) | Mean difference between FMT and SP | -0·92 | 2·97 | -6·67 | 4·82 | 0·76 | |
|  | Monthly variation SP | 0·23 | 0·62 | -0·98 | 1·44 | 0·71 | |
|  | Monthly variation FMT | 0·42 | 0·44 | -0·45 | 1·28 | 0·35 | |
|  | Mean difference in monthly variation | 0·18 | 0·76 | -1·30 | 1·67 |  | 0·81 |

| CXCR3 (% CD8+) | Mean difference between FMT and SP | 3·45 | 2·69 | -1·75 | 8·65 | 0·20 | |
| --- | --- | --- | --- | --- | --- | --- | --- |
|  | Monthly variation SP | -0·05 | 0·57 | -1·15 | 1·04 | 0·92 | |
|  | Monthly variation FMT | -0·71 | 0·41 | -1·49 | 0·08 | 0·088 | |
|  | Mean difference in monthly variation | -0·65 | 0·7 | -2·00 | 0·70 | 0·35 | |
| Tc1/Tc17 (% CD8+) | Mean difference between FMT and SP | 0·38 | 0·78 | -1·13 | 1·9 | 0·62 | |
|  | Monthly variation SP | 0·00 | 0·12 | -0·25 | 0·24 | 0·98 | |
|  | Monthly variation FMT | -0·14 | 0·09 | -0·32 | 0·03 | 0·12 | |
|  | Mean difference in monthly variation | -0·14 | 0·15 | -0·44 | 0·16 | 0·37 | |
| Tc1 (% CD8+) | Mean difference between FMT and SP | 1·20 | 1·65 | -1·98 | 4·38 | 0·47 | |
|  | Monthly variation SP | -0·03 | 0·35 | -0·70 | 0·65 | 0·94 | |
|  | Monthly variation FMT | -0·28 | 0·25 | -0·76 | 0·21 | 0·27 | |
|  | Mean difference in monthly variation | -0·25 | 0·43 | -1·08 | 0·58 | 0·56 | |
| CCR4+ (% CD8+) | Mean difference between FMT and SP | -1·17 | 10·04 | -20·58 | 18·25 | 0·91 | |
|  | Monthly variation SP | 2·70 | 1·80 | -0·81 | 6·23 | 0·14 | |
|  | Monthly variation FMT | 3·18 | 1·30 | 0·63 | 5·70 | 0·020 | |
|  | Mean difference in monthly variation | 0·48 | 2·22 | -3·89 | 4·80 | 0·83 | |
| Tc17 (% CD8+) | Mean difference between FMT and SP | 2·81 | 9·56 | -15·65 | 21·27 | 0·77 | |
|  | Monthly variation SP | 2·69 | 2·02 | -1·20 | 6·58 | 0·19 | |
|  | Monthly variation FMT | 2·23 | 1·45 | -0·57 | 5·02 | 0·13 | |
|  | Mean difference in monthly variation | -0·47 | 2·48 | -5·26 | 4·32 | 0·85 | |
| Tc2 (% CD8+) | Mean difference between FMT and SP | -3·46 | 9·3 | -21·41 | 14·49 | 0·71 | |
|  | Monthly variation SP | 0·01 | 1·96 | -3·77 | 3·80 | 0·99 | |
|  | Monthly variation FMT | 0·72 | 1·41 | -2·00 | 3·43 | 0·61 | |
|  | Mean difference in monthly variation | 0·71 | 2·41 | -3·95 | 5·36 | 0·77 | |
| Tc9 (% CD8+) | Mean difference between FMT and SP | -0·54 | 9·26 | -18·45 | 17·37 | 0·95 | |
|  | Monthly variation SP | -2·66 | 1·64 | -5·85 | 0·54 | 0·11 | |
|  | Monthly variation FMT | -2·38 | 1·18 | -4·68 | -0·07 | 0·052 | |
|  | Mean difference in monthly variation | 0·28 | 2·02 | -3·67 | 4·22 | 0·89 | |
| CCR4+ CCR6+ (% CD8+) | Mean difference between FMT and SP | 0·54 | 9·81 | -18·4 | 19·49 | 0·96 | |
|  | Monthly variation SP | 3·40 | 1·93 | -0·46 | 7·14 | 0·088 | |
|  | Monthly variation FMT | 3·12 | 1·39 | 0·40 | 5·84 | 0·031 | |
|  | Mean difference in monthly variation | -0·28 | 2·38 | -4·89 | 4·45 | 0·91 | |
| Tc22 (% CD8+) | Mean difference between FMT and SP | -0·08 | 0·23 | -0·53 | 0·38 | 0·74 | |
|  | Monthly variation SP | 0·06 | 0·03 | -0·01 | 0·12 | 0·10 | |
|  | Monthly variation FMT | 0·02 | 0·02 | -0·02 | 0·07 | 0·33 | |
|  | Mean difference in monthly variation | -0·03 | 0·04 | -0·11 | 0·05 |  | 0·43 |

Monthly variations and differences between monthly variations in each T cell subpopulation were estimated by linear mixed-effects models with random intercept. These models included as covariates arm, time and the interaction arm x time. Monthly variations were computed between baseline and month 6, as measurements at month 12 were too scarce. Mean differences between FMT and sham procedure patients are displayed at baseline, then monthly variations in SP and FMT-patients are showed, and finally the mean difference in the monthly variation between the two arms. T tests were used to test the null hypothesis that regression coefficients were equal to 0 and obtained p-values reported. Satterthwaite approximation was used to estimate degrees of freedom. Statistically significant differences were highlighted in bold characters.

Abbreviations: Conf. Int.: confidence interval; FMT: faecal microbiota transplantation; SP: sham procedure; Std. Error: standard error; Th: T-helper; Tc: T cytotoxic.

#### Supplementary Table 22. Changes from baseline to month 6 and 12 of intestinal immune cell subpopulations across treatment arms. Intention to treat analysis.

| **Intestinal T cells** | **Time point** | **Arm** |  | | | **absolute change from baseline** | | | |
| --- | --- | --- | --- | --- | --- | --- | --- | --- | --- |
|  |  |  | **N** | **Mean** | **SE** | **MD** | **95% CI** | | **P value** |
| CD3+  (% over CD45+) | Month 6 | SP | 10 | 8·39 | 6·94 | - | - | - | - |
|  |  | FMT | 19 | 12·07 | 5·04 | 3·68 | -13·13 | 20·32 | 0·67 |
|  | Month 12 | SP | 1 | -^§^ | -^§^ | - | - | - | - |
|  |  | FMT | 10 | -4·96 | 1·97 | -^§^ | -^§^ | -^§^ | -^§^ |
| CD8+CD4+ (% over CD3) | Month 6 | SP | 10 | -1·80 | 1·00 | - | - | - | - |
|  |  | FMT | 14 | -1·18 | 0·84 | 0·62 | -1·92 | 3·14 | 0·64 |
|  | Month 12 | SP | 1 | -^§^ | -^§^ | - | - | - | - |
|  |  | FMT | 6 | 2·74 | 2·39 | -^§^ | -^§^ | -^§^ | -^§^ |
| CD4+  (% over CD3) | Month 6 | SP | 10 | -7·53 | 8·01 | - | - | - | - |
|  |  | FMT | 19 | 7·50 | 5·80 | 15·03 | -4·33 | 34·26 | 0·14 |
|  | Month 12 | SP | 1 | -^§^ | -^§^ | - | - | - | - |
|  |  | FMT | 10 | -17·36 | 10·29 | -^§^ | -^§^ | -^§^ | -^§^ |
| Th0  (% over CD4+) | Month 6 | SP | 10 | -2·25 | 5·76 | - | - | - | - |
|  |  | FMT | 19 | -1·74 | 4·13 | 0·51 | -13·16 | 14·19 | 0·94 |
|  | Month 12 | SP | 1 | -^§^ | -^§^ | - | - | - | - |
|  |  | FMT | 9 | 16·31 | 6·92 | -^§^ | -^§^ | -^§^ | -^§^ |
| CxCR3+  (% over CD4+) | Month 6 | SP | 10 | 2·16 | 3·29 | - | - | - | - |
|  |  | FMT | 19 | -1·31 | 2·37 | -3·47 | -11·32 | 4·48 | 0·40 |
|  | Month 12 | SP | 1 | -^§^ | -^§^ | - | - | - | - |
|  |  | FMT | 9 | 0·61 | 4·06 | -^§^ | -^§^ | -^§^ | -^§^ |
| Th1/Th17 | Month 6 | SP | 10 | -0·07 | 0·13 | - | - | - | - |
|  |  | FMT | 19 | -0·08 | 0·09 | -0·01 | -0·32 | 0·31 | 0·96 |
|  | Month 12 | SP | 1 | -^§^ | -^§^ | - | - | - | - |
|  |  | FMT | 9 | -0·22 | 0·09 | -^§^ | -^§^ | -^§^ | -^§^ |
| Th1  (% over CD4+) | Month 6 | SP | 10 | 1·37 | 1·91 | - | - | - | - |
|  |  | FMT | 19 | -0·25 | 1·37 | -1·62 | -6·14 | 2·91 | 0·49 |
|  | Month 12 | SP | 1 | -^§^ | -^§^ | - | - | - | - |
|  |  | FMT | 9 | -1·87 | 1·52 | -^§^ | -^§^ | -^§^ | -^§^ |
| CCR4+  (% over CD4+) | Month 6 | SP | 10 | 6·03 | 9·10 | - | - | - | - |
|  |  | FMT | 19 | 9·77 | 6·58 | 3·74 | -18·36 | 25·51 | 0·74 |
|  | Month 12 | SP | 1 | -^§^ | -^§^ | - | - | - | - |
|  |  | FMT | 9 | 0·26 | 10·11 | -^§^ | -^§^ | -^§^ | -^§^ |
| Th17  (% over CD4+) | Month 6 | SP | 10 | 6·10 | 4·65 | - | - | - | - |
|  |  | FMT | 19 | -1·49 | 3·37 | -7·59 | -19·62 | 3·58 | 0·20 |
|  | Month 12 | SP | 1 | -^§^ | -^§^ | - | - | - | - |
|  |  | FMT | 9 | -1·12 | 3·71 | -^§^ | -^§^ | -^§^ | -^§^ |
| Th2  (% over CD4+) | Month 6 | SP | 10 | 0·38 | 9·15 | - | - | - | - |
|  |  | FMT | 19 | 12·20 | 6·59 | 11·82 | -10·17 | 33·78 | 0·30 |
|  | Month 12 | SP | 1 | -^§^ | -^§^ | - | - | - | - |
|  |  | FMT | 9 | 4·62 | 9·31 | -^§^ | -^§^ | -^§^ | -^§^ |
| Th9  (% over CD4+) | Month 6 | SP | 10 | -0·39 | 0·70 | - | - | - | - |
|  |  | FMT | 19 | -1·14 | 0·51 | -0·75 | -2·42 | 0·92 | 0·39 |
|  | Month 12 | SP | 1 | -^§^ | -^§^ | - | - | - | - |
|  |  | FMT | 9 | -0·99 | 0·68 | -^§^ | -^§^ | -^§^ | -^§^ |
| CCR6+ CCR4+  (% over CD4+) | Month 6 | SP | 10 | 5·91 | 7·10 | - | - | - | - |
|  |  | FMT | 19 | -4·28 | 5·14 | -10·19 | -27·08 | 6·70 | 0·25 |
|  | Month 12 | SP | 1 | -^§^ | -^§^ | - | - | - | - |
|  |  | FMT | 9 | 2·32 | 6·31 | -^§^ | -^§^ | -^§^ | -^§^ |
| Th22  (% over CD4+) | Month 6 | SP | 10 | -0·16 | 0·26 | - | - | - | - |
|  |  | FMT | 19 | -0·31 | 0·19 | -0·15 | -0·80 | 0·48 | 0·64 |
|  | Month 12 | SP | 1 | -^§^ | -^§^ | - | - | - | - |
|  |  | FMT | 9 | -0·33 | 0·33 | -^§^ | -^§^ | -^§^ | -^§^ |
| CD8+  (% over CD3) | Month 6 | SP | 10 | 4·32 | 5·91 | - | - | - | - |
|  |  | FMT | 19 | -7·96 | 4·31 | -12·28 | -26·46 | 2·13 | 0·10 |
|  | Month 12 | SP | 1 | -^§^ | -^§^ | - | - | - | - |
|  |  | FMT | 10 | -16·59 | 6·47 | -^§^ | -^§^ | -^§^ | -^§^ |
| Tc0  (% over CD8+) | Month 6 | SP | 10 | -5·59 | 6·07 | - | - | - | - |
|  |  | FMT | 18 | 3·09 | 4·46 | 8·67 | -5·86 | 23·21 | 0·25 |
|  | Month 12 | SP | 1 | -^§^ | -^§^ | - | - | - | - |
|  |  | FMT | 7 | 4·34 | 4·69 | -^§^ | -^§^ | -^§^ | -^§^ |
| CXCR3  (% over CD8+) | Month 6 | SP | 10 | 1·79 | 2·23 | - | - | - | - |
|  |  | FMT | 18 | -1·77 | 1·65 | -3·56 | -8·92 | 1·92 | 0·21 |
|  | Month 12 | SP | 1 | -^§^ | -^§^ | - | - | - | - |
|  |  | FMT | 7 | -2·04 | 2·79 | -^§^ | -^§^ | -^§^ | -^§^ |
| Tc1/Tc17 | Month 6 | SP | 10 | -0·04 | 0·16 | - | - | - | - |
|  |  | FMT | 18 | -0·10 | 0·12 | -0·07 | -0·44 | 0·31 | 0·74 |
|  | Month 12 | SP | 1 | -^§^ | -^§^ | - | - | - | - |
|  |  | FMT | 7 | -0·04 | 0·02 | -^§^ | -^§^ | -^§^ | -^§^ |
| Tc1  (% over CD8+) | Month 6 | SP | 10 | 1·22 | 0·92 | - | - | - | - |
|  |  | FMT | 18 | -0·64 | 0·68 | -1·86 | -4·07 | 0·35 | 0·11 |
|  | Month 12 | SP | 1 | -^§^ | -^§^ | - | - | - | - |
|  |  | FMT | 7 | -0·68 | 0·71 | -^§^ | -^§^ | -^§^ | -^§^ |
| CCR4+  (% over CD8+) | Month 6 | SP | 10 | 17·12 | 10·04 | - | - | - | - |
|  |  | FMT | 18 | 11·59 | 7·48 | -5·53 | -30·20 | 18·73 | 0·66 |
|  | Month 12 | SP | 1 | -^§^ | -^§^ | - | - | - | - |
|  |  | FMT | 7 | 1·72 | 12·23 | -^§^ | -^§^ | -^§^ | -^§^ |
| Tc17  (% over CD8+) | Month 6 | SP | 10 | 12·56 | 8·09 | - | - | - | - |
|  |  | FMT | 18 | 2·81 | 6·00 | -9·76 | -29·43 | 9·98 | 0·34 |
|  | Month 12 | SP | 1 | -^§^ | -^§^ | - | - | - | - |
|  |  | FMT | 7 | 0·68 | 4·36 | -^§^ | -^§^ | -^§^ | -^§^ |
| Tc2  (% over CD8+) | Month 6 | SP | 10 | 5·19 | 9·51 | - | - | - | - |
|  |  | FMT | 18 | 4·92 | 7·06 | -0·27 | -23·72 | 22·62 | 0·98 |
|  | Month 12 | SP | 1 | -^§^ | -^§^ | - | - | - | - |
|  |  | FMT | 7 | 6·35 | 10·80 | -^§^ | -^§^ | -^§^ | -^§^ |
| Tc9  (% over CD8+) | Month 6 | SP | 10 | -2·67 | 2·43 | - | - | - | - |
|  |  | FMT | 18 | -2·60 | 1·78 | 0·07 | -5·73 | 5·87 | 0·98 |
|  | Month 12 | SP | 1 | -^§^ | -^§^ | - | - | - | - |
|  |  | FMT | 7 | -5·14 | 1·81 | -^§^ | -^§^ | -^§^ | -^§^ |
| CCR4+ CCR6+  (% over CD8+) | Month 6 | SP | 10 | 5·33 | 8·94 | - | - | - | - |
|  |  | FMT | 18 | 8·09 | 6·68 | 2·76 | -19·00 | 24·65 | 0·81 |
|  | Month 12 | SP | 1 | -^§^ | -^§^ | - | - | - | - |
|  |  | FMT | 7 | 0·36 | 6·10 | -^§^ | -^§^ | -^§^ | -^§^ |
| Tc22  (% over CD8+) | Month 6 | SP | 10 | 0·14 | 0·94 | - | - | - | - |
|  |  | FMT | 18 | 1·28 | 0·69 | 1·14 | -1·11 | 3·39 | 0·33 |
|  | Month 12 | SP | 1 | -^§^ | -^§^ | - | - | - | - |
|  |  | FMT | 7 | 0·23 | 0·51 | -^§^ | -^§^ | -^§^ | -^§^ |

# Mean absolute changes from baseline to month 6 and 12 are showed for each treatment group and comparison were performed using linear mixed-effects regression models that include indicator variables for treatment arms, time and the interaction time x arm as independent variables. T tests were used to test the null hypothesis that regression coefficients were equal to 0 and obtained p-values reported. Satterthwaite approximation was used to estimate degrees of freedom. Given the exploratory nature of these analyses, no adjustment for multiple comparisons was performed and nominal p-values were reported

# Abbreviations: N: number of samples for analysis; MD = Mean Difference; se = Standard Error; CI = Confidence Interval; SP: Sham Procedure; FMT: Faecal Microbiota Transplantation.

^§^ these values couldn’t be calculated as only one observation per SP arm was available.

#### Supplementary Table 23. Mean variations of intestinal immune cells subpopulations at baseline and over time (from baseline to month 6), in FMT and SP arms, and from the comparison of the treatment arms over time. Intention-to-treat analysis.

| **Immune cells populations** |  | **Estimate** | **Std·**  **Error** | **95% Conf· Int·** | | **P Value** |
| --- | --- | --- | --- | --- | --- | --- |
| FOXP3/b2M expression | Mean difference between FMT and SP | -0·41 | 1·38 | -3·07 | 2·25 | 0·77 |
|  | Monthly variation SP | -0·02 | 0·21 | -0·44 | 0·39 | 0·91 |
|  | Monthly variation FMT | 0·18 | 0·14 | -0·09 | 0·45 | 0·20 |
|  | Mean difference in monthly variation | 0·20 | 0·25 | -0·29 | 0·70 | 0·43 |
| CD3+ (% CD45+) | Mean difference between FMT and SP | -1·35 | 6·81 | -14·52 | 11·81 | 0·84 |
|  | Monthly variation SP | 1·40 | 1·16 | -0·85 | 3·66 | 0·23 |
|  | Monthly variation FMT | 2·01 | 0·84 | 0·37 | 3·64 | 0·022 |
|  | Mean difference in monthly variation | 0·61 | 1·43 | -2·19 | 3·39 | 0·67 |
| CD4+ (% CD3+) | Mean difference between FMT and SP | -4·29 | 7·37 | -18·52 | 9·92 | 0·56 |
|  | Monthly variation SP | -1·25 | 1·33 | -3·87 | 1·34 | 0·35 |
|  | Monthly variation FMT | 1·25 | 0·97 | -0·65 | 3·12 | 0·20 |
|  | Mean difference in monthly variation | 2·50 | 1·65 | -0·72 | 5·71 | 0·14 |
| Th0 (% CD4+) | Mean difference between FMT and SP | 0·22 | 4·65 | -8·75 | 9·20 | 0·96 |
|  | Monthly variation SP | -0·38 | 0·96 | -2·23 | 1·48 | 0·70 |
|  | Monthly variation FMT | -0·29 | 0·69 | -1·62 | 1·04 | 0·67 |
|  | Mean difference in monthly variation | 0·09 | 1·18 | -2·19 | 2·36 | 0·94 |
| CxCR3+ (% CD4+) | Mean difference between FMT and SP | -0·44 | 2·83 | -5·89 | 5·03 | 0·88 |
|  | Monthly variation SP | 0·36 | 0·55 | -0·7 | 1·43 | 0·51 |
|  | Monthly variation FMT | -0·22 | 0·40 | -0·98 | 0·57 | 0·58 |
|  | Mean difference in monthly variation | -0·58 | 0·68 | -1·89 | 0·75 | 0·40 |
| Th1/Th17 (% CD4+) | Mean difference between FMT and SP | 0·00 | 0·11 | -0·21 | 0·21 | 0·99 |
|  | Monthly variation SP | -0·01 | 0·02 | -0·05 | 0·03 | 0·57 |
|  | Monthly variation FMT | -0·01 | 0·02 | -0·04 | 0·02 | 0·39 |
|  | Mean difference in monthly variation | 0·00 | 0·03 | -0·05 | 0·05 | 0·96 |
| Th1 (% CD4+) | Mean difference between FMT and SP | -0·46 | 1·54 | -3·43 | 2·51 | 0·77 |
|  | Monthly variation SP | 0·23 | 0·32 | -0·39 | 0·84 | 0·48 |
|  | Monthly variation FMT | -0·04 | 0·23 | -0·48 | 0·40 | 0·86 |
|  | Mean difference in monthly variation | -0·27 | 0·39 | -1·02 | 0·48 | 0·49 |
| CCR4+ (% CD4+) | Mean difference between FMT and SP | 2·59 | 8·15 | -13·16 | 18·32 | 0·75 |
|  | Monthly variation SP | 1·01 | 1·52 | -1·95 | 3·98 | 0·51 |
|  | Monthly variation FMT | 1·63 | 1·10 | -0·53 | 3·75 | 0·15 |
|  | Mean difference in monthly variation | 0·62 | 1·87 | -3·06 | 4·25 | 0·74 |
| Th17 (% CD4+) | Mean difference between FMT and SP | 3·73 | 4·22 | -4·43 | 11·88 | 0·38 |
|  | Monthly variation SP | 1·02 | 0·78 | -0·54 | 2·57 | 0·21 |
|  | Monthly variation FMT | -0·25 | 0·56 | -1·46 | 0·83 | 0·66 |
|  | Mean difference in monthly variation | -1·27 | 0·96 | -3·27 | 0·60 | 0·20 |
| Th2 (% CD4+) | Mean difference between FMT and SP | -1·17 | 7·84 | -16·3 | 13·94 | 0·88 |
|  | Monthly variation SP | 0·06 | 1·52 | -2·9 | 3·05 | 0·97 |
|  | Monthly variation FMT | 2·03 | 1·10 | -0·10 | 4·18 | 0·072 |
|  | Mean difference in monthly variation | 1·97 | 1·88 | -1·69 | 5·63 | 0·30 |
| Th9 (% CD4+) | Mean difference between FMT and SP | 0·38 | 0·57 | -0·73 | 1·48 | 0·51 |
|  | Monthly variation SP | -0·06 | 0·12 | -0·29 | 0·16 | 0·58 |
|  | Monthly variation FMT | -0·19 | 0·08 | -0·35 | -0·03 | 0·029 |
|  | Mean difference in monthly variation | -0·12 | 0·14 | -0·40 | 0·15 | 0·39 |
| CCR6+ CCR4+ (% CD4+) | Mean difference between FMT and SP | 4·39 | 5·77 | -6·74 | 15·52 | 0·45 |
|  | Monthly variation SP | 0·99 | 1·18 | -1·30 | 3·27 | 0·41 |
|  | Monthly variation FMT | -0·71 | 0·86 | -2·36 | 0·94 | 0·41 |
|  | Mean difference in monthly variation | -1·70 | 1·46 | -4·51 | 1·12 | 0·25 |
| Th22 (% CD4+) | Mean difference between FMT and SP | 0·22 | 0·22 | -0·22 | 0·65 | 0·34 |
|  | Monthly variation SP | -0·03 | 0·04 | -0·11 | 0·06 | 0·55 |
|  | Monthly variation FMT | -0·05 | 0·03 | -0·12 | 0·01 | 0·12 |
|  | Mean difference in monthly variation | -0·03 | 0·05 | -0·13 | 0·08 | 0·64 |
| CD8+ (% CD3) | Mean difference between FMT and SP | 5·62 | 6·53 | -7·01 | 18·29 | 0·39 |
|  | Monthly variation SP | 0·72 | 0·98 | -1·20 | 2·64 | 0·47 |
|  | Monthly variation FMT | -1·33 | 0·72 | -2·72 | 0·10 | 0·074 |
|  | Mean difference in monthly variation | -2·05 | 1·22 | -4·41 | 0·35 | 0·10 |
| Tc0 (% CD8+) | Mean difference between FMT and SP | -2·42 | 4·94 | -11·94 | 7·10 | 0·63 |
|  | Monthly variation SP | -0·93 | 1·01 | -2·88 | 1·02 | 0·36 |
|  | Monthly variation FMT | 0·51 | 0·74 | -0·92 | 1·95 | 0·49 |
|  | Mean difference in monthly variation | 1·45 | 1·26 | -0·98 | 3·87 | 0·25 |
| CXCR3 % CD8+ | Mean difference between FMT and SP | 1·02 | 1·86 | -2·57 | 4·61 | 0·59 |
|  | Monthly variation SP | 0·30 | 0·37 | -0·43 | 1·02 | 0·43 |
|  | Monthly variation FMT | -0·29 | 0·27 | -0·82 | 0·25 | 0·29 |

|  | Mean difference in monthly variation | -0·59 | 0·46 | -1·49 | 0·32 | 0·21 |
| --- | --- | --- | --- | --- | --- | --- |
| Tc1/Tc17 (% CD8+) | Mean difference between FMT and SP | 0·10 | 0·13 | -0·15 | 0·34 | 0·45 |
|  | Monthly variation SP | -0·01 | 0·03 | -0·06 | 0·04 | 0·81 |
|  | Monthly variation FMT | -0·02 | 0·02 | -0·05 | 0·02 | 0·37 |
|  | Mean difference in monthly variation | -0·01 | 0·03 | -0·07 | 0·05 | 0·74 |
| Tc1 (% CD8+) | Mean difference between FMT and SP | 0·07 | 0·75 | -1·38 | 1·52 | 0·92 |
|  | Monthly variation SP | 0·20 | 0·15 | -0·09 | 0·50 | 0·19 |
|  | Monthly variation FMT | -0·11 | 0·11 | -0·33 | 0·11 | 0·35 |
|  | Mean difference in monthly variation | -0·31 | 0·19 | -0·68 | 0·06 | 0·11 |
| CCR4+ (% CD8+) | Mean difference between FMT and SP | 5·71 | 9·01 | -11·68 | 23·1 | 0·53 |
|  | Monthly variation SP | 2·85 | 1·67 | -0·40 | 6·15 | 0·10 |
|  | Monthly variation FMT | 1·93 | 1·25 | -0·51 | 4·35 | 0·13 |
|  | Mean difference in monthly variation | -0·92 | 2·09 | -5·03 | 3·12 | 0·66 |
| Tc17 (% CD8+) | Mean difference between FMT and SP | 6·81 | 7·00 | -6·69 | 20·32 | 0·33 |
|  | Monthly variation SP | 2·09 | 1·35 | -0·55 | 4·73 | 0·13 |
|  | Monthly variation FMT | 0·47 | 1·00 | -1·48 | 2·42 | 0·64 |
|  | Mean difference in monthly variation | -1·63 | 1·68 | -4·90 | 1·66 | 0·34 |
| Tc2 (% CD8+) | Mean difference between FMT and SP | -0·58 | 8·32 | -16·64 | 15·47 | 0·94 |
|  | Monthly variation SP | 0·87 | 1·58 | -2·21 | 4·00 | 0·59 |
|  | Monthly variation FMT | 0·82 | 1·18 | -1·49 | 3·10 | 0·49 |
|  | Mean difference in monthly variation | -0·05 | 1·97 | -3·95 | 3·77 | 0·98 |
| Tc9 (% CD8+) | Mean difference between FMT and SP | 0·11 | 1·97 | -3·69 | 3·92 | 0·95 |
|  | Monthly variation SP | -0·44 | 0·40 | -1·22 | 0·33 | 0·27 |
|  | Monthly variation FMT | -0·43 | 0·30 | -1·01 | 0·14 | 0·15 |
|  | Mean difference in monthly variation | 0·01 | 0·50 | -0·96 | 0·98 | 0·98 |
| CCR4+ CCR6+ (% CD8+) | Mean difference between FMT and SP | 5·13 | 8·32 | -10·92 | 21·22 | 0·54 |
|  | Monthly variation SP | 0·89 | 1·49 | -2·05 | 3·79 | 0·56 |
|  | Monthly variation FMT | 1·35 | 1·11 | -0·83 | 3·52 | 0·23 |
|  | Mean difference in monthly variation | 0·46 | 1·86 | -3·17 | 4·11 | 0·81 |
| Tc22 (% CD8+) | Mean difference between FMT and SP | 0·03 | 0·77 | -1·45 | 1·51 | 0·97 |
|  | Monthly variation SP | 0·02 | 0·16 | -0·28 | 0·33 | 0·89 |
|  | Monthly variation FMT | 0·21 | 0·12 | -0·01 | 0·43 | 0·070 |
|  | Mean difference in monthly variation | 0·19 | 0·19 | -0·19 | 0·57 | 0·33 |

Monthly variations and differences between monthly variations in each intestinal immune cells subpopulation were estimated by linear mixed-effects models with random intercept. These models included as covariates arm, time and the interaction arm x time. Monthly variations were computed between baseline and month 6, as measurements at month 12 were too scarce. Mean differences between FMT and sham procedure patients are displayed at baseline, then monthly variations in SP and FMT-patients are showed, and finally the mean difference in the monthly variation between the two arms. T tests were used to test the null hypothesis that regression coefficients were equal to 0 and obtained p-values reported. Satterthwaite approximation was used to estimate degrees of freedom. Abbreviations: Conf. Int.: confidence interval; FMT: faecal microbiota transplantation; SP: sham procedure; Std. Error: standard error; Th: T-helper; Tc: T cytotoxic.

#### Supplementary Table 24. Changes from baseline to month 6 and 12 of serum cytokines across treatment arms. Intention to treat analysis.

| **Serum cytokines Time points** | | **Arm** | **N** | **Mean ratio** | **SE** | **MD** | **change from baseline**  **95% CI P value** | | |
| --- | --- | --- | --- | --- | --- | --- | --- | --- | --- |
| Month 6 | | SP | 10 | 1·54 | 0·55 | - | - | - - | |
| IL-1β |  | FMT | 23 | 1·30 | 0·3 | 0·85 | 0·39 | 1·84 | 0·67 |
|  | Month 12 | SP | 6 | 3·39 | 1·43 | - | - | - | - |
|  |  | FMT | 16 | 1·64 | 0·41 | 0·48 | 0·2 | 1·16 | 0·11 |
|  | Month 6 | SP | 10 | 1·29 | 0·3 | - | - | - | - |
| IL-2 |  | FMT | 23 | 1·37 | 0·21 | 1·07 | 0·63 | 1·80 | 0·80 |

-§	-§

| -  -§ | -  -§ | -  -§ | -  -§ |
| --- | --- | --- | --- |
| - | - | - | - |

| Month 12 | SP | 6 |
| --- | --- | --- |
|  | FMT | 16 |
| Month 6 | SP | 10 |

-§	-§

1·00	0·16

| IL-4 |  | FMT | 23 | 1·15 | 0·12 | 1·15 | 0·80 | 1·66 | 0·45 |
| --- | --- | --- | --- | --- | --- | --- | --- | --- | --- |
|  | Month 12 | SP | 6 | 1·45 | 0·26 | - | - | - | - |
|  |  | FMT | 16 | 1·22 | 0·14 | 0·84 | 0·56 | 1·25 | 0·40 |
|  | Month 6 | SP | 10 | 0·80 | 0·44 | - | - | - | - |
| IL-5 |  | FMT | 23 | 1·26 | 0·43 | 1·57 | 0·52 | 4·70 | 0·42 |
|  | Month 12 | SP | 6 | 1·75 | 1·19 | - | - | - | - |
|  |  | FMT | 16 | 2·24 | 0·84 | 1·28 | 0·36 | 4·49 | 0·70 |
|  | Month 6 | SP | 10 | 2·14 | 1·15 | - | - | - | - |
| IL-6 |  | FMT | 23 | 1·80 | 0·61 | 0·84 | 0·28 | 2·51 | 0·76 |
|  | Month 12 | SP | 6 | 0·69 | 0·42 | - | - | - | - |
|  |  | FMT | 16 | 1·20 | 0·41 | 1·73 | 0·53 | 5·6 | 0·37 |
|  | Month 6 | SP | 10 | 1·96 | 2·29 | - | - | - | - |
| IL-7 |  | FMT | 22 | 1·35 | 0·87 | 0·69 | 0·11 | 4·17 | 0·68 |
|  | Month 12 | SP | 6 | 0·46 | 0·32 | - | - | - | - |
|  |  | FMT | 16 | 0·86 | 0·32 | 1·86 | 0·52 | 6·61 | 0·35 |
|  | Month 6 | SP | 10 | 3·57 | 3·05 | - | - | - | - |
| IL-8 |  | FMT | 23 | 1·66 | 0·82 | 0·46 | 0·10 | 2·07 | 0·32 |
|  | Month 12 | SP | 6 | 0·84 | 1·05 | - | - | - | - |
|  |  | FMT | 16 | 2·52 | 1·53 | 3·00 | 0·48 | 18·7 | 0·25 |
|  | Month 6 | SP | 10 | 0·82 | 0·46 | - | - | - | - |
| IL-10 |  | FMT | 22 | 1·96 | 0·70 | 2·38 | 0·77 | 7·34 | 0·13 |
|  | Month 12 | SP | 6 | 1·41 | 0·94 | - | - | - | - |
|  |  | FMT | 15 | 2·53 | 0·97 | 1·79 | 0·51 | 6·32 | 0·37 |
|  | Month 6 | SP | 10 | 0·79 | 0·63 | - | - | - | - |
| IL-12p70 |  | FMT | 23 | 1·41 | 0·66 | 1·79 | 0·43 | 7·53 | 0·43 |
|  | Month 12 | SP | 6 | 1·14 | 1·08 | - | - | - | - |
|  |  | FMT | 16 | 0·87 | 0·42 | 0·76 | 0·16 | 3·61 | 0·73 |
|  | Month 6 | SP | 10 | 1·29 | 0·56 | - | - | - | - |
| IL-13 |  | FMT | 23 | 1·29 | 0·36 | 1·01 | 0·41 | 2·50 | 0·99 |
|  | Month 12 | SP | 6 | 2·71 | 0·94 | - | - | - | - |
|  |  | FMT | 16 | 1·42 | 0·30 | 0·53 | 0·25 | 1·11 | 0·10 |
|  | Month 6 | SP | 10 | 1·45 | 0·64 | - | - | - | - |
| IL-17 |  | FMT | 22 | 1·00 | 0·29 | 0·69 | 0·27 | 1·75 | 0·43 |
|  | Month 12 | SP | 6 | 2·68 | 1·41 | - | - | - | - |
|  |  | FMT | 16 | 1·29 | 0·39 | 0·48 | 0·17 | 1·36 | 0·18 |
|  | Month 6 | SP | 10 | 0·74 | 0·57 | - | - | - | - |
| G-CSF |  | FMT | 23 | 1·33 | 0·61 | 1·79 | 0·44 | 7·32 | 0·42 |
|  | Month 12 | SP | 6 | 3·07 | 2·31 | - | - | - | - |
|  |  | FMT | 16 | 1·72 | 0·73 | 0·56 | 0·14 | 2·19 | 0·41 |
|  | Month 6 | SP | 10 | 1·00 | 0·20 | - | - | - | - |
| GM-CSF |  | FMT | 23 | 1·03 | 0·14 | 1·03 | 0·66 | 1·61 | 0·88 |
|  | Month 12 | SP | 6 | 1·00 | 0·25 | - | - | - | - |
|  |  | FMT | 16 | 1·09 | 0·16 | 1·09 | 0·63 | 1·89 | 0·76 |
|  | Month 6 | SP | 10 | 1·88 | 0·76 | - | - | - | - |
| IFN-γ |  | FMT | 23 | 0·96 | 0·25 | 0·51 | 0·22 | 1·21 | 0·13 |
|  | Month 12 | SP | 6 | 0·76 | 0·35 | - | - | - | - |
|  |  | FMT | 16 | 0·88 | 0·24 | 1·15 | 0·45 | 2·97 | 0·77 |
|  | Month 6 | SP | 10 | 1·41 | 0·52 | - | - | - | - |
| MCP-1 |  | FMT | 23 | 0·96 | 0·22 | 0·68 | 0·31 | 1·51 | 0·35 |
|  | Month 12 | SP | 6 | 0·53 | 0·22 | - | - | - | - |
|  |  | FMT | 16 | 0·89 | 0·22 | 1·67 | 0·70 | 4·02 | 0·25 |
|  | Month 6 | SP | 10 | 2·18 | 1·30 | - | - | - | - |
| MIP-1α |  | FMT | 23 | 1·17 | 0·43 | 0·54 | 0·17 | 1·74 | 0·30 |
|  | Month 12 | SP | 6 | 0·76 | 0·42 | - | - | - | - |
|  |  | FMT | 16 | 1·15 | 0·37 | 1·51 | 0·50 | 4·53 | 0·47 |
|  | Month 6 | SP | 10 | 2·01 | 0·90 | - | - | - | - |
| MIP-1β |  | FMT | 23 | 1·13 | 0·32 | 0·56 | 0·22 | 1·45 | 0·23 |
|  | Month 12 | SP | 6 | 0·23 | 0·12 | - | - | - | - |
|  |  | FMT | 16 | 0·93 | 0·28 | 4·08 | 1·44 | 11·56 | 0·012 |

| **Serum cytokines Time points** | | **Arm** | **N** | **Mean ratio** | **SE** | **MD** | **change from baseline**  **95% CI P value** | | |
| --- | --- | --- | --- | --- | --- | --- | --- | --- | --- |
| Month 6 | | SP | 10 | 1·68 | 1·99 | - | - | - - | |
| TNFalpha |  | FMT | 23 | 1·23 | 0·77 | 0·73 | 0·12 | 4·44 | 0·74 |
|  | Month 12 | SP | 6 | 0·46 | 0·54 | - | - | - | - |
|  |  | FMT | 16 | 0·95 | 0·54 | 2·06 | 0·36 | 11·8 | 0·42 |
|  | Month 6 | SP | 10 | 1·00 | 0·36 | - | - | - | - |
| VEGF |  | FMT | 23 | 1·30 | 0·30 | 1·30 | 0·59 | 2·85 | 0·52 |
|  | Month 12 | SP | 6 | 1·93 | 0·46 | - | - | - | - |
|  |  | FMT | 16 | 1·00 | 0·15 | 0·52 | 0·30 | 0·88 | 0·018 |

Mean absolute changes from baseline to month 6 are showed for each treatment group and comparison were performed using linear mixed-effects regression models that include indicator variables for treatment arms. time and the interaction time x arm as independent variables. For these analyses, laboratory parameters were scaled using a logarithmic transformation, to handle their strongly left-skewed distributions. The term "mean ratio" indicates the ratio of the geometric mean at 6 months and the geometric mean at baseline. T tests were used to test the null hypothesis that regression coefficients were equal to 0 and obtained p-values reported. Satterthwaite approximation was used to estimate degrees of freedom.

Given the exploratory nature of these analyses, no adjustment for multiple comparisons was performed and nominal p-values were reported Abbreviations: N: number of samples for analysis; MD = Mean Difference (ratio between mean ratio of FMT arm and SP arm); se = Standard Error; CI

= Confidence Interval; SP: Sham Procedure; FMT: Faecal Microbiota Transplantation.

^§^ these values couldn’t be calculated as concentrations were under the lower limit of quantification (LLOQ) of the assay.

#### Supplementary Table 25. Average monthly changes of serum cytokines in the SP and the FMT arms, and their ratio over time (estimated from baseline to month 12). Intention-to-treat analysis.

|  |  | **Estimate** | **Std· Error** | **95% Conf· Int·** | | **P Value** |
| --- | --- | --- | --- | --- | --- | --- |
| IL-1β | Mean ratio between FMT and SP | 1·3 | 1·29 | 0·79 | 2·16 | 0·30 |
|  | Monthly variation in SP arm (mean ratio) | 1·08 | 1·03 | 1·02 | 1·14 | 0·010 |
|  | Monthly variation in FMT arm (mean ratio) | 1·03 | 1·02 | 0·99 | 1·06 | 0·17 |
|  | Mean ratio of monthly variation between FMT and SP | 0·95 | 1·03 | 0·89 | 1·02 | 0·16 |
| IL-2 | Mean ratio between FMT and SP | 0·96 | 1·15 | 0·72 | 1·26 | 0·75 |
|  | Monthly variation in SP arm (mean ratio) | 1·00 | 1·02 | 0·96 | 1·03 | 0·86 |
|  | Monthly variation in FMT arm (mean ratio) | 1·00 | 1·01 | 0·98 | 1·03 | 0·87 |
|  | Mean ratio of monthly variation between FMT and SP | 1·01 | 1·02 | 0·96 | 1·05 | 0·81 |
| IL-4 | Mean ratio between FMT and SP | 1·11 | 1·11 | 0·91 | 1·36 | 0·30 |
|  | Monthly variation in SP arm (mean ratio) | 1·03 | 1·01 | 1·01 | 1·06 | 0·010 |
|  | Monthly variation in FMT arm (mean ratio) | 1·01 | 1·01 | 0·99 | 1·03 | 0·20 |
|  | Mean ratio of monthly variation between FMT and SP | 0·98 | 1·02 | 0·95 | 1·01 | 0·15 |
| IL-5 | Mean ratio between FMT and SP | 0·88 | 1·38 | 0·47 | 1·67 | 0·70 |
|  | Monthly variation in SP arm (mean ratio) | 1·00 | 1·04 | 0·93 | 1·09 | 0·91 |
|  | Monthly variation in FMT arm (mean ratio) | 1·04 | 1·03 | 0·99 | 1·09 | 0·16 |
|  | Mean ratio of monthly variation between FMT and SP | 1·03 | 1·05 | 0·94 | 1·14 | 0·49 |
| IL-6 | Mean ratio between FMT and SP | 0·79 | 1·43 | 0·39 | 1·59 | 0·50 |
|  | Monthly variation in SP arm (mean ratio) | 0·99 | 1·05 | 0·90 | 1·08 | 0·83 |
|  | Monthly variation in FMT arm (mean ratio) | 1·03 | 1·03 | 0·97 | 1·09 | 0·38 |
|  | Mean ratio of monthly variation between FMT and SP | 1·04 | 1·06 | 0·93 | 1·16 | 0·51 |
| IL-7 | Mean ratio between FMT and SP | 0·59 | 1·81 | 0·19 | 1·90 | 0·38 |
|  | Monthly variation in SP arm (mean ratio) | 0·95 | 1·07 | 0·83 | 1·08 | 0·45 |
|  | Monthly variation in FMT arm (mean ratio) | 0·99 | 1·05 | 0·91 | 1·08 | 0·85 |
|  | Mean ratio of monthly variation between FMT and SP | 1·04 | 1·08 | 0·89 | 1·22 | 0·61 |
| IL-8 | Mean ratio between FMT and SP | 0·83 | 1·58 | 0·34 | 2·02 | 0·70 |
|  | Monthly variation in SP arm (mean ratio) | 1·02 | 1·06 | 0·90 | 1·14 | 0·78 |
|  | Monthly variation in FMT arm (mean ratio) | 1·05 | 1·04 | 0·97 | 1·14 | 0·20 |
|  | Mean ratio of monthly variation between FMT and SP | 1·03 | 1·07 | 0·90 | 1·19 | 0·64 |
| IL-10 | Mean ratio between FMT and SP | 0·94 | 1·43 | 0·47 | 1·89 | 0·87 |
|  | Monthly variation in SP arm (mean ratio) | 1·02 | 1·04 | 0·95 | 1·11 | 0·54 |
|  | Monthly variation in FMT arm (mean ratio) | 1·07 | 1·03 | 1·01 | 1·13 | 0·020 |
|  | Mean ratio of monthly variation between FMT and SP | 1·04 | 1·05 | 0·95 | 1·15 | 0·41 |
| IL-12p70 | Mean ratio between FMT and SP | 1·18 | 1·75 | 0·40 | 3·53 | 0·77 |
|  | Monthly variation in SP arm (mean ratio) | 1·02 | 1·05 | 0·92 | 1·13 | 0·69 |
|  | Monthly variation in FMT arm (mean ratio) | 0·99 | 1·04 | 0·92 | 1·06 | 0·77 |
|  | Mean ratio of monthly variation between FMT and SP | 0·97 | 1·07 | 0·85 | 1·1 | 0·62 |
| IL-13 | Mean ratio between FMT and SP | 1·22 | 1·31 | 0·72 | 2·07 | 0·47 |
|  | Monthly variation in SP arm (mean ratio) | 1·08 | 1·03 | 1·01 | 1·15 | 0·019 |
|  | Monthly variation in FMT arm (mean ratio) | 1·03 | 1·02 | 0·99 | 1·08 | 0·16 |
|  | Mean ratio of monthly variation between FMT and SP | 0·95 | 1·04 | 0·88 | 1·03 | 0·23 |
| IL-17 | Mean ratio between FMT and SP | 1·09 | 1·33 | 0·63 | 1·89 | 0·76 |
|  | Monthly variation in SP arm (mean ratio) | 1·07 | 1·03 | 1·01 | 1·15 | 0·034 |
|  | Monthly variation in FMT arm (mean ratio) | 1·02 | 1·02 | 0·98 | 1·07 | 0·40 |
|  | Mean ratio of monthly variation between FMT and SP | 0·95 | 1·04 | 0·88 | 1·03 | 0·20 |
| G-CSF | Mean ratio between FMT and SP | 1·02 | 1·52 | 0·45 | 2·33 | 0·96 |
|  | Monthly variation in SP arm (mean ratio) | 1·09 | 1·05 | 0·99 | 1·21 | 0·086 |
|  | Monthly variation in FMT arm (mean ratio) | 1·06 | 1·03 | 0·99 | 1·13 | 0·11 |
|  | Mean ratio of monthly variation between FMT and SP | 0·97 | 1·06 | 0·86 | 1·09 | 0·59 |
| GM-CSF | Mean ratio between FMT and SP | 1·10 | 1·16 | 0·82 | 1·47 | 0·53 |
|  | Monthly variation in SP arm (mean ratio) | 1·00 | 1·02 | 0·97 | 1·03 | 1·00 |
|  | Monthly variation in FMT arm (mean ratio) | 1·00 | 1·01 | 0·98 | 1·02 | 0·87 |
|  | Mean ratio of monthly variation between FMT and SP | 1·00 | 1·02 | 0·96 | 1·04 | 0·93 |
| IFN-γ | Mean ratio between FMT and SP | 0·98 | 1·31 | 0·58 | 1·68 | 0·95 |
|  | Monthly variation in SP arm (mean ratio) | 0·98 | 1·03 | 0·92 | 1·05 | 0·65 |
|  | Monthly variation in FMT arm (mean ratio) | 0·99 | 1·02 | 0·95 | 1·04 | 0·79 |
|  | Mean ratio of monthly variation between FMT and SP | 1·01 | 1·04 | 0·93 | 1·09 | 0·81 |
| MCP-1 | Mean ratio between FMT and SP | 0·9 | 1·35 | 0·50 | 1·63 | 0·72 |
|  | Monthly variation in SP arm (mean ratio) | 0·94 | 1·04 | 0·87 | 1·01 | 0·071 |
|  | Monthly variation in FMT arm (mean ratio) | 0·98 | 1·02 | 0·94 | 1·03 | 0·53 |
|  | Mean ratio of monthly variation between FMT and SP | 1·05 | 1·04 | 0·97 | 1·15 | 0·24 |
| MIP-1α | Mean ratio between FMT and SP | 0·98 | 1·43 | 0·48 | 1·98 | 0·95 |
|  | Monthly variation in SP arm (mean ratio) | 1·00 | 1·05 | 0·91 | 1·09 | 0·93 |
|  | Monthly variation in FMT arm (mean ratio) | 1·00 | 1·03 | 0·94 | 1·06 | 0·95 |
|  | Mean ratio of monthly variation between FMT and SP | 1·00 | 1·05 | 0·90 | 1·11 | 0·97 |
| MIP-1β | Mean ratio between FMT and SP | 1·07 | 1·48 | 0·50 | 2·32 | 0·86 |

|  | Monthly variation in SP arm (mean ratio) | 0·92 | 1·05 | 0·84 | 1·01 | 0·10 |
| --- | --- | --- | --- | --- | --- | --- |
|  | Monthly variation in FMT arm (mean ratio) | 0·98 | 1·03 | 0·92 | 1·04 | 0·49 |
|  | Mean ratio of monthly variation between FMT and SP | 1·06 | 1·06 | 0·95 | 1·19 | 0·32 |
| TNF-α | Mean ratio between FMT and SP | 0·60 | 1·74 | 0·20 | 1·77 | 0·36 |
|  | Monthly variation in SP arm (mean ratio) | 0·96 | 1·07 | 0·84 | 1·09 | 0·51 |
|  | Monthly variation in FMT arm (mean ratio) | 0·97 | 1·05 | 0·89 | 1·06 | 0·52 |
|  | Mean ratio of monthly variation between FMT and SP | 1·02 | 1·08 | 0·87 | 1·19 | 0·85 |
| VEGF | Mean ratio between FMT and SP | 1·32 | 1·24 | 0·87 | 2·01 | 0·19 |
|  | Monthly variation in SP arm (mean ratio) | 1·04 | 1·03 | 0·98 | 1·09 | 0·22 |
|  | Monthly variation in FMT arm (mean ratio) | 0·99 | 1·02 | 0·96 | 1·03 | 0·65 |
|  | Mean ratio of monthly variation between FMT and SP | 0·96 | 1·03 | 0·90 | 1·02 | 0·20 |

For these analyses, laboratory parameters were scaled using a logarithmic transformation, to handle their strongly left-skewed distributions. The term "mean ratio" indicates the ratio of the geometric mean of arm FMT and the geometric mean of arm SP. Monthly variations and differences between monthly variations were estimated using linear mixed-effects models with random intercept. These models included as covariates arm, time and the interaction arm x time. T tests were used to test the null hypothesis that regression coefficients were equal to 0 and obtained p-values reported. Satterthwaite approximation was used to estimate degrees of freedom.

Abbreviations: IL: interleukin, TNFalpha: tumour necrosis factor-alpha; GM-CSF: granulocyte-macrophage colony-stimulating factor; MCP: monocyte-chemoattactant factor; VEGF: vascular endothelial growth factor. FMT: faecal microbiota transplantation; SP: Sham Procedure.

**Supplementary Table 26. Changes from baseline to month 6 and 12 of salivary cytokines across treatment arms. Intention to treat analysis.**

| **Salivary cytokines** | **Time point** | **Arm** |  | | | **change from baseline** | | | |
| --- | --- | --- | --- | --- | --- | --- | --- | --- | --- |
|  |  |  | **N** | **Mean ratio** | **SE** | **MD** | **95% CI** | | **P value** |
| IL-1β | Month 6 | SP | 9 | 0·68 | 0·57 | - | - | - | - |
|  |  | FMT | 17 | 1·96 | 1·05 | 2·90 | 0·64 | 13·15 | 0·18 |
|  | Month 12 | SP | 1 | -^§^ | -^§^ | - | - | - | - |
|  |  | FMT | 9 | 1·46 | 1·53 | -^§^ | -^§^ | -^§^ | -^§^ |
| IL-2 | Month 6 | SP | 9 | 0·80 | 0·29 | - | - | - | - |
|  |  | FMT | 16 | 0·88 | 0·24 | 1·10 | 0·48 | 2·50 | 0·82 |
|  | Month 12 | SP | 1 | -^§^ | -^§^ | - | - | - | - |
|  |  | FMT | 8 | 0·91 | 0·15 | -^§^ | -^§^ | -^§^ | -^§^ |
| IL-4 | Month 6 | SP | 9 | 0·90 | 0·23 | - | - | - | - |
|  |  | FMT | 17 | 1·16 | 0·22 | 1·29 | 0·70 | 2·38 | 0·41 |
|  | Month 12 | SP | 1 | -^§^ | -^§^ | - | - | - | - |
|  |  | FMT | 9 | 1·07 | 0·15 | -^§^ | -^§^ | -^§^ | -^§^ |
| IL-5 | Month 6 | SP | 9 | 1·95 | 1·22 | - | - | - | - |
|  |  | FMT | 17 | 0·71 | 0·31 | 0·36 | 0·10 | 1·28 | 0·12 |
|  | Month 12 | SP | 1 | -^§^ | -^§^ | - | - | - | - |
|  |  | FMT | 9 | 1·13 | 0·64 | -^§^ | -^§^ | -^§^ | -^§^ |
| IL-6 | Month 6 | SP | 9 | 0·75 | 0·47 | - | - | - | - |
|  |  | FMT | 17 | 1·30 | 0·55 | 1·73 | 0·49 | 6·04 | 0·40 |
|  | Month 12 | SP | 1 | -^§^ | -^§^ | - | - | - | - |
|  |  | FMT | 9 | 1·92 | 0·97 | -^§^ | -^§^ | -^§^ | -^§^ |
| IL-7 | Month 6 | SP | 9 | 0·94 | 1·04 | - | - | - | - |
|  |  | FMT | 17 | 1·21 | 0·82 | 1·29 | 0·22 | 7·64 | 0·78 |
|  | Month 12 | SP | 1 | -^§^ | -^§^ | - | - | - | - |
|  |  | FMT | 9 | 0·58 | 0·36 | -^§^ | -^§^ | -^§^ | -^§^ |
| IL-8 | Month 6 | SP | 9 | 0·65 | 0·53 | - | - | - | - |
|  |  | FMT | 17 | 2·14 | 1·08 | 3·27 | 0·75 | 14·29 | 0·12 |
|  | Month 12 | SP | 1 | -^§^ | -^§^ | - | - | - | - |
|  |  | FMT | 9 | 1·87 | 1·84 | -^§^ | -^§^ | -^§^ | -^§^ |
| IL-10 | Month 6 | SP | 9 | 0·93 | 0·42 | - | - | - | - |
|  |  | FMT | 17 | 1·57 | 0·49 | 1·68 | 0·63 | 4·47 | 0·30 |
|  | Month 12 | SP | 1 | -^§^ | -^§^ | - | - | - | - |
|  |  | FMT | 9 | 0·91 | 0·29 | -^§^ | -^§^ | -^§^ | -^§^ |
| IL-12p70 | Month 6 | SP | 9 | 0·92 | 0·48 | - | - | - | - |
|  |  | FMT | 17 | 0·84 | 0·31 | 0·91 | 0·30 | 2·74 | 0·86 |
|  | Month 12 | SP | 1 | -^§^ | -^§^ | - | - | - | - |
|  |  | FMT | 8 | 0·6 | 0·31 | -^§^ | -^§^ | -^§^ | -^§^ |
| IL-13 | Month 6 | SP | 9 | 0·66 | 0·15 | - | - | - | - |
|  |  | FMT | 16 | 1·12 | 0·19 | 1·71 | 0·99 | 2·95 | 0·060 |
|  | Month 12 | SP | 1 | -^§^ | -^§^ | - | - | - | - |
|  |  | FMT | 9 | 1·02 | 0·17 | -^§^ | -^§^ | -^§^ | -^§^ |
| IL-15 | Month 6 | SP | 9 | 1·67 | 5·38 | - | - | - | - |
|  |  | FMT | 17 | 1·46 | 2·13 | 0·87 | 0·05 | 14·41 | 0·93 |
|  | Month 12 | SP | 1 | -^§^ | -^§^ | - | - | - | - |
|  |  | FMT | 9 | 0·83 | 2·72 | -^§^ | -^§^ | -^§^ | -^§^ |
| IL-17 | Month 6 | SP | 9 | 0·84 | 0·29 | - | - | - | - |
|  |  | FMT | 17 | 1·19 | 0·31 | 1·42 | 0·64 | 3·15 | 0·39 |
|  | Month 12 | SP | 1 | -^§^ | -^§^ | - | - | - | - |
|  |  | FMT | 9 | 1·3 | 0·49 | -^§^ | -^§^ | -^§^ | -^§^ |
| G-CSF | Month 6 | SP | 9 | 0·96 | 0·34 | - | - | - | - |
|  |  | FMT | 17 | 0·73 | 0·18 | 0·76 | 0·34 | 1·70 | 0·51 |
|  | Month 12 | SP | 1 | -^§^ | -^§^ | - | - | - | - |
|  |  | FMT | 9 | 0·63 | 0·21 | -^§^ | -^§^ | -^§^ | -^§^ |
| GM-CSF | Month 6 | SP | 9 | 1·06 | 0·51 | - | - | - | - |
|  |  | FMT | 17 | 0·77 | 0·25 | 0·73 | 0·26 | 2·02 | 0·54 |
|  | Month 12 | SP | 1 | -^§^ | -^§^ | - | - | - | - |
|  |  | FMT | 9 | 0·81 | 0·16 | -^§^ | -^§^ | -^§^ | -^§^ |
| IFN-γ | Month 6 | SP | 9 | 1·14 | 0·27 | - | - | - | - |
|  |  | FMT | 17 | 1·10 | 0·2 | 0·97 | 0·55 | 1·70 | 0·91 |
|  | Month 12 | SP | 1 | -^§^ | -^§^ | - | - | - | - |
|  |  | FMT | 9 | 1·32 | 0·36 | -^§^ | -^§^ | -^§^ | -^§^ |
| MCP-1 | Month 6 | SP | 9 | 1·11 | 0·74 | - | - | - | - |
|  |  | FMT | 17 | 1·75 | 0·77 | 1·59 | 0·43 | 5·83 | 0·49 |
|  | Month 12 | SP | 1 | -^§^ | -^§^ | - | - | - | - |
|  |  | FMT | 9 | 1·78 | 1·32 | -^§^ | -^§^ | -^§^ | -^§^ |
| MIP-1α | Month 6 | SP | 9 | 1·20 | 0·4 | - | - | - | - |
|  |  | FMT | 17 | 1·21 | 0·28 | 1·01 | 0·48 | 2·14 | 0·98 |
|  | Month 12 | SP | 1 | -^§^ | -^§^ | - | - | - | - |
|  |  | FMT | 9 | 1·26 | 0·3 | -^§^ | -^§^ | -^§^ | -^§^ |
| MIP1-beta | Month 6 | SP | 9 | 1·21 | 0·44 | - | - | - | - |
|  |  | FMT | 17 | 1·22 | 0·32 | 1·01 | 0·44 | 2·29 | 0·98 |
|  | Month 12 | SP | 1 | -^§^ | -^§^ | - | - | - | - |
|  |  | FMT | 9 | 1·43 | 0·52 | -^§^ | -^§^ | -^§^ | -^§^ |
| TNF-alfa | Month 6 | SP | 9 | 1·02 | 0·6 | - | - | - | - |
|  |  | FMT | 17 | 1·46 | 0·57 | 1·43 | 0·44 | 4·65 | 0·56 |
|  | Month 12 | SP | 1 | -^§^ | -^§^ | - | - | - | - |
|  |  | FMT | 9 | 1·62 | 1·01 | -^§^ | -^§^ | -^§^ | -^§^ |
| VEGF | Month 6 | SP | 9 | 0·82 | 0·55 | - | - | - | - |
|  |  | FMT | 17 | 0·85 | 0·37 | 1·03 | 0·28 | 3·81 | 0·96 |
|  | Month 12 | SP | 1 | -^§^ | -^§^ | - | - | - | - |
|  |  | FMT | 9 | 0·49 | 0·57 | -^§^ | -^§^ | -^§^ | -^§^ |

Mean absolute changes from baseline to month 6 are showed for each treatment group and comparison were performed using linear mixed-effects regression models that include indicator variables for treatment arms, time and the interaction time x arm as independent variables. For these analyses, laboratory parameters were scaled using a logarithmic transformation, to handle their strongly left-skewed distributions. The term "mean ratio" indicates the ratio of the geometric mean at 6 months and the geometric mean at baseline. T tests were used to test the null hypothesis that regression coefficients were equal to 0 and obtained p-values reported. Satterthwaite approximation was used to estimate degrees of freedom.

Given the exploratory nature of these analyses, no adjustment for multiple comparisons was performed and nominal p-values were reported. Abbreviations: N: number of samples for analysis; MD = Mean Difference; SE = Standard Error; CI = Confidence Interval; SP: Sham Procedure; FMT: Faecal Microbiota Transplantation.

^§^ these values couldn’t be calculated as only one observation per SP arm was available.

#### Supplementary Table 27. Average monthly changes of salivary cytokines in the SP and FMT arms, and their ratio over time (estimated from baseline to month 6). Intention-to-treat analysis

|  |  | **Estimate** | **Std· Error** | **95% Conf· Int·** | | **P value** |
| --- | --- | --- | --- | --- | --- | --- |
| IL-1β | Mean ratio between FMT and SP | 0·47 | 1·92 | 0·13 | 1·69 | 0·25 |
|  | Monthly variation in SP arm (mean ratio) | 0·94 | 1·11 | 0·76 | 1·15 | 0·54 |
|  | Monthly variation in FMT arm (mean ratio) | 1·12 | 1·08 | 0·96 | 1·30 | 0·15 |
|  | Mean ratio of monthly variation between FMT and SP | 1·19 | 1·14 | 0·93 | 1·54 | 0·18 |
| IL-2 | Mean ratio between FMT and SP | 0·99 | 1·54 | 0·43 | 2·29 | 0·98 |
|  | Monthly variation in SP arm (mean ratio) | 0·96 | 1·06 | 0·86 | 1·08 | 0·52 |
|  | Monthly variation in FMT arm (mean ratio) | 0·98 | 1·04 | 0·90 | 1·06 | 0·62 |
|  | Mean ratio of monthly variation between FMT and SP | 1·02 | 1·07 | 0·89 | 1·17 | 0·82 |
| IL-4 | Mean ratio between FMT and SP | 0·88 | 1·33 | 0·50 | 1·54 | 0·65 |
|  | Monthly variation in SP arm (mean ratio) | 0·98 | 1·04 | 0·91 | 1·07 | 0·67 |
|  | Monthly variation in FMT arm (mean ratio) | 1·03 | 1·03 | 0·97 | 1·09 | 0·42 |
|  | Mean ratio of monthly variation between FMT and SP | 1·04 | 1·05 | 0·94 | 1·16 | 0·41 |
| IL-5 | Mean ratio between FMT and SP | 0·66 | 1·50 | 0·30 | 1·46 | 0·30 |
|  | Monthly variation in SP arm (mean ratio) | 1·12 | 1·09 | 0·94 | 1·32 | 0·20 |
|  | Monthly variation in FMT arm (mean ratio) | 0·94 | 1·07 | 0·83 | 1·07 | 0·38 |
|  | Mean ratio of monthly variation between FMT and SP | 0·85 | 1·11 | 0·69 | 1·04 | 0·12 |
| IL-6 | Mean ratio between FMT and SP | 0·68 | 1·78 | 0·22 | 2·11 | 0·51 |
|  | Monthly variation in SP arm (mean ratio) | 0·95 | 1·09 | 0·81 | 1·13 | 0·58 |
|  | Monthly variation in FMT arm (mean ratio) | 1·04 | 1·07 | 0·92 | 1·18 | 0·50 |
|  | Mean ratio of monthly variation between FMT and SP | 1·10 | 1·11 | 0·89 | 1·35 | 0·40 |
| IL-7 | Mean ratio between FMT and SP | 2·19 | 1·90 | 0·62 | 7·71 | 0·23 |
|  | Monthly variation in SP arm (mean ratio) | 0·99 | 1·13 | 0·78 | 1·26 | 0·93 |
|  | Monthly variation in FMT arm (mean ratio) | 1·03 | 1·09 | 0·86 | 1·23 | 0·73 |
|  | Mean ratio of monthly variation between FMT and SP | 1·04 | 1·16 | 0·77 | 1·40 | 0·78 |
| IL-8 | Mean ratio between FMT and SP | 0·45 | 1·72 | 0·16 | 1·32 | 0·15 |
|  | Monthly variation in SP arm (mean ratio) | 0·93 | 1·11 | 0·76 | 1·14 | 0·49 |
|  | Monthly variation in FMT arm (mean ratio) | 1·14 | 1·08 | 0·98 | 1·31 | 0·092 |
|  | Mean ratio of monthly variation between FMT and SP | 1·22 | 1·13 | 0·95 | 1·56 | 0·12 |
| IL-10 | Mean ratio between FMT and SP | 1·1 | 1·53 | 0·48 | 2·54 | 0·82 |
|  | Monthly variation in SP arm (mean ratio) | 0·99 | 1·07 | 0·87 | 1·13 | 0·86 |
|  | Monthly variation in FMT arm (mean ratio) | 1·08 | 1·05 | 0·98 | 1·19 | 0·14 |
|  | Mean ratio of monthly variation between FMT and SP | 1·09 | 1·09 | 0·93 | 1·28 | 0·30 |
| IL-12p70 | Mean ratio between FMT and SP | 0·85 | 1·52 | 0·37 | 1·94 | 0·70 |
|  | Monthly variation in SP arm (mean ratio) | 0·99 | 1·08 | 0·85 | 1·14 | 0·86 |
|  | Monthly variation in FMT arm (mean ratio) | 0·97 | 1·06 | 0·87 | 1·08 | 0·60 |
|  | Mean ratio of monthly variation between FMT and SP | 0·98 | 1·10 | 0·82 | 1·18 | 0·86 |
| IL-13 | Mean ratio between FMT and SP | 0·89 | 1·23 | 0·6 | 1·33 | 0·57 |
|  | Monthly variation in SP arm (mean ratio) | 0·93 | 1·04 | 0·87 | 1·00 | 0·075 |
|  | Monthly variation in FMT arm (mean ratio) | 1·02 | 1·03 | 0·97 | 1·08 | 0·48 |
|  | Mean ratio of monthly variation between FMT and SP | 1·09 | 1·05 | 1·00 | 1·20 | 0·065 |
| IL-15 | Mean ratio between FMT and SP | 0·47 | 3·03 | 0·05 | 4·17 | 0·50 |
|  | Monthly variation in SP arm (mean ratio) | 1·09 | 1·21 | 0·75 | 1·59 | 0·66 |
|  | Monthly variation in FMT arm (mean ratio) | 1·06 | 1·15 | 0·81 | 1·40 | 0·66 |
|  | Mean ratio of monthly variation between FMT and SP | 0·98 | 1·27 | 0·61 | 1·56 | 0·93 |
| IL-17 | Mean ratio between FMT and SP | 0·63 | 1·63 | 0·24 | 1·65 | 0·35 |
|  | Monthly variation in SP arm (mean ratio) | 0·97 | 1·06 | 0·87 | 1·08 | 0·59 |
|  | Monthly variation in FMT arm (mean ratio) | 1·03 | 1·04 | 0·95 | 1·12 | 0·47 |
|  | Mean ratio of monthly variation between FMT and SP | 1·06 | 1·07 | 0·93 | 1·21 | 0·39 |
| G-CSF | Mean ratio between FMT and SP | 0·64 | 1·78 | 0·21 | 2·00 | 0·45 |
|  | Monthly variation in SP arm (mean ratio) | 0·99 | 1·06 | 0·89 | 1·11 | 0·89 |
|  | Monthly variation in FMT arm (mean ratio) | 0·95 | 1·04 | 0·88 | 1·03 | 0·20 |
|  | Mean ratio of monthly variation between FMT and SP | 0·96 | 1·07 | 0·84 | 1·09 | 0·51 |
| GM-CSF | Mean ratio between FMT and SP | 0·75 | 1·44 | 0·36 | 1·53 | 0·43 |
|  | Monthly variation in SP arm (mean ratio) | 1·01 | 1·07 | 0·88 | 1·16 | 0·88 |
|  | Monthly variation in FMT arm (mean ratio) | 0·96 | 1·05 | 0·87 | 1·06 | 0·41 |
|  | Mean ratio of monthly variation between FMT and SP | 0·95 | 1·09 | 0·80 | 1·12 | 0·54 |
| IFNg | Mean ratio between FMT and SP | 0·82 | 1·32 | 0·48 | 1·42 | 0·48 |
|  | Monthly variation in SP arm (mean ratio) | 1·02 | 1·04 | 0·95 | 1·10 | 0·57 |
|  | Monthly variation in FMT arm (mean ratio) | 1·02 | 1·03 | 0·96 | 1·07 | 0·57 |
|  | Mean ratio of monthly variation between FMT and SP | 0·99 | 1·05 | 0·91 | 1·09 | 0·91 |
| MCP-1 | Mean ratio between FMT and SP | 1·06 | 1·68 | 0·38 | 2·93 | 0·91 |
|  | Monthly variation in SP arm (mean ratio) | 1·02 | 1·09 | 0·85 | 1·21 | 0·85 |
|  | Monthly variation in FMT arm (mean ratio) | 1·10 | 1·07 | 0·97 | 1·25 | 0·16 |
|  | Mean ratio of monthly variation between FMT and SP | 1·08 | 1·12 | 0·87 | 1·34 | 0·49 |
| MIP-1α | Mean ratio between FMT and SP | 1·18 | 1·4 | 0·61 | 2·27 | 0·63 |

|  | Monthly variation in SP arm (mean ratio) | 1·03 | 1·05 | 0·93 | 1·14 | 0·56 |
| --- | --- | --- | --- | --- | --- | --- |
|  | Monthly variation in FMT arm (mean ratio) | 1·03 | 1·04 | 0·96 | 1·11 | 0·40 |
|  | Mean ratio of monthly variation between FMT and SP | 1·00 | 1·07 | 0·88 | 1·14 | 0·98 |
| MIP-1β | Mean ratio between FMT and SP | 0·90 | 1·61 | 0·36 | 2·30 | 0·83 |
|  | Monthly variation in SP arm (mean ratio) | 1·03 | 1·06 | 0·92 | 1·15 | 0·58 |
|  | Monthly variation in FMT arm (mean ratio) | 1·03 | 1·04 | 0·95 | 1·12 | 0·43 |
|  | Mean ratio of monthly variation between FMT and SP | 1·00 | 1·07 | 0·87 | 1·15 | 0·98 |
| TNFalpha | Mean ratio between FMT and SP | 0·47 | 1·85 | 0·14 | 1·56 | 0·22 |
|  | Monthly variation in SP arm (mean ratio) | 1·00 | 1·08 | 0·86 | 1·18 | 0·97 |
|  | Monthly variation in FMT arm (mean ratio) | 1·06 | 1·06 | 0·95 | 1·20 | 0·30 |
|  | Mean ratio of monthly variation between FMT and SP | 1·06 | 1·11 | 0·87 | 1·29 | 0·56 |
| VEGF | Mean ratio between FMT and SP | 0·67 | 2·72 | 0·09 | 4·77 | 0·69 |
|  | Monthly variation in SP arm (mean ratio) | 0·97 | 1·09 | 0·81 | 1·15 | 0·72 |
|  | Monthly variation in FMT arm (mean ratio) | 0·97 | 1·07 | 0·86 | 1·11 | 0·68 |
|  | Mean ratio of monthly variation between FMT and SP | 1·01 | 1·12 | 0·81 | 1·25 | 0·96 |

For these analyses, laboratory parameters were scaled using a logarithmic transformation, to handle their strongly left-skewed distributions. The term "mean ratio" indicates the ratio of the geometric mean of the FMT arm and the geometric mean of the SP arm. Monthly variation computed between 0 and 6 months. Monthly variations and differences between monthly variations were estimated using linear mixed-effects models with random intercept. These models included as covariates arm, time and the interaction arm x time. T tests were used to test the null hypothesis that regression coefficients were equal to 0 and obtained p-values reported. Satterthwaite approximation was used to estimate degrees of freedom.

Abbreviations: IL: interleukin, TNFalpha: tumour necrosis factor-alpha; GM-CSF: granulocyte-macrophage colony-stimulating factor; G-CSF: granulocyte colony-stimulating factor; MCP: monocyte-chemoattactant factor; MIP-1α: macrophage inflammatory protein-1-alpha; MIP-1β: macrophage inflammatory protein-1-beta; VEGF: vascular endothelial growth factor. FMT: faecal microbiota transplantation; SP: Sham Procedure

#### Supplementary Table 28. Changes from baseline to month 6 and 12 of homogenized intestinal tissue cytokines across treatment arms. Intention to treat analysis.

| **Tissue cytokines** | **Time point** | **Arm** |  | | | **change from baseline** | | | |
| --- | --- | --- | --- | --- | --- | --- | --- | --- | --- |
|  |  |  | **N** | **Mean ratio** | **SE** | **MD** | **95% CI** | | **P value** |
| IL-1β | Month 6 | SP | 10 | 1·23 | 0·3 | - | - | - | - |
|  |  | FMT | 19 | 0·89 | 0·16 | 0·73 | 0·41 | 1·29 | 0·28 |
|  | Month 12 | SP | 1 | -^§^ | -^§^ | - | - | - | - |
|  |  | FMT | 10 | 0·55 | 0·11 | -^§^ | -^§^ | -^§^ | -^§^ |
| IL-2 | Month 6 | SP | 10 | 1·11 | 0·37 | - | - | - | - |
|  |  | FMT | 19 | 0·86 | 0·2 | 0·78 | 0·37 | 1·65 | 0·52 |
|  | Month 12 | SP | 1 | -^§^ | -^§^ | - | - | - | - |
|  |  | FMT | 10 | 1·48 | 0·4 | -^§^ | -^§^ | -^§^ | -^§^ |
| IL-4 | Month 6 | SP | 10 | 1·62 | 0·48 | - | - | - | - |
|  |  | FMT | 19 | 1·13 | 0·24 | 0·70 | 0·36 | 1·37 | 0·31 |
|  | Month 12 | SP | 1 | -^§^ | -^§^ | - | - | - | - |
|  |  | FMT | 10 | 1·51 | 0·51 | -^§^ | -^§^ | -^§^ | -^§^ |
| IL-5 | Month 6 | SP | 10 | 0·70 | 0·4 | - | - | - | - |
|  |  | FMT | 19 | 0·79 | 0·31 | 1·13 | 0·35 | 3·67 | 0·84 |
|  | Month 12 | SP | 1 | -^§^ | -^§^ | - | - | - | - |
|  |  | FMT | 10 | 0·25 | 0·16 | -^§^ | -^§^ | -^§^ | -^§^ |
| IL-6 | Month 6 | SP | 10 | 1·16 | 0·37 | - | - | - | - |
|  |  | FMT | 19 | 1·35 | 0·31 | 1·17 | 0·56 | 2·43 | 0·68 |
|  | Month 12 | SP | 1 | -^§^ | -^§^ | - | - | - | - |
|  |  | FMT | 10 | 1·22 | 0·56 | -^§^ | -^§^ | -^§^ | -^§^ |
| IL-7 | Month 6 | SP | 10 | 1·05 | 0·24 | - | - | - | - |
|  |  | FMT | 19 | 0·96 | 0·16 | 0·91 | 0·53 | 1·58 | 0·75 |
|  | Month 12 | SP | 1 | -^§^ | -^§^ | - | - | - | - |
|  |  | FMT | 10 | 0·74 | 0·1 | -^§^ | -^§^ | -^§^ | -^§^ |
| IL-8 | Month 6 | SP | 10 | 0·90 | 0·28 | - | - | - | - |
|  |  | FMT | 19 | 1·39 | 0·31 | 1·53 | 0·75 | 3·12 | 0·25 |
|  | Month 12 | SP | 1 | -^§^ | -^§^ | - | - | - | - |
|  |  | FMT | 10 | 1·42 | 0·42 | -^§^ | -^§^ | -^§^ | -^§^ |
| IL-10 | Month 6 | SP | 10 | 1·60 | 0·41 | - | - | - | - |
|  |  | FMT | 19 | 1·08 | 0·2 | 0·67 | 0·37 | 1·23 | 0·21 |
|  | Month 12 | SP | 1 | -^§^ | -^§^ | - | - | - | - |
|  |  | FMT | 10 | 1·02 | 0·34 | -^§^ | -^§^ | -^§^ | -^§^ |
| IL-12p70 | Month 6 | SP | 10 | 1·56 | 0·36 | - | - | - | - |
|  |  | FMT | 19 | 1·10 | 0·19 | 0·70 | 0·41 | 1·21 | 0·22 |
|  | Month 12 | SP | 1 | -^§^ | -^§^ | - | - | - | - |
|  |  | FMT | 10 | 0·87 | 0·17 | -^§^ | -^§^ | -^§^ | -^§^ |
| IL-13 | Month 6 | SP | 10 | 1·15 | 0·3 | - | - | - | - |
|  |  | FMT | 19 | 0·89 | 0·17 | 0·78 | 0·43 | 1·42 | 0·43 |
|  | Month 12 | SP | 1 | -^§^ | -^§^ | - | - | - | - |
|  |  | FMT | 10 | 0·75 | 0·1 | -^§^ | -^§^ | -^§^ | -^§^ |
| IL-15 | Month 6 | SP | 10 | 1·13 | 2·74 | - | - | - | - |
|  |  | FMT | 19 | 0·72 | 0·85 | 0·64 | 0·05 | 8·10 | 0·73 |
|  | Month 12 | SP | 1 | -^§^ | -^§^ | - | - | - | - |
|  |  | FMT | 10 | 5·46 | 26·74 | -^§^ | -^§^ | -^§^ | -^§^ |
| IL-17 | Month 6 | SP | 10 | 1·18 | 0·25 | - | - | - | - |
|  |  | FMT | 19 | 1·17 | 0·18 | 0·99 | 0·60 | 1·62 | 0·96 |
|  | Month 12 | SP | 1 | -^§^ | -^§^ | - | - | - | - |
|  |  | FMT | 10 | 1·64 | 0·34 | -^§^ | -^§^ | -^§^ | -^§^ |
| G-CSF | Month 6 | SP | 10 | 1·03 | 0·25 | - | - | - | - |
|  |  | FMT | 19 | 0·95 | 0·17 | 0·92 | 0·52 | 1·62 | 0·78 |
|  | Month 12 | SP | 1 | -^§^ | -^§^ | - | - | - | - |
|  |  | FMT | 10 | 0·36 | 0·19 | -^§^ | -^§^ | -^§^ | -^§^ |
| GM-CSF | Month 6 | SP | 10 | 1·26 | 0·42 | - | - | - | - |
|  |  | FMT | 19 | 1·31 | 0·3 | 1·04 | 0·49 | 2·18 | 0·92 |
|  | Month 12 | SP | 1 | -^§^ | -^§^ | - | - | - | - |
|  |  | FMT | 10 | 0·89 | 0·07 | -^§^ | -^§^ | -^§^ | -^§^ |
| IFN-γ | Month 6 | SP | 10 | 1·03 | 0·24 | - | - | - | - |
|  |  | FMT | 19 | 0·95 | 0·16 | 0·92 | 0·53 | 1·59 | 0·77 |
|  | Month 12 | SP | 1 | -^§^ | -^§^ | - | - | - | - |
|  |  | FMT | 10 | 0·68 | 0·08 | -^§^ | -^§^ | -^§^ | -^§^ |
| MCP-1 | Month 6 | SP | 10 | 0·58 | 0·13 | - | - | - | - |
|  |  | FMT | 19 | 0·98 | 0·17 | 1·68 | 0·98 | 2·88 | 0·072 |
|  | Month 12 | SP | 1 | -^§^ | -^§^ | - | - | - | - |
|  |  | FMT | 10 | 1·08 | 0·44 | -^§^ | -^§^ | -^§^ | -^§^ |
| MIP-1α | Month 6 | SP | 10 | 0·52 | 0·12 | - | - | - | - |
|  |  | FMT | 19 | 1·12 | 0·18 | 2·16 | 1·29 | 3·64 | 0·0071 |
|  | Month 12 | SP | 1 | -^§^ | -^§^ | - | - | - | - |
|  |  | FMT | 10 | 1·36 | 0·49 | -^§^ | -^§^ | -^§^ | -^§^ |
| MIP-1β | Month 6 | SP | 10 | 0·62 | 0·27 | - | - | - | - |
|  |  | FMT | 19 | 0·95 | 0·29 | 1·54 | 0·59 | 4·00 | 0·39 |
|  | Month 12 | SP | 1 | -^§^ | -^§^ | - | - | - | - |
|  |  | FMT | 10 | 1·25 | 0·49 | -^§^ | -^§^ | -^§^ | -^§^ |
| TNF-α | Month 6 | SP | 10 | 1·06 | 0·16 | - | - | - | - |
|  |  | FMT | 19 | 1·22 | 0·14 | 1·15 | 0·79 | 1·66 | 0·47 |
|  | Month 12 | SP | 1 | -^§^ | -^§^ | - | - | - | - |
|  |  | FMT | 10 | 1·43 | 0·29 | -^§^ | -^§^ | -^§^ | -^§^ |
| VEGF | Month 6 | SP | 10 | 0·42 | 2·48 | - | - | - | - |
|  |  | FMT | 19 | 1·63 | 3·46 | 3·84 | 0·14 | 104·80 | 0·43 |
|  | Month 12 | SP | 1 | -^§^ | -^§^ | - | - | - | - |
|  |  | FMT | 10 | 0·16 | 0·58 | -^§^ | -^§^ | -^§^ | -^§^ |

Mean absolute changes from baseline to month 6 are showed for each treatment group and comparison were performed using linear mixed-effects regression models that include indicator variables for treatment arms. time and the interaction time x arm as independent variables. For these analyses, laboratory parameters were scaled using a logarithmic transformation, to handle their strongly left-skewed distributions. The term "mean ratio" indicates the ratio of the geometric mean at 6 months and the geometric mean at baseline. Significant changes between treatment arms were highlighted in bold characters. T tests were used to test the null hypothesis that regression coefficients were equal to 0 and obtained p-values reported. Satterthwaite approximation was used to estimate degrees of freedom.

Given the exploratory nature of these analyses, no adjustment for multiple comparisons was performed and nominal p-values were reported. Abbreviations: N: number of samples for analysis; MD = Mean Difference; SE = Standard Error; CI = Confidence Interval; SP: Sham Procedure; FMT: Faecal Microbiota Transplantation.

^§^ these values couldn’t be calculated as only one observation per SP arm was available.

#### Supplementary Table 29. Average monthly changes of homogenized intestinal tissue sample cytokines in the SP and FMT arms, and their ratio over time (estimated from baseline to month 6). Intention-to-treat analysis

|  |  | **Estimate** | **Std· Error** | **95% Conf· Int·** | | **P value** |
| --- | --- | --- | --- | --- | --- | --- |
| IL-1β | Mean ratio between FMT and SP | 1·08 | 1·23 | 0·72 | 1·62 | 0·71 |
|  | Monthly variation in SP arm (mean ratio) | 1·04 | 1·04 | 0·96 | 1·12 | 0·38 |
|  | Monthly variation in FMT arm (mean ratio) | 0·98 | 1·03 | 0·93 | 1·04 | 0·53 |
|  | Mean ratio of monthly variation between FMT and SP | 0·95 | 1·05 | 0·86 | 1·04 | 0·28 |
| IL-2 | Mean ratio between FMT and SP | 0·88 | 1·38 | 0·47 | 1·67 | 0·71 |
|  | Monthly variation in SP arm (mean ratio) | 1·02 | 1·05 | 0·92 | 1·12 | 0·75 |
|  | Monthly variation in FMT arm (mean ratio) | 0·98 | 1·04 | 0·91 | 1·05 | 0·52 |
|  | Mean ratio of monthly variation between FMT and SP | 0·96 | 1·07 | 0·85 | 1·09 | 0·52 |
| IL-4 | Mean ratio between FMT and SP | 1·13 | 1·30 | 0·67 | 1·9 | 0·65 |
|  | Monthly variation in SP arm (mean ratio) | 1·08 | 1·05 | 0·99 | 1·19 | 0·094 |
|  | Monthly variation in FMT arm (mean ratio) | 1·02 | 1·03 | 0·95 | 1·09 | 0·55 |
|  | Mean ratio of monthly variation between FMT and SP | 0·94 | 1·06 | 0·84 | 1·05 | 0·31 |
| IL-5 | Mean ratio between FMT and SP | 1·87 | 1·63 | 0·71 | 4·87 | 0·21 |
|  | Monthly variation in SP arm (mean ratio) | 0·94 | 1·08 | 0·8 | 1·11 | 0·47 |
|  | Monthly variation in FMT arm (mean ratio) | 0·96 | 1·06 | 0·86 | 1·08 | 0·52 |
|  | Mean ratio of monthly variation between FMT and SP | 1·02 | 1·11 | 0·84 | 1·24 | 0·84 |
| IL-6 | Mean ratio between FMT and SP | 0·9 | 1·49 | 0·41 | 1·95 | 0·78 |
|  | Monthly variation in SP arm (mean ratio) | 1·02 | 1·05 | 0·93 | 1·13 | 0·63 |
|  | Monthly variation in FMT arm (mean ratio) | 1·05 | 1·04 | 0·98 | 1·13 | 0·18 |
|  | Mean ratio of monthly variation between FMT and SP | 1·03 | 1·06 | 0·91 | 1·16 | 0·68 |
| IL-7 | Mean ratio between FMT and SP | 1·03 | 1·20 | 0·72 | 1·47 | 0·87 |
|  | Monthly variation in SP arm (mean ratio) | 1·01 | 1·04 | 0·94 | 1·08 | 0·83 |
|  | Monthly variation in FMT arm (mean ratio) | 0·99 | 1·03 | 0·94 | 1·05 | 0·81 |
|  | Mean ratio of monthly variation between FMT and SP | 0·99 | 1·05 | 0·9 | 1·08 | 0·75 |
| IL-8 | Mean ratio between FMT and SP | 0·85 | 1·32 | 0·49 | 1·47 | 0·56 |
|  | Monthly variation in SP arm (mean ratio) | 0·98 | 1·05 | 0·89 | 1·08 | 0·73 |
|  | Monthly variation in FMT arm (mean ratio) | 1·06 | 1·04 | 0·98 | 1·13 | 0·14 |
|  | Mean ratio of monthly variation between FMT and SP | 1·07 | 1·06 | 0·95 | 1·21 | 0·25 |
| IL-10 | Mean ratio between FMT and SP | 1·34 | 1·27 | 0·83 | 2·14 | 0·23 |
|  | Monthly variation in SP arm (mean ratio) | 1·08 | 1·04 | 1·00 | 1·17 | 0·068 |
|  | Monthly variation in FMT arm (mean ratio) | 1·01 | 1·03 | 0·95 | 1·07 | 0·68 |
|  | Mean ratio of monthly variation between FMT and SP | 0·94 | 1·05 | 0·85 | 1·04 | 0·21 |
| IL-12p70 | Mean ratio between FMT and SP | 1·1 | 1·25 | 0·71 | 1·71 | 0·67 |
|  | Monthly variation in SP arm (mean ratio) | 1·08 | 1·04 | 1·00 | 1·16 | 0·058 |
|  | Monthly variation in FMT arm (mean ratio) | 1·02 | 1·03 | 0·96 | 1·07 | 0·58 |
|  | Mean ratio of monthly variation between FMT and SP | 0·94 | 1·05 | 0·86 | 1·03 | 0·22 |
| IL-13 | Mean ratio between FMT and SP | 1·11 | 1·28 | 0·69 | 1·8 | 0·66 |
|  | Monthly variation in SP arm (mean ratio) | 1·02 | 1·04 | 0·94 | 1·11 | 0·59 |
|  | Monthly variation in FMT arm (mean ratio) | 0·98 | 1·03 | 0·92 | 1·04 | 0·54 |
|  | Mean ratio of monthly variation between FMT and SP | 0·96 | 1·05 | 0·87 | 1·06 | 0·43 |
| IL-15 | Mean ratio between FMT and SP | 2·95 | 3·19 | 0·3 | 28·66 | 0·35 |
|  | Monthly variation in SP arm (mean ratio) | 1·02 | 1·19 | 0·73 | 1·44 | 0·91 |
|  | Monthly variation in FMT arm (mean ratio) | 0·95 | 1·14 | 0·74 | 1·21 | 0·67 |
|  | Mean ratio of monthly variation between FMT and SP | 0·93 | 1·24 | 0·61 | 1·42 | 0·73 |
| IL-17 | Mean ratio between FMT and SP | 0·98 | 1·2 | 0·69 | 1·39 | 0·92 |
|  | Monthly variation in SP arm (mean ratio) | 1·03 | 1·03 | 0·96 | 1·1 | 0·42 |
|  | Monthly variation in FMT arm (mean ratio) | 1·03 | 1·03 | 0·98 | 1·08 | 0·32 |
|  | Mean ratio of monthly variation between FMT and SP | 1·00 | 1·04 | 0·92 | 1·08 | 0·96 |
| G-CSF | Mean ratio between FMT and SP | 0·95 | 1·21 | 0·65 | 1·37 | 0·77 |
|  | Monthly variation in SP arm (mean ratio) | 1·01 | 1·04 | 0·93 | 1·08 | 0·89 |
|  | Monthly variation in FMT arm (mean ratio) | 0·99 | 1·03 | 0·94 | 1·05 | 0·78 |
|  | Mean ratio of monthly variation between FMT and SP | 0·99 | 1·05 | 0·90 | 1·08 | 0·78 |
| GM-CSF | Mean ratio between FMT and SP | 1·03 | 1·28 | 0·63 | 1·67 | 0·92 |
|  | Monthly variation in SP arm (mean ratio) | 1·04 | 1·05 | 0·94 | 1·15 | 0·45 |
|  | Monthly variation in FMT arm (mean ratio) | 1·05 | 1·04 | 0·97 | 1·13 | 0·24 |
|  | Mean ratio of monthly variation between FMT and SP | 1·01 | 1·07 | 0·89 | 1·14 | 0·92 |
| IFNgamma | Mean ratio between FMT and SP | 0·94 | 1·21 | 0·64 | 1·37 | 0·74 |
|  | Monthly variation in SP arm (mean ratio) | 1·00 | 1·04 | 0·93 | 1·08 | 0·91 |
|  | Monthly variation in FMT arm (mean ratio) | 0·99 | 1·03 | 0·94 | 1·05 | 0·74 |
|  | Mean ratio of monthly variation between FMT and SP | 0·99 | 1·05 | 0·9 | 1·08 | 0·77 |
| MCP-1 | Mean ratio between FMT and SP | 0·83 | 1·40 | 0·43 | 1·61 | 0·59 |
|  | Monthly variation in SP arm (mean ratio) | 0·91 | 1·04 | 0·85 | 0·98 | 0·023 |
|  | Monthly variation in FMT arm (mean ratio) | 1·00 | 1·03 | 0·94 | 1·05 | 0·89 |
|  | Mean ratio of monthly variation between FMT and SP | 1·09 | 1·05 | 1·00 | 1·19 | 0·072 |
| MIP-1α | Mean ratio between FMT and SP | 0·76 | 1·35 | 0·42 | 1·38 | 0·37 |

|  | Monthly variation in SP arm (mean ratio) | 0·90 | 1·04 | 0·84 | 0·96 | 0·0051 |
| --- | --- | --- | --- | --- | --- | --- |
|  | Monthly variation in FMT arm (mean ratio) | 1·02 | 1·03 | 0·97 | 1·07 | 0·48 |
|  | Mean ratio of monthly variation between FMT and SP | 1·14 | 1·05 | 1·04 | 1·24 | 0·0072 |
| MIP-1β | Mean ratio between FMT and SP | 0·87 | 1·61 | 0·34 | 2·2 | 0·77 |
|  | Monthly variation in SP arm (mean ratio) | 0·92 | 1·07 | 0·81 | 1·05 | 0·23 |
|  | Monthly variation in FMT arm (mean ratio) | 0·99 | 1·05 | 0·9 | 1·09 | 0·87 |
|  | Mean ratio of monthly variation between FMT and SP | 1·07 | 1·08 | 0·92 | 1·26 | 0·39 |
| TNFalpha | Mean ratio between FMT and SP | 1·03 | 1·20 | 0·72 | 1·46 | 0·88 |
|  | Monthly variation in SP arm (mean ratio) | 1·01 | 1·03 | 0·96 | 1·06 | 0·69 |
|  | Monthly variation in FMT arm (mean ratio) | 1·03 | 1·02 | 1·00 | 1·07 | 0·084 |
|  | Mean ratio of monthly variation between FMT and SP | 1·02 | 1·03 | 0·96 | 1·09 | 0·47 |
| VEGF | Mean ratio between FMT and SP | 0·22 | 3·16 | 0·02 | 2·06 | 0·19 |
|  | Monthly variation in SP arm (mean ratio) | 0·87 | 1·26 | 0·56 | 1·35 | 0·53 |
|  | Monthly variation in FMT arm (mean ratio) | 1·08 | 1·18 | 0·78 | 1·5 | 0·63 |
|  | Mean ratio of monthly variation between FMT and SP | 1·25 | 1·32 | 0·72 | 2·17 | 0·43 |

For these analyses, laboratory parameters were scaled using a logarithmic transformation, to handle their strongly left-skewed distributions. The term "mean ratio" indicates the ratio of the geometric mean of FMT-treated arm and the geometric mean of the SP arm. Monthly variations were computed between 0 and 6 months. Monthly variations and differences between monthly variations were estimated using linear mixed-effects models with random intercept. These models included as covariates arm, time and the interaction arm x time. T tests were used to test the null hypothesis that regression coefficients were equal to 0 and obtained p-values reported. Satterthwaite approximation was used to estimate degrees of freedom. Significant changes between treatment arms were highlighted in bold characters.

Abbreviations: IL: interleukin, TNFalpha: tumour necrosis factor-alpha; GM-CSF: granulocyte-macrophage colony-stimulating factor; G-CSF: granulocyte colony-stimulating factor; MCP: monocyte-chemoattactant factor; MIP-1α: macrophage inflammatory protein-1-alpha; MIP-1β: macrophage inflammatory protein-1-beta; VEGF: vascular endothelial growth factor. FMT: faecal microbiota transplantation; SP: Sham Procedure.

#### Supplementary Table 30. Changes from baseline to month 6 and 12 of faecal cytokines across treatment arms.

#### Intention to treat analysis

| **Faecal cytokines** | **Time point** | **Arm** |  | | | **change from baseline** | | | |
| --- | --- | --- | --- | --- | --- | --- | --- | --- | --- |
|  |  |  | **N** | **Mean ratio** | **SE** | **MD** | **95% CI** | | **P value** |
| IL-1β | Month 6 | SP | 6 | 0·50 | 1·69 | - | - | - | - |
|  |  | FMT | 13 | 0·60 | 0·8 | 1·22 | 0·08 | 19·73 | 0·89 |
|  | Month 12 | SP | 1 | -^§^ | -^§^ | - | - | - | - |
|  |  | FMT | 5 | 1·34 | 4·78 | -^§^ | -^§^ | -^§^ | -^§^ |
| IL-2 | Month 6 | SP | 6 | 0·49 | 1·06 | - | - | - | - |
|  |  | FMT | 13 | 0·50 | 0·51 | 1·00 | 0·09 | 10·95 | 0·99 |
|  | Month 12 | SP | 1 | -^§^ | -^§^ | - | - | - | - |
|  |  | FMT | 5 | 2·08 | 9·71 | -^§^ | -^§^ | -^§^ | -^§^ |
| IL-4 | Month 6 | SP | 6 | -^#^ | -^#^ | - | - | - | - |
|  |  | FMT | 13 | -^#^ | -^#^ | -^#^ | -^#^ | -^#^ | -^#^ |
|  | Month 12 | SP | 1 | -^§^ | -^§^ | - | - | - | - |
|  |  | FMT | 5 | -^#^ | -^#^ | -^#^ | -^#^ | -^#^ | -^#^ |
| IL-5 | Month 6 | SP | 6 | 0·88 | 0·06 | - | - | - | - |
|  |  | FMT | 13 | 1·00 | 0·05 | 1·14 | 0·97 | 1·33 | 0·14 |
|  | Month 12 | SP | 1 | -^§^ | -^§^ | - | - | - | - |
|  |  | FMT | 5 | -^#^ | -^#^ | -^#^ | -^#^ | -^#^ | -^#^ |
| IL-6 | Month 6 | SP | 6 | 1·00 | 1·04 | - | - | - | - |
|  |  | FMT | 13 | 0·54 | 0·32 | 0·54 | 0·10 | 2·91 | 0·47 |
|  | Month 12 | SP | 1 | -^§^ | -^§^ | - | - | - | - |
|  |  | FMT | 5 | 0·48 | 0·66 | -^§^ | -^§^ | -^§^ | -^§^ |
| IL-7 | Month 6 | SP | 4 | -^#^ | -^#^ | - | - | - | - |
|  |  | FMT | 9 | -^#^ | -^#^ | -^#^ | -^#^ | -^#^ | -^#^ |
|  | Month 12 | SP | 1 | -^§^ | -^§^ | - | - | - | - |
|  |  | FMT | 1 | -^§^ | -^§^ | -^§^ | -^§^ | -^§^ | -^§^ |
| IL-8 | Month 6 | SP | 6 | 5·02 | 3·83 | - | - | - | - |
|  |  | FMT | 13 | 1·09 | 0·5 | 0·22 | 0·05 | 0·88 | 0·039 |
|  | Month 12 | SP | 1 | -^§^ | -^§^ | - | - | - | - |
|  |  | FMT | 5 | 0·9 | 0·54 | -^§^ | -^§^ | -^§^ | -^§^ |
| IL-10 | Month 6 | SP | 6 | 0·77 | 0·79 | - | - | - | - |
|  |  | FMT | 13 | 0·64 | 0·37 | 0·84 | 0·16 | 4·48 | 0·84 |
|  | Month 12 | SP | 1 | -^§^ | -^§^ | - | - | - | - |
|  |  | FMT | 5 | 1·46 | 1·65 | -^§^ | -^§^ | -^§^ | -^§^ |
| IL-12p70 | Month 6 | SP | 6 | 2·83 | 4·09 | - | - | - | - |
|  |  | FMT | 13 | 1·00 | 0·75 | 0·36 | 0·05 | 2·64 | 0·33 |
|  | Month 12 | SP | 1 | -^§^ | -^§^ | - | - | - | - |
|  |  | FMT | 5 | 0·54 | 0·74 | -^§^ | -^§^ | -^§^ | -^§^ |
| IL-13 | Month 6 | SP | 6 | 2·07 | 1·49 | - | - | - | - |
|  |  | FMT | 13 | 0·71 | 0·31 | 0·34 | 0·09 | 1·32 | 0·13 |
|  | Month 12 | SP | 1 | -^§^ | -^§^ | - | - | - | - |
|  |  | FMT | 5 | 1·65 | 1·47 | -^§^ | -^§^ | -^§^ | -^§^ |
| IL-15 | Month 6 | SP | 4 | 1·00 | 5·42 | - | - | - | - |
|  |  | FMT | 9 | 5·66 | 9·78 | 5·66 | 0·24 | 134·68 | 0·29 |
|  | Month 12 | SP | 1 | -^§^ | -^§^ | - | - | - | - |
|  |  | FMT | 1 | -^§^ | -^§^ | -^§^ | -^§^ | -^§^ | -^§^ |
| IL-17 | Month 6 | SP | 5 | 0·89 | 1·65 | - | - | - | - |
|  |  | FMT | 13 | 1·28 | 1·06 | 1·45 | 0·16 | 13·11 | 0·74 |
|  | Month 12 | SP | 1 | -^§^ | -^§^ | - | - | - | - |
|  |  | FMT | 5 | 1·02 | 1·83 | -^§^ | -^§^ | -^§^ | -^§^ |
| G-CSF | Month 6 | SP | 3 | 9·06 | 63·5 | - | - | - | - |
|  |  | FMT | 9 | 0·97 | 1·59 | 0·11 | 0·00 | 2·88 | 0·20 |
|  | Month 12 | SP | 1 | -^§^ | -^§^ | - | - | - | - |
|  |  | FMT | 1 | -^§^ | -^§^ | -^§^ | -^§^ | -^§^ | -^§^ |
| GM-CSF | Month 6 | SP | 6 | 0·24 | 0·81 | - | - | - | - |
|  |  | FMT | 13 | 0·97 | 1·16 | 4·04 | 0·26 | 62·23 | 0·32 |
|  | Month 12 | SP | 1 | -^§^ | -^§^ | - | - | - | - |
|  |  | FMT | 5 | 0·47 | 1·14 | -^§^ | -^§^ | -^§^ | -^§^ |
| IFN-γ | Month 6 | SP | 6 | 1·46 | 1·81 | - | - | - | - |
|  |  | FMT | 13 | 0·98 | 0·65 | 0·67 | 0·11 | 4·30 | 0·68 |
|  | Month 12 | SP | 1 | -^§^ | -^§^ | - | - | - | - |
|  |  | FMT | 5 | 0·47 | 0·43 | -^§^ | -^§^ | -^§^ | -^§^ |
| MCP-1 | Month 6 | SP | 6 | 0·51 | 0·77 | - | - | - | - |
|  |  | FMT | 13 | 0·52 | 0·4 | 1·02 | 0·13 | 7·85 | 0·99 |
|  | Month 12 | SP | 1 | -^§^ | -^§^ | - | - | - | - |
|  |  | FMT | 5 | 2·37 | 5·28 | -^§^ | -^§^ | -^§^ | -^§^ |
| MIP-1α | Month 6 | SP | 4 | 1·00 | 0·65 | - | - | - | - |
|  |  | FMT | 9 | 1·80 | 0·71 | 1·80 | 0·52 | 6·23 | 0·36 |
|  | Month 12 | SP | 1 | -^§^ | -^§^ | - | - | - | - |
|  |  | FMT | 1 | -^§^ | -^§^ | -^§^ | -^§^ | -^§^ | -^§^ |
| MIP-1β | Month 6 | SP | 6 | 1·00 | 0·76 | - | - | - | - |
|  |  | FMT | 13 | 2·42 | 1·11 | 2·42 | 0·60 | 9·80 | 0·22 |
|  | Month 12 | SP | 1 | -^§^ | -^§^ | - | - | - | - |
|  |  | FMT | 5 | 6·64 | 4·78 | -^§^ | -^§^ | -^§^ | -^§^ |
| TNF-α | Month 6 | SP | 6 | 0·74 | 1·84 | - | - | - | - |
|  |  | FMT | 13 | 0·66 | 0·71 | 0·89 | 0·07 | 10·88 | 0·93 |
|  | Month 12 | SP | 1 | -^§^ | -^§^ | - | - | - | - |
|  |  | FMT | 5 | 2·66 | 9·4 | -^§^ | -^§^ | -^§^ | -^§^ |
| VEGF | Month 6 | SP | 4 | -^#^ | -^#^ | - | - | - | - |
|  |  | FMT | 9 | -^#^ | -^#^ | -^#^ | -^#^ | -^#^ | -^#^ |
|  | Month 12 | SP | 1 | -^§^ | -^§^ | - | - | - | - |
|  |  | FMT | 1 | -^§^ | -^§^ | -^§^ | -^§^ | -^§^ | -^§^ |

Mean absolute changes from baseline to month 6 are showed for each treatment group and comparison were performed using linear mixed-effects regression models that include indicator variables for treatment arms, time and the interaction time x arm as independent variables. For these analyses, laboratory parameters were scaled using a logarithmic transformation, to handle their strongly left-skewed distributions. The term "mean ratio" indicates the ratio of the geometric mean at 6 months and the geometric mean at baseline. Significant changes between treatment arms were highlighted in bold characters. T tests were used to test the null hypothesis that regression coefficients were equal to 0 and obtained p-values reported. Satterthwaite approximation was used to estimate degrees of freedom.

Given the exploratory nature of these analyses, no adjustment for multiple comparisons was performed and nominal p-values were reported. Abbreviations: N: number of samples for analysis; MD = Mean Difference (ratio between mean ratio of FMT arm and SP arm); se = Standard Error; CI = Confidence Interval; SP: Sham Procedure; FMT: Faecal Microbiota Transplantation.

^§^ these values couldn’t be calculated as only one observation per SP arm was available.

^#^these values couldn’t be calculated as concentrations were under the lower limit of quantification (LLOQ) of the assay.

#### Supplementary Table 31. Average monthly changes of faecal cytokines in the SP and FMT arms, and their ratio over time (estimated from baseline to month 6). Intention-to-treat analysis

|  |  | **Estimate** | **Std· Error** | **95% Conf· Int·** | | **P value** |
| --- | --- | --- | --- | --- | --- | --- |
| IL-1β | Mean ratio between FMT and SP | 0·68 | 2·77 | 0·09 | 5·00 | 0·71 |
|  | Monthly variation in SP arm (mean ratio) | 0·89 | 1·22 | 0·61 | 1·30 | 0·55 |
|  | Monthly variation in FMT arm (mean ratio) | 0·92 | 1·14 | 0·71 | 1·20 | 0·54 |
|  | Mean ratio of monthly variation between FMT and SP | 1·03 | 1·27 | 0·65 | 1·64 | 0·89 |
| IL-2 | Mean ratio between FMT and SP | 1·80 | 2·49 | 0·30 | 10·72 | 0·52 |
|  | Monthly variation in SP arm (mean ratio) | 0·89 | 1·18 | 0·64 | 1·23 | 0·49 |
|  | Monthly variation in FMT arm (mean ratio) | 0·89 | 1·12 | 0·71 | 1·12 | 0·32 |
|  | Mean ratio of monthly variation between FMT and SP | 1·00 | 1·23 | 0·67 | 1·49 | 0·99 |
| IL-5 | Mean ratio between FMT and SP | 0·49 | 1·57 | 0·20 | 1·20 | 0·13 |
|  | Monthly variation in SP arm (mean ratio) | 0·98 | 1·01 | 0·96 | 1·00 | 0·084 |
|  | Monthly variation in FMT arm (mean ratio) | 1·00 | 1·01 | 0·99 | 1·01 | 1·00 |
|  | Mean ratio of monthly variation between FMT and SP | 1·02 | 1·01 | 0·99 | 1·05 | 0·14 |
| IL-6 | Mean ratio between FMT and SP | 2·10 | 1·8 | 0·66 | 6·66 | 0·22 |
|  | Monthly variation in SP arm (mean ratio) | 1·00 | 1·13 | 0·79 | 1·26 | 1·00 |
|  | Monthly variation in FMT arm (mean ratio) | 0·9 | 1·09 | 0·77 | 1·06 | 0·21 |
|  | Mean ratio of monthly variation between FMT and SP | 0·9 | 1·15 | 0·68 | 1·19 | 0·47 |
| IL-8 | Mean ratio between FMT and SP | 1·91 | 1·63 | 0·73 | 4·98 | 0·20 |
|  | Monthly variation in SP arm (mean ratio) | 1·31 | 1·10 | 1·08 | 1·59 | 0·0091 |
|  | Monthly variation in FMT arm (mean ratio) | 1·01 | 1·07 | 0·89 | 1·16 | 0·84 |
|  | Mean ratio of monthly variation between FMT and SP | 0·77 | 1·13 | 0·61 | 0·98 | 0·04 |
| IL-10 | Mean ratio between FMT and SP | 1·49 | 1·84 | 0·45 | 4·90 | 0·52 |
|  | Monthly variation in SP arm (mean ratio) | 0·96 | 1·12 | 0·76 | 1·20 | 0·71 |
|  | Monthly variation in FMT arm (mean ratio) | 0·93 | 1·08 | 0·79 | 1·09 | 0·37 |
|  | Mean ratio of monthly variation between FMT and SP | 0·97 | 1·15 | 0·73 | 1·28 | 0·84 |
| IL-12p70 | Mean ratio between FMT and SP | 1·43 | 2·14 | 0·32 | 6·40 | 0·64 |
|  | Monthly variation in SP arm (mean ratio) | 1·19 | 1·15 | 0·90 | 1·57 | 0·23 |
|  | Monthly variation in FMT arm (mean ratio) | 1·00 | 1·10 | 0·83 | 1·21 | 0·99 |
|  | Mean ratio of monthly variation between FMT and SP | 0·84 | 1·19 | 0·60 | 1·18 | 0·33 |
| IL-13 | Mean ratio between FMT and SP | 1·41 | 1·60 | 0·56 | 3·54 | 0·47 |
|  | Monthly variation in SP arm (mean ratio) | 1·13 | 1·10 | 0·94 | 1·36 | 0·21 |
|  | Monthly variation in FMT arm (mean ratio) | 0·94 | 1·07 | 0·83 | 1·07 | 0·39 |
|  | Mean ratio of monthly variation between FMT and SP | 0·84 | 1·12 | 0·67 | 1·05 | 0·13 |
| IL-15 | Mean ratio between FMT and SP | 1·00 | 2·97 | 0·12 | 8·46 | 1·00 |
|  | Monthly variation in SP arm (mean ratio) | 1·00 | 1·25 | 0·65 | 1·55 | 1·00 |
|  | Monthly variation in FMT arm (mean ratio) | 1·33 | 1·16 | 0·99 | 1·80 | 0·070 |
|  | Mean ratio of monthly variation between FMT and SP | 1·33 | 1·31 | 0·79 | 2·26 | 0·29 |
| IL-17 | Mean ratio between FMT and SP | 1·53 | 2·10 | 0·36 | 6·54 | 0·57 |
|  | Monthly variation in SP arm (mean ratio) | 0·98 | 1·17 | 0·72 | 1·33 | 0·90 |
|  | Monthly variation in FMT arm (mean ratio) | 1·04 | 1·11 | 0·85 | 1·28 | 0·69 |
|  | Mean ratio of monthly variation between FMT and SP | 1·06 | 1·21 | 0·74 | 1·54 | 0·74 |
| G-CSF | Mean ratio between FMT and SP | 0·78 | 2·90 | 0·10 | 6·26 | 0·81 |
|  | Monthly variation in SP arm (mean ratio) | 1·44 | 1·27 | 0·91 | 2·3 | 0·13 |
|  | Monthly variation in FMT arm (mean ratio) | 1·00 | 1·16 | 0·74 | 1·33 | 0·98 |
|  | Mean ratio of monthly variation between FMT and SP | 0·69 | 1·32 | 0·40 | 1·19 | 0·20 |
| GM-CSF | Mean ratio between FMT and SP | 1·13 | 2·51 | 0·19 | 6·85 | 0·90 |
|  | Monthly variation in SP arm (mean ratio) | 0·79 | 1·21 | 0·54 | 1·15 | 0·23 |
|  | Monthly variation in FMT arm (mean ratio) | 0·99 | 1·14 | 0·77 | 1·28 | 0·97 |
|  | Mean ratio of monthly variation between FMT and SP | 1·26 | 1·26 | 0·8 | 1·99 | 0·32 |
| IFN-γ | Mean ratio between FMT and SP | 0·95 | 1·91 | 0·27 | 3·39 | 0·94 |
|  | Monthly variation in SP arm (mean ratio) | 1·07 | 1·14 | 0·83 | 1·37 | 0·63 |
|  | Monthly variation in FMT arm (mean ratio) | 1·00 | 1·09 | 0·84 | 1·19 | 0·97 |
|  | Mean ratio of monthly variation between FMT and SP | 0·94 | 1·17 | 0·69 | 1·28 | 0·68 |
| MCP-1 | Mean ratio between FMT and SP | 1·7 | 2·11 | 0·39 | 7·34 | 0·48 |
|  | Monthly variation in SP arm (mean ratio) | 0·89 | 1·15 | 0·68 | 1·18 | 0·44 |
|  | Monthly variation in FMT arm (mean ratio) | 0·90 | 1·10 | 0·74 | 1·09 | 0·28 |
|  | Mean ratio of monthly variation between FMT and SP | 1·00 | 1·19 | 0·71 | 1·41 | 0·99 |
| MIP-1α | Mean ratio between FMT and SP | 1·39 | 1·53 | 0·60 | 3·21 | 0·45 |
|  | Monthly variation in SP arm (mean ratio) | 1·00 | 1·09 | 0·84 | 1·19 | 1·00 |
|  | Monthly variation in FMT arm (mean ratio) | 1·10 | 1·06 | 0·98 | 1·24 | 0·11 |
|  | Mean ratio of monthly variation between FMT and SP | 1·10 | 1·11 | 0·90 | 1·36 | 0·36 |
| MIP-1β | Mean ratio between FMT and SP | 1·00 | 1·63 | 0·38 | 2·61 | 1·00 |
|  | Monthly variation in SP arm (mean ratio) | 1·00 | 1·10 | 0·83 | 1·21 | 1·00 |
|  | Monthly variation in FMT arm (mean ratio) | 1·16 | 1·07 | 1·01 | 1·32 | 0·04 |
|  | Mean ratio of monthly variation between FMT and SP | 1·16 | 1·13 | 0·92 | 1·46 | 0·22 |
| TNF-α | Mean ratio between FMT and SP | 0·97 | 2·68 | 0·14 | 6·68 | 0·97 |

|  | Monthly variation in SP arm (mean ratio) | 0·95 | 1·19 | 0·67 | 1·34 | 0·78 |
| --- | --- | --- | --- | --- | --- | --- |
|  | Monthly variation in FMT arm (mean ratio) | 0·93 | 1·13 | 0·74 | 1·18 | 0·57 |
|  | Mean ratio of monthly variation between FMT and SP | 0·98 | 1·24 | 0·65 | 1·49 | 0·93 |

For these analyses, laboratory parameters were scaled using a logarithmic transformation, to handle their strongly left-skewed distributions. The term "mean ratio" indicates the ratio of the geometric mean of the FMT arm and the geometric mean of the SP arm. Monthly variations were computed between 0 and 6 months. Monthly variations and differences between monthly variations were estimated using linear mixed-effects models with random intercept. These models included as covariates arm, time and the interaction arm x time. T tests were used to test the null hypothesis that regression coefficients were equal to 0 and obtained p-values reported. Satterthwaite approximation was used to estimate degrees of freedom.

Abbreviations: IL: interleukin, TNFalpha: tumour necrosis factor-alpha; GM-CSF: granulocyte-macrophage colony-stimulating factor; G-CSF: granulocyte colony-stimulating factor; MCP: monocyte-chemoattactant factor; MIP-1α: macrophage inflammatory protein-1-alpha; MIP-1β: macrophage inflammatory protein-1-beta; VEGF: vascular endothelial growth factor; ICAM-1: Intercellular Adhesion Molecule 1; CD62E: CD62 antigen-like family member E (E-selectin). FMT: faecal microbiota transplantation; SP: Sham Procedure.

#### Supplementary Table 32. Mean changes in serum free fatty acids from baseline to month 6 and month 12 between FMT and SP arms.

| **Serum free fatty acid** | Monthly variation | **Estimate** | **Std·**  **Error** | **95% CI** | | **P value** |
| --- | --- | --- | --- | --- | --- | --- |
| Acetic (μmol/L) | Mean difference between FMT and SP (baseline) | 0·51 | 16·15 | -31·14 | 32·15 | 0·97 |
|  | Mean variation between T6 and T0 in SP group | -30·28 | 18·88 | -67·28 | 6·73 | 0·11 |
|  | Mean variation between T12 and T0 in SP group | -8·57 | 22·52 | -52·72 | 35·57 | 0·70 |
|  | Mean difference in variation T6-T0 between FMT and SP | -2·09 | 22·80 | -46·77 | 42·59 | 0·93 |
|  | Mean difference in variation T12-T0 between FMT and SP | 11·58 | 26·72 | -40·79 | 63·94 | 0·67 |
| Propionic (μmol/L) | Mean difference between FMT and SP (baseline) | -1·15 | 0·99 | -3·09 | 0·79 | 0·25 |
|  | Mean variation between T6 and T0 in SP group | -4·17 | 1·07 | -6·27 | -2·07 | <0·0001 |
|  | Mean variation between T12 and T0 in SP group | -1·16 | 1·28 | -3·68 | 1·36 | 0·37 |
|  | Mean difference in variation T6-T0 between FMT and SP | 2·44 | 1·29 | -0·10 | 4·97 | 0·062 |
|  | Mean difference in variation T12-T0 between FMT and SP | 0·42 | 1·52 | -2·56 | 3·40 | 0·78 |
| isoButiric (μmol/L) | Mean difference between FMT and SP (baseline) | 1·16 | 1·88 | -2·51 | 4·84 | 0·54 |
|  | Mean variation between T6 and T0 in SP group | -0·87 | 1·94 | -4·67 | 2·93 | 0·66 |
|  | Mean variation between T12 and T0 in SP group | -4·75 | 2·33 | -9·31 | -0·19 | 0·043 |
|  | Mean difference in variation T6-T0 between FMT and SP | -1·89 | 2·34 | -6·47 | 2·70 | 0·42 |
|  | Mean difference in variation T12-T0 between FMT and SP | 2·69 | 2·76 | -2·71 | 8·09 | 0·33 |
| Butiric (μmol/L) | Mean difference between FMT and SP (baseline) | 0·31 | 1·88 | -3·37 | 3·99 | 0·87 |
|  | Mean variation between T6 and T0 in SP group | 0·13 | 2·14 | -4·07 | 4·32 | 0·95 |
|  | Mean variation between T12 and T0 in SP group | 15·91 | 2·56 | 10·90 | 20·93 | <0·0001 |
|  | Mean difference in variation T6-T0 between FMT and SP | 1·39 | 2·58 | -3·67 | 6·45 | 0·59 |
|  | Mean difference in variation T12-T0 between FMT and SP | -10·24 | 3·03 | -16·18 | -4·30 | 0·0010 |
| isoValeric (μmol/L) | Mean difference between FMT and SP(baseline) | 2·49 | 5·28 | -7·87 | 12·84 | 0·64 |
|  | Mean variation between T6 and T0 in SP group | -2·10 | 5·67 | -13·21 | 9·00 | 0·71 |
|  | Mean variation between T12 and T0 in SP group | -12·71 | 6·79 | -26·01 | 0·60 | 0·064 |
|  | Mean difference in variation T6-T0 between FMT and SP | -4·64 | 6·84 | -18·04 | 8·75 | 0·50 |
|  | Mean difference in variation T12-T0 between FMT and SP | 11·18 | 8·05 | -4·59 | 26·95 | 0·17 |
| 2MetilButiric (μmol/L) | Mean difference between FMT and SP (baseline) | 0·87 | 2·20 | -3·43 | 5·17 | 0·69 |
|  | Mean variation between T6 and T0 in SP group | -0·09 | 2·38 | -4·76 | 4·59 | 0·97 |
|  | Mean variation between T12 and T0 in SP group | -5·78 | 2·86 | -11·38 | -0·18 | 0·045 |
|  | Mean difference in variation T6-T0 between FMT and SP | -2·12 | 2·88 | -7·75 | 3·52 | 0·46 |
|  | Mean difference in variation T12-T0 between FMT and SP | 4·20 | 3·39 | -2·44 | 10·83 | 0·22 |
| Hexanoic (μmol/L) | Mean difference between FMT and SP (baseline) | 0·06 | 0·16 | -0·25 | 0·38 | 0·70 |
|  | Mean variation between T6 and T0 in SP group | 0·31 | 0·20 | -0·08 | 0·70 | 0·12 |
|  | Mean variation between T12 and T0 in SP group | -0·05 | 0·24 | -0·52 | 0·41 | 0·82 |
|  | Mean difference in variation T6-T0 between FMT and SP | -0·19 | 0·24 | -0·66 | 0·28 | 0·43 |
|  | Mean difference in variation T12-T0 between FMT and SP | 0·02 | 0·28 | -0·53 | 0·56 | 0·96 |
| Octanoic (μmol/L) | Mean difference between FMT and SP (baseline) | -8·54 | 6·87 | -22·00 | 4·91 | 0·21 |
|  | Mean variation between T6 and T0 in SP group | -4·56 | 8·68 | -21·57 | 12·45 | 0·60 |
|  | Mean variation between T12 and T0 in SP group | -15·93 | 10·23 | -35·98 | 4·12 | 0·12 |
|  | Mean difference in variation T6-T0 between FMT and SP | 14·42 | 10·49 | -6·15 | 34·99 | 0·17 |
|  | Mean difference in variation T12-T0 between FMT and SP | 8·46 | 12·16 | -15·38 | 32·29 | 0·49 |
| Decanoic (μmol/L) | Mean difference between FMT and SP (baseline) | -3·33 | 2·67 | -8·56 | 1·91 | 0·21 |
|  | Mean variation between T6 and T0 in SP group | -1·43 | 3·38 | -8·05 | 5·20 | 0·67 |
|  | Mean variation between T12 and T0 in SP group | -6·43 | 3·98 | -14·24 | 1·37 | 0·11 |
|  | Mean difference in variation T6-T0 between FMT and SP | 3·13 | 4·09 | -4·88 | 11·14 | 0·44 |
|  | Mean difference in variation T12-T0 between FMT and SP | 3·15 | 4·73 | -6·13 | 12·42 | 0·51 |
| Dodecanoic (μmol/L) | Mean difference between FMT and SP (baseline) | -0·12 | 0·41 | -0·92 | 0·68 | 0·77 |
|  | Mean variation between T6 and T0 in SP group | -0·91 | 0·44 | -1·77 | -0·05 | 0·041 |
|  | Mean variation between T12 and T0 in SP group | -0·72 | 0·53 | -1·75 | 0·31 | 0·17 |
|  | Mean difference in variation T6-T0 between FMT and SP | 0·22 | 0·53 | -0·82 | 1·26 | 0·68 |
|  | Mean difference in variation T12-T0 between FMT and SP | 0·61 | 0·63 | -0·62 | 1·83 | 0·33 |
| Tetradecanoic (μmol/L) | Mean difference between FMT and SP (baseline) | -4·35 | 3·07 | -10·36 | 1·66 | 0·16 |
|  | Mean variation between T6 and T0 in SP group | -11·82 | 3·44 | -18·56 | -5·08 | 0·0011 |
|  | Mean variation between T12 and T0 in SP group | -7·32 | 4·11 | -15·38 | 0·74 | 0·077 |
|  | Mean difference in variation T6-T0 between FMT and SP | 7·24 | 4·15 | -0·90 | 15·37 | 0·084 |
|  | Mean difference in variation T12-T0 between FMT and SP | 9·91 | 4·88 | 0·35 | 19·47 | 0·044 |
| Hexadecanoic (μmol/L) | Mean difference between FMT and SP (baseline) | -18·17 | 76·02 | -167·17 | 130·82 | 0·81 |
|  | Mean variation between T6 and T0 in SP group | -127·69 | 73·54 | -271·83 | 16·46 | 0·085 |
|  | Mean variation between T12 and T0 in SP group | 121·30 | 88·32 | -51·81 | 294·40 | 0·17 |
|  | Mean difference in variation T6-T0 between FMT and SP | 177·64 | 88·68 | 3·82 | 351·45 | 0·047 |
|  | Mean difference in variation T12-T0 between FMT and SP | 230·82 | 104·66 | 25·70 | 435·94 | 0·029 |
| Octadecanoic (μmol/L) | Mean difference between FMT and SP (baseline) | 19·07 | 77·81 | -133·43 | 171·57 | 0·81 |
|  | Mean variation between T6 and T0 in SP group | -67·39 | 73·18 | -210·82 | 76·03 | 0·36 |
|  | Mean variation between T12 and T0 in SP group | 260·34 | 87·91 | 88·04 | 432·64 | 0·0041 |
|  | Mean difference in variation T6-T0 between FMT and SP | 149·79 | 88·23 | -23·14 | 322·72 | 0·092 |
|  | Mean difference in variation T12-T0 between FMT and SP | 43·82 | 104·16 | -160·33 | 247·98 | 0·67 |

Mean variations and differences between mean variations between each timepoint (month 6 and month 12) and baseline in the two treatment arms were estimated by linear mixed-effect models with random intercept. These models included as covariates arm, time and the interaction arm x time. T tests were used to test the null hypothesis that regression coefficients were equal to 0 and obtained p-values reported. Satterthwaite approximation was used to estimate degrees of freedom.

Given the exploratory nature of these analyses, no adjustment for multiple comparisons was performed and nominal p-values were reported. Abbreviations: CI: confidence interval; FMT: faecal microbiota transplantation; SP: Sham Procedure, T6: month 6 from randomization, T12: month 12 from randomization, T0: baseline, SE: standard error.

#### Supplementary Table 33. Average monthly changes of serum free fatty acid in the SP and FMT arms, and their mean difference over time (estimated from baseline to month 12).

| **Serum free fatty acid** | Monthly variation | **Estimate** | **SE** | **95% CI** | | **P value** |
| --- | --- | --- | --- | --- | --- | --- |
| Acetic (μmol/L) | Monthly variation SP | -0·88 | 1·65 | -4·12 | 2·36 | 0·60 |
|  | Monthly variation FMT | -0·90 | 1·10 | -3·06 | 1·25 | 0·41 |
|  | Mean difference in monthly variation | -0·03 | 1·99 | -3·92 | 3·86 | 0·99 |
| Propionic (μmol/L) | Monthly variation SP | -0·17 | 0·10 | -0·36 | 0·02 | 0·075 |
|  | Monthly variation FMT | -0·06 | 0·06 | -0·18 | 0·07 | 0·37 |
|  | Mean difference in monthly variation | 0·12 | 0·12 | -0·11 | 0·35 | 0·32 |
| IsoButiric (μmol/L) | Monthly variation SP | -0·41 | 0·20 | -0·80 | -0·02 | 0·040 |
|  | Monthly variation FMT | -0·26 | 0·13 | -0·51 | 0·00 | 0·051 |
|  | Mean difference in monthly variation | 0·15 | 0·24 | -0·31 | 0·62 | 0·52 |
| Butiric (μmol/L) | Monthly variation SP | 1·03 | 0·28 | 0·49 | 1·57 | <0·0001 |
|  | Monthly variation FMT | 0·57 | 0·18 | 0·20 | 0·93 | 0·0032 |
|  | Mean difference in monthly variation | -0·46 | 0·33 | -1·12 | 0·19 | 0·16 |
| IsoValeric (μmol/L) | Monthly variation SP | -1·04 | 0·55 | -2·11 | 0·04 | 0·060 |
|  | Monthly variation FMT | -0·49 | 0·36 | -1·20 | 0·23 | 0·18 |
|  | Mean difference in monthly variation | 0·55 | 0·66 | -0·74 | 1·84 | 0·40 |
| 2MetilButiric (μmol/L) | Monthly variation SP | -0·43 | 0·22 | -0·86 | 0·01 | 0·060 |
|  | Monthly variation FMT | -0·25 | 0·15 | -0·54 | 0·04 | 0·10 |
|  | Mean difference in monthly variation | 0·18 | 0·27 | -0·35 | 0·70 | 0·51 |
| Hexanoic (μmol/L) | Monthly variation SP | 0·00 | 0·02 | -0·03 | 0·03 | 0·98 |
|  | Monthly variation FMT | -0·01 | 0·01 | -0·03 | 0·02 | 0·60 |
|  | Mean difference in monthly variation | -0·01 | 0·02 | -0·05 | 0·03 | 0·76 |
| Octacanoic (μmol/L) | Monthly variation SP | -1·01 | 0·75 | -2·48 | 0·45 | 0·18 |
|  | Monthly variation FMT | -0·38 | 0·50 | -1·36 | 0·59 | 0·44 |
|  | Mean difference in monthly variation | 0·63 | 0·90 | -1·13 | 2·39 | 0·48 |
| Decanoic (μmol/L) | Monthly variation SP | -0·42 | 0·29 | -0·98 | 0·14 | 0·14 |
|  | Monthly variation FMT | -0·20 | 0·19 | -0·57 | 0·18 | 0·31 |
|  | Mean difference in monthly variation | 0·23 | 0·34 | -0·45 | 0·90 | 0·51 |
| Dodecanoic (μmol/L) | Monthly variation SP | -0·08 | 0·04 | -0·15 | 0·00 | 0·040 |
|  | Monthly variation FMT | -0·01 | 0·03 | -0·06 | 0·04 | 0·62 |
|  | Mean difference in monthly variation | 0·07 | 0·05 | -0·02 | 0·16 | 0·15 |
| Tetradecanoic (μmol/L) | Monthly variation SP | -0·79 | 0·31 | -1·40 | -0·18 | 0·013 |
|  | Monthly variation FMT | 0·07 | 0·21 | -0·33 | 0·48 | 0·73 |
|  | Mean difference in monthly variation | 0·86 | 0·37 | 0·12 | 1·59 | 0·024 |
| Hexadecanoic (μmol/L) | Monthly variation SP | 7·71 | 6·88 | -5·78 | 21·20 | 0·26 |
|  | Monthly variation FMT | 22·28 | 4·55 | 13·36 | 31·21 | <0·0001 |
|  | Mean difference in monthly variation | 14·57 | 8·25 | -1·61 | 30·75 | 0·080 |
| Octadecanoid (μmol/L) | Monthly variation SP | 18·56 | 6·98 | 4·89 | 32·24 | 0·0091 |
|  | Monthly variation FMT | 20·24 | 4·61 | 11·20 | 29·29 | <0·0001 |
|  | Mean difference in monthly variation | 1·68 | 8·36 | -14·71 | 18·07 | 0·84 |

Serum free fatty acids were measured at baseline, month 3, month 6, month 9 and month 12. Monthly variations and differences between monthly variations in the two treatment arms were estimated by linear mixed-effect models with random intercept, which included all available repeated measures for each patient. These models included as covariates arm, time and the interaction arm x time. T tests were used to test the null hypothesis that regression coefficients were equal to 0 and obtained p-values reported. Satterthwaite approximation was used to estimate degrees of freedom. Significant variations over time were highlighted in bold character.

Abbreviations: CI: confidence interval; FMT: faecal microbiota transplantation; SP: Sham Procedure, T6: month 6 from randomization, T12: month 12 from randomization, T0: baseline, SE: standard error.

#### Supplementary Table 34: Relative abundances at Phylum level at baseline and at month 6, in the FMT and SP arms.

|  | **FMT** | | | **SP** | | |
| --- | --- | --- | --- | --- | --- | --- |
| **Phylum** | **T0**  **Mean;median [IQR]** | **T6 Mean;median [IQR]** | **p value** | **T0**  **Mean;median [IQR]** | **T6**  **Mean;median [IQR]** | **p value** |
| **Actinobacteria** | 0·053; 0·035 [0·020–0·053] | 0·134; 0·049 [0·026–0·110] | 0·0001 | 0·074; 0·058 [0·025–0·078] | 0·065; 0·039 [0·024–0·101] | 0·687 |
| Bacteroidetes | 0·404; 0·402 [0·322–0·467] | 0·337; 0·324 [0·258–0·496] | 0·004 | 0·366; 0·376 [0·294–0·422] | 0·356; 0·350 [0·294–0·385] | 0·312 |
| **Firmicutes** | 0·491; 0·493 [0·472–0·537] | 0·492; 0·525 [0·420–0·612] | 0·438 | 0·502; 0·489 [0·431–0·584] | 0·526; 0·572 [0·451–0·601] | 0·437 |
| **Fusobacteria** | 0·00002; 0·00000 [0·00000–0·00002] | 0·00004; 0·00000 [0·00000–0·00002] | 0·201 | 0·00093; 0·00000 [0·00000–0·00004] | 0·00001; 0·00002 [0·00000–0·00002] | 0·583 |
| **Proteobacteria** | 0·030; 0·019 [0·004–0·030] | 0·017; 0·012 [0·004–0·026] | 0·0032 | 0·056; 0·036 [0·012–0·063] | 0·049; 0·017 [0·009–0·065] | 0·218 |
| **Tenericutes** | 0·0011; 0·0000 [0·0000–0·0000] | 0·00001; 0·0000 [0·0000–0·0000] | 0·181 | 0·00000; 0·0000 [0·0000–0·0000] | 0·00000; 0·0000 [0·0000–0·0000] | 1·000 |
| Verrucomicrobia | 0·0198; 0·00073 [0·00003–0·02705] | 0·0201; 0·00028 [0·00000–0·01881] | 0·0649 | 0·00135; 0·00111 [0·00036–0·00180] | 0·00366; 0·00135 [0·00096–0·00273] | 0·031 |

Data are reported as the mean, median and 25th–75th percentile. Paired Wilcoxon signed-rank test was used to assess the statistical significance among the two groups. Benjamini-Hochberg correction was applied for multiple comparisons.

#### Supplementary Table 35. Relative abundances at Genus level (top 20 genera) in SP and FMT arms

|  | **FMT arm** | | | **SP arm** | | |
| --- | --- | --- | --- | --- | --- | --- |
| **Genus** | **T0** | **T6** | **P values** | **T0** | **T6** | **P values** |
| Akkermansia | 0·0250; 0·0006  [0·0001-0·0375] | 0·0277; 0·0004  [<0·0001-0·0249] | 0·61 | 0·0019; 0·0017  [0·0005-0·0024] | 0·0048; 0·0021  [0·0012-0·0039] | 0·06 |
| Alistipes | 0·0572; 0·0459  [0·0218-0·0609] | 0·0581; 0·0502  [0·0329-0·0595] | 0·74 | 0·0464; 0·0358  [0·0294-0·0399] | 0·0750; 0·0548  [0·0475-0·0600] | 0·031 |
| Bacteroides | 0·2415; 0·1998  [0·1757-0·3133] | 0·2208; 0·1638  [0·1224-0·3205] | 0·018 | 0·1729; 0·1572  [0·0954-0·2419] | 0·1537; 0·1632  [0·0934-0·2090] | 0·44 |
| Barnesiella | 0·0373; 0·0229  [0·0045-0·0395] | 0·0144; 0·0106  [0·0047-0·0253] | 0·0010 | 0·0245; 0·0184  [0·0097-0·0359] | 0·0471; 0·0439  [0·0217-0·0751] | 0·06 |
| Bifidobacterium | 0·0217; 0·0105  [0·0013-0·0334] | 0·0453; 0·0188  [0·0020-0·0496] | 0·0009 | 0·0460; 0·0127  [0·0016-0·0384] | 0·0243; 0·0055  [0·0032-0·0250] | 0·44 |
| Blautia | 0·0590; 0·0404  [0·0272-0·0662] | 0·0390; 0·0402  [0·0185-0·0522] | 0·0017 | 0·0374; 0·0235  [0·0134-0·0429] | 0·0513; 0·0211  [0·0125-0·0305] | 0·44 |
| Catenibacterium | 0·0385; <0·0001  [<0·0001-0·0014] | 0·0369; <0·0001  [<0·0001-0·0022] | 0·10 | 0·0147; <0·0001  [<0·0001-<0·0001] | 0·0204; <0·0001  [<0·0001-0·0001] | 1·00 |
| Collinsella | 0·0208; 0·0080  [0·0001-0·0162] | 0·0219; 0·0167  [0·0043-0·0282] | 0·0052 | 0·0152; 0·0060  [0·0036-0·0208] | 0·0208; 0·0085  [0·0038-0·0122] | 0·56 |
| Faecalibacterium | 0·0495; 0·0321  [0·0110-0·0838] | 0·0410; 0·0343  [0·0107-0·0533] | 0·16 | 0·0524; 0·0710  [0·0290-0·0748] | 0·0387; 0·0288  [0·0231-0·0575] | 0·31 |
| Gemmiger | 0·0210; 0·0143  [0·0076-0·0332] | 0·0178; 0·0151  [0·0065-0·0240] | 0·015 | 0·0130; 0·0131  [0·0097-0·0167] | 0·0205; 0·0146  [0·0107-0·0294] | 0·06 |
| Lachnospiracea  incertae sedis | 0·0412; 0·0372  [0·0214-0·0549] | 0·0368; 0·0294  [0·0130-0·0602] | 0·021 | 0·0424; 0·0409  [0·0165-0·0604] | 0·0201; 0·0125  [0·0091-0·0334] | 0·031 |
| Oscillibacter | 0·0152; 0·0096  [0·0067-0·0127] | 0·0141; 0·0105  [0·0045-0·0177] | 0·60 | 0·0216; 0·0214  [0·0125-0·0308] | 0·0472; 0·0219  [0·0108-0·0485] | 0·69 |
| Parabacteroides | 0·0519; 0·0505  [0·0201-0·0674] | 0·0378; 0·0296  [0·0252-0·0542] | 0·0008 | 0·0361; 0·0385  [0·0335-0·0473] | 0·0326; 0·0355  [0·0299-0·0411] | 0·09 |
| Prevotella | 0·0131; 0·0001 [<0·0001-0·0035] | 0·0121; 0·0001 [<0·0001-0·0116] | 0·26 | 0·0646; 0·0179 [0·0038-0·1097] | 0·0358;0·0182 [0·0033-0·0372] | 0·56 |
| Ruminococcus | 0·0121; 0·0081  [0·0035-0·0144] | 0·0172; 0·0047  [0·0028-0·0263] | 0·60 | 0·0330; 0·0207  [0·0119-0·0414] | 0·0135; 0·0115  [0·0089-0·0148] | 0·031 |
| unclassified Bacteroidales | 0·0468; 0·0379 [0·0327-0·0565] | 0·0414; 0·0397 [0·0284-0·0566] | 0·025 | 0·0575; 0·0555 [0·0427-0·0641] | 0·0393; 0·0382 [0·0314-0·0496] | 0·031 |
| unclassified  Clostridiales | 0·0432; 0·0454  [0·0198-0·0559] | 0·0639; 0·0482  [0·0250-0·0740] | 0·0006 | 0·0650; 0·0750  [0·0464-0·0781] | 0·0791; 0·0643  [0·0481-0·1080] | 0·31 |
| unclassified Firmicutes | 0·0220; 0·0147 [0·0076-0·0235] | 0·0400; 0·0249 [0·0115-0·0456] | <0·0001 | 0·0237; 0·0228 [0·0153-0·0265] | 0·0285; 0·0240 [0·0185-0·0345] | 0·031 |
| unclassified  Lachnospiraceae | 0·1204; 0·1085  [0·0890-0·1581] | 0·1152; 0·1067  [0·0715-0·1689] | 0·11 | 0·1419; 0·1543  [0·1439-0·1572] | 0·1232; 0·1553  [0·0633-0·1663] | 0·84 |
| unclassified Ruminococcaceae | 0·0627; 0·0484 [0·0360-0·0731] | 0·0983; 0·0650 [0·0414-0·1224] | 0·0001 | 0·0899; 0·0937 [0·0710-0·1009] | 0·1241; 0·1017 [0·0732-0·1667] | 0·031 |

Data are reported as the mean, median and 25th–75th percentile. Paired Wilcoxon signed-rank test was used to assess the statistical significance among the two groups. Significant changes between treatment arms were highlighted in bold characters. Benjamini–Hochberg correction was appled for multiple comparisons.

# 3. Supplementary Figures

#### Supplementary Figure 1. Variations in Tregs over time in the two treatment arms. Circulating Tregs (% over CD4+ lymphocytes) are shown in panel A, and tissue Tregs from intestinal biopsies in panel B. In both panels thin lines represent individual participant trajectories and thick lines indicate group averages; blue lines indicate the FMT group, red lines the SP group; thick lines represent group averages.

#### Supplementary Figure 2. Variations in ALSFRS-R and FVC over time in the two treatment arms. Panel A shows individual and mean decline in ALSFRS-R scores over time. Panel B illustrates the decline in FVC over time. In both panels A and B thin lines represent individual participant trajectories and thick lines indicate group averages; blue lines indicate the FMT group, red lines the SP group; thick lines represent group averages.

#### Supplementary Figure 3. Variations in BMI and ALSAQ40 over time in the two treatment arms. Panel A shows monthly variations in BMI, and panel B reports changes in ALSAQ-40 scores. In both panels A and B, thin lines represent individual participant trajectories and thick lines indicate group averages; blue lines indicate the FMT group, red lines the SP group; thick lines represent group averages.

#### Supplementary Figure 4. Tracheostomy-free survival rates in the two treatment arms.

#### Blue lines indicate the FMT group, red lines the SP group.

**Supplementary Figure 5. Safety analyses up to 6 months.** Panel A shows the Kaplan–Meier estimates of time to first adverse event. Panel B shows the mean cumulative function (MCF) for adverse events (AEs), representing the cumulative number of AEs per participant over time and accounting for recurrent events and variable follow-up duration. Numbers at risk are reported below each panel. Blue lines indicate the FMT group and red lines the SP group.

**Supplementary Figure 6. Safety analyses over the full 12-month follow-up.** Panel A shows the Kaplan–Meier estimates of time to first adverse event. Panel B shows the mean cumulative function (MCF) for adverse events (AEs), representing the cumulative number of AEs per participant over time and accounting for recurrent events and variable follow-up duration. Numbers at risk are reported below each panel. Blue lines indicate the FMT group and red lines the SP group.

**Supplementary Figure 7: Variations in neurofilament over time in the two treatment arms.** Panel A and B show serum neurofilament heavy (NfH) and light (NfL) variations in FMT-treated and SP patients, and the average of each arm, in intention-to-treat population. Blue lines indicate the FMT group and red lines the SP group;thick lines represent group averages.

#### Supplementary Figure 8: Variations in T cells over time in the two treatment arms. Panel A and B show Circulating CD45+ T cells and CD4+ lymphocytes in FMT-treated and untreated patients, and the average of each arm, in intention-to-treat population. Blue lines indicate the FMT group, red lines the SP group; thick lines represent group averages.

**Supplementary Figure 9: Variations in the ratios of circulating T cells over time in the two treatment arms.** Panel A and B show The Treg/Th1 and Treg/Th17 ratios of circulating T cells in FMT-treated and untreated patients, and the average of each arm, in intention-to-treat population. Blue lines indicate the FMT group, red lines the SP group; thick lines represent group averages; thick lines represent group averages.

#### Supplementary Figure 10: Comparison of gut microbiota alpha diversity metrics at baseline and month 6 in SP and FMT arms.

**A**

**C**

**B**

Violin plots show the distribution of three alpha diversity indices: (a) Richness (Observed species), (b) Shannon diversity, and (c) Pielou’s Evenness, measured in stool samples collected at baseline (T0) and six-month follow-up (T1) from participants in the SP (SP Procedure, n=6) and FMT (Faecal Microbiota Transplantation, n=16) groups. Each black dot represents an individual sample, and lines connect paired samples across time points. Box plots within the violins indicate medians and interquartile ranges. Statistical comparisons between T0 and T1 within each group were performed by using the paired Wilcoxon signed-rank test; there were no significant differences (*p>0.05*).

#### Supplementary Figure 11: Comparison of Bray-Curtis beta diversity of gut microbiota composition in SP and FMT arms at baseline and month 6.

Principal Coordinate Analysis (PCoA) plot based on Bray–Curtis dissimilarity shows beta diversity of gut microbiota, measured at the genus level, in stool samples collected at baseline (T0) and six-month follow-up (T1) from participants in the SP (SP Procedure, n = 6, blue) and FMT (Faecal Microbiota Transplantation, n = 16, red) groups. Each point represents a sample, with shapes indicating time points (circles = T0, triangles = T1). Lines connect paired samples from the same individual. Ellipses represent 95% confidence intervals for each group and time point. PERMANOVA analyses based on Bray–Curtis distances showed no significant differences in microbial community composition between T0 and T1 within either group (SP: *R² = 0.045, F = 0.47, p = 0.865*; FMT: *R² = 0.025, F*

*= 0.78, p = 0.656*).

#### Supplementary Figure 12: Assessment of dispersion of gut microbiota communities at baseline (T0) and at month 6 (T1) in SP and FMT arms.

**a**

**c**

**b**

**d**

Dispersion analysis based on Bray-Curtis dissimilarity was performed to evaluate intra-group variability in microbial community structure at T0 and T1 in SP (a, b) and FMT (c, d) groups. Principal Coordinates Analysis (PCoA) plots display the spatial distribution of individual samples at the two time points (T0, black circles; T1, red triangles), with lines connecting each sample to the group centroid. Convex hulls delineate the area occupied by samples from each time point. Boxplots (b, d) report the distances of individual samples to the group centroid, providing a quantitative measure of within-group dispersion. Statistical significance of differences in dispersion between T0 and T1 was tested using a permutational analysis of multivariate dispersions (PERMDISP). No significant differences in dispersion were detected between time points in either group (SP: *F=0.315, p=0.631*; FMT: *F=0.499, p=0.516*), indicating comparable levels of within-group variability before and after treatment.

**Supplementary Figure 13: Relative abundance of the bacterial phyla at baseline and at month 6 in SP and FMT arms.**

Box plots showing the relative abundance of the most representative bacterial phyla detected at baseline (T0) and after the FMT intervention (T1). Data are expressed as relative abundance (%) of each phylum. Each coloured dot represents an individual sample, and lines connect paired samples across time points. Statistical analysis was performed using the Wilcoxon signed-rank test for paired data. The upper line plots represent SP group, while the lower line plots show FMT group. A statistically significant increase in Actinobacteria (***p < 0.001) and a significant decrease in Bacteroidetes and Proteobacteria were observed at month 6 compared with baseline. In the SP arm, Verrucomicrobia showed a significant increase over time Exact p-values are indicated in the corresponding panels. No other phyla displayed significant longitudinal changes.All results obtained analysis are shown in Supplementary table 34.

#### Supplementary Figure 14: Relative abundance of the top 20 genera at baseline and at month 6 in SP and FMT arms.

Box plots showing the relative abundance of the twenty most abundant bacterial genera detected at baseline (T0) and after intervention (T1). Data are reported as relative abundance (%) of each genus. Each coloured dot represents an individual sample, and lines connect paired samples across time points. Statistical analysis was performed using the Wilcoxon signed-rank test for paired data. Statistical comparisons between T0 and T1 were performed using the Wilcoxon signed-rank test for paired data. The upper two-line plots represent SP group, while the two lower line plots represent FMT group. In the SP arm, significant increases were observed for *Alistipes*, *unclassified Firmicutes*, and *unclassified Ruminococcaceae*, whereas *Lachnospiraceae* *incertae sedis*, *Ruminococcus*, and *unclassified Bacteroidales* significantly decreased. In the FMT arm, significant increases were observed for *Bifidobacterium*, *Collinsella*, *unclassified Firmicutes*, and *unclassified Ruminococcaceae*, whereas *Barnesiella*, *Blautia*, *Lachnospiraceae incertae sedis*, *Gemmiger*, and *Parabacteroides* significantly decreased. Exact p-values are indicated in the corresponding panels. All results obtained by analysis are shown in Supplementary table 35.

**Supplementary Figure 15. Differential abundance analysis of bacterial taxa at baseline and month 6 in FMT and SP arms.** Volcano plots showing bacterial taxa differentially abundant between baseline (T0) and month 6 (T1) in the SP (A) and FMT (B) groups, as identified by DESeq2 analysis. Each dot represents a bacterial taxon. The x-axis shows the magnitude and direction of change (log2FoldChange), with positive values indicating enrichment at T1 and negative values indicating enrichment at T0. The y-axis represents statistical significance (-log10 adjusted p-value). P-values were obtained using the Wald test implemented in DESeq2 and adjusted for multiple comparisons using the Benjamini–Hochberg method.

Taxa significantly enriched at baseline (T0) are shown in green, whereas taxa significantly enriched at month 6 (T1) are shown in red. Black dots represent taxa that did not meet the predefined significance criteria. Overall, a greater number of taxa displayed significant longitudinal changes in the FMT arm compared with the SP arm, although the direction and magnitude of these changes varied across taxa. Dashed vertical and horizontal lines indicate the thresholds used to define differential abundance (|log2FoldChange| > 1 and adjusted p < 0.05).

#### Supplementary Figure 16. Microbial community composition differences across donors and SP and FMT groups.

**a**

**b**

Panel a: Principal Coordinate Analysis (PCoA) based on Bray-Curtis distances showing the beta-diversity of gut microbiota among donor (green circles), FMT (red), and SP (blue) groups at different time points: baseline (T0, triangles), and post-treatment (T1, diamonds). Each point represents a sample, and lines connect individual samples to their respective group centroid, illustrating group dispersion. Panel b: Pairwise PERMANOVA results comparing microbial community composition between groups. Each square represents an R² value (effect size) and FDR-adjusted p-value (False Discovery Rate) for the indicated group comparison. The intensity of the red to blue colour scale indicates the significance level of the comparisons, with red indicating significant differences (FDR < 0.05).
